# Anionic Polymerization of O‑Benzyl and O-*tert-*Butyldimethylsilyl Dienes Derived from Myrcene to Yield Functional Polyenes

**DOI:** 10.1021/acsomega.6c02544

**Published:** 2026-06-09

**Authors:** Joe Stanley, Rowan Radmall, William Pointer, Gary Walker, David M. Haddleton, Martin Wills

**Affiliations:** † Department of Chemistry, 2707 University of Warwick, Coventry CV4 7AL, U.K.; ‡ The Lubrizol Corporation, Hazelwood, Derby DE56 4AN, U.K.

## Abstract

We report a concise route to two functionalized myrcene-based monomers via ozonolysis of myrcene, followed by the addition of benzyl or *tert*-butyldimethylsilyl groups to protect the alcohol functionality, allowing these monomers to be polymerized by anionic polymerization (AP). Both protected monomers undergo controlled homopolymerization and copolymerization with isoprene in THF solution to form polymers containing varying levels of functional group incorporation. DOSY NMR was used to monitor chain propagation kinetics in cyclohexane solution, revealing that the addition of ca. 7 mol % of functionalized OBn diene into a copolymerization with isoprene was sufficient to change the microstructure of the resulting polymer from ca. 6.5% 3,4-polymer to ca. 30% 3,4- (the remainder being 1,4-polymer). Removal of the TBS group using TBAF, or palladium-catalyzed hydrogenation of the Bn group with concomitant reduction of the alkenes, provides access to unsaturated or saturated hydroxyl-functional polymers, respectively. This work establishes a convenient method to produce ether-containing monomers from myrcene and their use in AP.

## Graphical abstract

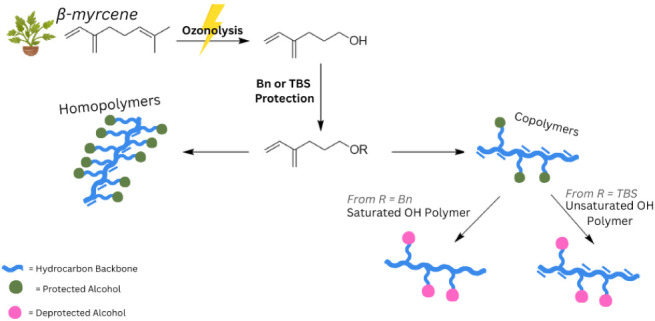

## Introduction

The reliance of polymer production on fossil fuels is a recognized issue, with oil sources depleting and the extraction of crude oil causing health, economic, and environmental problems.^1^ Biobased polymers, therefore, offer a more sustainable avenue for polymer synthesis.^2^ Obtaining monomers from biomass which feature diene moieties in their structure allows for sustainable anionic polymerization, which is already well regarded for its atom efficiency and rapid conversion rates.^3–6^

Myrcene (Figure 1, also known as β-myrcene), derived from biomass, contains three olefin bonds that exhibit very different reactivity, offering the opportunity to target different monomers by suitable functionalization.^7–9^ It is possible to modify polymyrcene, e.g., by incorporation of thiol groups^10,11^ or by subsequent epoxidation and further transformations. However, since the double bonds in both the polymer backbone and side chains are epoxidized, regiocontrol is challenging.^12–14^ An alternative approach to the incorporation of functionality in a diene polymer is to use a functionalized diene from the outset, resulting in the direct formation of either the required functional polymer or one that can subsequently be modified further. Examples include the use of radical and emulsion polymerization to prepare copolymers, resulting in hydroxy-containing dienes and other functionalized copolymers with myrcene,^15–17^ while cobalt- and neodymium-catalyzed methods have also been employed to copolymerize myrcene with hydroxy-functionalized dienes.^18,19^

**Figure 1:**
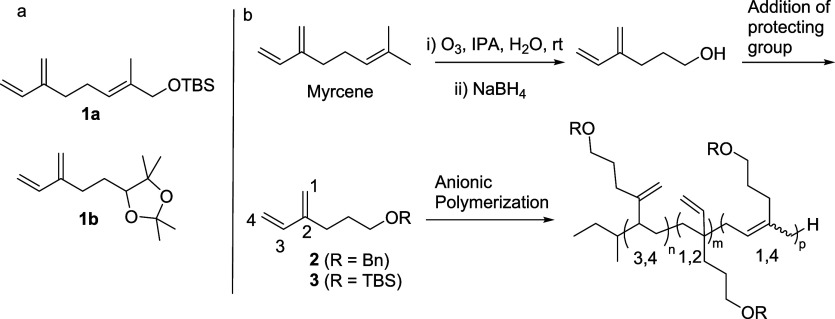
a. Myrcene-derived dienes reported by Frey et al.^34,35^ b. Summary of work in this paper: pathway from myrcene to polar monomers and subsequent polymerization.

Myrcene can be polymerized via anionic polymerization (AP)^20–22^ with alkyllithium initiation.^23–25^ In order to incorporate a functionalized diene monomer into a polyene, it must be compatible with the highly reactive alkyllithium initiator. Examples of this approach, using nonmyrcene-derived reagents, include the use of amine- and silyl-containing diene monomers.^26–32^ In some cases, elimination of the functional group can be a competing reaction; hence, the appropriate location of the group within the monomer is an important consideration.^33^ In a recent example of the preparation of a functionalized monomer derived from myrcene, Frey et al. used selenium dioxide to introduce a hydroxy group at the isolated alkene, which was then protected to create monomer **1a**, which was used successfully in anionic polymerization.^34^ Hence, the reaction of the electron-rich trisubstituted alkene in myrcene can be carried out selectively, leaving the conjugated diene intact to give a functionalized isoprene with potential for use in anionic polymerization (AP). In a further example, Frey et al. reported a protected diol derivative of myrcene, **1b**, which was also successfully converted into a functionalized polymer.^35^ In extensive studies of their copolymerization reactivity in AP with myrcene in hexane solution, polymers in which the proportion of 3,4-product increased from ca. 6% to ca. 30% were formed as the proportion of either **1a** or **1b** was raised from 0 to 100%. This reflects the behavior observed when a coordinating solvent such as THF is added to the reactions, indicating that the ether groups in both functionalized monomers act in a similar coordinating manner.^33,34^ A difference between the two monomers, however, was that **1a**, protected by the more hindered TBS group, reacted at a rate similar to that of myrcene and gave a random copolymer with functionalized groups distributed evenly along the chain.^33^ In contrast, **1b** was fully incorporated into the polymer at an earlier stage than myrcene.^34^

While the methods described above provide efficient routes to oxygen-containing diene monomers for LAP, the use of selenium dioxide, while catalytic in use, requires the removal of this potentially harmful metal from the monomer. In the second synthetic approach, an equivalent of meta-chloroperoxybenzoic acid (mCPBA) is required in the initial functionalization of myrcene, also requiring separation from the monomer. It is therefore desirable to have alternative approaches to useful functionalized monomers for this valuable application through milder routes. Furthermore, to the best of our knowledge, no anionic polymerization method has been reported that enables the incorporation of OH groups along the poly­(diene) backbone with selective control over backbone saturation, affording either saturated or unsaturated hydroxy-functional polymers. In this paper, we provide a method for achieving this.

Myrcene can react selectively via ozonolysis of the trisubstituted alkene.^10^ Depending on the workup, the intermediate ozonide can be transformed into a range of different functional groups.^36,37^ In this work (Figure 1), the ozonolysis of myrcene is followed by reductive workup using NaBH_4_, giving an intermediate primary alcohol that was subsequently further functionalized. Ozonolysis, leading to oxidative cleavage, was first explored in detail by Harries^38–43^ around half a century after the recognition of ozone as a chemical.^44–46^ Since ozonolysis of myrcene leads to the incorporation of oxygen into monomers, it is an effective way to introduce polar functionality into resulting polymer chains. This polarity is useful in the synthesis and production of dispersants,^47^ stimuli-sensitive cross-linked polymers,^48,49^ and thermoresponsive materials, for example.^50^ Recent market trends display the economic importance of copolymers, as their global consumption continues to rise.^1–5,36,37,51,52^

In the current work, we have employed ozonolysis to form two functionalized derivatives of myrcene, a biorenewable monomer, using air as an oxygen source and an electrically powered ozone generator. We subsequently demonstrated the successful homopolymerization of each monomer and the synthesis of functionalized copolymers with isoprene. Furthermore, we have employed in situ DOSY NMR spectroscopy to follow selected polymerizations in real time. Throughout the work, we also demonstrate that carbanionic polymerizations can be achieved using standard Schlenk line apparatus without the requirement of a glovebox to form polymers with satisfactory properties.

Anionic polymerization must be carried out under anhydrous and proton-free conditions. Therefore, alcohols in monomers must be protected, for example, using benzyl (Bn) and *tert*-butyldimethylsilyl (TBS) groups.^53^ These two protecting groups can be removed under complementary conditions, resulting in different polymer products. With three carbons between the alkoxy group and the diene, this would also circumvent any concerns that have been addressed around elimination.^33^ Benzyl ethers are typically deprotected by catalytic hydrogenation,^54,55,56^ which can lead to the formation of fully saturated polymer backbones if selective conditions are not used.^57^ TBS protecting groups are typically removed using a source of fluoride, commonly provided in the form of tetrabutylammonium fluoride (TBAF),^58^ which would leave the polymer backbone unsaturation intact.^34^ The current work focused on the potential of these to make new polymers and materials from a biorenewable monomer; the detailed kinetics and reactivity ratios will be the focus of future work.

## Results and Discussion

As the presence of moisture/protons in low ppm quantities leads to the premature termination of propagating polymer chains, rigorous drying of solvents, monomer solutions, and glassware was carried out to limit unwanted side reactions.^57,59^ All solvents and monomers were dried over 4 Å molecular sieves. Isoprene was dried neat, with **2** and **3** dried as 200 mg/mL solutions in cyclohexane. All monomers and solvents were taken to <10 ppm water^60^ as determined by Karl Fischer titration, prior to use. Myrcene was subject to ozonolysis (Scheme 1) for 4 h while stirring at room temperature on a 5 g scale using previously published methods, using air as the source of oxygen.^61,62^ The reaction was carried out until no myrcene was visible by thin layer chromatography (TLC).

**Scheme 1:**
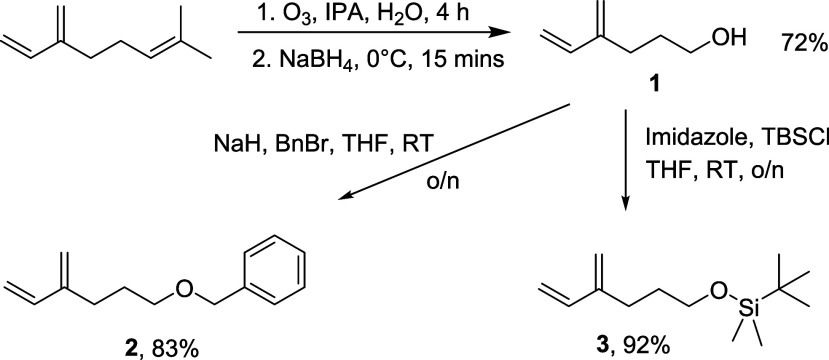
Synthesis of Functionalized Monomers **2** and **3**

The alcohol-containing product was then subjected to standard protecting group chemistry using both TBS and Bn protecting groups to yield the monomers **2** (OBn) and **3** (OTBS), respectively (Scheme 1).^62,63^ Protected monomers **2** and **3** were readily purified by column chromatography on silica gel. Both **2** and **3** are prone to autopolymerization when standing for long periods of time without an added antioxidant/stabilizer. When left at room temperature for 2 weeks, **2** turned into a white, stretchy material and **1** turned into a thick, light orange, moldable gel. Thus, the addition of antioxidants is required if these molecules are to be stored for any length of time.

### Anionic Homopolymerization of Myrcene Derivatives **2** and **3**

To test monomers **2** and **3** in anionic polymerizations, low-molecular-weight homopolymers, using a 10:1 ratio of diene:base initiator, were synthesized using *sec*-butyllithium (sBuLi) as an initiator and a combination of tetrahydrofuran (THF) and cyclohexane as the solvent (monomer stock solutions were prepared in cyclohexane). As expected of anionic diene polymerizations in the presence of THF, the reactions progressed with the full consumption of the monomers. The polyene microstructure was calculated by ^1^H NMR spectroscopy^64^ (Figure 2, Table 1, Supporting Information, Section 8).

**Figure 2:**
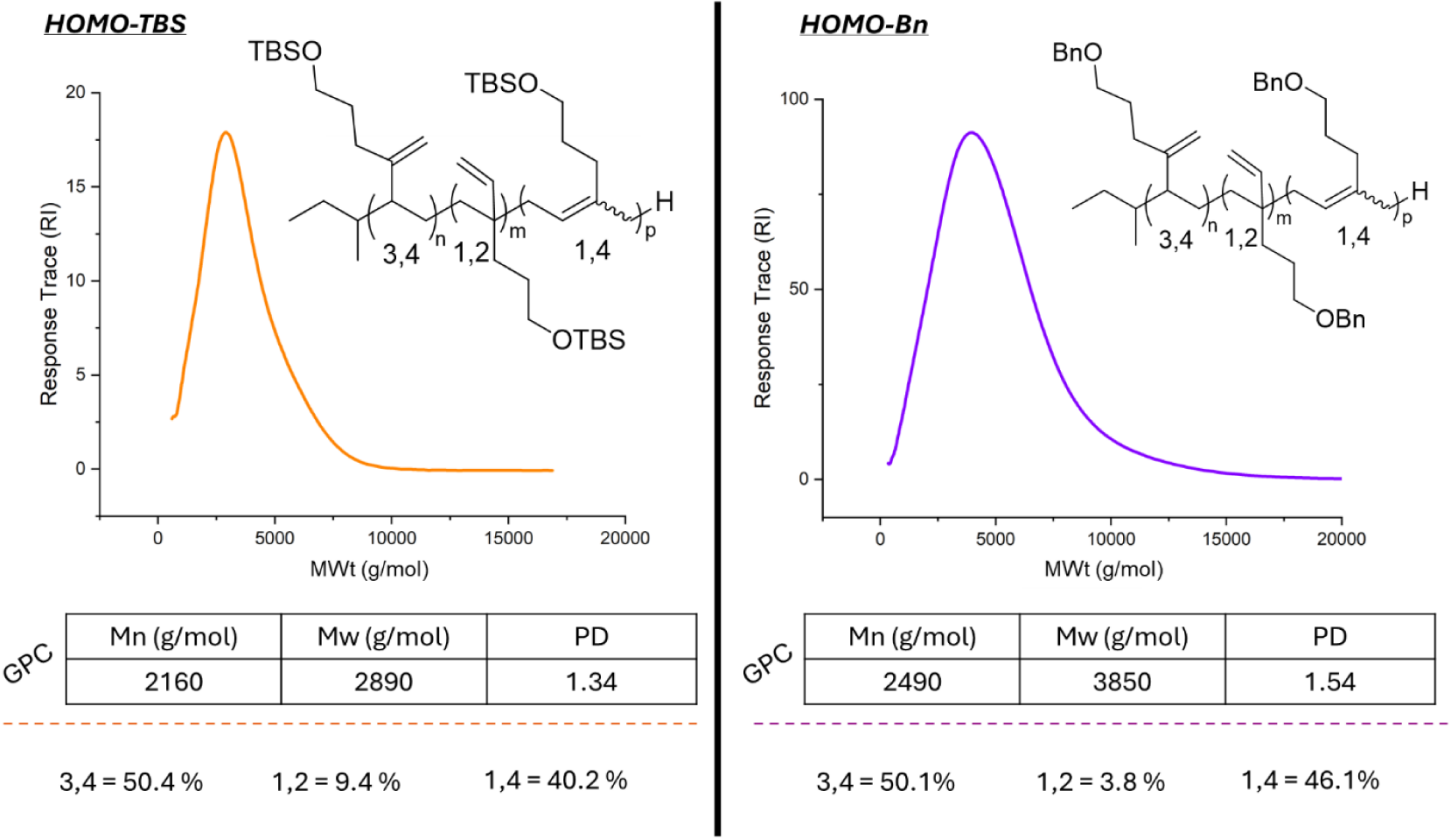
Characterization of polymers formed by homopolymerization of dienes **2** and **3**; gel permeation chromatography (GPC) of Homo-TBS and Homo-Bn polymers formed by AP of **2** and **3,** respectively, initiated using sBuLi in THF. Gel permeation chromatography (GPC) conditions: eluent = THF, mixed E column (see Supporting Information), microstructure information obtained by ^1^H NMR (CDCl_3_).

**Table 1:**
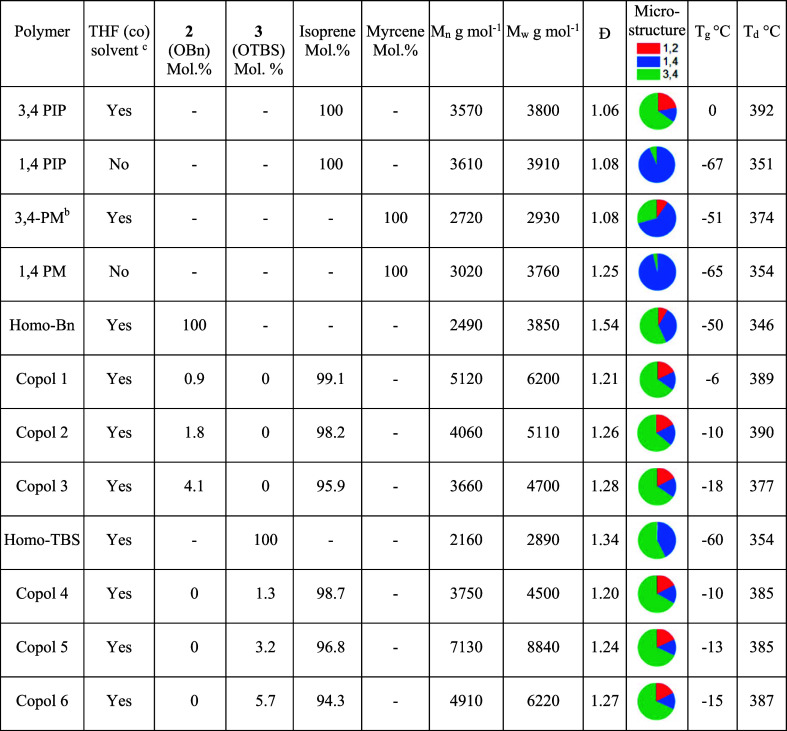
Monomer Content of Polymers Synthesized by AP in a Batch Process^a^

a PM = polymyrcene, PIP = polyisoprene. Number-average molecular weight (*M*_n_), weight-average molecular weight (*M*_w_), and dispersity (Đ) from GPC. Microstructure values were obtained by ^1^H NMR (CDCl_3_)—see the Supporting Information (ESI section 8) for a table of these values. Glass transition temperatures (*T*_g_) were obtained by DSC. Thermal decomposition temperatures (*T*_d_) were found through TGA. This resultant distribution of microstructures by this synthetic method aligns with published values.^64^

b Although the major microstructure is 1,4-polymer, it is termed ‘3,4-’ to distinguish it from the product formed in cyclohexane.

c “Yes” indicates that THF is present in the reaction mixture as a cosolvent. The 1,4-PIP and 1,4-PM entries were synthesized in 100% cyclohexane, while 3,4-PIP and 3,4-PM were synthesized in 100% THF. All other entries labeled “Yes” were performed in THF/cyclohexane mixtures, resulting from the addition of monomer stock solutions (200 mg mL^–1^ in cyclohexane) to THF. Approximate volume ratios were THF:cyclohexane ≈ 1:2 for homopolymerizations (Homo-Bn and Homo-TBS) and from 7.9:1.0 to 1.42:1 for copolymerizations (Copol 1–6). Full details are in the Supporting Information.

The resulting polymers exhibited molecular weights slightly higher than the target molecular weight (ca. 2000–2400 g mol^–1^). It is noted that systematic errors in MWt such as a variation in the concentration of sBuLi and the calibration method for the GPC can influence MWt measurements.^34^ The monomer conversion in both cases was complete, however. The GPCs of the homopolymers indicated a relatively high Đ for the Homo-Bn polymer, indicating a potential influence of the side chain functionality.^34,35^ In contrast (see below and Table 1), polymerizations of myrcene and isoprene alone, and copolymerizations of **2** and **3** with isoprene, were achieved with lower dispersity, more in line with what has previously been reported. The novel homopolymers of **2** and **3** formed in THF contained similar microstructures, with a predominant 3,4-polymerization in each case (ca. 57%), as expected with the polar THF solvent. For the Homo-TBS, almost all the remaining polymer (ca. 42%) was the 1,4-microstructure, whereas for the Homo-Bn, ca. 8% of 1,2-polymer (35% 1,4-) was also present. Comparisons with control reactions of polymerization of myrcene and isoprene alone (Table 1) under the same conditions revealed that the homopolymerizations using functionalized monomers **2** and **3** exhibited microstructures more similar to that of polyisoprene: 1,2-:1,4-:3,4- in ca. 22:13:65 ratio, whereas for myrcene, the ratio was ca. 9:61:30.^64^ The glass transition temperatures (*T*_g_) of the homopolymers were −50 °C (Homo-Bn) and −60 °C (Homo-TBS), respectively, similar to that of polymyrcene (−51 °C) but significantly lower than that of polyisoprene (ca. 0 °C) formed under the same conditions, also possibly reflecting the longer side chains in the new polymers. Both myrcene and isoprene give predominantly 1,4-polymers when polymerized in the low-polarity solvent, cyclohexane, as expected (Table 1).

### Batch Anionic Copolymerization of Myrcene Derivatives **2** and **3** with Isoprene

Copolymers of myrcene derivatives with isoprene have the potential to be interesting materials, with increased affinity for filler materials, for example,^18^ and tunable glass transition temperatures. Copolymers with a range of monomer compositions of both **2** and **3** with isoprene were synthesized and characterized by NMR, GPC, differential scanning calorimetry (DSC), and thermogravimetric analysis (TGA) (Table 1). The microstructures of the polymers were determined by ^1^H NMR, according to published studies.^64^ These polymers were synthesized on <300 mg scales using sBuLi as an initiator (see Table 1 for solvent information).

Polymerizations containing THF as a cosolvent gave copolymers with a predominantly 3,4-microstructure, reflecting the results with isoprene alone under these conditions. In anionic diene polymerizations, THF needs to be present only in very small amounts (corresponding to that introduced with the initiator solution) to exert a strong accelerating effect on the propagation rate and to increase the 3,4-microstructure content.^65–67^ In our reactions, however, all batch copolymerizations were allowed to proceed for 15 h prior to quenching to ensure total monomer consumption. Conversely, polymerizations conducted in nonpolar cyclohexane were much slower, giving higher levels of 1,4-microstructure. These relationships between the microstructure and reaction conditions in AP have previously been noted in numerous literature examples.^57,66–70^ All of the copolymers with isoprene gave similar ratios of polymer microstructure: ca. 17–18% 1,2-, 14–19% 1,4-, and 63–68% 3,4-, which is similar to that of isoprene alone when polymerized in THF (21.8, 12.8, 65.3%, respectively), reflecting the dominance of the isoprene monomer and the solvent in the control of the microstructures of these polymers.^66,71–75^ However, subsequent DOSY studies, which were carried out in cyclohexane (see below), indicate a different outcome in nonpolar solvents; notable in these later results is a significant change in microstructure for copolymers containing a small percentage of functionalized diene compared to pure isoprene.

The *T*_g_ values for all the copolymers were in the range of −6 to −18 °C, which decreased steadily as the proportion of functionalized polymer changed from ca. 1 mol % to a maximum of ca. 6 mol %. These values are below *T*_g_ of 3,4-polyisoprene (0 °C) but significantly above the homopolymer *T*_g_ values which were in the range of −50 to −60 °C. In contrast, the introduction of the functionalized monomers does not significantly alter *T*_d_ (decomposition temperatures) of the polymers, which are all >350 °C.

### DOSY NMR Reaction Monitoring of Copolymerizations in Cyclohexane

Polymer growth kinetics and microstructure can often be followed in real time using ^1^H NMR,^34,35,75,76^ and we used diffusion-ordered NMR spectroscopy (DOSY-NMR) on a benchtop NMR machine as a convenient method.^77,78^ Benchtop NMR allows for the collection of DOSY spectra to be observed in real time, with measurements typically taking under 5 min. Cyclohexane was chosen as a solvent system for this study due to the slow propagation kinetics, giving polymerization times of several hours to achieve high monomer conversion (Figure 3). It was also anticipated that, in cyclohexane solvent,^34,35^ any effects of the attached OBn and OTBS groups on the microstructure, for example, due to coordinating properties, were more likely to be observed.

**Figure 3:**
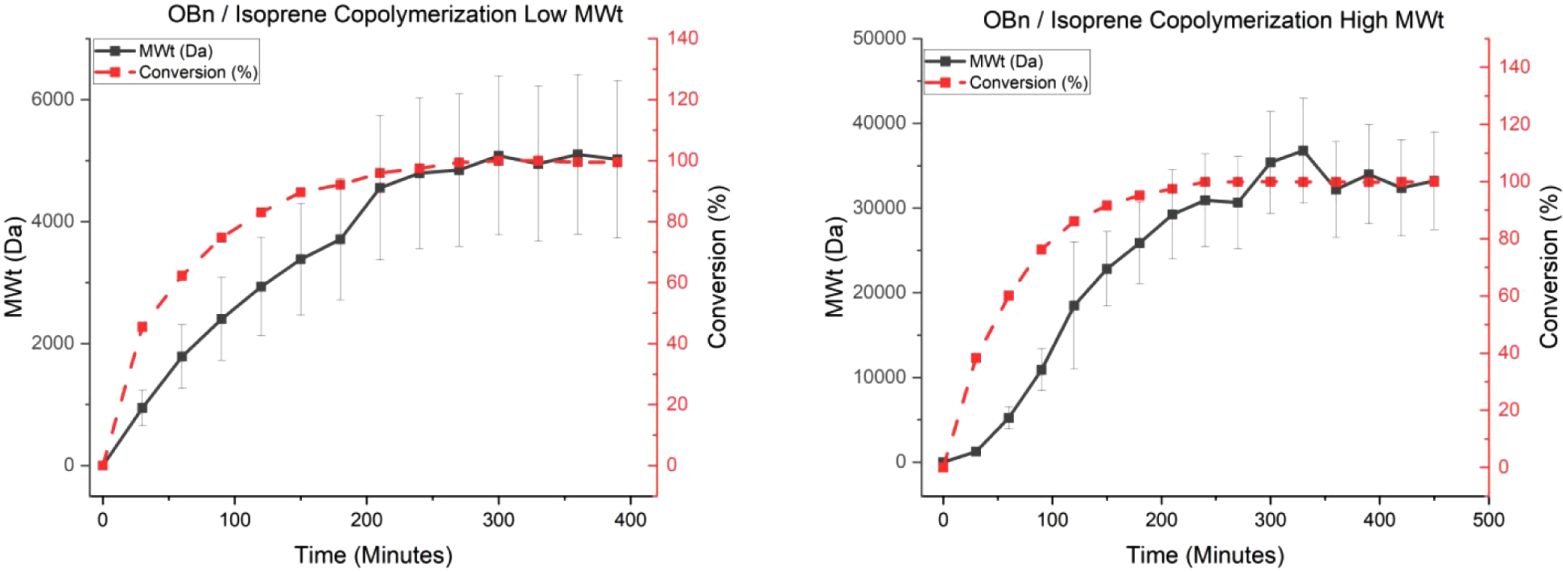
Reaction time vs MWt plot of the copolymerization of **2** with isoprene. MWt values were determined using published MaDDOSY calculations by Tooley et al.; associated errors for the final values are also included.^77,78^ Examples are shown of both low target molecular weight copolymer (4400 Da, 7.9 mol % **2** incorporated) and high target molecular weight copolymer (30 000 Da, 6.4 mol % **2** incorporated). The microstructure contents in each example were: −OBn low MWt: 0% 1,2-, 70.6% 1,4-, 29.4% 3,4-polymer, OBn high MWt: 0% 1,2-, 68.9% 1,4-, 31.1% 3,4-polymer.

Kinetic studies were undertaken on the copolymerizations of **2** and **3** with isoprene. Diffusion coefficients associated with the polymer generated were taken over time, converted to their corresponding molecular weights, and plotted over time (Figures 3 and 4, Supporting Information). Copolymerization of **2** and isoprene (at a ratio of sBuLi:**2**:isoprene of approximately 1:10:60 for the low MWt polymer, with a target MWt of ca. 6000 Da, and 1:20:120 for the high MWt polymer, with a target MWt of ca. 12 000 Da) went to completion in both cases and yielded products containing ca. 7.9 mol % and 6.4 mol % functional polymer, respectively, with MWts of ca. 4500 Da and 30 000 Da, respectively. It was thus possible to selectively prepare lower- and higher-MWt polymers as characterized by in situ DOSY NMR.

**Figure 4:**
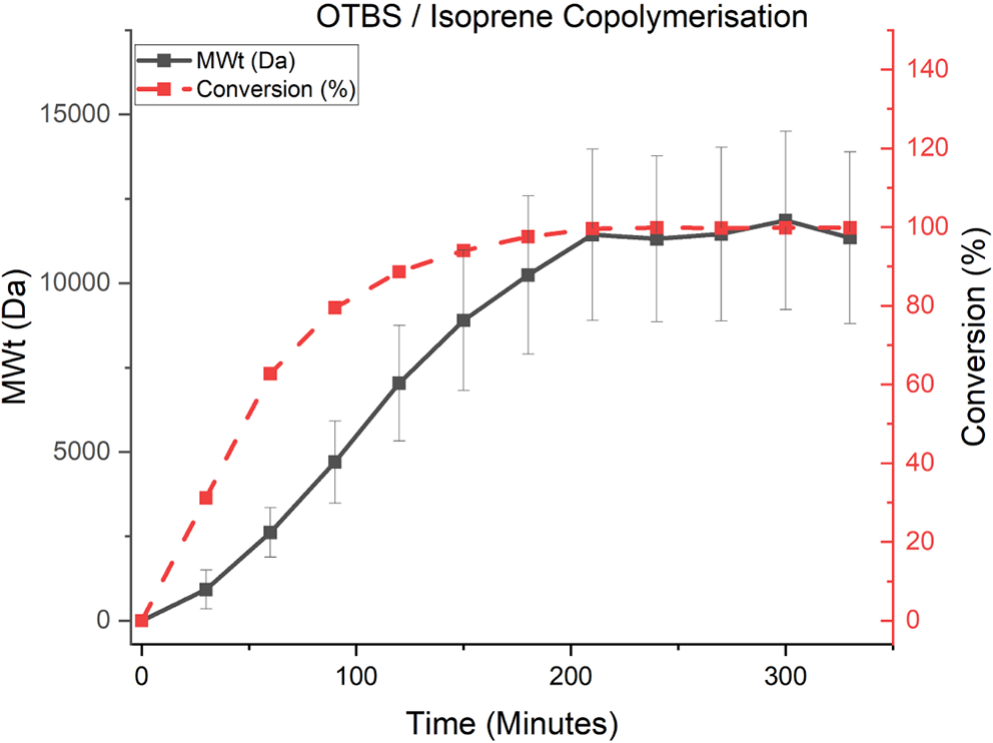
Reaction time vs MWt plot of the copolymerization of **3** with isoprene. MWt values were determined using published MaDDOSY calculations by Tooley et al. Associated errors for the final MWt value are also included.^63,77^ 3.4 mol % of **3** was incorporated into the product, and the product microstructure was 0% 1,2-, 92.4% 1,40-, and 7.6% 3,4-polymer.

Significantly, the proportion of 3,4-microstructure in each copolymer of **2** with isoprene, in cyclohexane, was ca. 29.4% and 31.1%, respectively, the balance being 1,4-, i.e., much higher than the ca. 6.5% 3,4-product microstructure formed for polyisoprene homopolymer in cyclohexane (Table 1). Thus, −OBn has an effect similar to that of THF, as has been evidenced in similar reactions of ether- and acetal-containing monomers **1a** and **1b**.^34,35^ Plotting the monomer conversion over time (see the Supporting Information) demonstrates that the points at which monomer conversion is complete match those at which the MWt plateaus in each case. This indicates that polymer growth continues until the monomer is fully consumed. The conversion slightly outpaces the increase in MWt in the early stages of the polymerization, reflecting previous observations with DOSY NMR,^78^ and this may be the result of very low-MWt oligomers diffusing at similar speeds to the monomers. Due to the lack of distinct peaks in each monomer and the functionalized polymer to track in the NMR, however, it was not possible to establish the relative rates of the polymerization of the isoprene vs the functionalized polymer, and this remains an objective for further study.

Copolymerization of **3** and isoprene was carried out with the same procedure as for **2** (Figure 4) with an sBuLi:**3**:isoprene ratio of 1:4.4:60 and a target polymer MWt of ca. 5000. Reflecting the earlier results, the conversion vs time plot shows that polymer growth plateaus only when monomer consumption is complete. In this case, the polymer product had an MWt of ca. 11 000 Da, contained ca. 3.4 mol % of functionalized monomer, and the final microstructure was ca. 7.6% 3,4- (the balance being 1,2-polyisoprene), which is not much more than the 6.5% previously obtained for isoprene polymerization in cyclohexane alone (Table 1), indicating a lesser coordination of the Li-terminated chain by the more hindered OTBS in **3**, compared to OBn in **2**. Although there is less OTBS functionality, as a lower feed ratio was used, in this polymer than in the −OBn examples, the % of 3,4-microstructure did not increase by a corresponding amount, suggesting the weaker activating effect of the OTBS. Hence, the choice of the protecting group in this copolymerization has a significant influence on the microstructure of the resulting copolymer.

In the polymerizations of both **2** and **3** with isoprene in cyclohexane, the DOSY NMR data for conversion over time also provided evidence for the living nature of the anionic polymerizations; in both cases, plots of ln­([M]_0_/[M]_t_) vs time show a nearly linear relationship, with an intercept at ca. zero, suggesting a constant number of active centers throughout the polymerization in each case (see the Supporting Information). Further investigations into this feature of the polymerization and its application to, for example, the preparation of block copolymers remain the objective of ongoing studies.

In the copolymerization of **2** and isoprene, ^1^H NMR peaks corresponding to the 3,4-microstructure (5.1 ppm) are more prevalent than when using monomer **3** and isoprene. During the anionic polymerization of **2** in cyclohexane, there is a bright yellow color (Supporting Information). This is also seen for dienes polymerized by AP using an alkyllithium initiator in polar solvents such as THF.^79^ The bright yellow color suggests that the −OBn group is sufficiently polar to promote sufficient solvation, which could be through an inter- or intramolecular manner (Figure 5). This was not seen for **3**, suggesting that the OTBS pendant chain has a lesser solvating effect (Supporting Information).

**Figure 5:**
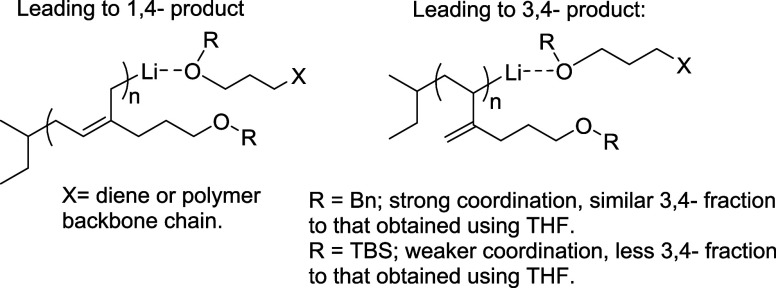
Possible coordination of the O groups of monomers **2** and **3** to the growing polymer chain in cyclohexane solution. The interactions may also be intramolecular.

### Selective Deprotection to Yield Both Saturated and Unsaturated Polymer Chains

Benzyl ether deprotection is usually carried out using hydrogenation, commonly by employing palladium on charcoal (Pd/C) catalysis. *tert*-Butyldimethylsilyl ethers have been removed from polymer moieties using tetra-*N*-butylammonium fluoride (TBAF).^80,81^ Using these deprotection conditions on homopolymers of **2** and **3** would potentially lead to saturated and unsaturated polymer products, respectively, as Pd/C hydrogenation has the potential to also hydrogenate all the double bonds (Figure 6).

**Figure 6:**
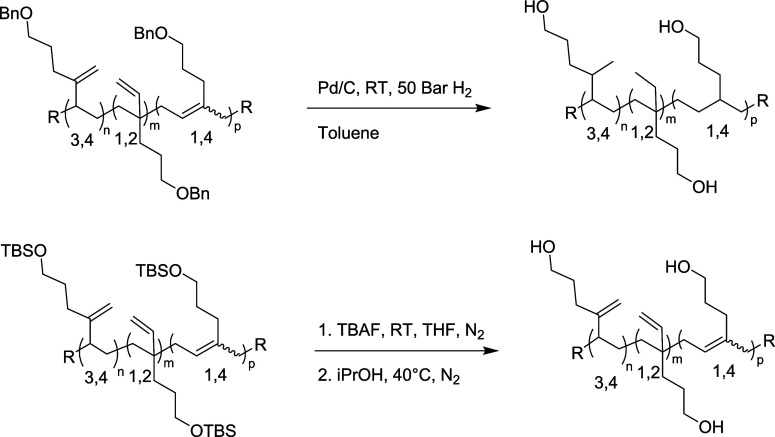
Hydrogenation and deprotection of polymers made from **2** (upper) and **3** (lower).

It is important to consider the potential and desirability of hydrogenation of the unsaturation in the polymer chain as well as end-group deprotection when selecting a deprotection method. Where hydrogenation of the polymer backbone is required, it would be preferable to use the Pd/C hydrogenation method to remove all alkenes in situ. For retention of the unsaturation, TBAF deprotection is a plausible method. Both methods were used in the current work.

First, Copol 3 was deprotected in an autoclave by hydrogenation using Pd/C as a catalyst. The product showed a very distinctive change in structure by ^1^H NMR after hydrogenation and full conversion to a new product. ^1^H NMR peaks between 4 and 6 ppm completely disappeared, indicating full hydrogenation of the double bonds within the polymer; peaks at 4.4 ppm and 7.0–7.5 ppm corresponding to O*C* **H**_**2**_Ph and O*C*H_2_ **Ph**, respectively, were also absent from the product (Figure 7). This demonstrated complete benzyl ether deprotection as well as alkene hydrogenation.

**Figure 7:**
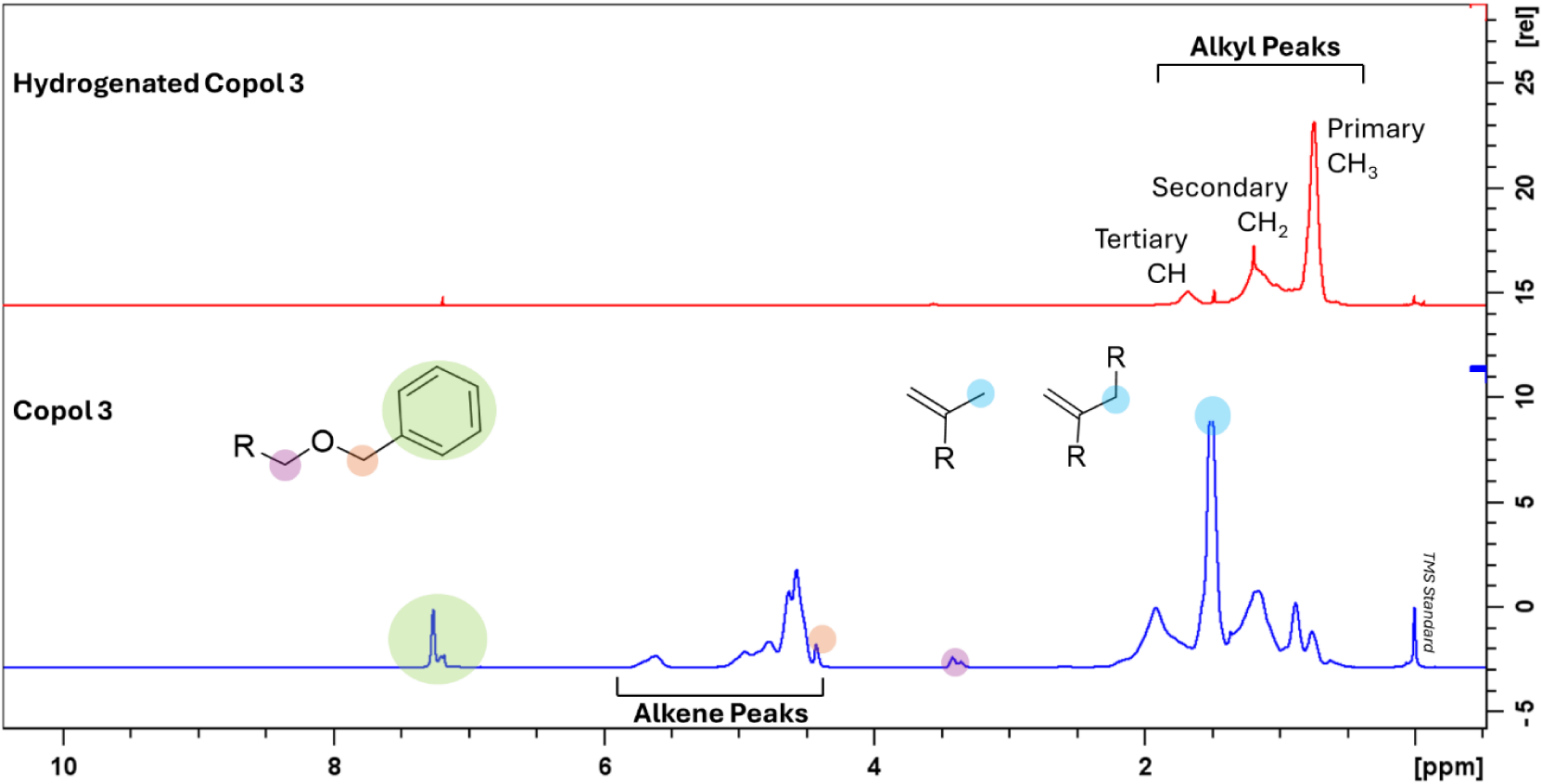
^1^H NMR stack plot of Copol 3 (upper) and the hydrogenated product (lower), showing the disappearance of all benzyl and alkene protons from the spectrum.

Although the change may be very subtle, hydrogenation of alkenes would lead to an increase in mass for each repeat unit of 2 Da, and the deprotection of benzyl ether functional groups would lead to a decrease in mass of 90 Da. For Copol 3, a polymer chain containing 4.1% monomer **2** and 95.9% isoprene, a decrease in MWt is expected and was observed by GPC, showing a significant change in the polymer following the hydrogenation (Figure 8).

**Figure 8:**
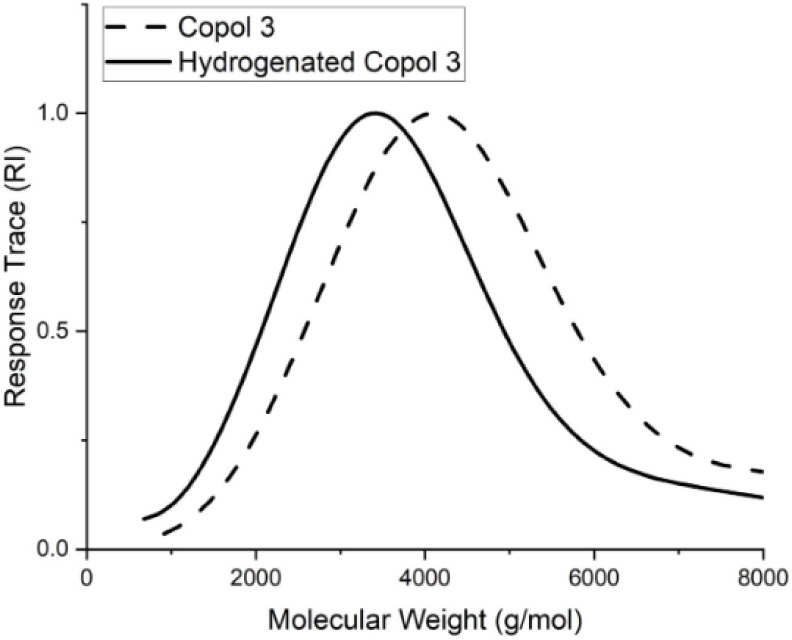
GPC plots of Copol 3 and hydrogenated Copol 3 overlaid to show the change in the polymer MWt following the hydrogenation procedure.

Copol 6 was deprotected using TBAF in THF at 50 °C for 2 h. Full deprotection was confirmed by ^1^H NMR (Figure 9), and the disappearance of the two signals from the TBS group was clearly reflected in the product (the other signal at δ 0.0 being the TMS reference peak). GPC analysis of the polymer product also showed an apparent decrease in mass as the large TBS groups were removed (Figure 10) from the polymer upon treatment with TBAF.

**Figure 9:**
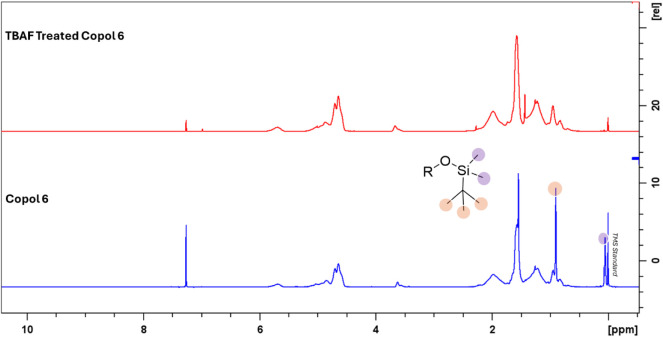
^1^H NMR stack plot of Copol 6 (lower) and deprotected Copol 6 (upper), showing the loss of peaks corresponding to the TBS group (ca. 0.05, 0.90 ppm) in the product.

**Figure 10:**
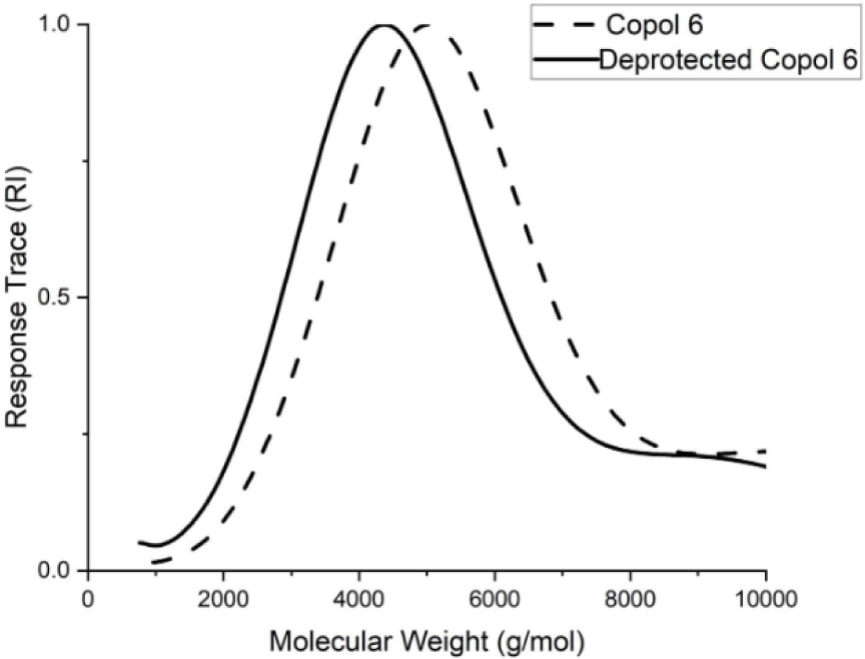
GPC plots of Copol 6 and TBAF-deprotected Copol 6 overlaid to show the change in the polymer during the deprotection procedure.

The hydrogenated/deprotected Copol 3 and Copol 6 were analyzed by DSC and TGA and compared to their unsaturated precursors. Changes in thermal degradation temperatures and glass transition temperatures were slight, although slightly higher than the temperatures for the protected polymers (Table 2).

**Table 2:** Properties of Selected Copolymers (Copol 3 and Copol 6) before and after Hydrogenation and Deprotection Respectively^a^

Polymer	*M*_n_ (g mol–^1^)	*M*_w_ (g mol–^1^)	Đ	*T*_g_/°C	*T*_d_/°C
Copol 3	3,660	4,700	1.28	–18	377
Hydrogenated Copol 3	2,500	3,420	1.37	–14	396
Copol 6	4,900	6,220	1.27	–15	387
Deprotected Copol 6	3,900	5,110	1.31	–9	388

a Number-average molecular weights (*M*_n_), weight-average molecular weights (*M*_w_), and dispersity (Đ) values all obtained from GPC. *T*_g_ was found using DSC, and *T*_d_ values were obtained by TGA.

Hydrogenation of double bonds in a polymer chain increases oxidative and thermal stability.^82^ Conversely, polymers containing unsaturation offer interesting material properties, with examples including cross-linking by traditional and inverse vulcanization,^83–86^ use as drying oils in the coatings industry,^87,88^ and in the production of shape memory materials (SMPs).^88^ TBAF-treated Copol 6 gives an example of how partial OH functionalization can be introduced into a polyene.

## Conclusions

Herein, we have demonstrated the potential of ozonolysis of myrcene and subsequent −OH protection to give new monomers, which subsequently produce new functional polymers. Homopolymers were first produced on a small scale, and copolymers were prepared using protected monomers with isoprene. The introduction of a small percentage of functionalized derivatives can result in changes to the properties and structure of the resulting polymers, such as a change to the microstructure of the polymer and to the *T*_g_ values, depending on the solvent and conditions used for the polymerization. Deprotection of select copolymers was carried out, monitoring changes to the molecular weight, microstructure, ^1^H NMR spectra, and thermal properties of the resulting polymers. The complementary protecting groups provided a means for the differential synthesis of hydroxylated homopolymers or copolymers with isoprene containing either a saturated or unsaturated polymer backbone. Thus, we have shown that very well-developed ozonolysis chemistry can be used with myrcene, a biorenewable chemical feedstock, to give novel monomers containing polar groups. Following standard organic chemistry protecting group chemistry, these monomers can be successfully polymerized by anionic polymerization to give copolymers with different microstructures. The resulting polymers are easily hydrogenated to give fully saturated polymers containing −OH functionality and thus increased polarity. Removal of the unsaturation improves stability, leading to a new family of functional polymers using biorenewable feedstock. As anionic polymerization has been used, it is expected that polymers can be chain extended to give block copolymers and end-functionalized copolymers, as would be expected from a living anionic polymerization, and this will be the focus of future work. This demonstrates the potential of ozonolysis for the production of functional monomers from myrcene, and further detailed studies are ongoing to define the scope of this methodology.

## Supplementary Material



## Notes

Experimental procedures, characterization data for small molecules and polymers, including spectra, and tables of data used in kinetic studies (PDF)
