# Supplementary material for: Anionic Polymerization of O‑Benzyl and O-tert-Butyldimethylsilyl Dienes Derived from Myrcene to Yield Functional Polyenes
Source: ACS Omega. 2026 Jun 9;11(24):35869–79. doi: 10.1021/acsomega.6c02544 (PMC13295024; doi:10.1021/acsomega.6c02544)
Supplement: Supplementary file 1 [file ao6c02544_si_001.pdf]

Supporting Information.

Anionic Polymerization of O-Benzyl and O-*tert*-Butyldimethylsilyl Dienes Derived from Myrcene to Yield Functional Polymers

Joe Stanley[I], Rowan Radmall,[1] William Pointer,[1] Gary Walker[II], David M. Haddleton[I], Martin Wills[I]*

[I]Department of Chemistry, University of Warwick, Coventry CV4 7AL, U.K.

[II]The Lubrizol Corporation, Hazelwood, Derby DE56 4AN, U.K.

CONTENTS

1 Characterization Instrument details 2
2 Synthetic Procedures 4
3 Monomer Synthesis - Spectra 12
4 Homopolymer Data 27
6. Polyene Standards Data 35
7 Copolymer Data 51
5 Deprotected Copolymer Data 69
6 TBAF Treated Copol 6 72
7 DOSY Tracking experiments data 75
8 Table of Quantitative Microstructure Values for polymers from Table 1 118
9 Diffusion constants obtained from DOSY experiments and their corresponding MWt and error values. 119
10 Conversion / time plots for DOSY spectra 121
11 Plots of time vs ln([M]o/[M]t) 160
12 References: 164

1 CHARACTERIZATION INSTRUMENT DETAILS

Nuclear Magnetic Resonance (^{1}H NMR) spectra were recorded on a Bruker DPX-400 spectrometer with samples solubilized in deuterated solvents obtained from Merck.

Gel Permeation Chromatography — Size Exclusion Chromatography (GPC-SEC) was employed to determine the number-average and weight-average molecular weights (M_n and M_w) and dispersity of the polymers using an Agilent 390-LC MDS system. Tetrahydrofuran (THF) containing 2 % TEA (triethylamine) and 0.01 % BHT (butylated hydroxytoluene) was used as the mobile phase at a flow rate of 1 mL/min and a column temperature of 30 °C. The equipment was fitted with a differential refractive index (DRI), viscometry (VS), dual Chapter 2 74 angle light scatter (LS) and dual wavelength UV detectors, 2 PLgel Mixed C columns (300 x 7.5 mm) and a PLgel 5 μm guard column for separation and an autosampler for sample injection. Narrow molecular weight poly(methyl methacrylate) and polystyrene standards (Agilent EasyVials) were used for calibration over the range 550 g mol^{-1} to 1,568,000 g mol^{-1}. Analyte samples were filtered through a GVHP membrane with 0.22 μm pore size prior to injection and all samples were passed through a 0.2 μm PTFE filter membrane before analysis.

Water Determination (WD) measurements were carried out with Mettler Toledo™ C20 and V20 Series Karl FischerTM titrator to quantify water content in liquids. A 0.5 mL aliquot of sample was drawn into a 1 mL syringe and injected into the KF titrator. Results were automatically reported after a 3 min measurement period.

Thermogravimetric Analysis (TGA) was performed on a TA Instruments TGA fitted with an autosampler. Nitrogen gas was used, and samples were heated at 10 °C/min in alumina pans from 25 °C to 600 °C.

Differential Scanning Calorimetry (DSC) analyses were conducted on a TA Instruments DSC with an autosampler. Samples were sealed in alumina crucibles with pierced lids and subjected to heating and cooling cycles from -100 to 250 °C under a flow of N_2 at a heating rate of 10 °C/min. Data reported correspond to the second heating cycle in all cases.

Matrix-Assisted Laser Desorption Ionization Time-of-Flight Mass Spectrometry (MALDI-ToF MS) measurements were acquired in reflectron positive mode using a 21kV acceleration voltage and a 25 kV reflectron voltage. Laser power was kept as low as possible, and spectra were obtained by accumulating and averaging 10000 shots. Polymer samples were prepared in $CHCl_3$ at 10 mg/mL with 1 mg/mL AgTFA added as a cationising agent (Ag^+). These solutions were mixed 1:1 with a 40 mg/mL solution of trans-2-[3-(4-tert-butylphenyl)-2-methyl-2 propenylidene] malononitrile (DCTB) in $CHCl_3$. Aliquots of 0.5 μL were spotted onto an MTP 384 ground steel target plate and analysed using a Bruker AutoFlex Speed II ToF analyser equipped with a 337 nm nitrogen laser.

DOSY NMR Tracking experiments: Magritek Spinsolve 80 Carbon benchtop NMR spectrometer fitted with a z-axis gradient coil capable of generating a maximum field gradient of 500 mT/m was used. 1D ^{1}H NMR spectra were

interpreted using Magritek Spinsolve version 2.3.6, and DOSY spectra were also analyzed using Magritek Spinsolve version 2.3.6.

IR (Infrared) Spectra were recorded using a Bruker Alpha FTIR Spectrometer, and spectra were analyzed using OPUS version 7.5.

Electrospray Ionization – Mass Spectrometry (ESI-MS): A Bruker Compact Mass spectrometer was used to obtain high resolution mass spectra (HRMS) for molecules **2** and **3**. Experimental details can be found in the analysis reports in the individual data sets below.

2 SYNTHETIC PROCEDURES

Monomer Syntheses

4-Methylenehex-5-en-1-ol: This compound is known and fully characterized.[1] To a round bottomed flask, isopropanol (50 mL), distilled water (10 mL), myrcene (>95% from Merck Life Science, 5.0 g, 37 mmol, 1 eq.) and a magnetic stirrer were added. The ozone generator, supplied with a flow of compressed air at 430 cm^3min^{-1}, was switched on and its exhaust was placed into the reaction mixture. The reaction was stirred for 4 hours during which it was cooled using an ice bath; a persistent white fume was observed for the whole duration. The ozone generator was then switched off and its exhaust removed from the reaction mixture. After 5 minutes of stirring at 0 °C, $NaBH_4$ (2.78 g, 73.5 mmol, 2 eq.) was added in portions. The reaction mixture turned yellow immediately and then clear. After 15 minutes of stirring, distilled H_2O (20 mL) was added and EtO_2 (3 x 50 mL) was used to extract the reaction mixture. The combined organic fractions were dried using $MgSO_4$ and concentrated under vacuum to yield a crude product (2.96 g, 26.5 mmol, 72%). ^{1}H NMR (400 MHz, $CDCl_3$) δ: 6.38 (dd, J = 17.5, 11.0 Hz, 1H, C***H***CH_2), 4.98-5.29 (4H, m, C***H***$_2$CCHC***H***$_2$), 3.69 (2H, t, J = 6.5 Hz, C***H***$_2$OH), 2.31 (2H, t, J = 7.5 Hz, CC***H***$_2$$CH_2$), 1.82–1.73 (2H, m, CH_2C***H***$_2$).

(((4-Methylenehex-5-en-1-yl)oxy)methyl)benzene (2): This compound is known and fully characterized.[2] To a vacuum dried Schlenk tube, 4-methylenehex-5-en-1-ol (2.0 g, 18 mmol, 1 eq.), THF (20 mL) and NaH (0.86 g, 36 mmol, 2 eq.) were added. The reaction mixture was stirred under nitrogen atmosphere for 30 minutes, a yellow to brown colour change was observed. BnBr (3.66 g, 21 mmol, 1.2 eq.) was added and the reaction mixture was stirred at room temperature overnight. $NH_4Cl_{\text{(sat. aq.)}}$ (20 mL) was added, and the aqueous layers were extracted using Et_2O (3 x 20 mL). The combined organic extracts were washed with brine (10 mL) and dried with $MgSO_4$ to yield a crude product. The crude product was purified using column chromatography on silica gel (8% EtOAc: 92% Hexane) to give pure product as a yellow oil (3.24 g, 16 mmol, 89% yield). ^{1}H NMR (400 MHz, $CDCl_3$) δ: 7.38 - 7.21 (5H, m, Ar***H***), 6.37 (1H, dd, J = 17.5, 10.5 Hz, C***H***CH_2), 5.28 – 4.97 (4H, m, C***H***$_2$CHCC***H***$_2$), 4.50 (2H, s, OC***H***$_2$Ar), 3.50 (2H, t, J = 6.5 Hz, CH_2C***H***$_2$O), 2.31 (2H, t, J = 6.5 Hz CC***H***$_2$$CH_2$), 1.86 – 1.77 (2H, m, CH_2C***H***$_2$$CH_2$). ^{13}C NMR δ_C (100 MHz, $CDCl_3$): 144.83, 137.77, 137.60, 127.32, 126.58, 126.47, 114.82, 112.28, 71.86, 68.89, 27.17, 26.79. ν_{max} 3030, 2926, 2855, 1595, 1495, 1454, 1363, 1308, 1259, 1204, 1102, 1028, 991, 895, 805, 733, 696, 611, 457. HRMS (ESI-TOF) m/z: $[M + H]^+$ Calcd for $C_{14}H_{19}O$ 203.1430; Found 203.1430 (error 0 ppm).

tert*-Butyldimethyl((4-methylenehex-5-en-1-yl)oxy)silane (3)**: This compound is known and fully characterized.[3] To a vacuum dried Schlenk tube, 4-methylenehex-5-en-1-ol (2 g, 18 mmol, 1 eq.), CH_2Cl_2 (20 mL) and imidazole (1.63 g, 24 mmol, 1.5 eq.) were added. The mixture was stirred at room temperature under nitrogen atmosphere for 30 minutes. TBSCl (3.1 g, 21 mmol, 1.3 eq.) was then added and the reaction mixture was left to stir overnight. Distilled water (30 mL) was then added, and the aqueous layer was extracted using DCM (3 x 20 mL). The combined organic extracts were washed with $NaHCO_{3\,\text{(sat. aq.)}}$ (15 mL), dried using $MgSO_4$. The crude product was purified using column chromatography on silica gel (2% EtOAc: 98% Hexane) to give pure product as a yellow oil (3.16 g, 14 mmol, 78% yield). ^{1}H NMR (400 MHz, $CDCl_3$) δ: 6.62 (1H, dd, J = 17.5, 10.5 Hz, CH***CH_2), 5.23 – 4.93 (4H, m, C***H***$_2$CHCC***H***$_2$), 3.59 (2H, t, J = 6.5 Hz, C***H***$_2$O), 2.22 (2H, t, J = 8.0 Hz, CC***H***$_2$$CH_2$), 1.70-1.61 (2H, m, CH_2C***H***$_2$$CH_2$). ^{13}C

NMR δ_C (100 MHz, $CDCl_3$): 146.13, 138.87, 115.70, 113.26, 62.78, 31.32, 27.57, 25.97, 18.3, -5.28. ν_{max} 2953, 2929, 2888, 2857, 1595, 1471, 1462, 1253, 1101, 1006, 990, 893, 812, 773. HRMS (ESI-TOF) m/z: $[M + Na]^+$ Calcd. for $C_{13}H_{26}NaOSi$ 249.1645; Found 249.1642 (error 1.3 ppm).

Homopolymer Synthesis

Monomers **2** and **3** were made up into 200 mg mL^{-1} solutions in cyclohexane prior to use. These solutions were dried using 4 A molecular sieves, which were activated through heating under vacuum. An example homo-polymerization of **2** with a targeted DP = 10 is as follows.

A solution of **2** in cyclohexane (1 mL of soln., 200 mg of **2**, 0.99 mmol of **2,** 10 eq. of **2**) was added to a flame dried round bottomed flask equipped with a magnetic stirrer bead and a Schlenk adapter under N_2 atmosphere. THF (0.5 mL) was added, followed by *sec*-Butyllithium (0.071 mL of 1.4M soln., 0.099 mmol, 1 eq.) and the reaction was stirred for 15 hours. Anhydrous MeOH (2 mL) was then used to quench the reaction, and the mixture was concentrated under vacuum to yield the final polymer product.

Observation when conducting anionic polymerisations using **2** and **3** in purely cyclohexane solvent:

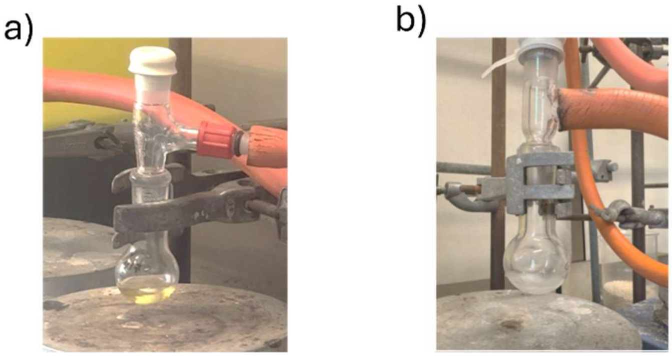

Figure S1: a) A bright yellow polymerization mixture using the OBn derivative 2 as a monomer in cyclohexane. b) A pale polymerization mixture using the OTBS derivative 3 as a monomer in cyclohexane.

Copolymer Synthesis

Monomers **2** and **3** were made up into 200 mg mL^{-1} solutions in cyclohexane prior to use. These solutions were dried using 4 A molecular sieves, which were activated through heating under vacuum. Isoprene was dried in the same manner, but with no cyclohexane solvent. An example synthesis of **Copol 3** is as follows.

A solution of **2** in cyclohexane ((0.32 mL of 200 mg mL-1) 63 mg, 0.31 mmol, 5 eq.) was added to a flame dried round bottomed flask equipped with a magnetic stirrer bead and a Schlenk adapter under N_2 atmosphere. Anhydrous Isoprene (442 mg, 6.5 mmol, 105 eq.) and anhydrous THF (0.5 mL) was added, followed by the addition of *sec*-

butyllithium (0.044 mL (1.4M soln.), 0.062 mmol, 1 eq.). The reaction was stirred for 15 hours. Anhydrous MeOH (2 mL) was then used to quench the reaction. The polymer product was then precipitated in MeOH (30 mL) at 0 °C. The MeOH layer was decanted, and the precipitate was dried under vacuum to yield the final polymer product.

Table S1: Added quantities in each Copol synthesis:

Polymer ID	Amount of 2 or 3	Amount of isoprene	Volume of THF	Amount of *sec*-BuLi
Copol 1 (OBn)	**2** (0.063 mL of 200 mg mL-1) 13 mg, 0.062 mmol, 1 eq.	442 mg, 6.5 mmol, 105 eq.	0.5 mL	0.044 mL (1.4M soln.), 0.062 mmol, 1 eq.)
Copol 2 (OBn)	**2** (0.19 mL of 200 mg mL-1) 38 mg, 0.19 mmol, 3 eq.	442 mg, 6.5 mmol, 105 eq.	0.5 mL	0.044 mL (1.4M soln.), 0.062 mmol, 1 eq.)
Copol 3 (OBn)	**2** (0.32 mL of 200 mg mL-1) 63 mg, 0.31 mmol, 5 eq.	442 mg, 6.5 mmol, 105 eq.	0.5 mL	0.044 mL (1.4M soln.), 0.062 mmol, 1 eq.)
Copol 4 (OTBS)	**3** (0.07 mL of 200 mg mL-1) 14 mg, 0.062 mmol, 1 eq.	442 mg, 6.5 mmol, 105 eq.	0.5 mL	0.044 mL (1.4M soln.), 0.062 mmol, 1 eq.)
Copol 5 (OTBS)	**3** (0.21 mL of 200 mg mL-1) 42 mg, 0.186 mmol, 3 eq.	442 mg, 6.5 mmol, 105 eq.	0.5 mL	0.044 mL (1.4M soln.), 0.062 mmol, 1 eq.)
Copol 6 (OTBS)	**3** (0.35 mL of 200 mg mL-1) 70 mg, 0.31 mmol, 5 eq.	442 mg, 6.5 mmol, 105 eq.	0.5 mL	0.044 mL (1.4M soln.), 0.062 mmol, 1 eq.)

Pd/C catalysed hydrogenation of **Copol 3**

The polymer chain consists of 4.1 mol.% of monomer **2**, and 94.9 mol.% isoprene. Monomer **2** contains two groups which need to be hydrogenated (alkene + benzyl), and isoprene units only have 1 alkene.

The desired Pd/C loading was to be 0.1 eq. of Pd to 1 eq. hydrogenation targets. This was calculated below.

$$\frac{\left((68 \times 95.9) + (202 \times 4.1)\right)}{100} = 73.5\ Da$$

$$= Average\ Mw\ of\ monomer\ subunits\ in\ Copol\ 3$$

$$\frac{\mathbf{100}}{\mathbf{73.5}} = \mathbf{1.36}\ \boldsymbol{mmol}$$

$$= \boldsymbol{mmol\ of\ subunits\ added\ to\ hydrogenation\ reaction}$$

$$\mathbf{1.36} + \left(\frac{\mathbf{1.36}}{\mathbf{100}} \times \mathbf{4.05}\right) = \mathbf{1.42}\ \boldsymbol{mmol\ of\ hydrogenation\ targets\ in}\ \mathbf{100}\ \boldsymbol{mg\ of\ Copol}\ \mathbf{3}$$

$$\frac{\mathbf{1.43}}{\mathbf{10}} = \mathbf{0.142}\ \boldsymbol{mmol\ of\ Pd\ required}$$

Copol 3 (100 mg, 1 eq. wrt double bonds + benzyl groups) was added to a glass insert along with toluene (10 mL). Pd/C (150 mg [10 wt.% Pd loading], 0.142 mmol Pd, 0.1 eq. Pd) was added to the reaction mixture along with a stirrer bar. The insert was placed inside an autoclave which was pressurized to 50 bar of H_2 for 4 days. After which point the autoclave was depressurized, the reaction mixture was passed through a silica plug and concentrated under vacuum to yield 85% hydrogenated polymer.

At this point, the reaction was run again using this polymer product as starting material but switching the solvent to cyclohexane. This allowed for the complete hydrogenation of the remaining alkenes. The reaction mixture was then passed through a silica plug once more to yield 100% hydrogenated polymer gel.

TBAF catalysed Deprotection of **Copol 6**

Calculations of the amount of OTBS in each chain of Copol 6.

$$\frac{\left((\mathbf{68} \times \mathbf{94.3}) + (\mathbf{226}\ \times \mathbf{5.7})\right)}{\mathbf{100}} = \mathbf{77}\ \boldsymbol{Da}$$

$$= \boldsymbol{Average\ Mw\ of\ monomer\ subunits\ in\ Copol}\ \mathbf{6}$$

$$\frac{\mathbf{100}\ \boldsymbol{mg}}{\mathbf{77}\ \boldsymbol{Da}} = \mathbf{1.29}\ \boldsymbol{mmol}$$

$$= \boldsymbol{mmol\ of\ subunits\ added\ to\ hydrogenation\ reaction}$$

$$\mathbf{1.29} \times \mathbf{0.0571} = \mathbf{0.074}\ \boldsymbol{mmol\ of\ OTBS\ in\ each\ polymer\ chain}$$

Copol **6** (100 mg, 0.074 mmol of OTBS groups, 1 eq.) was dissolved in THF (10 mL). To the solution, tetrabutylammonium fluoride (TBAF, 1M soln. in THF) (146 mg, 0.56 mmol, 7.5 eq.) was added. The mixture was heated at 50 °C for 2 hours while stirring. The reaction mixture was concentrated under vacuum, then diluted with DCM (20 mL). The DCM solution was then washed with deionized H_2O (3 x 20 mL) and dried using anhydrous Na_2SO_4. The resultant mixture was concentrated under vacuum to yield deprotected Copol 6 as a yellow gel.

DOSY tracking experiments

OTBS + Isoprene copolymerization example.

A solution of **3** in cyclohexane (0.05 mL of 100 $mgmL^{-1}$ soln., 5 mg, 0.022 mmol, 4.4 eq.) was added to a flame dried NMR tube equipped with a suba seal under N_2 atmosphere. Anhydrous Isoprene (0.03 mL, 20 mg, 0.3 mmol, 60 eq.) and anhydrous cyclohexane (0.4 mL) was added, followed by the addition of *sec*-butyllithium (0.007 mL of 1.4M soln., 0.005 mmol, 1 eq.). The NMR tube containing the reaction mixture was inserted into the benchtop NMR, and a reaction monitoring sequence was performed. The machine was programmed to shim to cyclohexane, perform a ^{1}H-NMR, then perform a DOSY NMR. After this, the machine was programmed to pause for 30 minutes then repeat the analytical procedure again. This process repeated for 6 hours.

Table S2: Quantities of reagents and solvents added to DOSY monitoring polymerizations.

**Experiment**	**Amount of 2 or 3**	**Amount of isoprene**	**Volume of Cyclohexane**	**Amount of *sec*-BuLi**
OBn DOSY Low MWt	**2** (0.1 mL of 100 mg/mL soln.) 10 mg, 0.05 mmol, 10 eq.	20.4 mg, 0.3 mmol, 60 eq.	0.4 mL	0.007 mL of 1.4 M soln., 0.005 mmol, 1 eq.
OTBS DOSY	**3** (0.05 mL of 100 mg/mL soln.) 5 mg, 0.022 mmol, 4.4 eq.	20.4 mg, 0.3 mmol, 60 eq.	0.4 mL	0.007 mL of 1.4 M soln., 0.005 mmol, 1 eq.
OBn DOSY High MWt	**2** (0.2 mL of 100 mg/mL soln.) 20 mg, 0.10 mmol, 20 eq.	40.8 mg, 0.6 mmol, 120 eq.	0.4 mL	0.007 mL of 1.4 M soln., 0.005 mmol, 1 eq.

Table S3: Theoretical vs observed monomer loadings and molecular weights

	**Monomer quantities added to reaction (2 s.f)**			**Theoretical monomer loadings (1 d.p)**			**Found Monomer Loadings (1 d.p.)**		
Experiment	Function-al Mono-mer	Iso-prene	*Sec*-BuLi	Function-al Mono-mer %	Iso-prene %	Theoreti-cal MWt	Function-al Mono-mer %	Iso-prene %	Found Final MWt (By DOSY)
OBn DOSY Low MWt	10	60	1	14.3%	85.7%	6100	7.9%	92.1%	5021 (±1290)
OTBS DOSY	4.4	60	1	6.8%	93.2%	5100	3.4%	96.6%	11350 (±2547)
OBn DOSY High MWt	20	120	1	14.3%	85.7%	12000	6.4%	93.6%	33208 (±5769)

Table S4: Quantified microstructures of each DOSY tracked final polymer spectrum

**Polymer**	**Percentage Composition (1 d.p)**			
	1,2	1,4	3,4	Functional Monomer mol.%
OTBS / Isoprene Copolymerization	0%	92.4%	7.6%	3.4%
OBn / Isoprene High MWt	0%	68.9%	31.1%	6.4%
OBn / Isoprene Low MWt	0%	70.6%	29.4%	7.9%

To obtain polymer molecular weights from diffusion co-efficients, the alkenyl region (4.5 – 5.5 ppm) of each DOSY spectrum was integrated in Spinsolve 2.3.6. The associated diffusion co-efficient was then used to find the polymer MWt according to Tooley *et. al.*[4]

Calculating Microstructure from ^{1}H NMR

Published methods were used to deduce polymer microstructure from ^{1}H NMR.[5-7]

Total ^{1}H integrations from NMR (see example NMR spectrum below, corrected for number of protons contributing to each integral).

$$\%\ 1{,}2\ Microstrucuture\ content\ = \frac{I(1{,}2)}{(\ I(1{,}2)\ + (I(1{,}4)\ - (2\ \times\ I(1{,}2)\ )\ +\ \frac{I(3{,}4)}{2}}$$

)

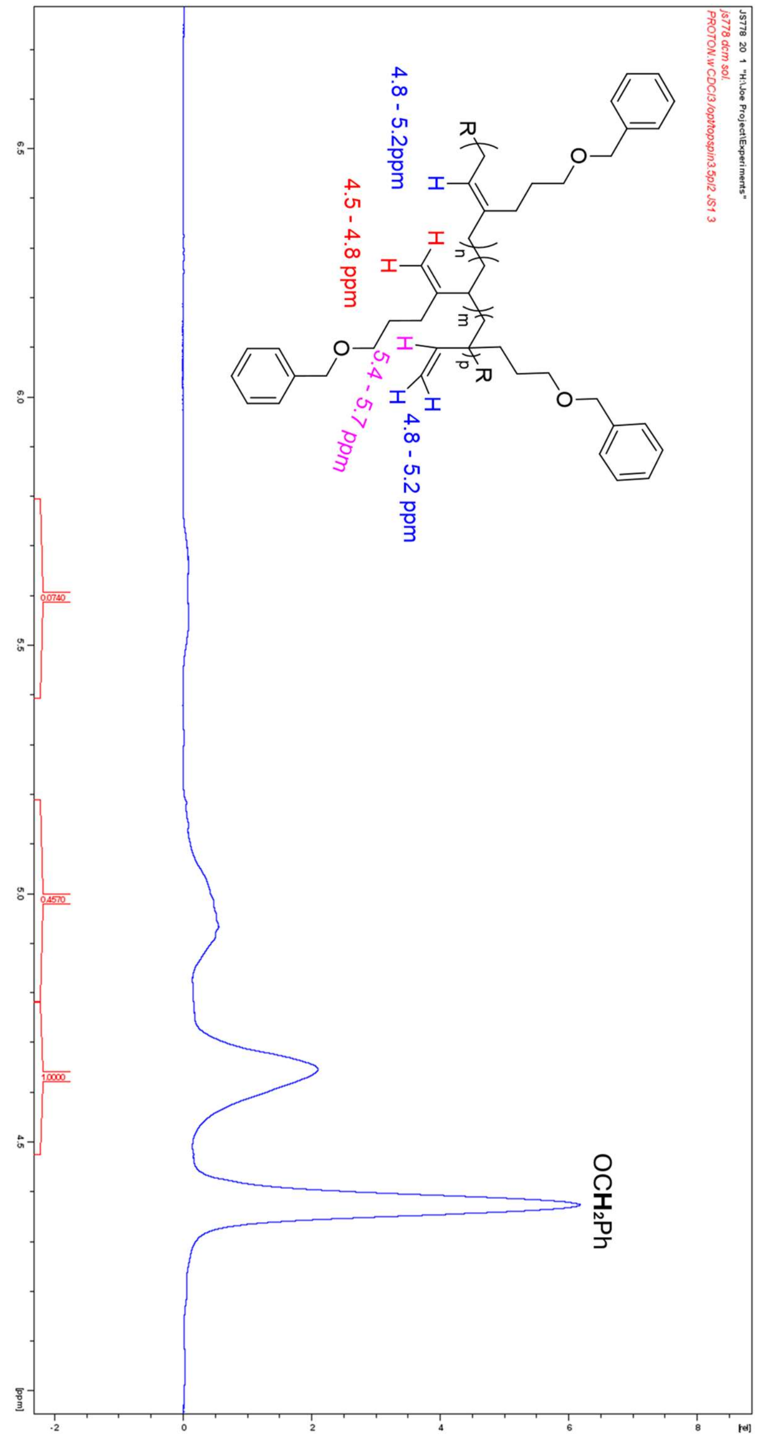

Figure S2: An example of an integrated spectrum (Homo – Bn)

3 Monomer Synthesis - Spectra

4-Methylenehex-5-en-1-ol:

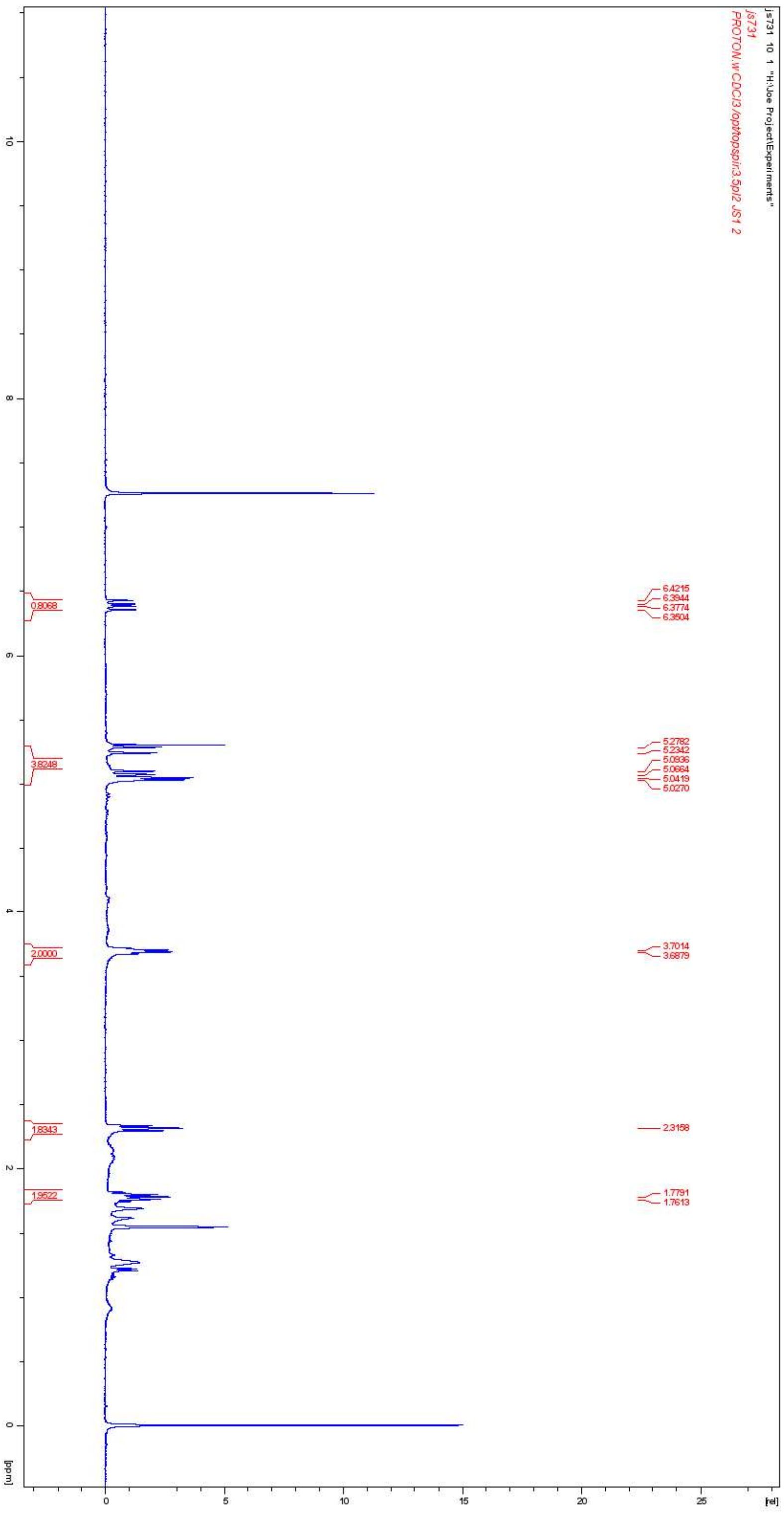

Figure S3: 4-Methylenehex-5-en-1-ol 1H NMR (400 MHz, $CDCl_3$):

(((4-Methylenehex-5-en-1-yl)oxy)methyl)benzene (2):

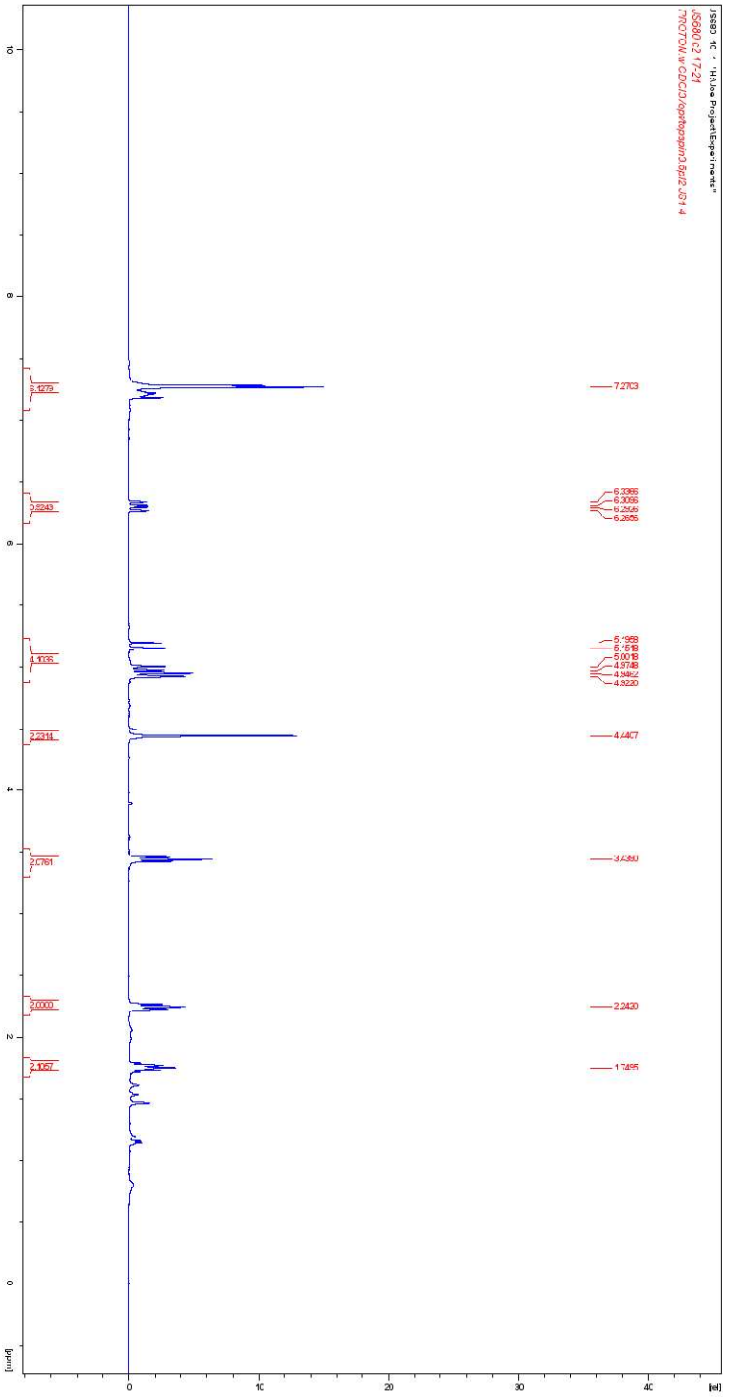

*Figure S4: (((4-Methylenehex-5-en-1-yl)oxy)methyl)benzene (**2**) ^1H NMR (400 MHz $CDCl_3$):*

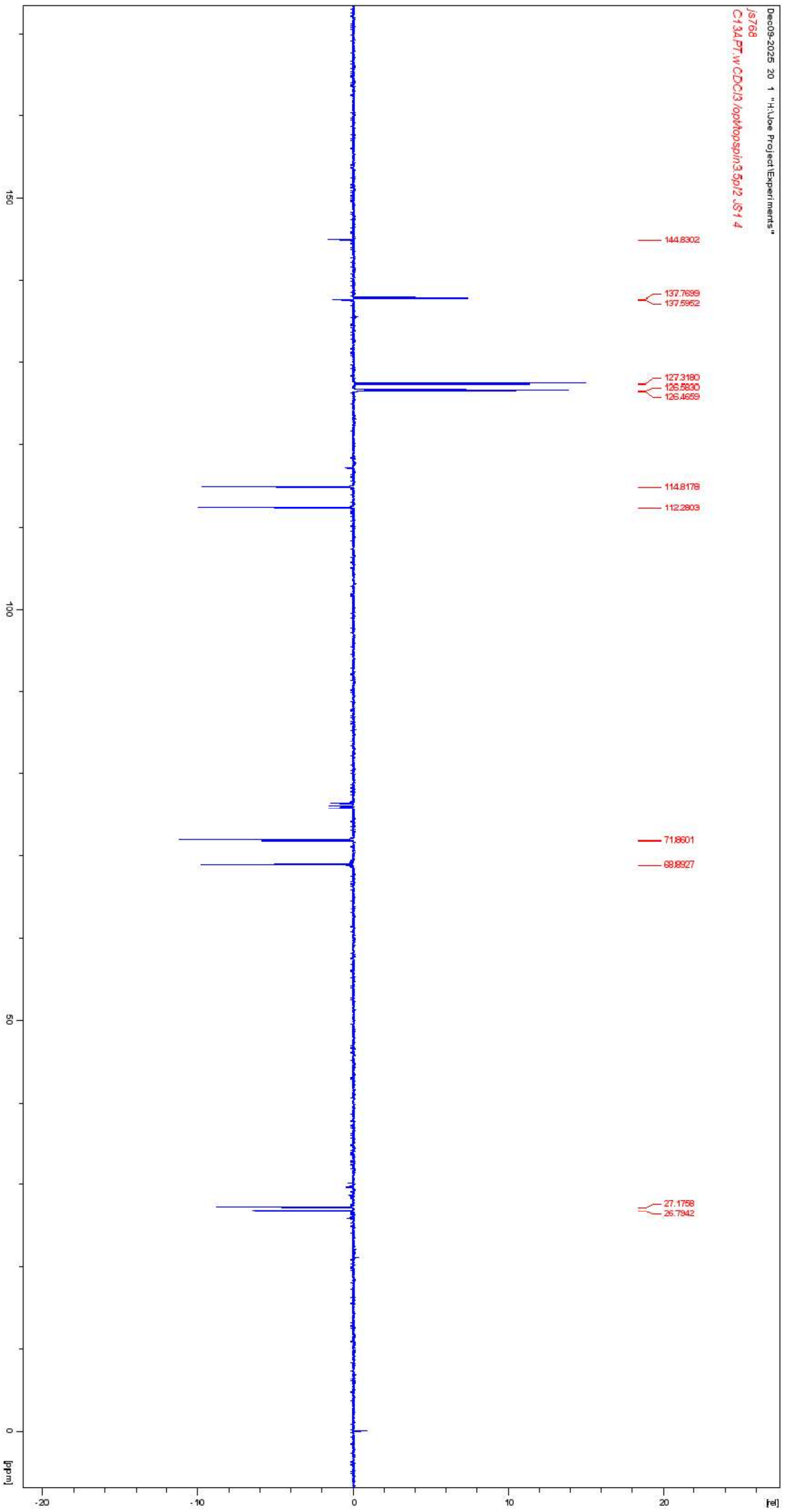

*Figure S5: (((4-Methylenehex-5-en-1-yl)oxy)methyl)benzene (**2**) ^{13}C NMR (400 MHz, $CDCl_3$):*

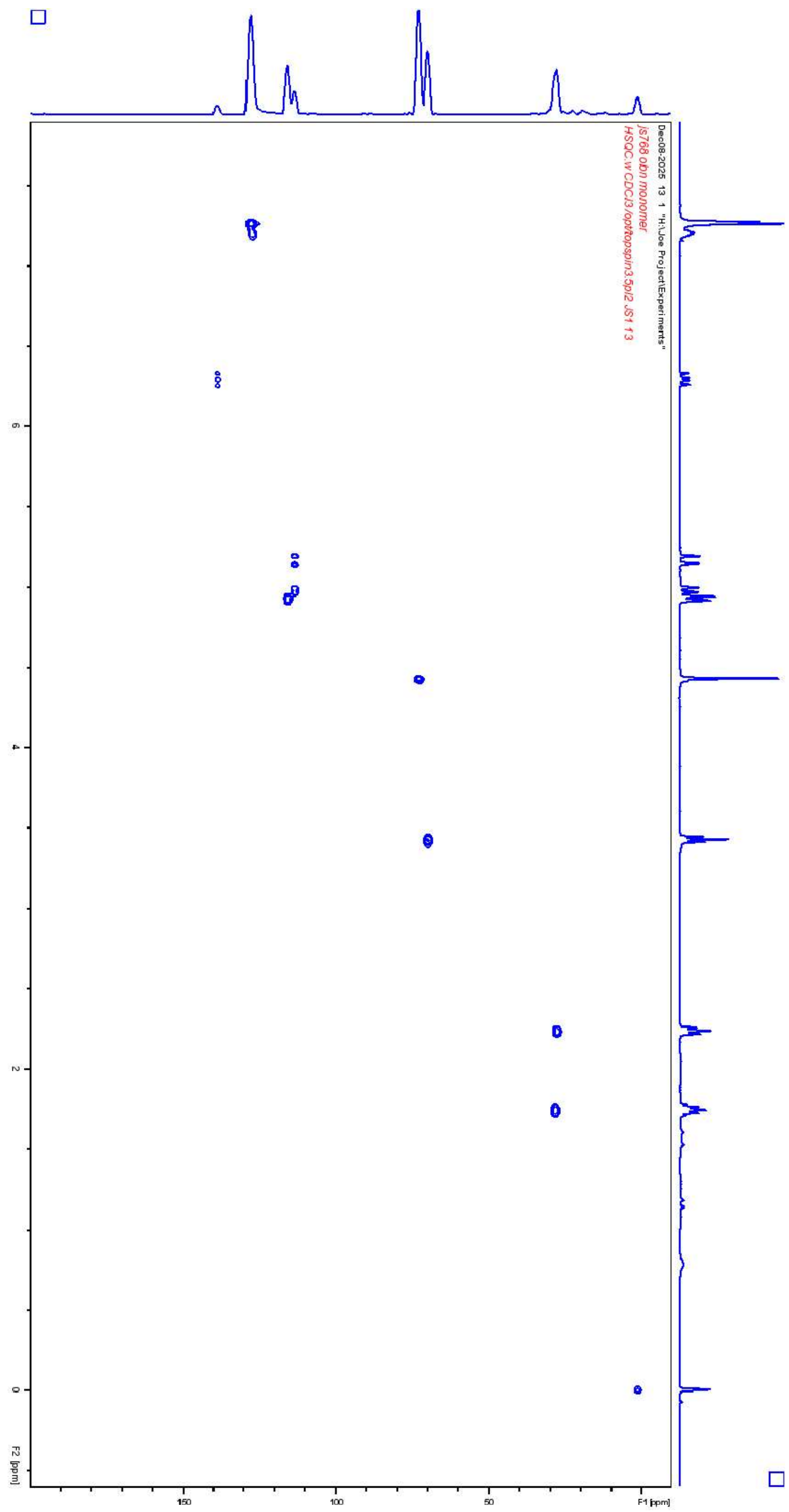

*Figure S6: (((4-Methylenehex-5-en-1-yl)oxy)methyl)benzene (**2**) HSQC NMR (400 MHz, $CDCl_3$)*

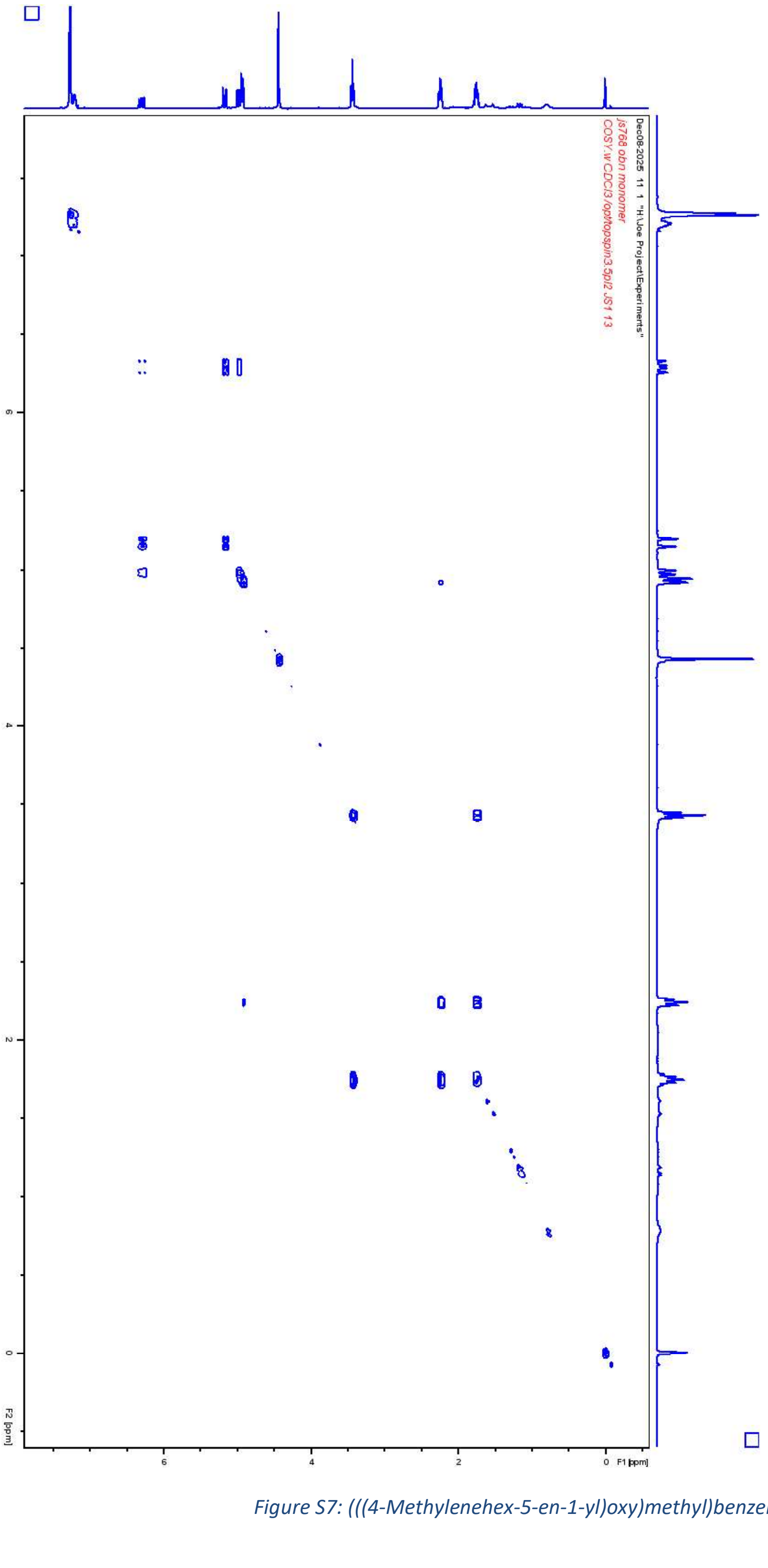

Figure S7: (((4-Methylenehex-5-en-1-yl)oxy)methyl)benzene (2) COSY NMR (400 MHz, $CDCl_3$)

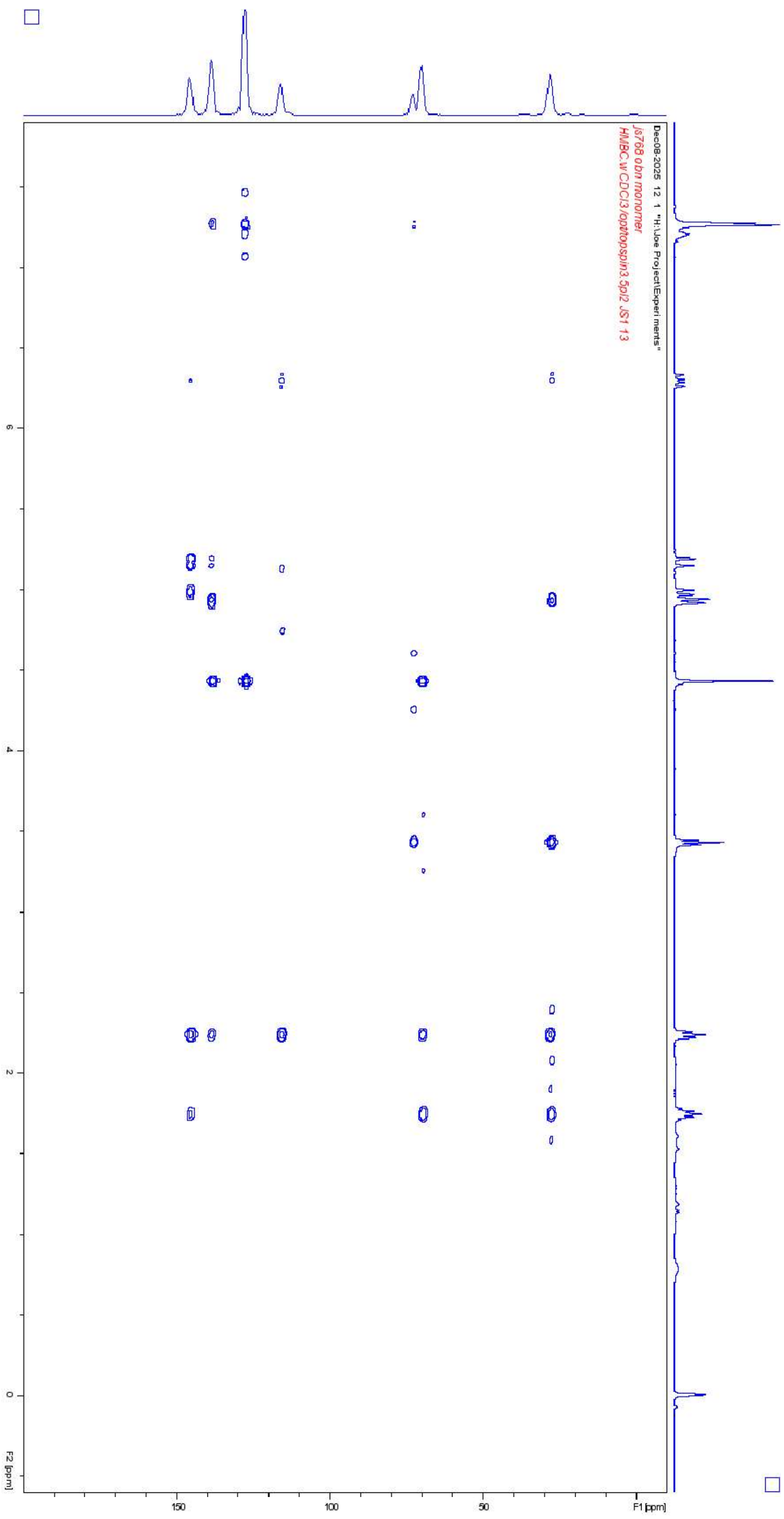

*Figure S8: (((4-Methylenehex-5-en-1-yl)oxy)methyl)benzene (**2**) HMBC NMR (400 MHz, $CDCl_3$)*

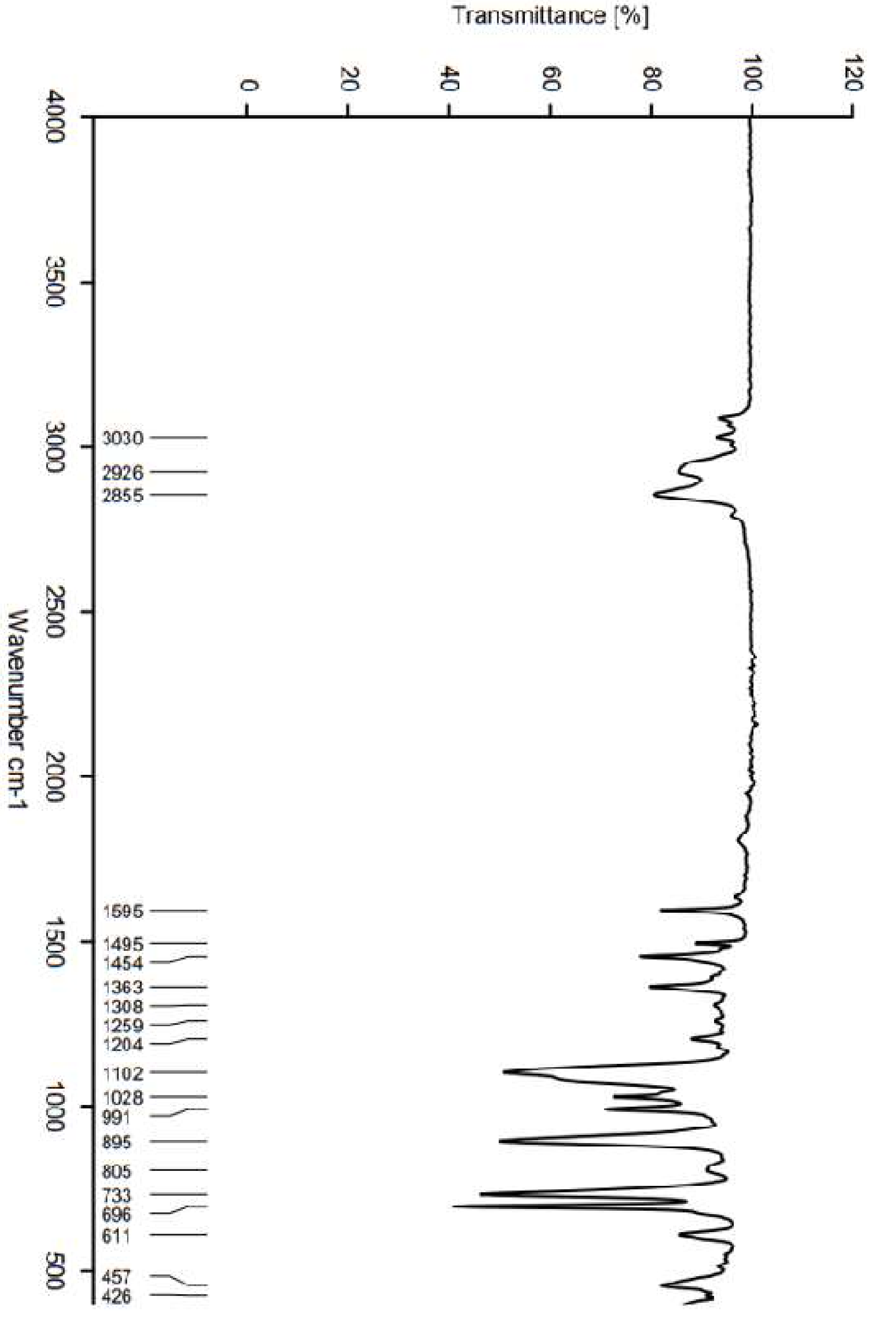

*Figure S9: (((4-Methylenehex-5-en-1-yl)oxy)methyl)benzene (**2**) IR Spectrum*

Mass Spectrum SmartFormula Report

Analysis Info

Analysis Name	D:\Data\SongDec2025\JS OBn.d	Acquisition Date	12/12/2025 1:53:28 PM
Method	lowpositive.m	Operator	Demo User
Sample Name	6	Instrument	compact 8255754.20162
Comment			

Acquisition Parameter

Source Type	ESI	Ion Polarity	Positive	Set Nebulizer	0.3 Bar
Focus	Active	Set Capillary	4200 V	Set Dry Heater	200 °C
Scan Begin	50 m/z	Set End Plate Offset	-500 V	Set Dry Gas	4.0 l/min
Scan End	3000 m/z	Set Charging Voltage	2000 V	Set Divert Valve	Source
		Set Corona	0 nA	Set APCI Heater	0 °C

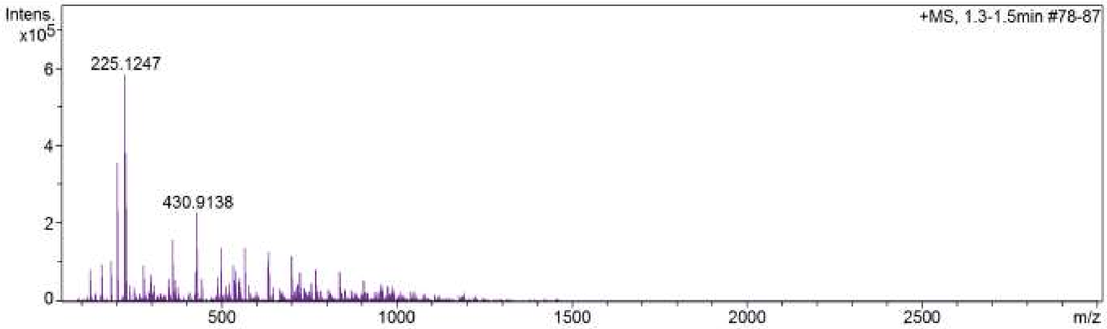

Meas. m/z	#	Ion Formula	m/z	err [ppm]	mSigma	# mSigma	Score	rdb	e⁻ Conf	N-Rule
203.1430	1	C14H19O	203.1430	0.0	6.0	1	100.00	6.0	even	ok

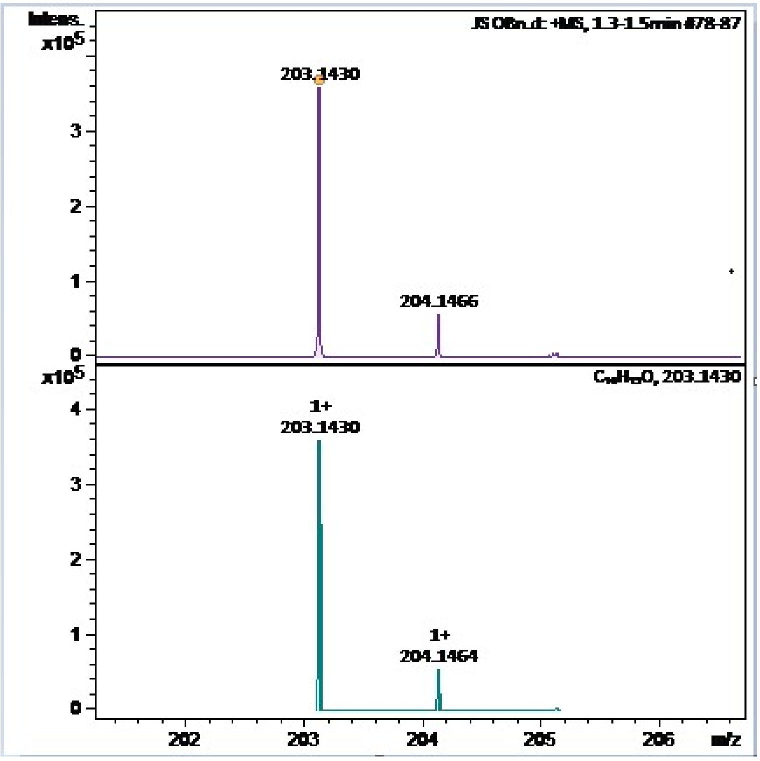

*Figure S10: (((4-Methylenehex-5-en-1-yl)oxy)methyl)benzene (**2**) HRMS-ESI Full analysis report (top) and observed peaks (bottom).*

tert-Butyldimethyl((4-methylenehex-5-en-1-yl)oxy)silane (3):

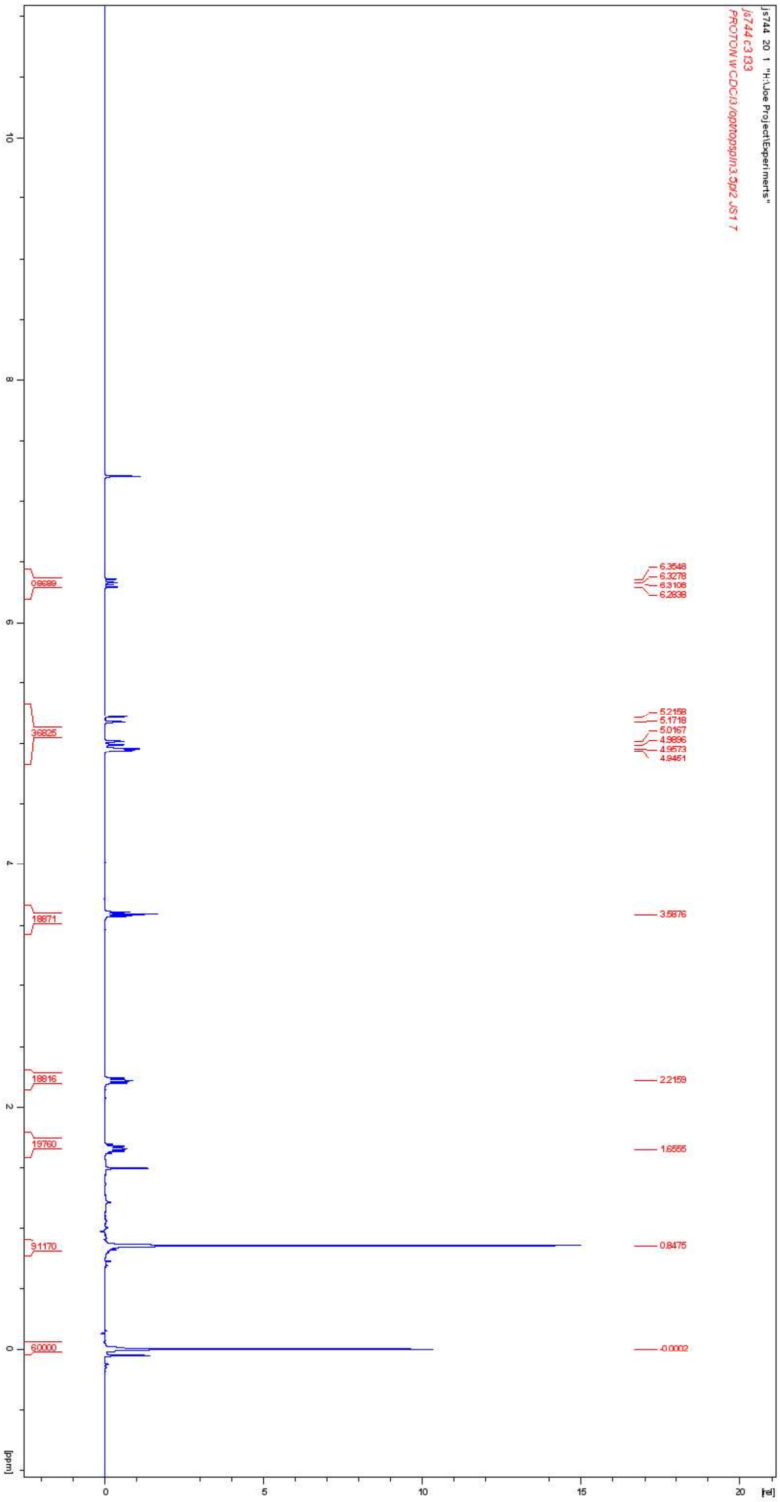

*Figure S11: **tert-Butyldimethyl((4-methylenehex-5-en-1-yl)oxy)silane (3)** ^{1}H NMR (400 MHz, $CDCl_3$):*

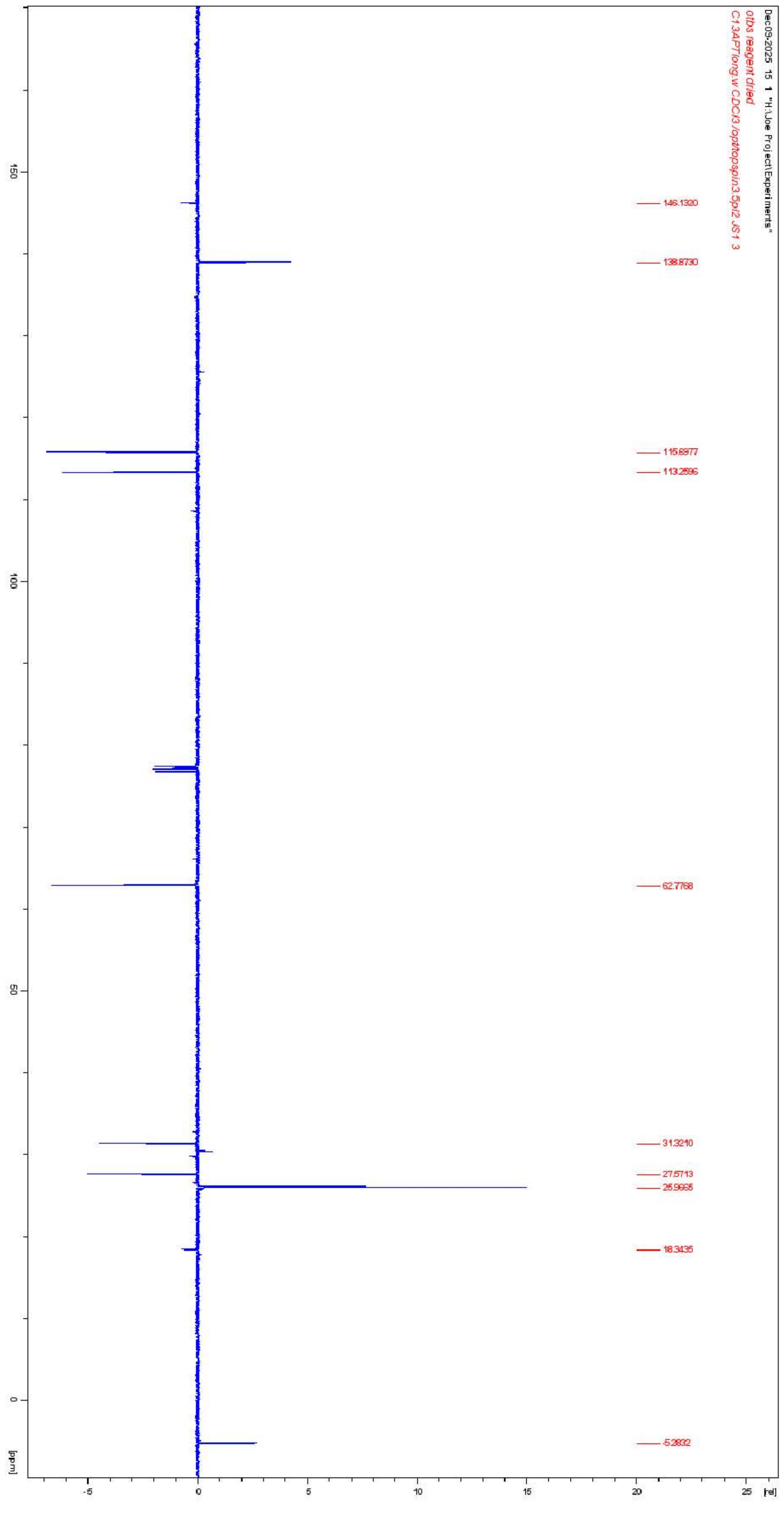

Figure S12: tert-Butyldimethyl((4-methylenehex-5-en-1-yl)oxy)silane (3) ^{13}C NMR (400 MHz, $CDCl_3$):

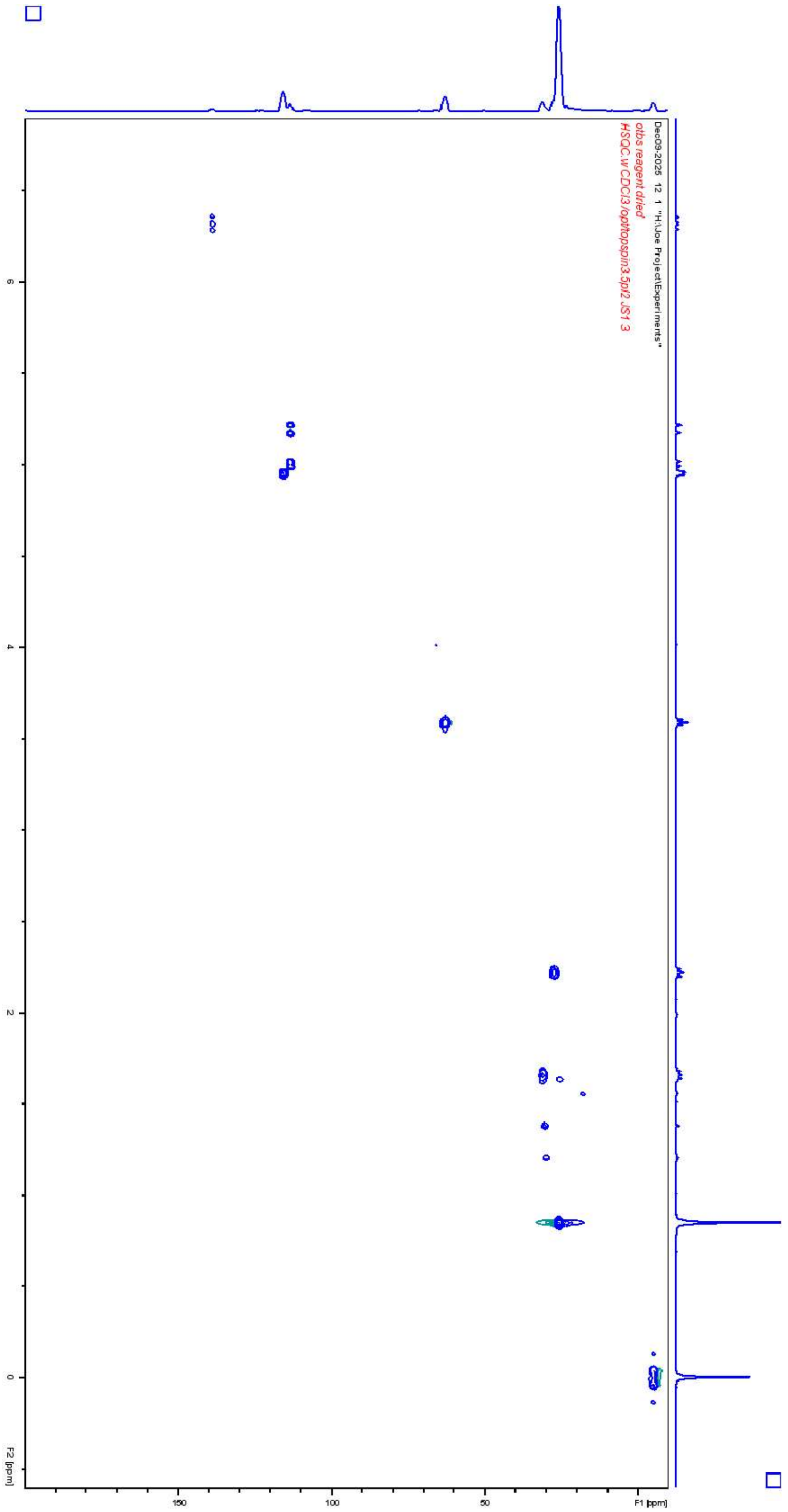

*Figure S13: tert-Butyldimethyl((4-methylenehex-5-en-1-yl)oxy)silane (**3**) HSQC NMR (400 MHz, $CDCl_3$):*

COSY NMR (400 MHz, CDCl₃):

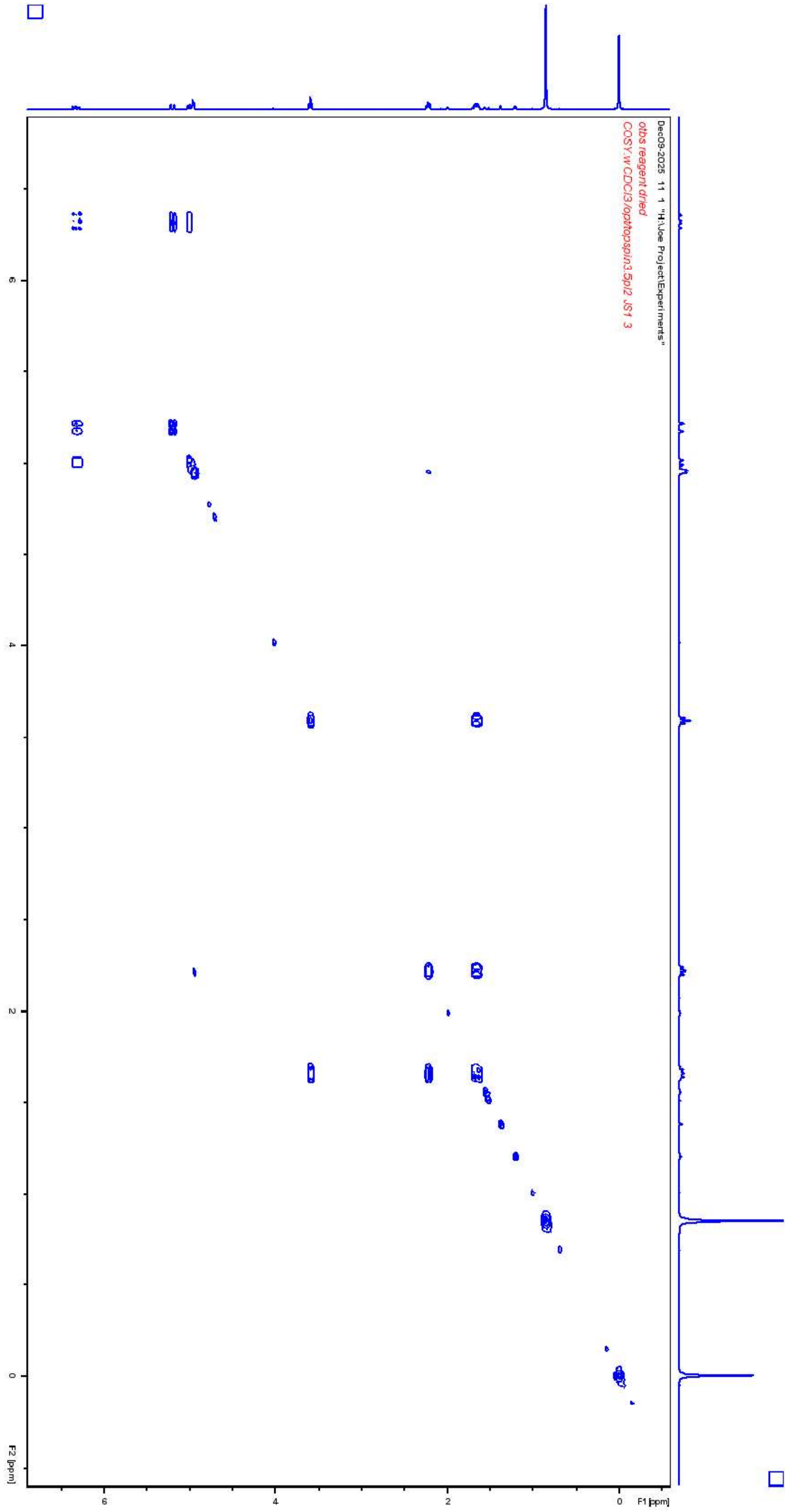

*Figure S14: tert-Butyldimethyl((4-methylenehex-5-en-1-yl)oxy)silane (**3**) COSY NMR (400 MHz, CDCl3):*

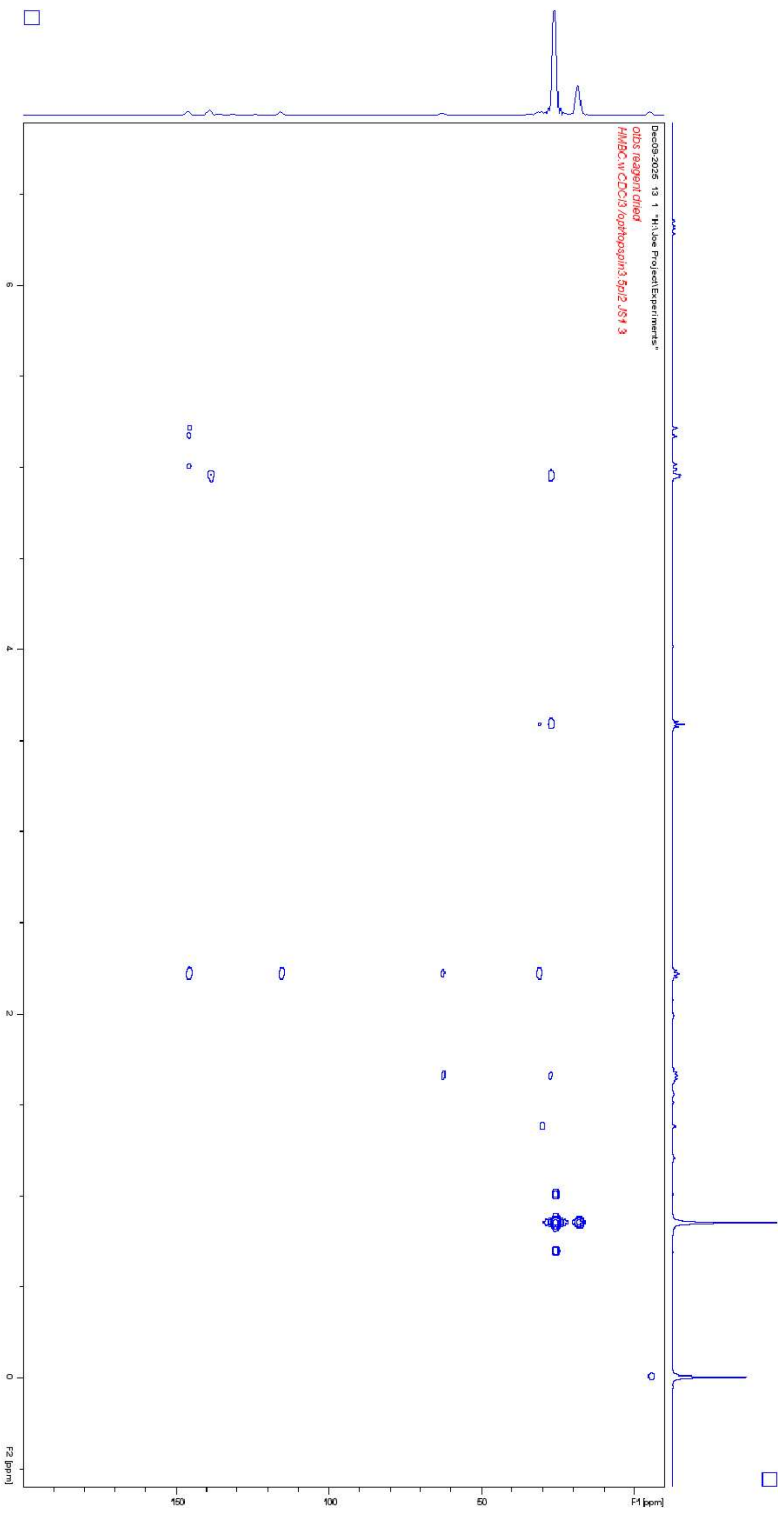

*Figure S15: tert-Butyldimethyl((4-methylenehex-5-en-1-yl)oxy)silane (**3**) HMBC NMR (400 MHz, CDCl3):*

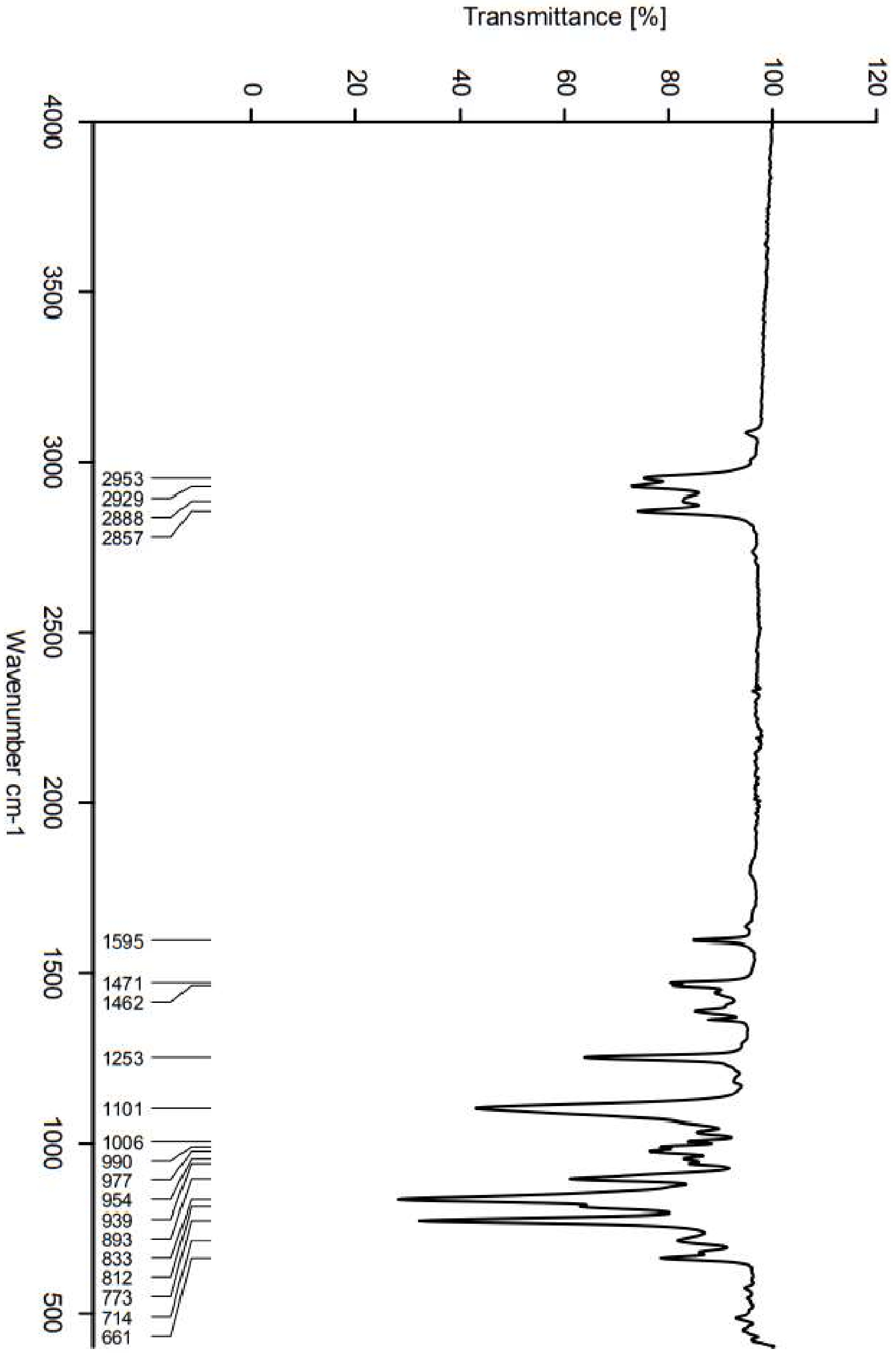

*Figure S16: tert-Butyldimethyl((4-methylenehex-5-en-1-yl)oxy)silane (**3**) IR Spectrum*

Mass Spectrum SmartFormula Report

Analysis Info

Analysis Name	D:\Data\SongDec2025\JS OTBS.d	Acquisition Date	12/12/2025 2:05:16 PM
Method	lowpositive.m	Operator	Demo User
Sample Name	6	Instrument	compact 8255754.20162
Comment			

Acquisition Parameter

Source Type	ESI	Ion Polarity	Positive	Set Nebulizer	0.3 Bar
Focus	Active	Set Capillary	4200 V	Set Dry Heater	200 °C
Scan Begin	50 m/z	Set End Plate Offset	-500 V	Set Dry Gas	4.0 l/min
Scan End	3000 m/z	Set Charging Voltage	2000 V	Set Divert Valve	Source
		Set Corona	0 nA	Set APCI Heater	0 °C

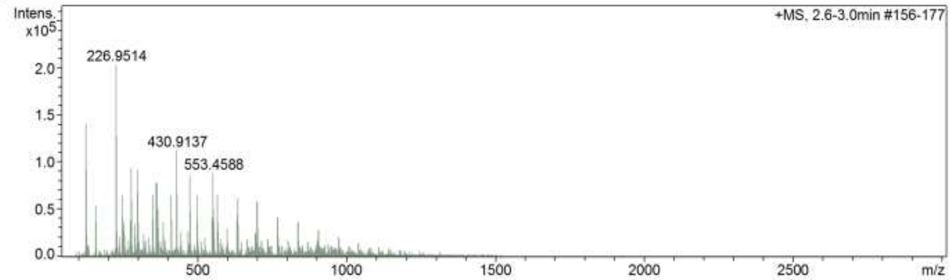

Meas. m/z	#	Ion Formula	m/z	err [ppm]	mSigma	# mSigma	Score	rdb	e⁻ Conf	N-Rule
249.1642	1	C13H26NaOSi	249.1645	1.3	25.0	1	100.00	2.0	even	ok

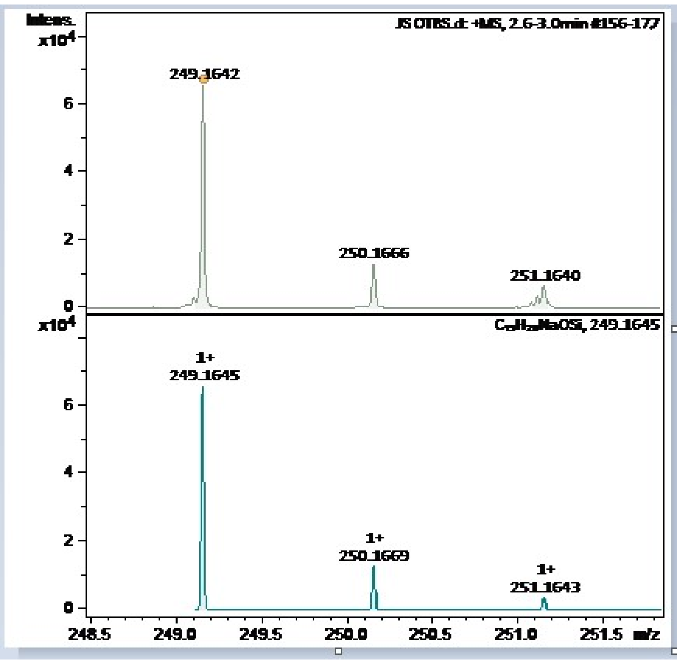

Figure S17: HRMS-ESI full analysis report (top) and observed peaks (bottom).

4 Homopolymer Data

HOMO - TBS

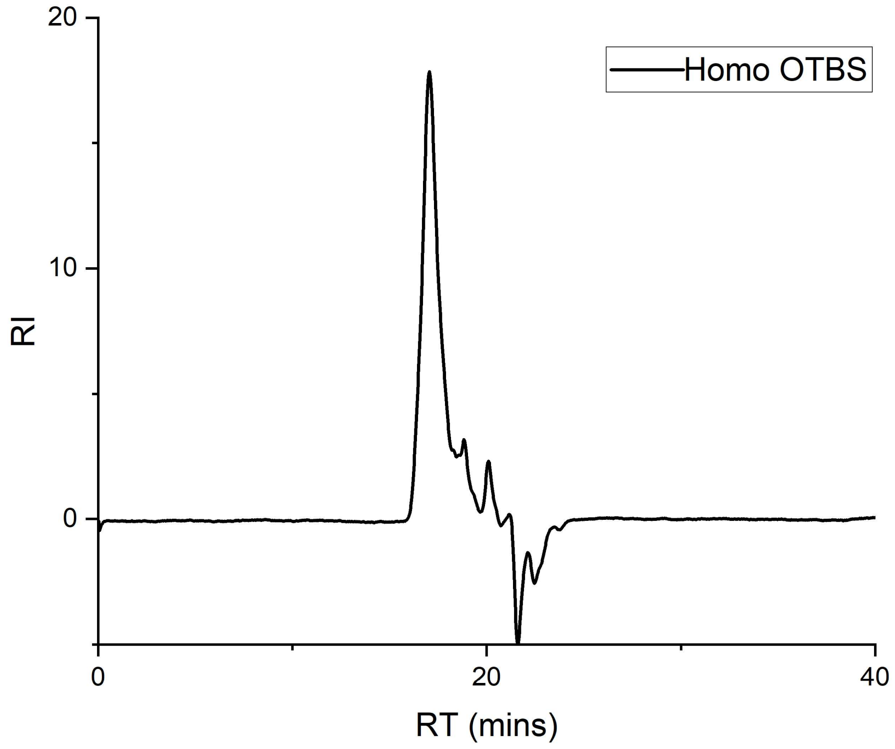

-

Figure S18: Homo-TBS GPC Trace

Table S5: MWt values obtained from GPC analysis of Homo-TBS

MW Averages							
Peaks	M_p (g/mol)	M_n (g/mol)	M_w (g/mol)	M_z (g/mol)	M_{z+1} (g/mol)	M_v (g/mol)	**Đ**
Peak 1	2910	2160	2890	3630	4350	2780	1.34

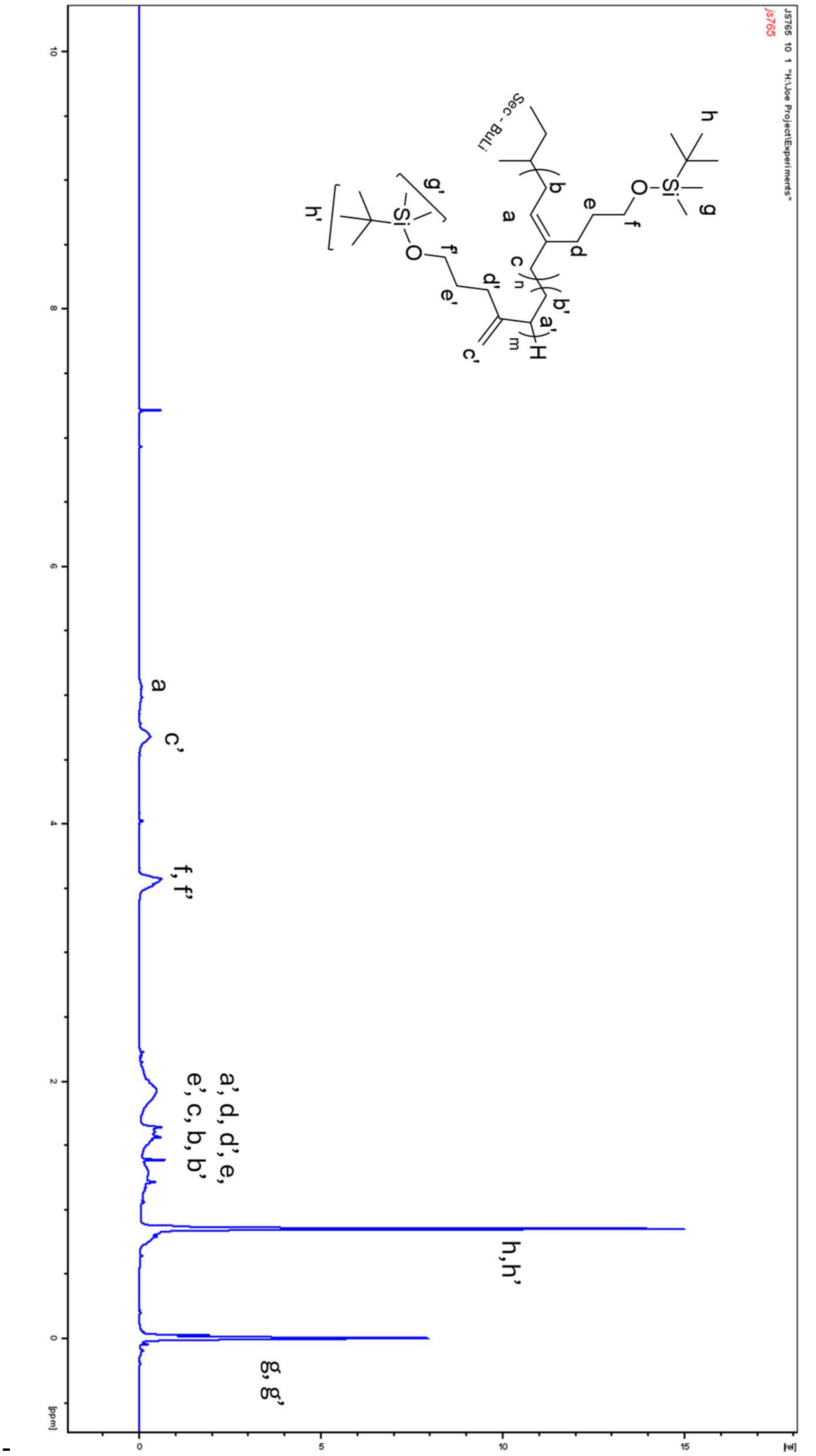

Figure S19: ^{1}H NMR (400 MHz, $CDCl_3$) spectrum of HOMO - TBS

-

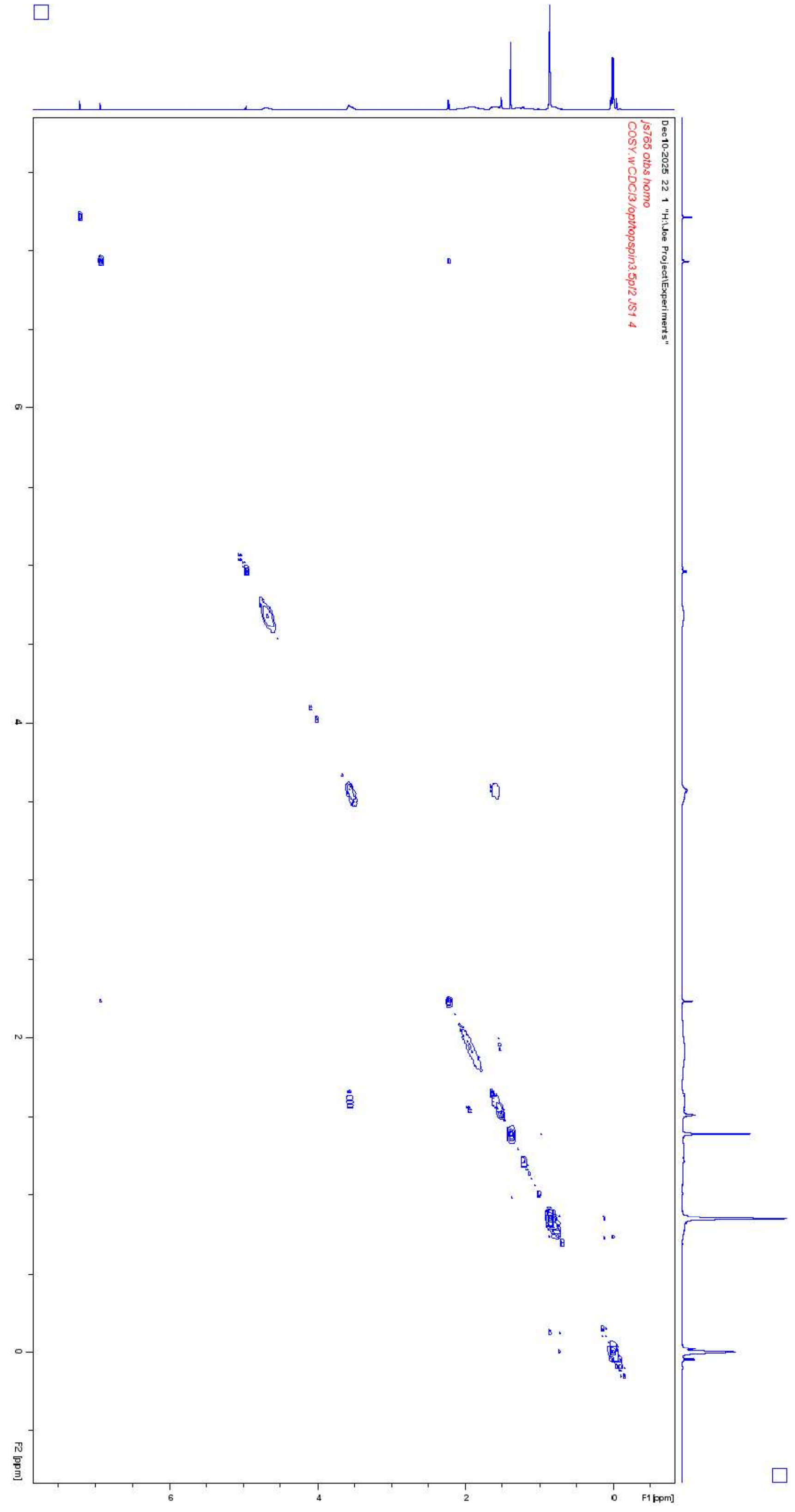

Figure S20: COSY NMR spectrum (400 MHz, $CDCl_3$) HOMO- TBS

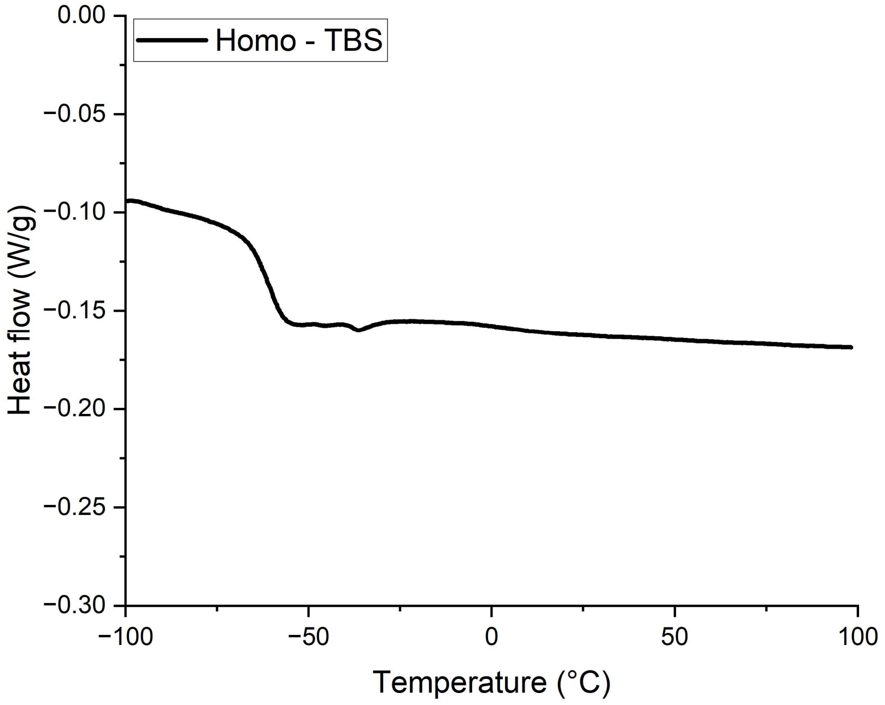

-

Figure S21: DSC (2nd Heating cycle) Homo - TBS

-

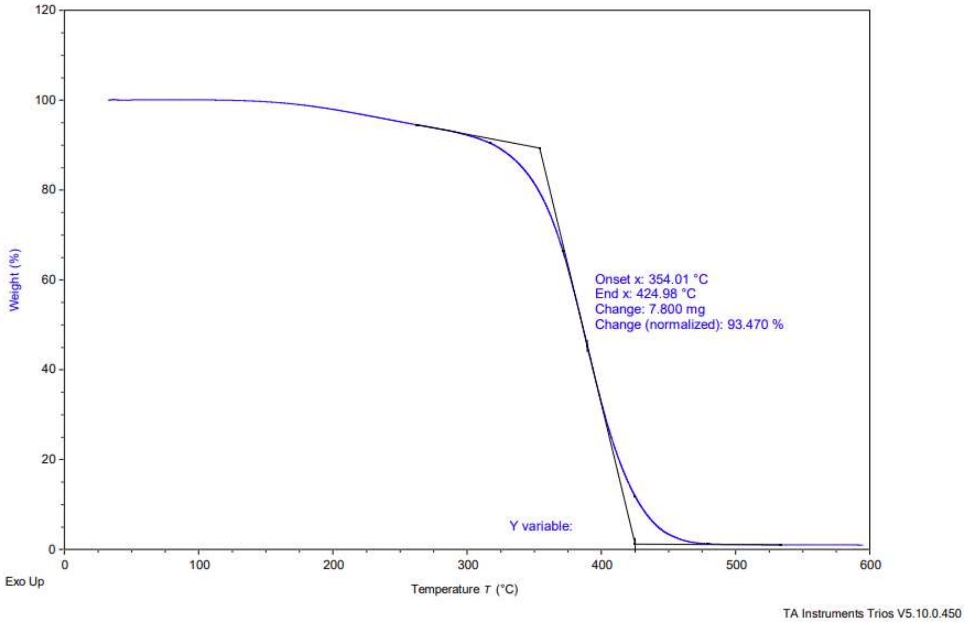

Figure S22: TGA Trace of Homo-TBS

HOMO - Bn:

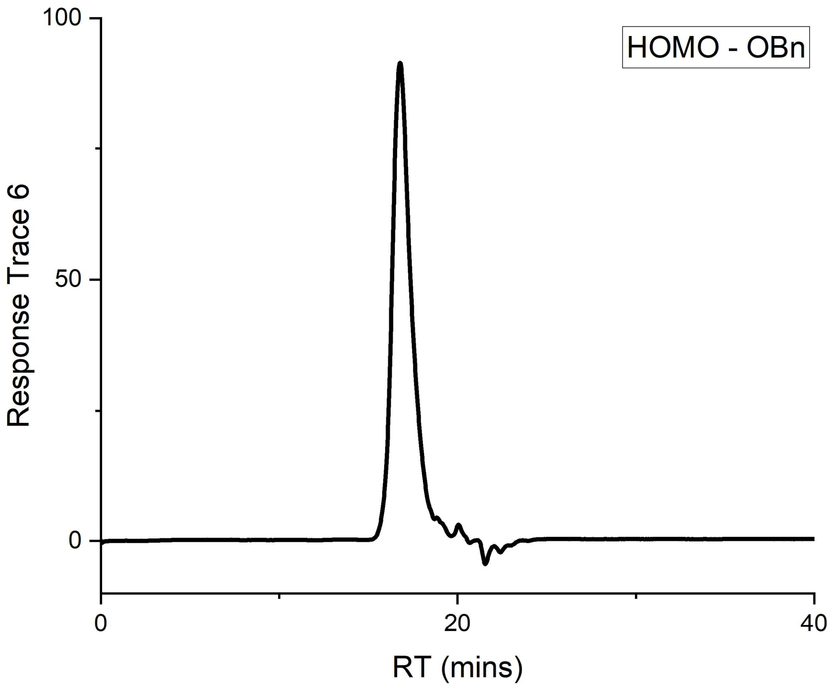

-

Figure S23: GPC Trace of Homo-Bn

Table S6: MWt Values given by GPC analysis of Homo-Bn

MW Averages							
Peaks	Mp (g/mol)	Mn (g/mol)	Mw (g/mol)	Mz (g/mol)	Mz+1 (g/mol)	Mv (g/mol)	**Đ**
Peak 1	3940	2490	3850	5220	6690	3650	1.544

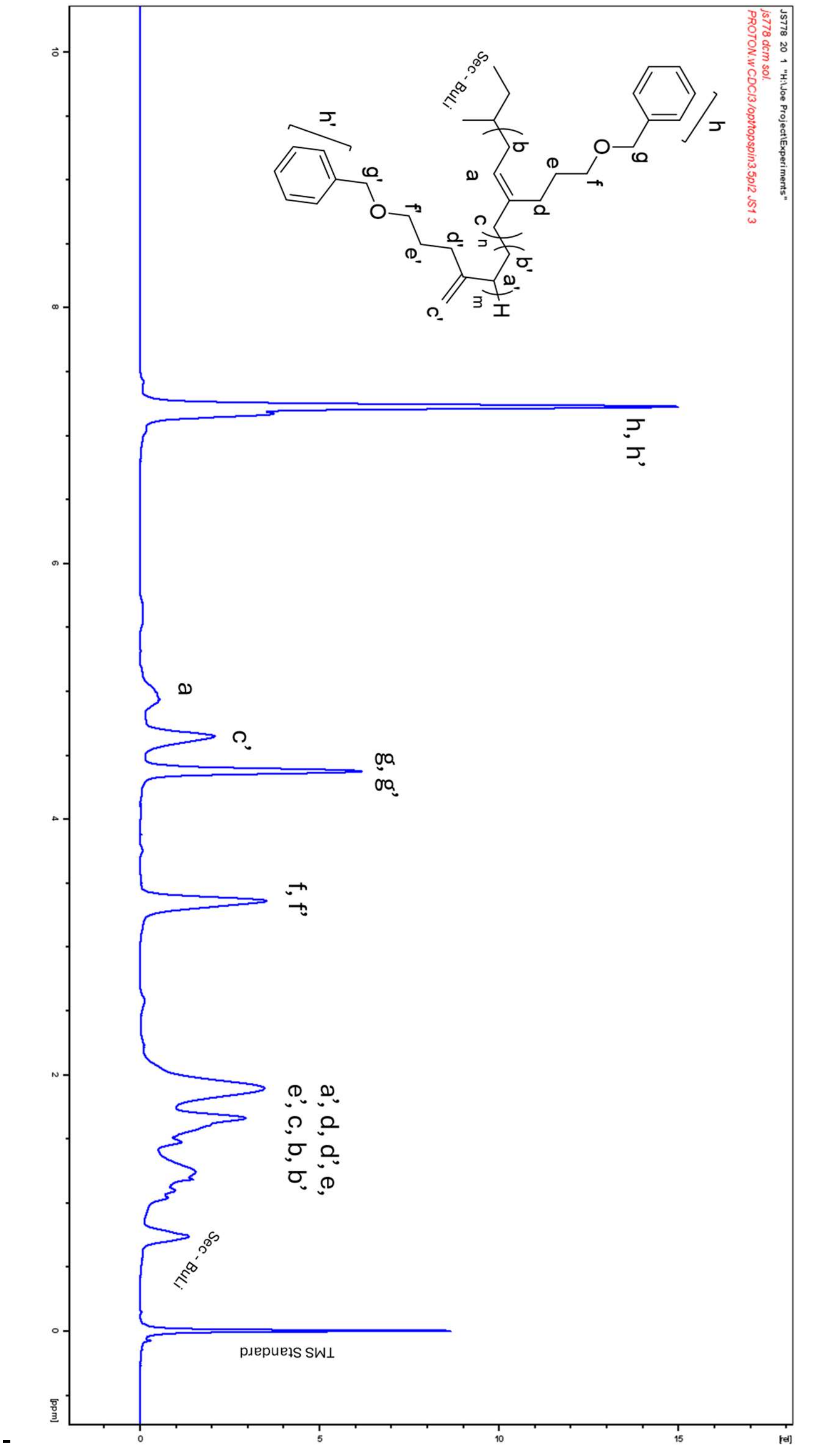

- *Figure S24: -^{1}H NMR* ***(400 MHz, $CDCl_3$): HOMO - Bn***

-

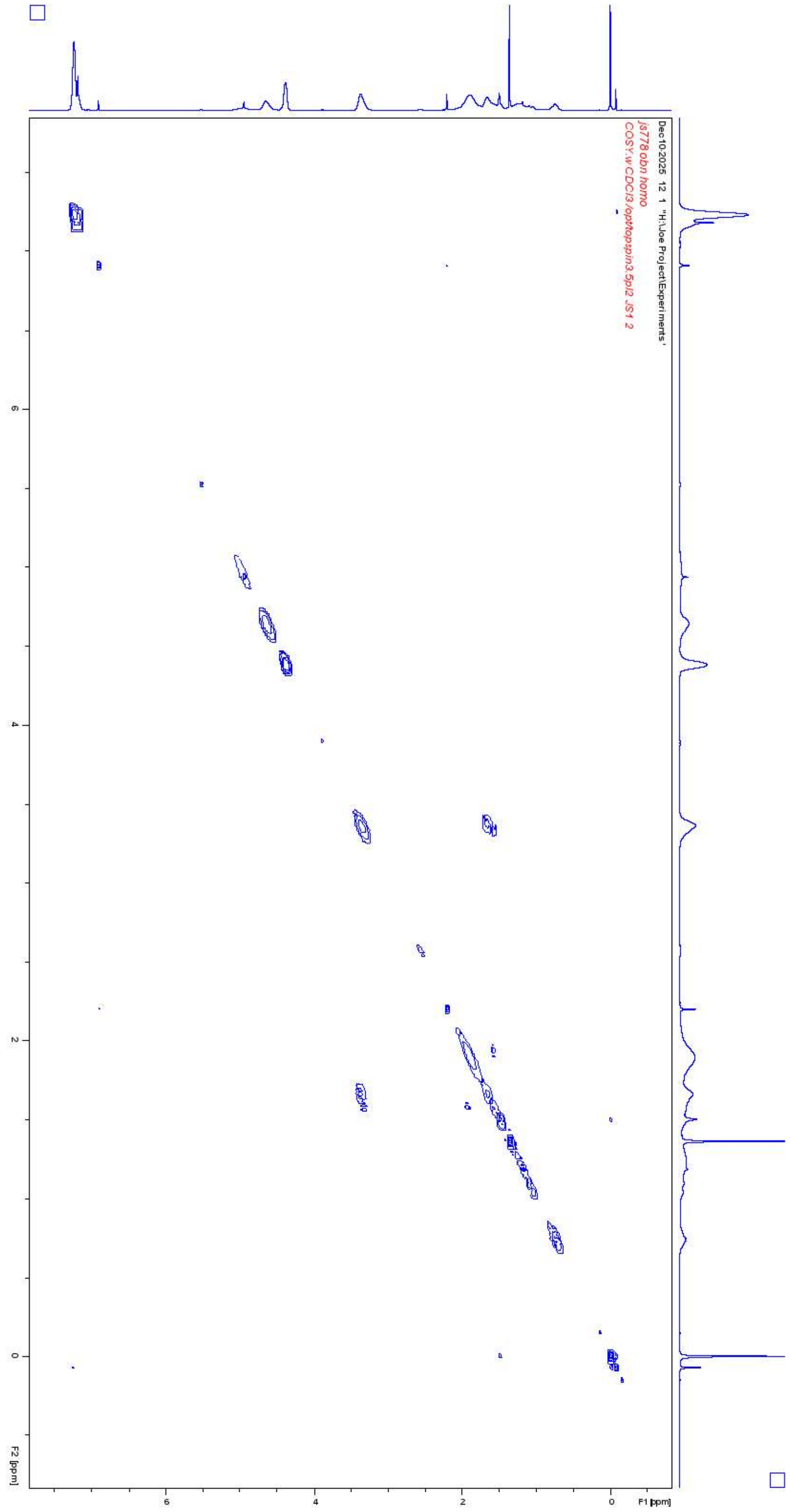

Figure S25: COSY NMR spectrum (400 MHz, $CDCl_3$) HOMO - Bn

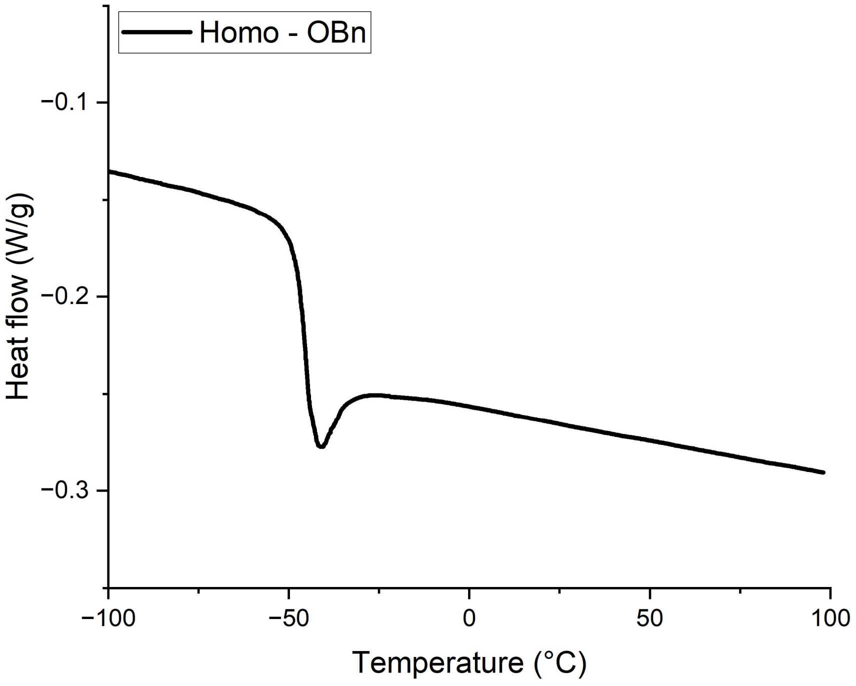

-

Figure S26: DSC (2nd Heating cycle) Trace of Homo-Bn

-

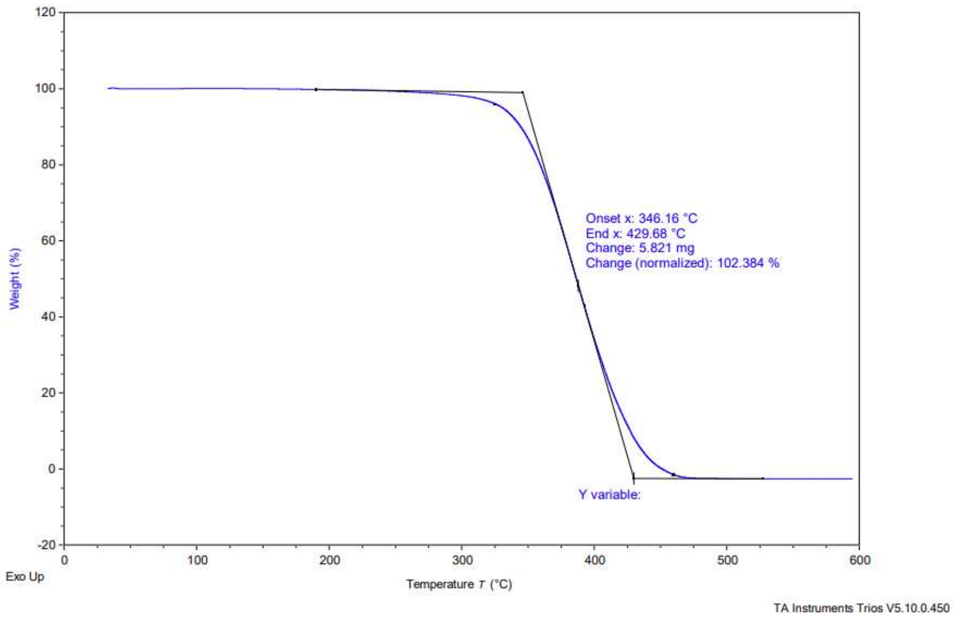

-

Figure S27: TGA Analysis of Homo-Bn

6. POLYENE STANDARDS DATA

3,4-Polyisoprene (3,4-PI)

- GPC

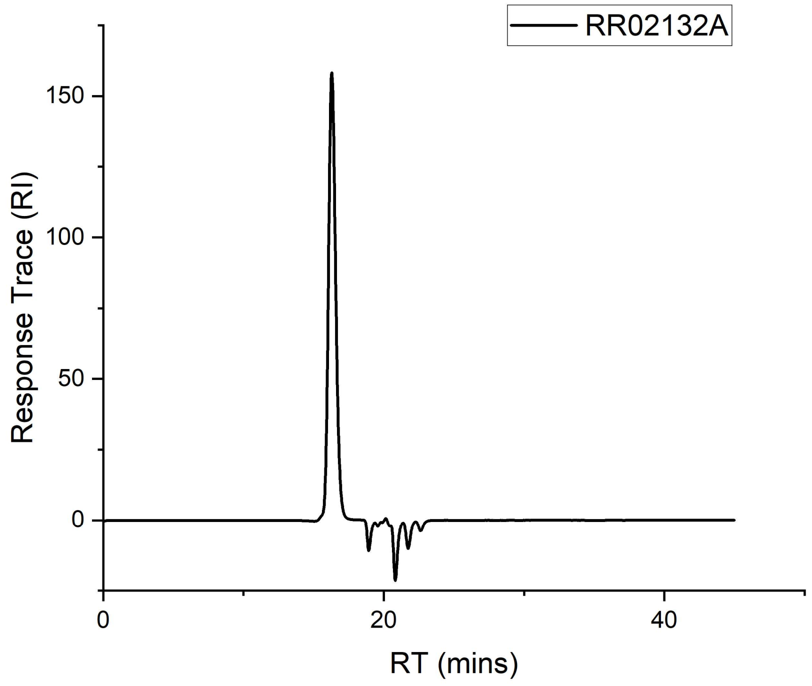

Figure S28: GPC Trace of 3,4-PI

Table S7: MWt Values obtained from GPC Analysis of 3,4-PI.

MW Averages							
Peaks	Mp (g/mol)	Mn (g/mol)	Mw (g/mol)	Mz (g/mol)	Mz+1 (g/mol)	Mv (g/mol)	**Đ**
Peak 1	3790	3570	3800	4030	4260	3770	1.06

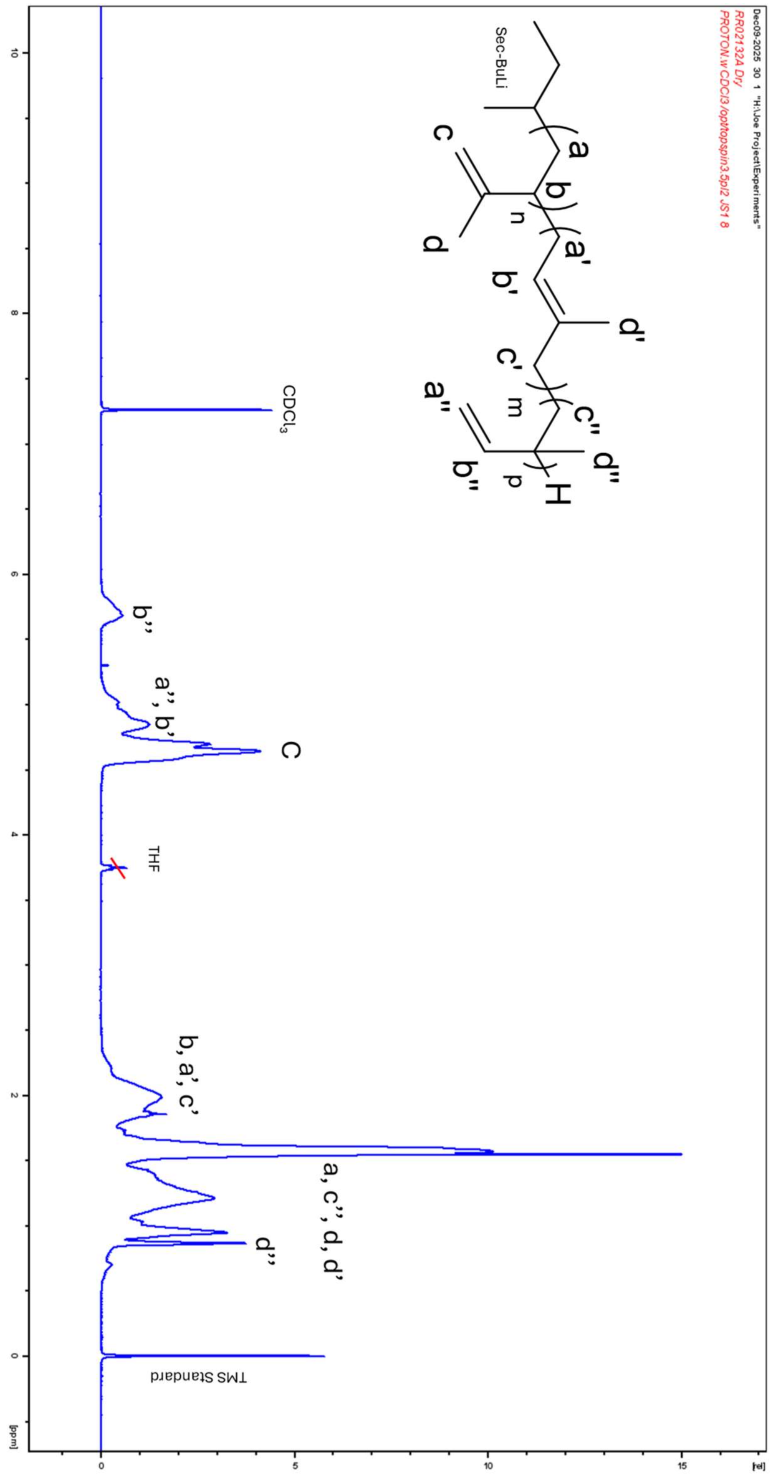

- *Figure S29: ^{1}H NMR* ***(400 MHz, $CDCl_3$): 3,4-polyisoprene***

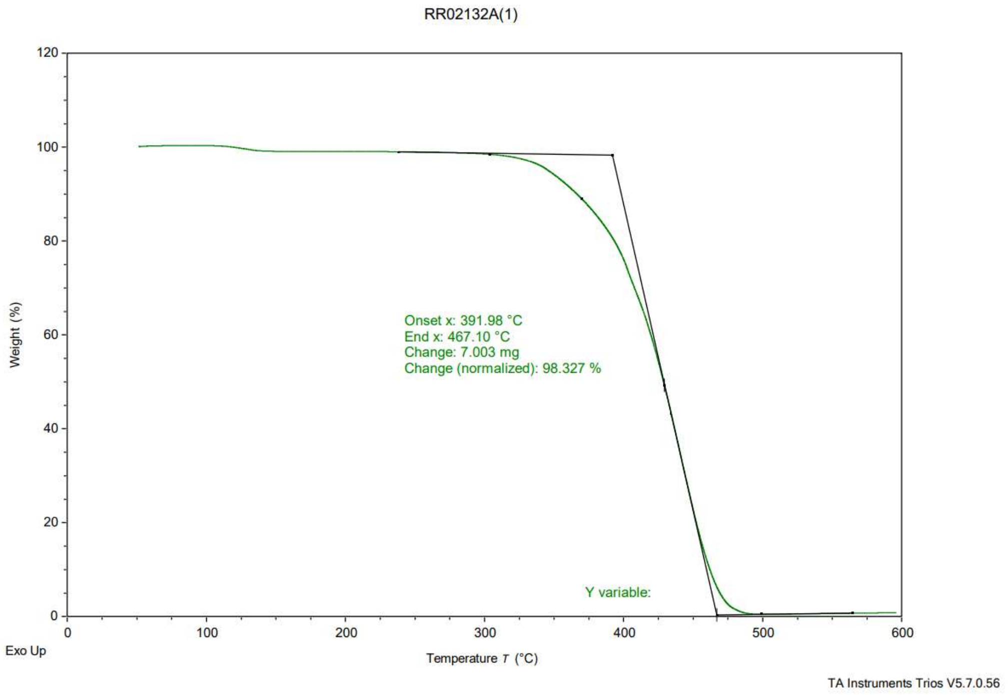

Figure S30: TGA Trace of 3,4-PI

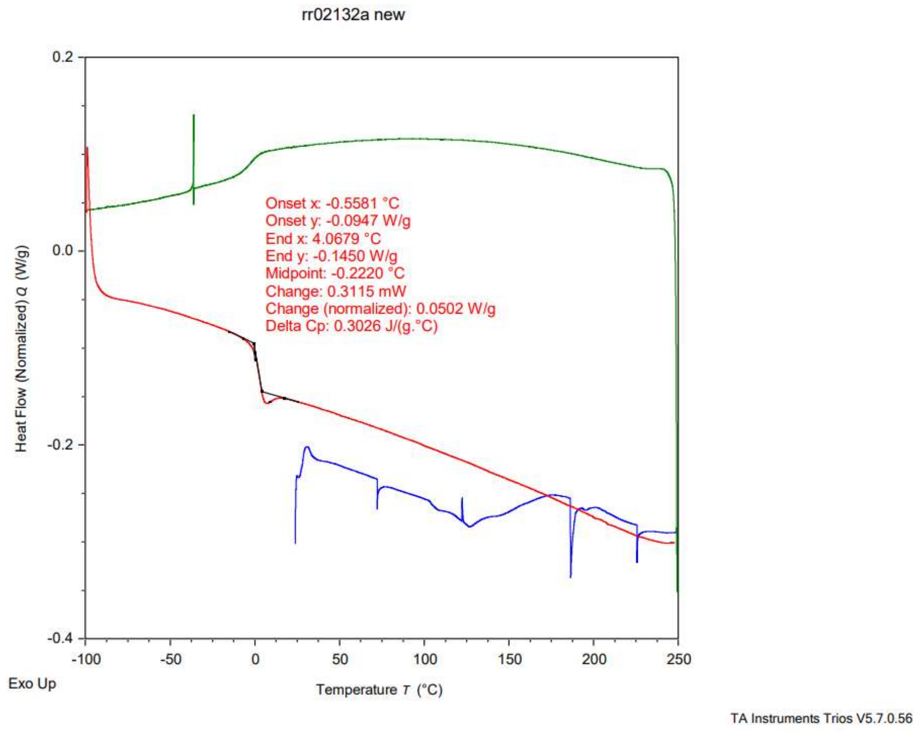

Figure S31: DSC Analysis of 3,4-PI

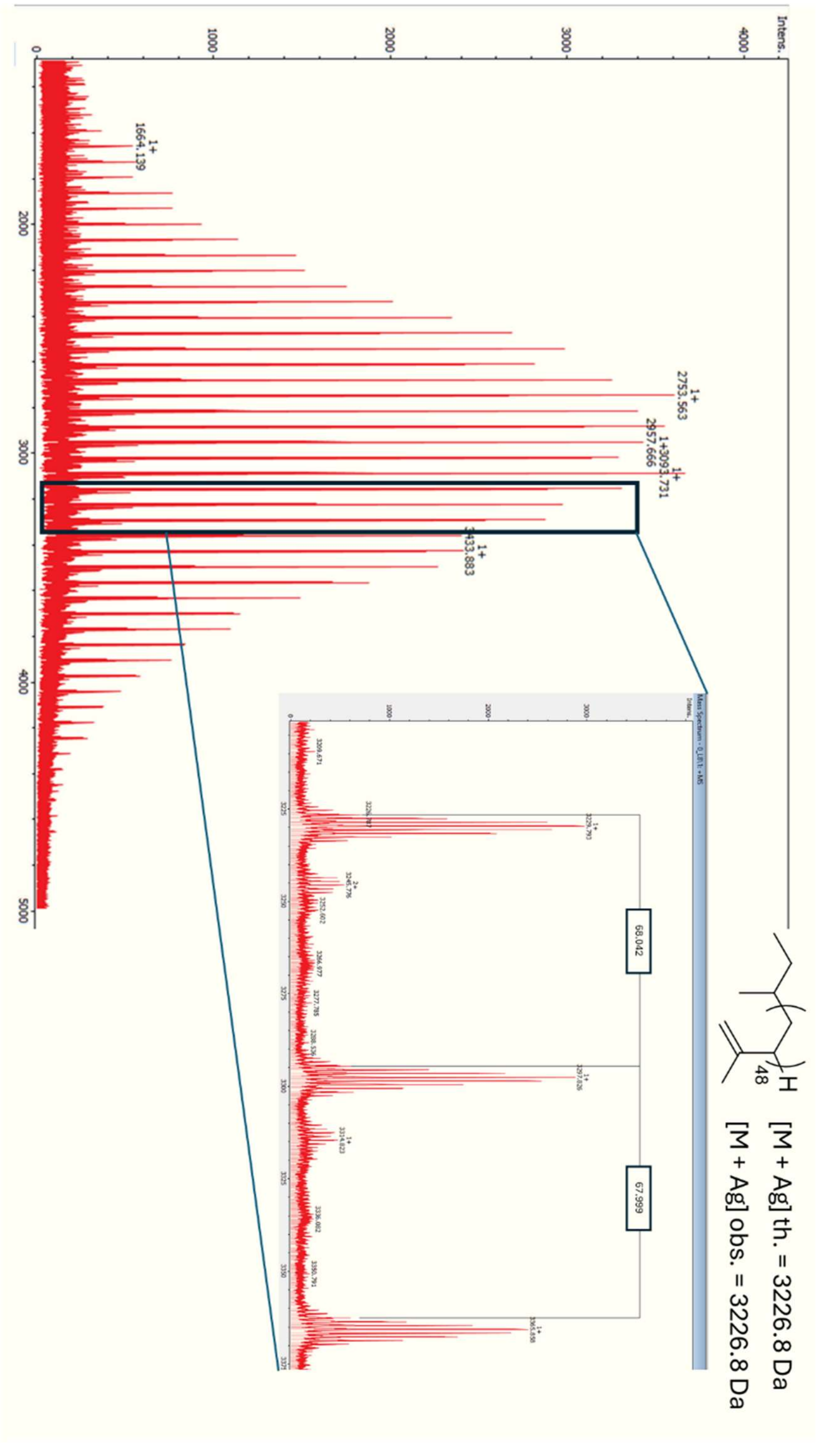

Figure S32: MALDI-TOF Spectrum 3,4-polyisoprene

1,4 Polyisoprene

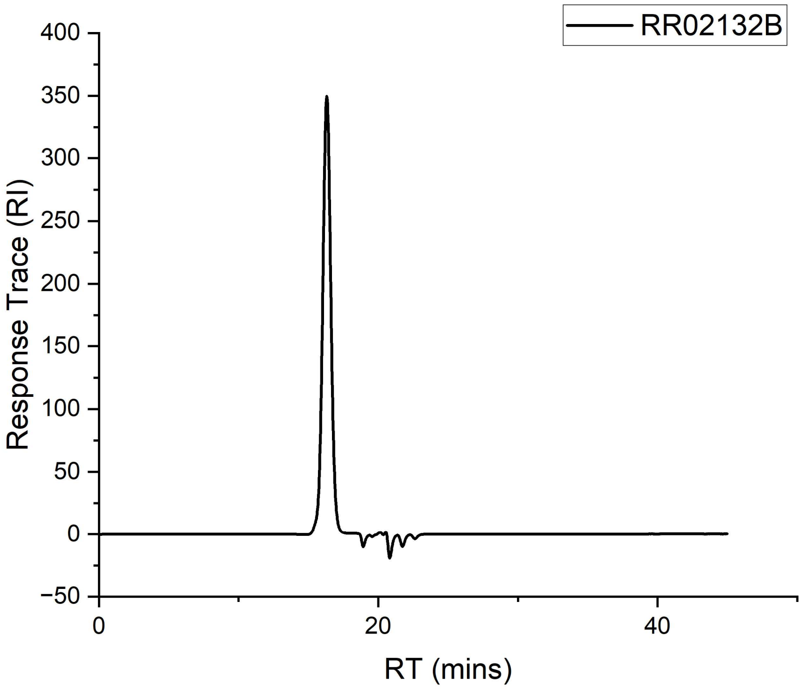

-

Figure S33: GPC Trace of 1,4-PI

Table S8: MWt values obtained from GPC analysis of 1,4-PI

MW Averages							
Peaks	Mp (g/mol)	Mn (g/mol)	Mw (g/mol)	Mz (g/mol)	Mz+1 (g/mol)	Mv (g/mol)	**Đ**
Peak 1	3730	3610	3910	4250	4650	3870	1.08

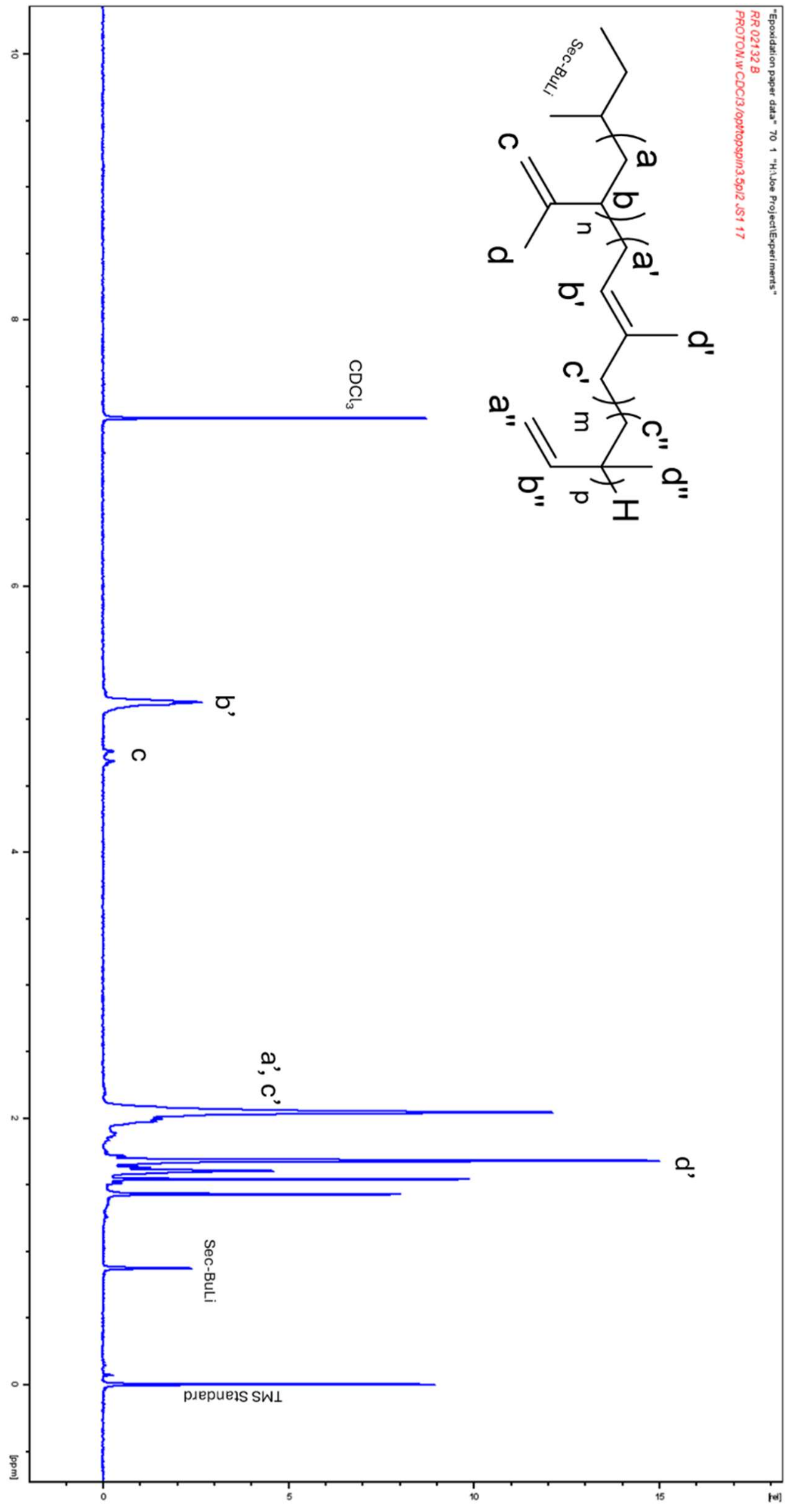

- *Figure S34: ^{1}H NMR **(400 MHz, $CDCl_3$): 1,4-polyisoprene***

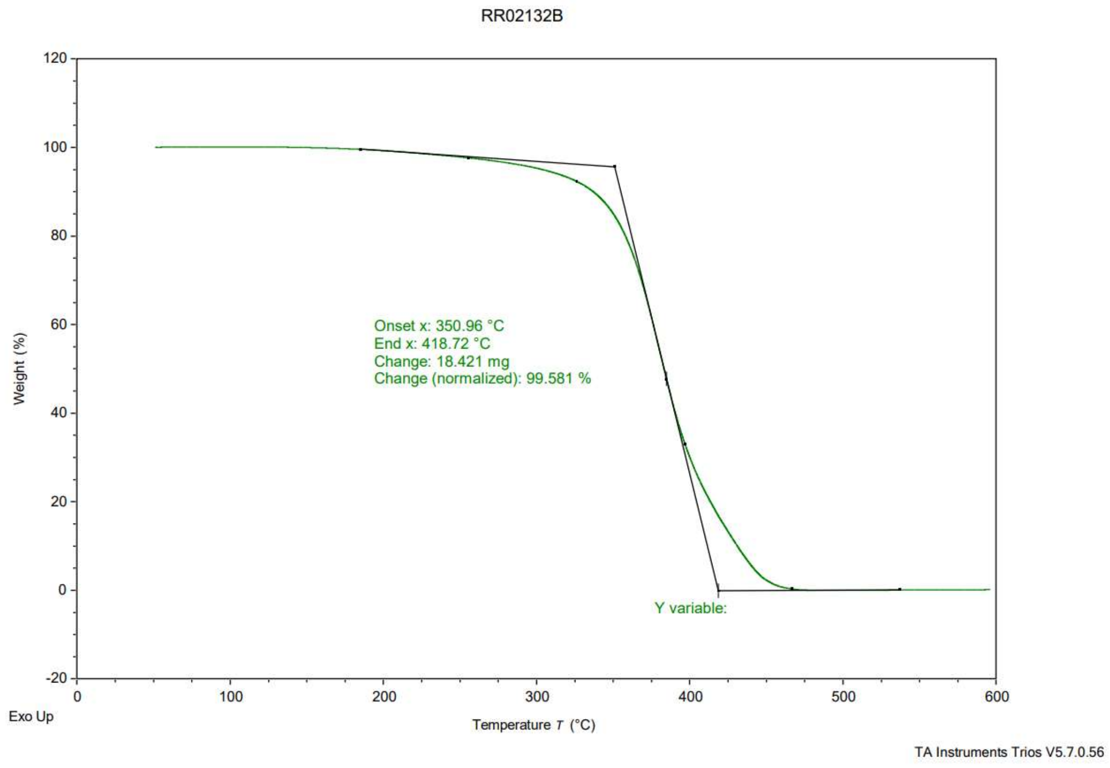

Figure S35: TGA Analysis of 1,4-PI

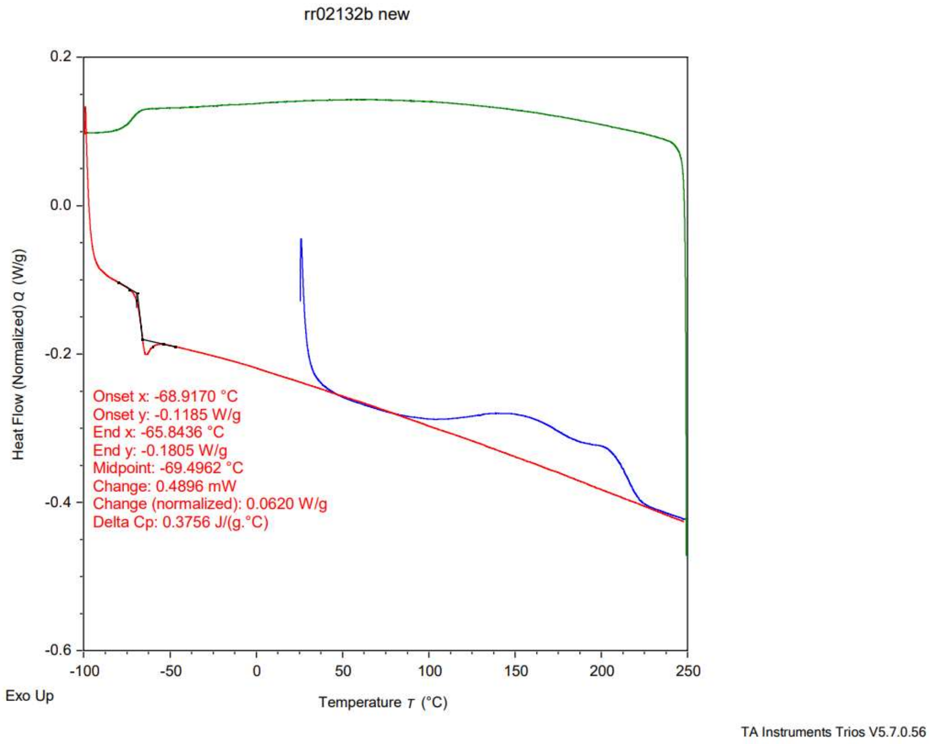

Figure S36: DSC Analysis of 1,4-PI

-

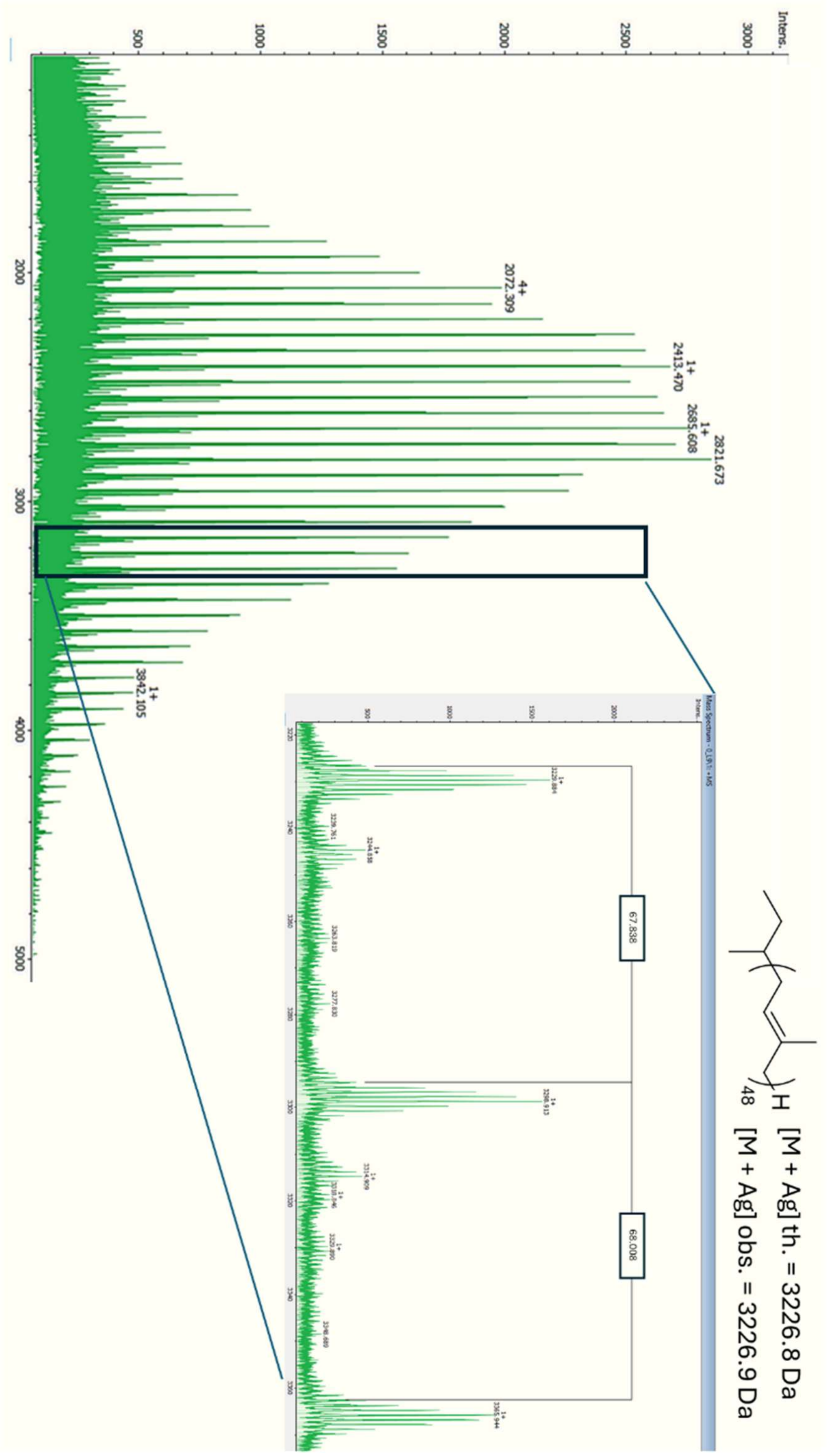

- *Figure S37: MALDI-TOF Spectrum 1,4-polyisoprene*

3,4-polymyrcene (3,4-PM)

-

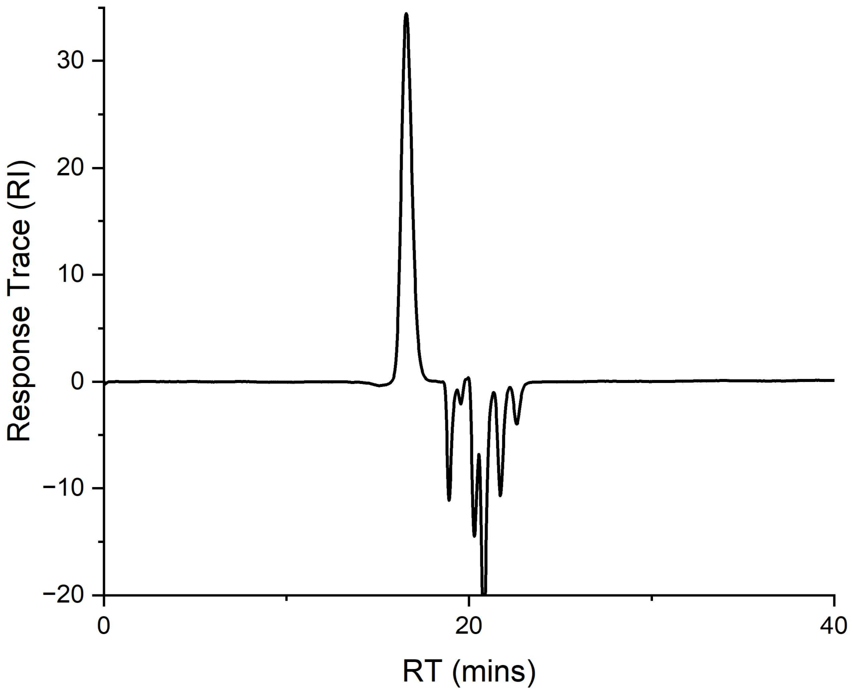

Figure S38: GPC Trace of 3,4-PM

Table S9: MWt Values obtained from GPC Analysis of 3,4-PM

MW Averages							
Peaks	Mp (g/mol)	Mn (g/mol)	Mw (g/mol)	Mz (g/mol)	Mz+1 (g/mol)	Mv (g/mol)	PD
Peak 1	2931	2720	2930	3126	3322	2896	1.08

-

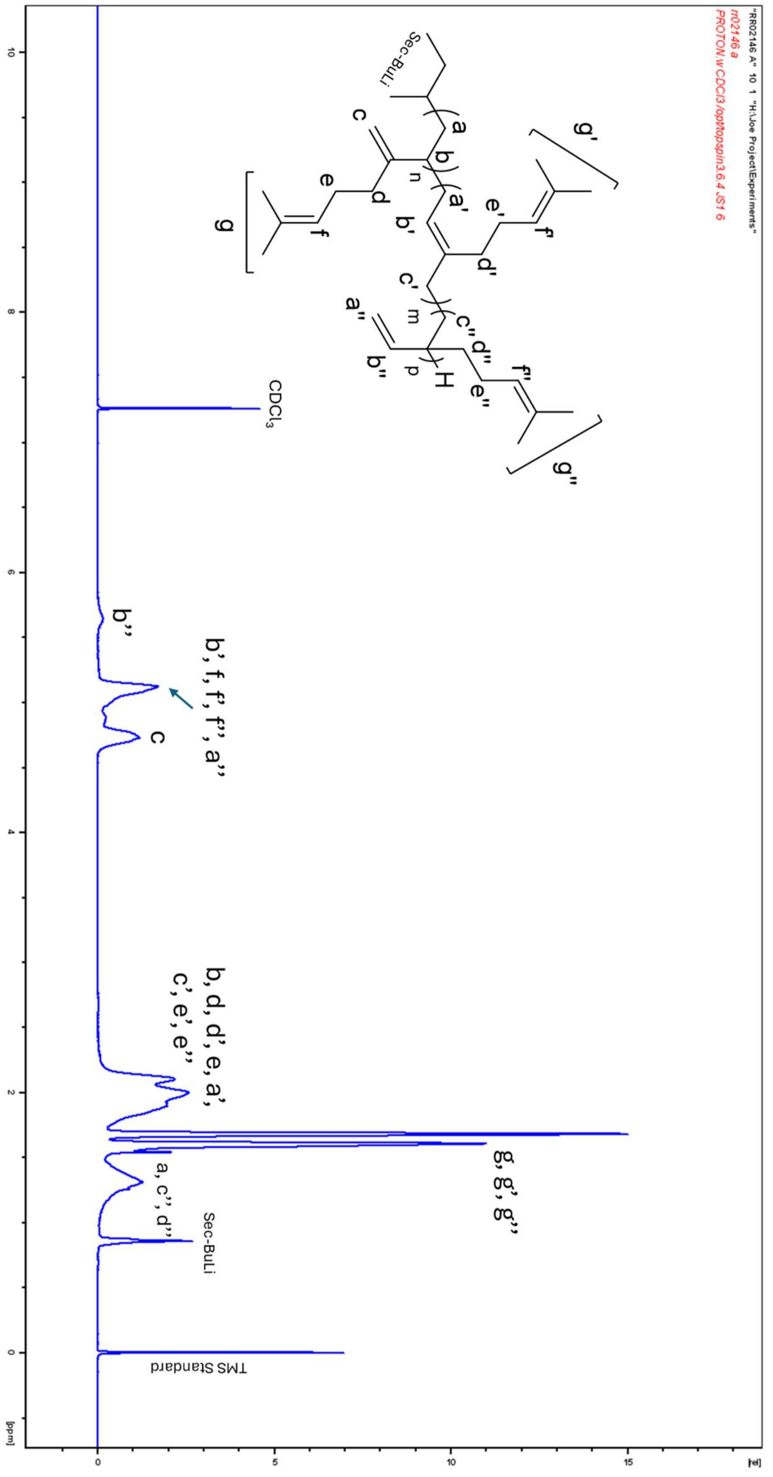

- *Figure S39: ¹H NMR **(400 MHz, CDCl$_3$): 3,4-polymyrcene***

-

-

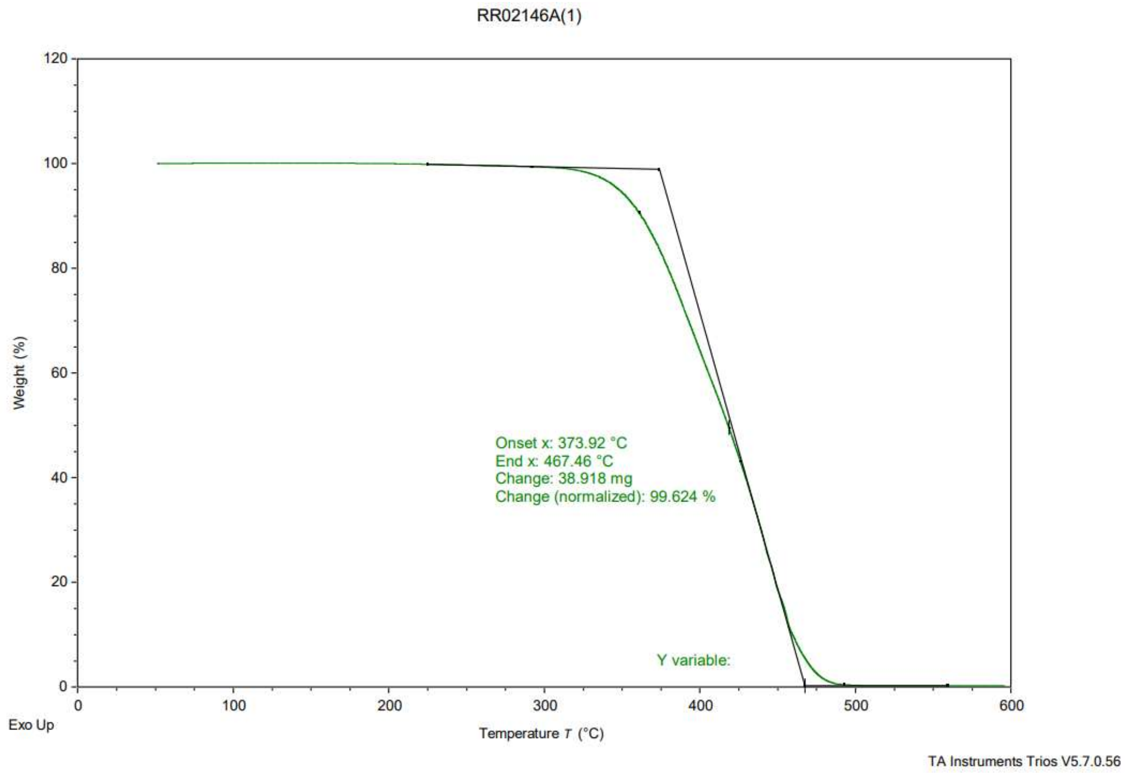

Figure S40: TGA Analysis trace of 3,4-PM.

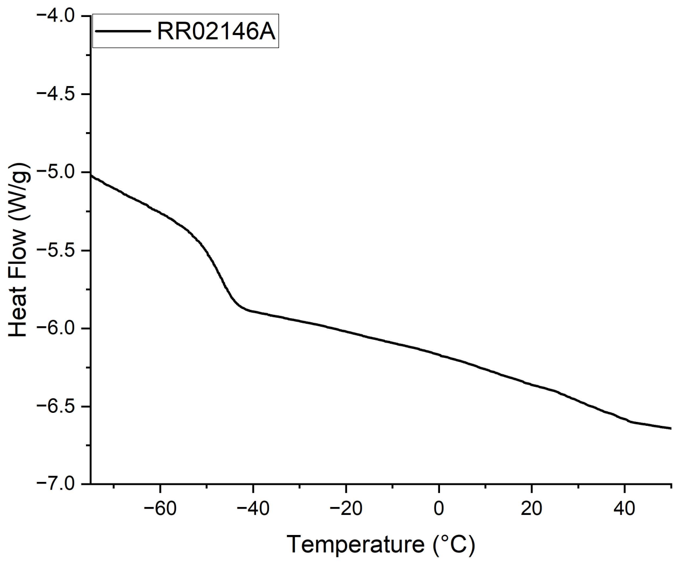

-

Figure S41: DSC (2nd Heating cycle) Trace of 3,4-PM.

-

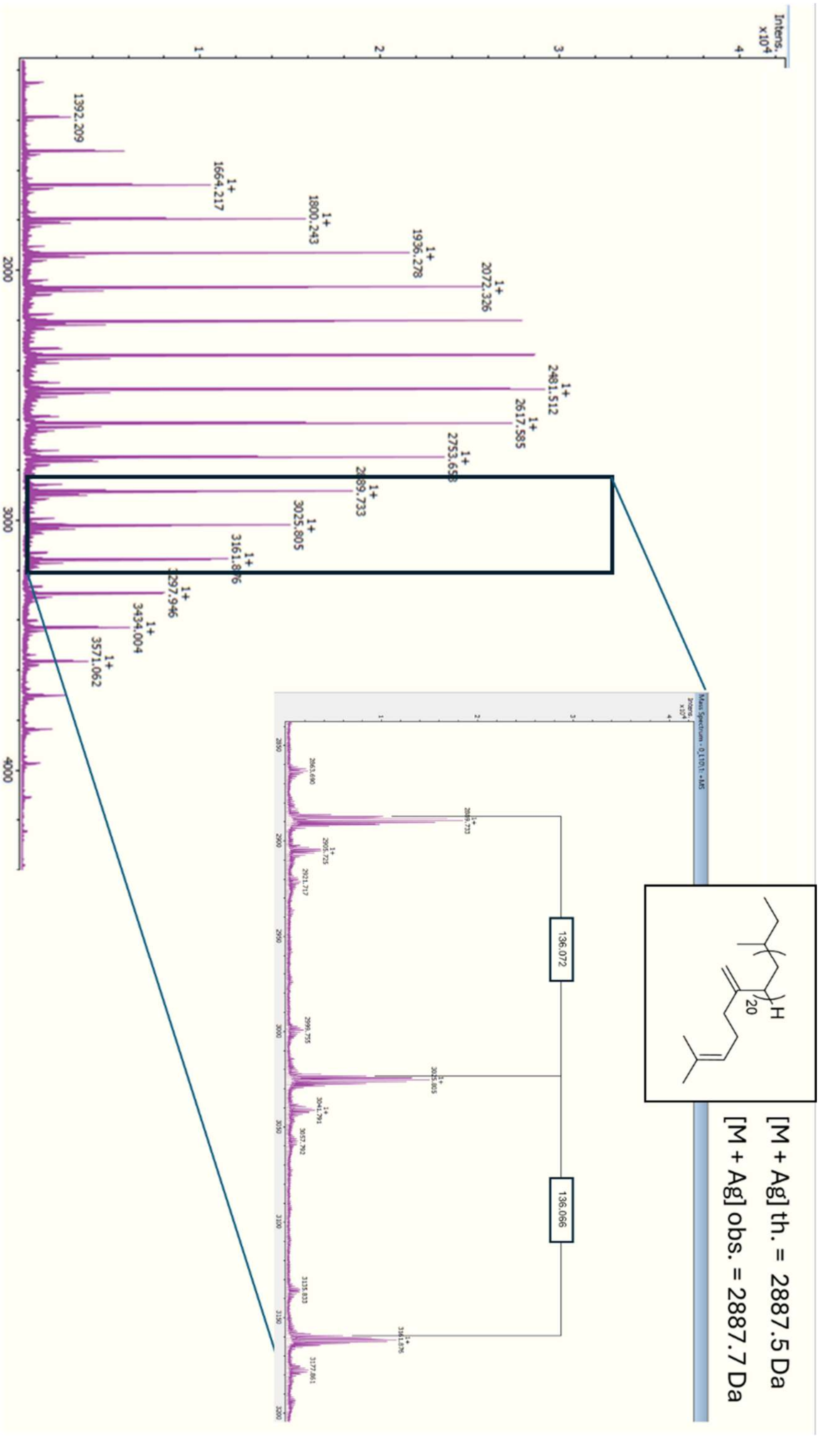

- *Figure S42: MALDI-TOF Spectrum* ***3,4-polymyrcene***

1,4 Polymyrcene (1,4-PM)

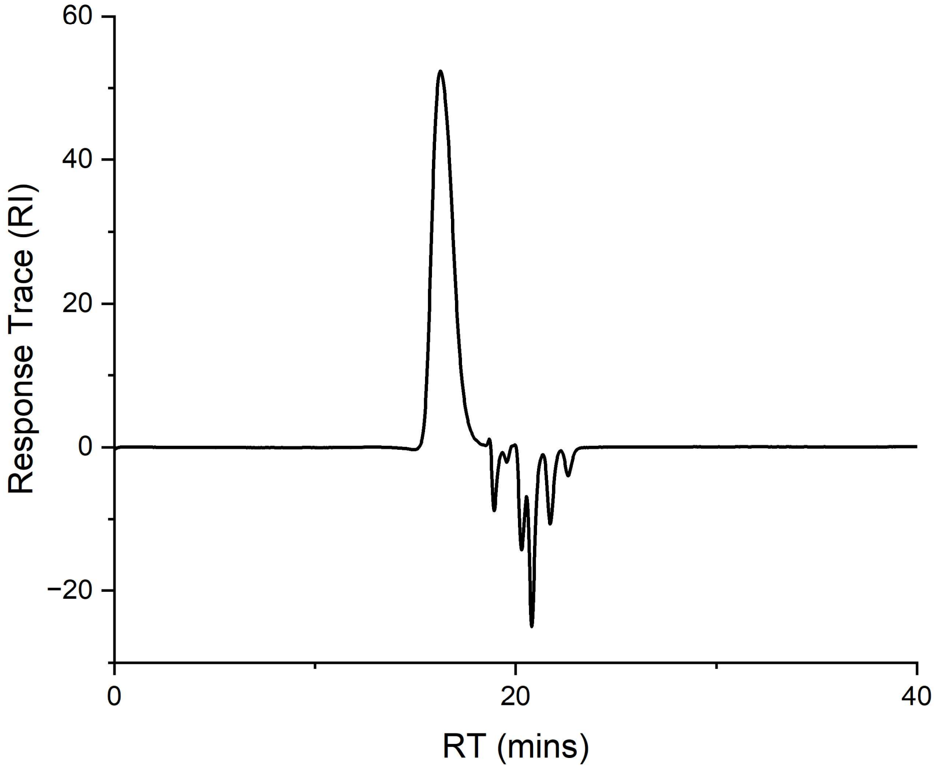

-

Figure S43: GPC Trace of 1,4-PM

Table S10: MWt values obtained from GPC analysis of 1,4-PM

MW Averages							
Peaks	Mp (g/mol)	Mn (g/mol)	Mw (g/mol)	Mz (g/mol)	Mz+1 (g/mol)	Mv (g/mol)	**Đ**
Peak 1	3980	3020	3760	4470	5130	3660	1.25

-

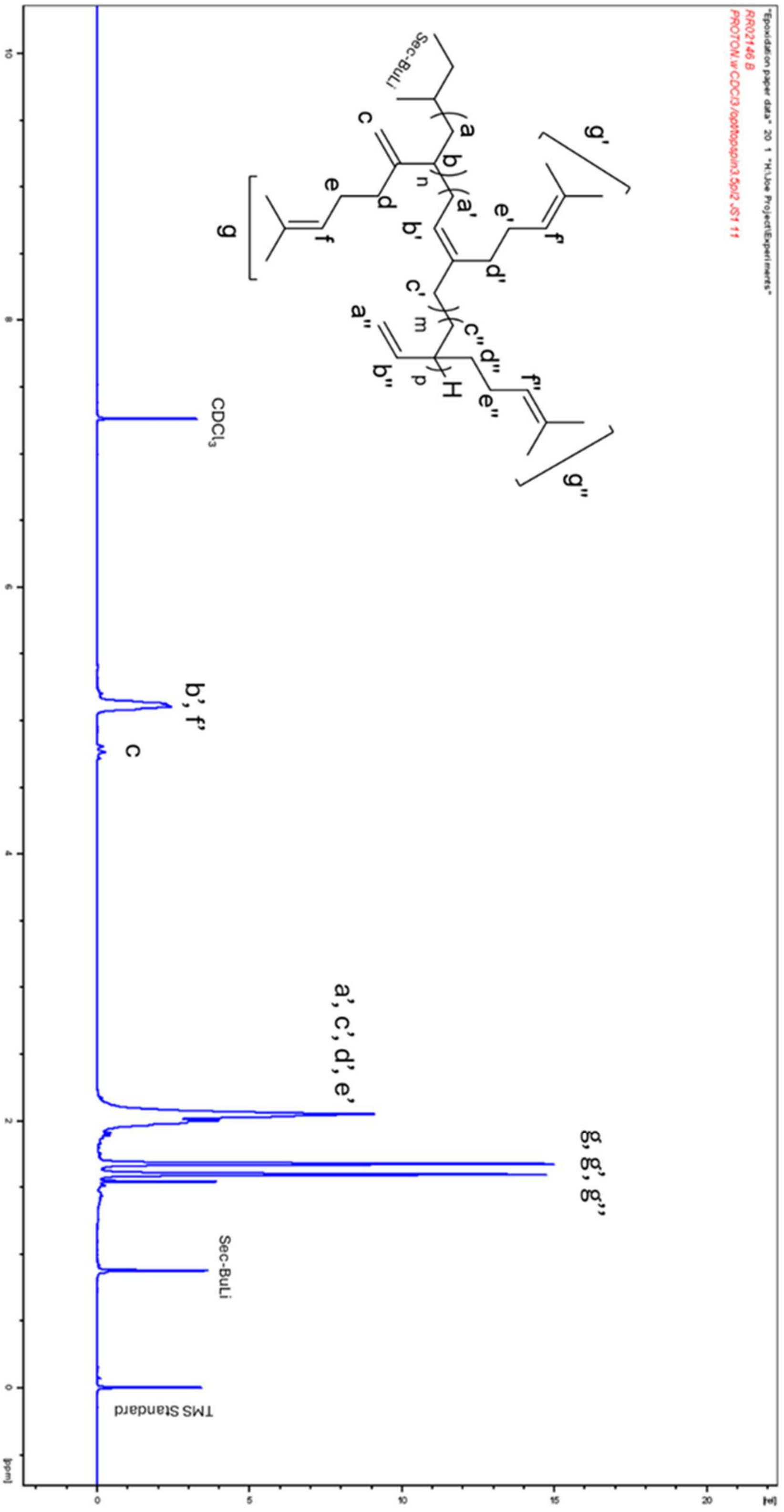

- *Figure S44: ^{1}H NMR* ***(400 MHz, $CDCl_3$): 1,4-polymyrcene***

-

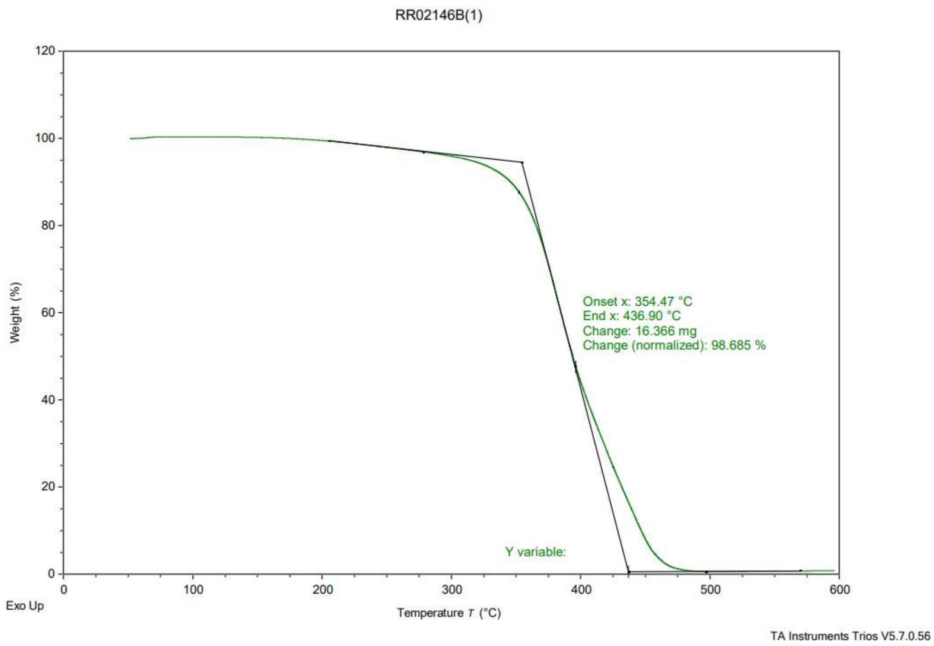

Figure S45: TGA Analysis of 1,4-PM

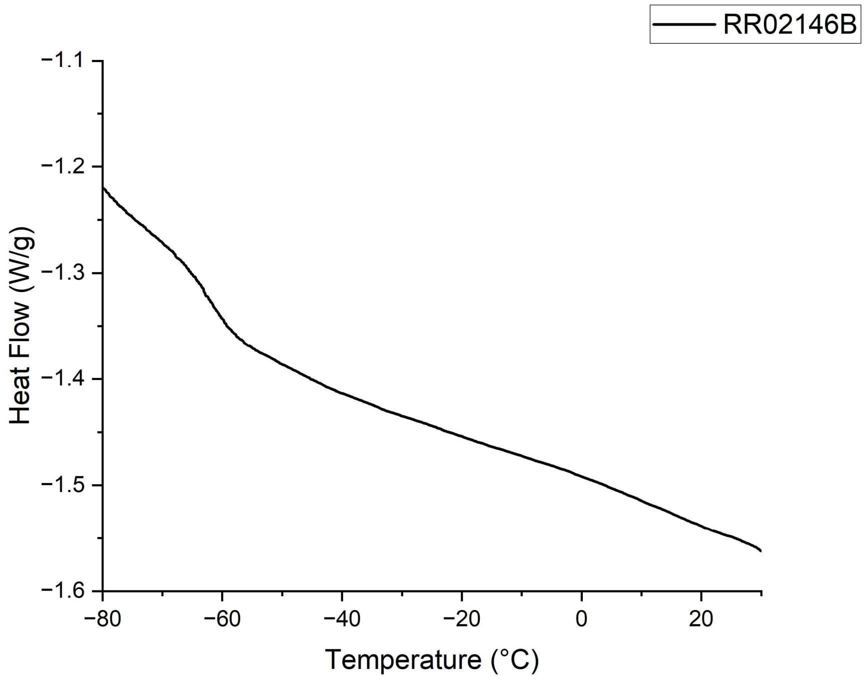

Figure S46: DSC (2nd Heating cycle) of 1,4-PM

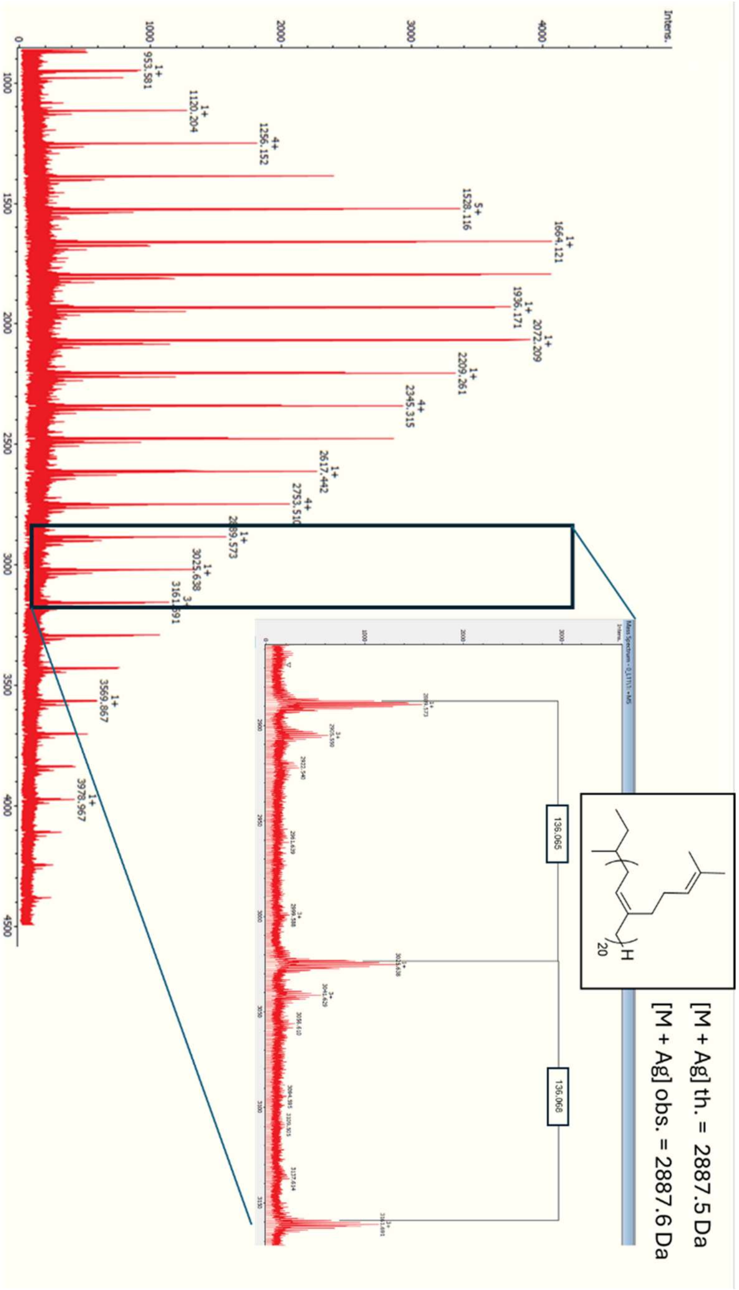

Figure S47: MALDI-TOF Spectrum 1,4-polymyrcene

7 COPOLYMER DATA

Copol 1

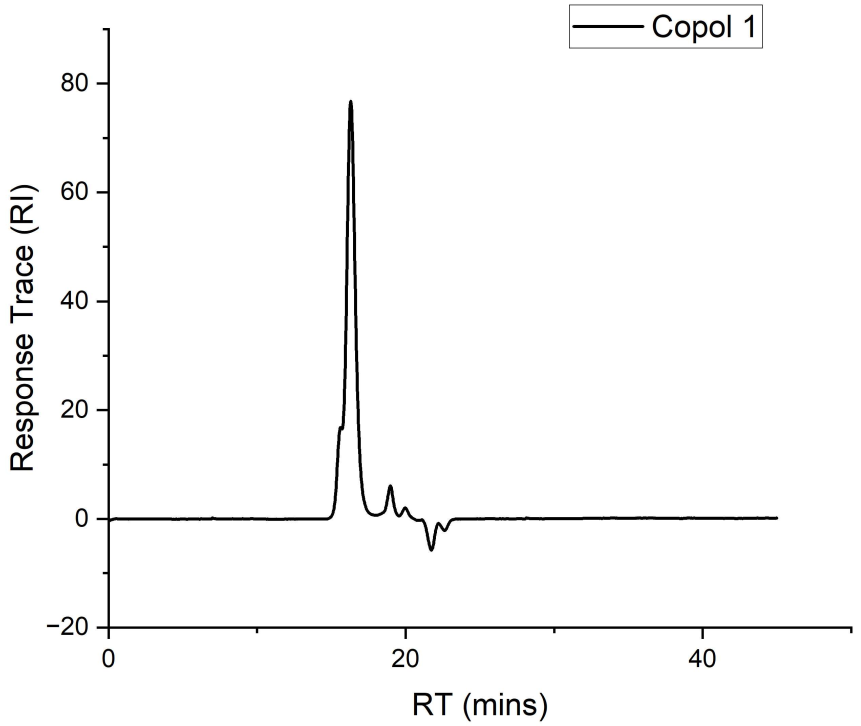

-

Figure S48: GPC Trace of Copol 1

Table S11 : MWt values obtained from the GPC Analysis of Copol 1

MW Averages							
Peaks	Mp (g/mol)	Mn (g/mol)	Mw (g/mol)	Mz (g/mol)	Mz+1 (g/mol)	Mv (g/mol)	**Đ**
Peak 1	5430	5120	6200	7560	9250	6020	1.21

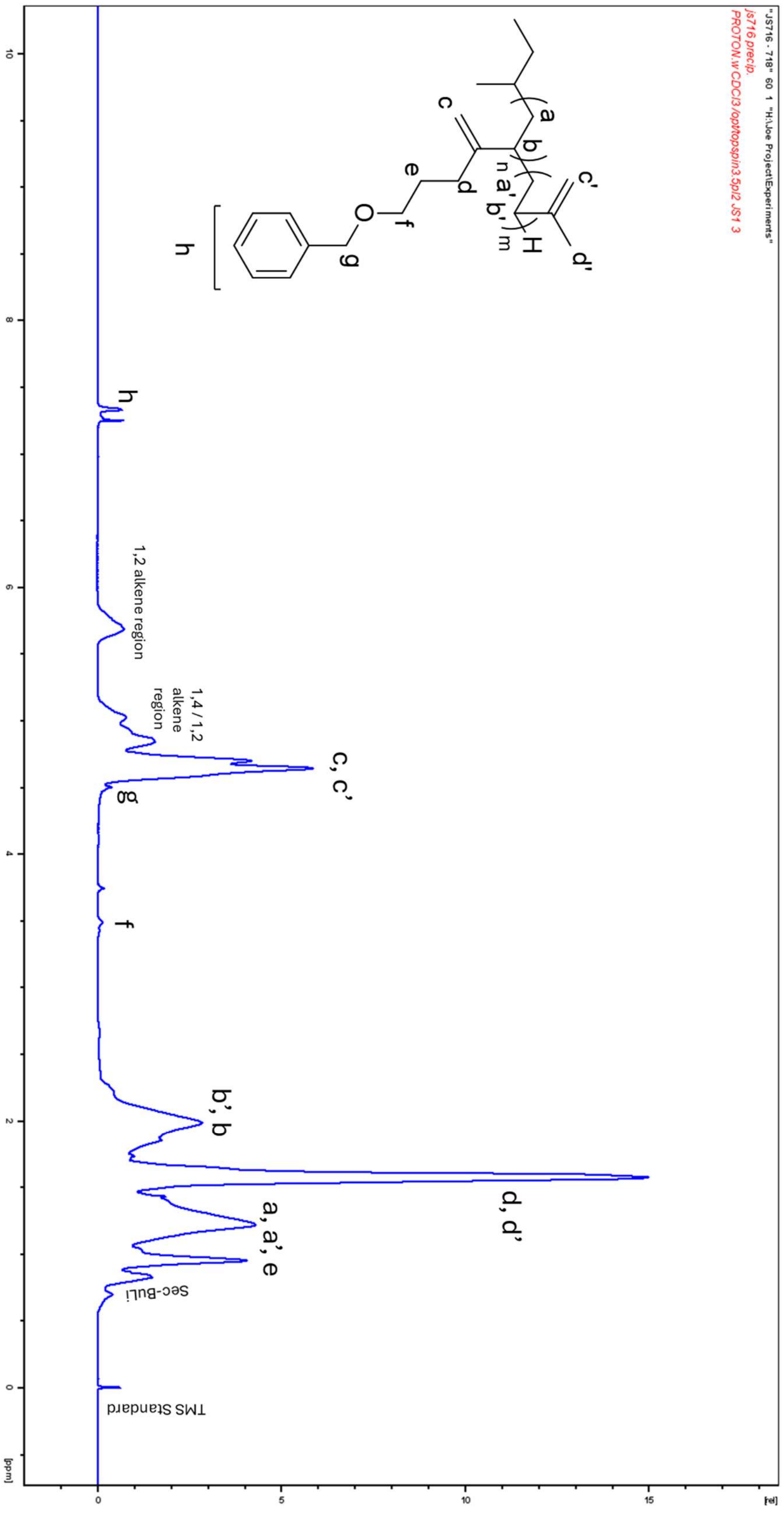

- *Figure S49: ^1H NMR **(400 MHz, CDCl$_3$): Copol 1***

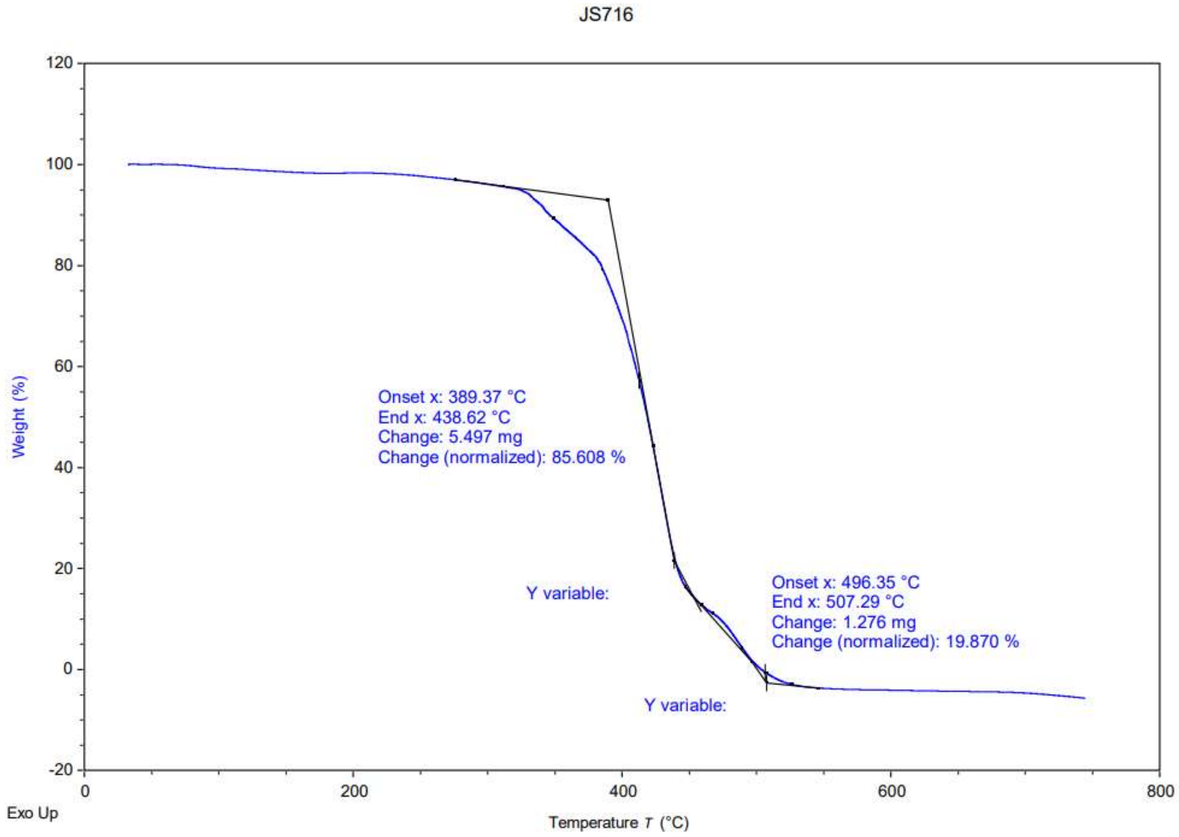

Figure S50: TGA analysis of Copol 1

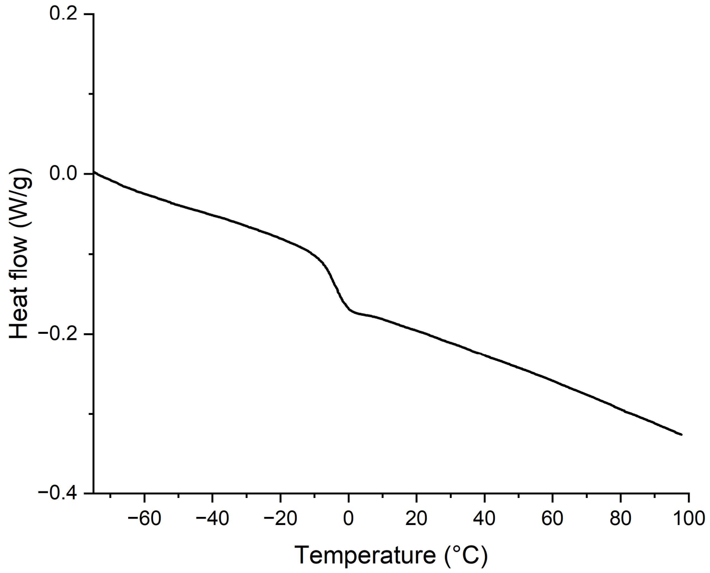

Figure S51: TGA Copol 1

Copol 2

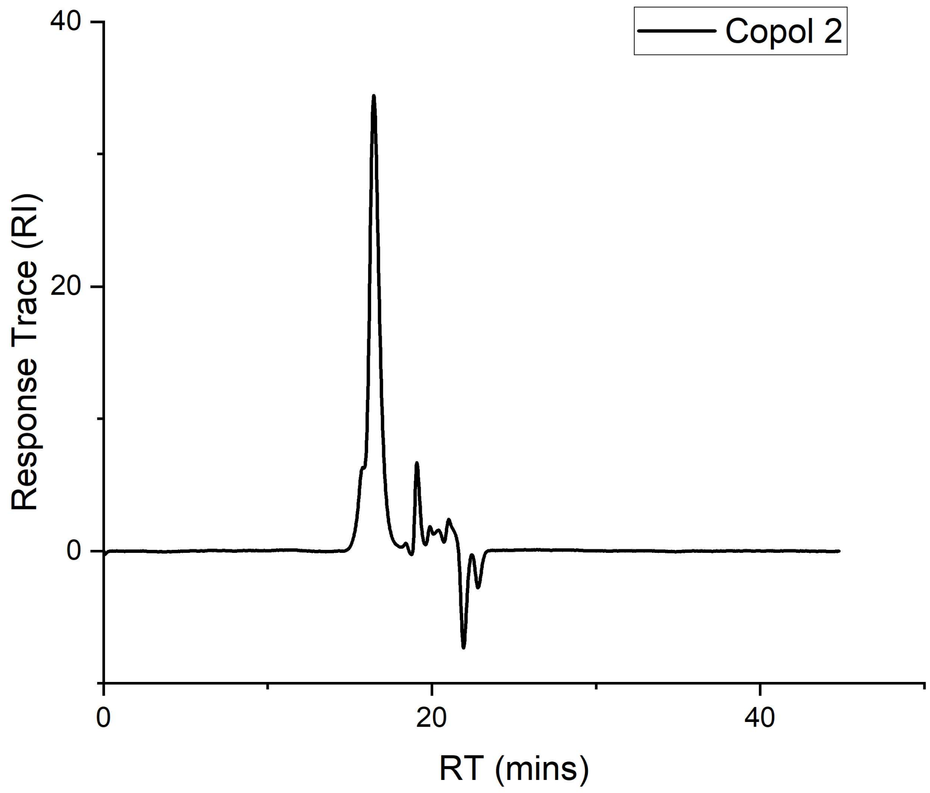

Figure S52: GPC analysis trace of Copol 2

Table S12: MWt values from the GPC analysis of Copol 2

MW Averages							
Peaks	Mp (g/mol)	Mn (g/mol)	Mw (g/mol)	Mz (g/mol)	Mz+1 (g/mol)	Mv (g/mol)	**Đ**
Peak 1	4520	4060	5110	6540	8550	4930	1.26

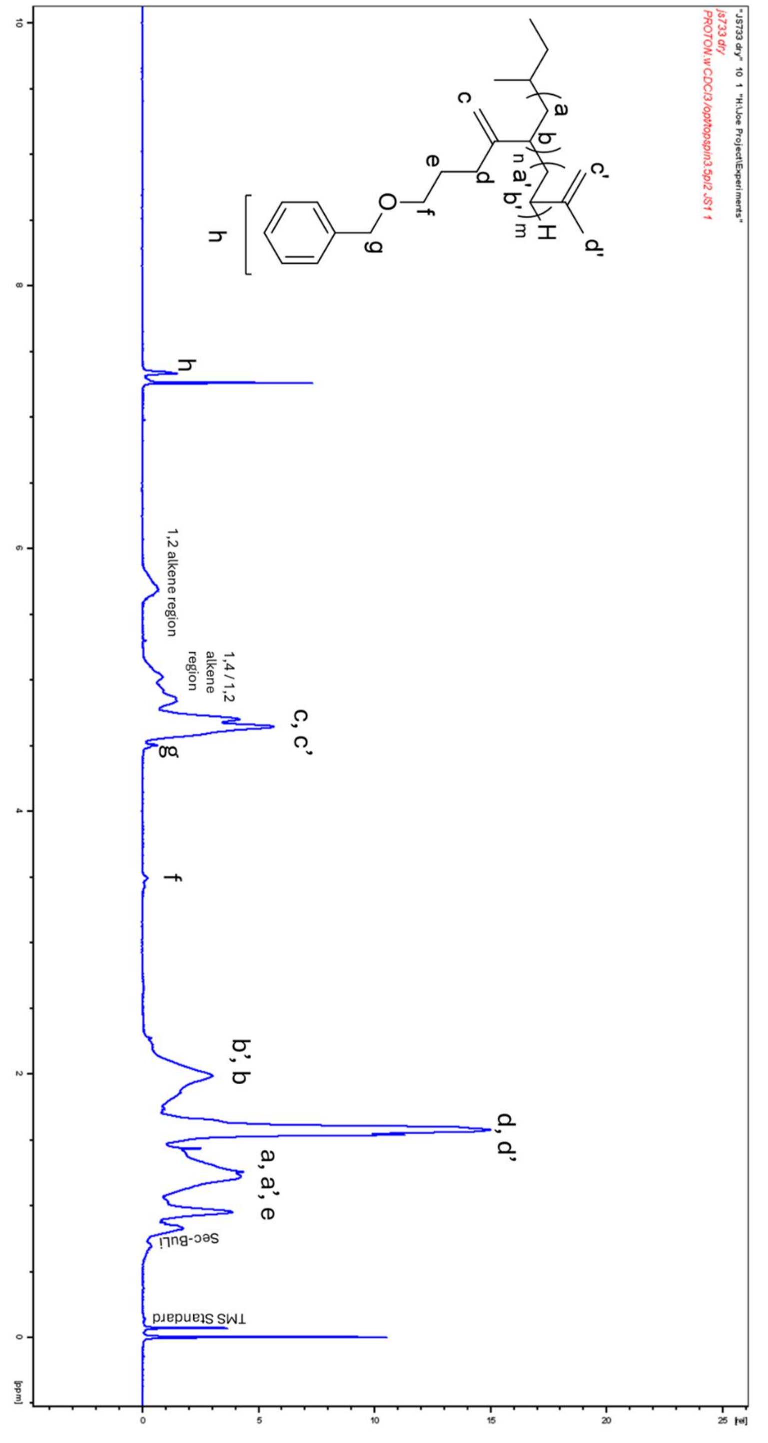

- *Figure S53: ^{1}H NMR **(400 MHz, $CDCl_3$): Copol 2***

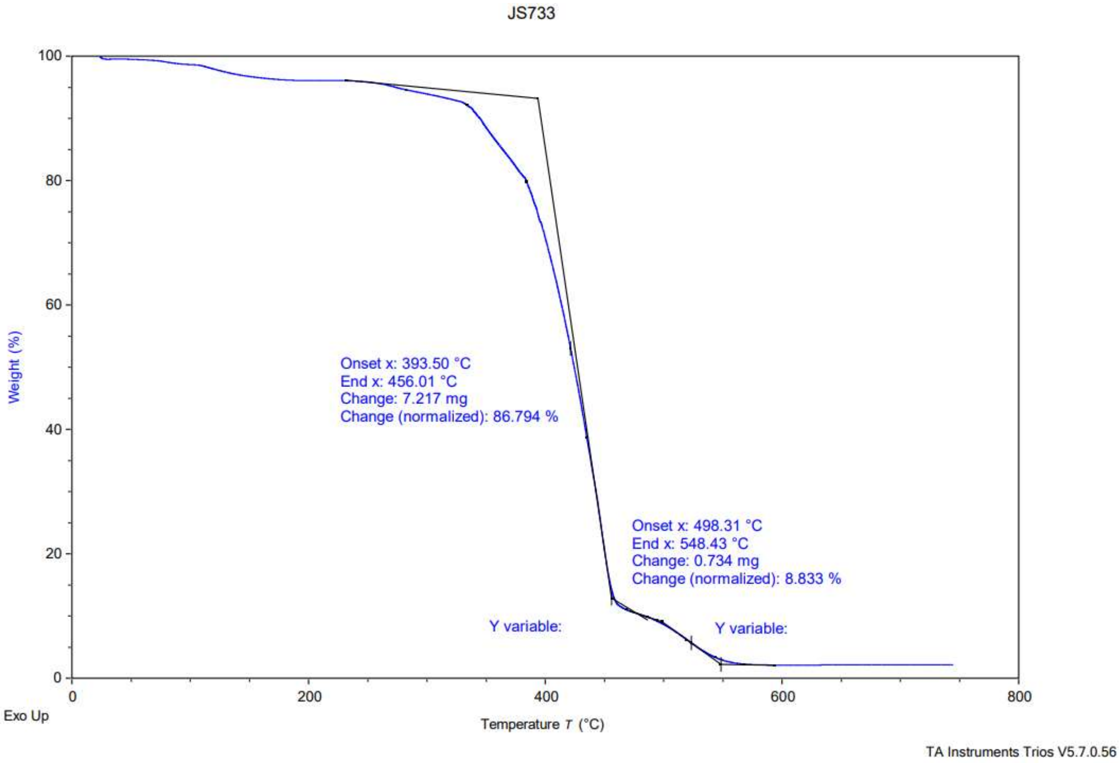

Figure S54: TGA Analysis of Copol 2

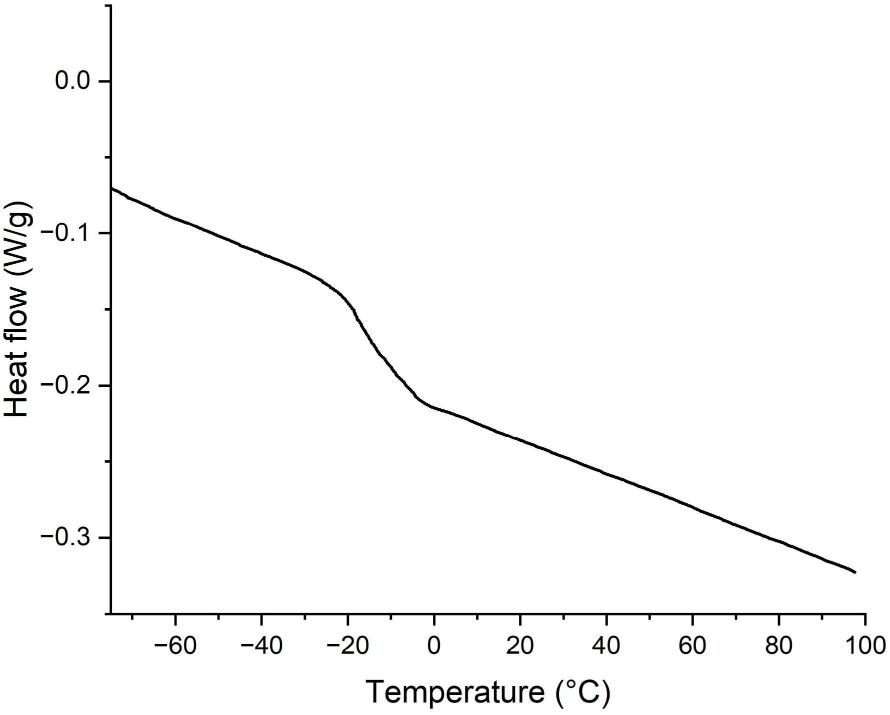

Figure S55: DSC (2nd Heating cycle) of Copol 2

Copol 3

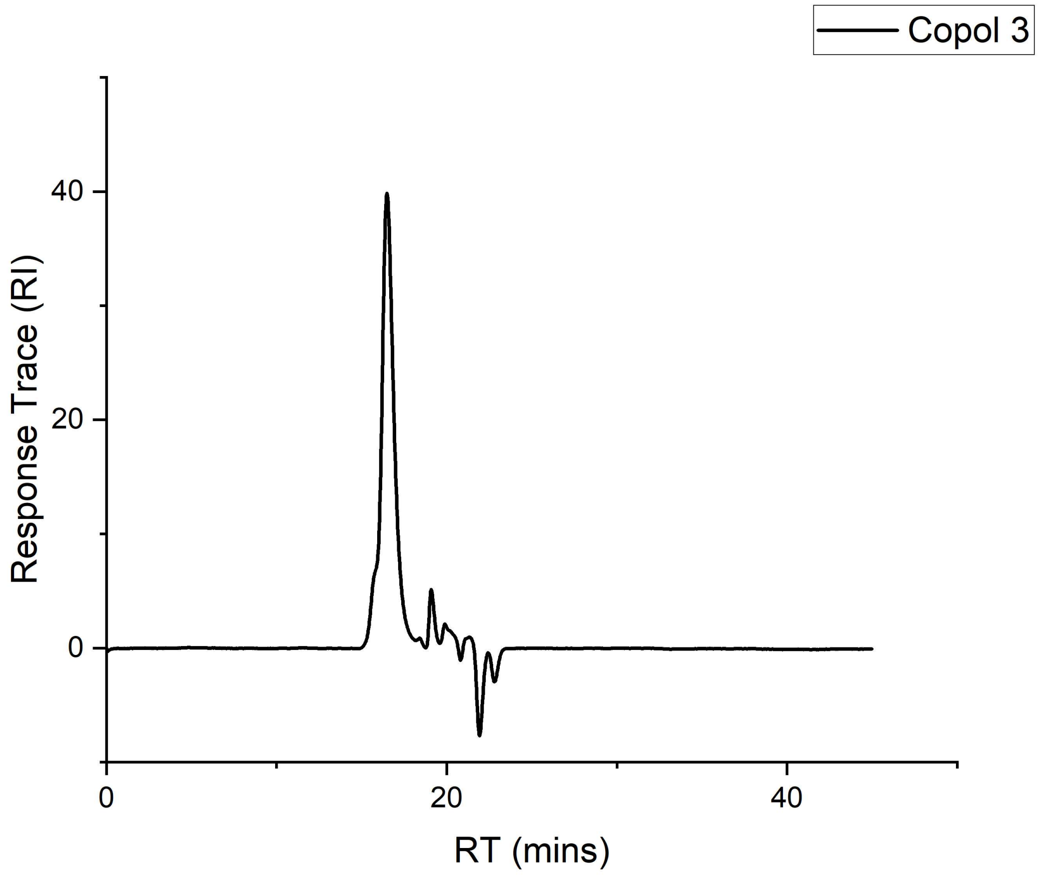

Figure S56: GPC Trace of Copol 3

Table S13: MWt values obtained from the GPC analysis of Copol 3

MW Averages							
Peaks	Mp (g/mol)	Mn (g/mol)	Mw (g/mol)	Mz (g/mol)	Mz+1 (g/mol)	Mv (g/mol)	**Đ**
Peak 1	4440	3660	4700	5970	7620	4530	1.28

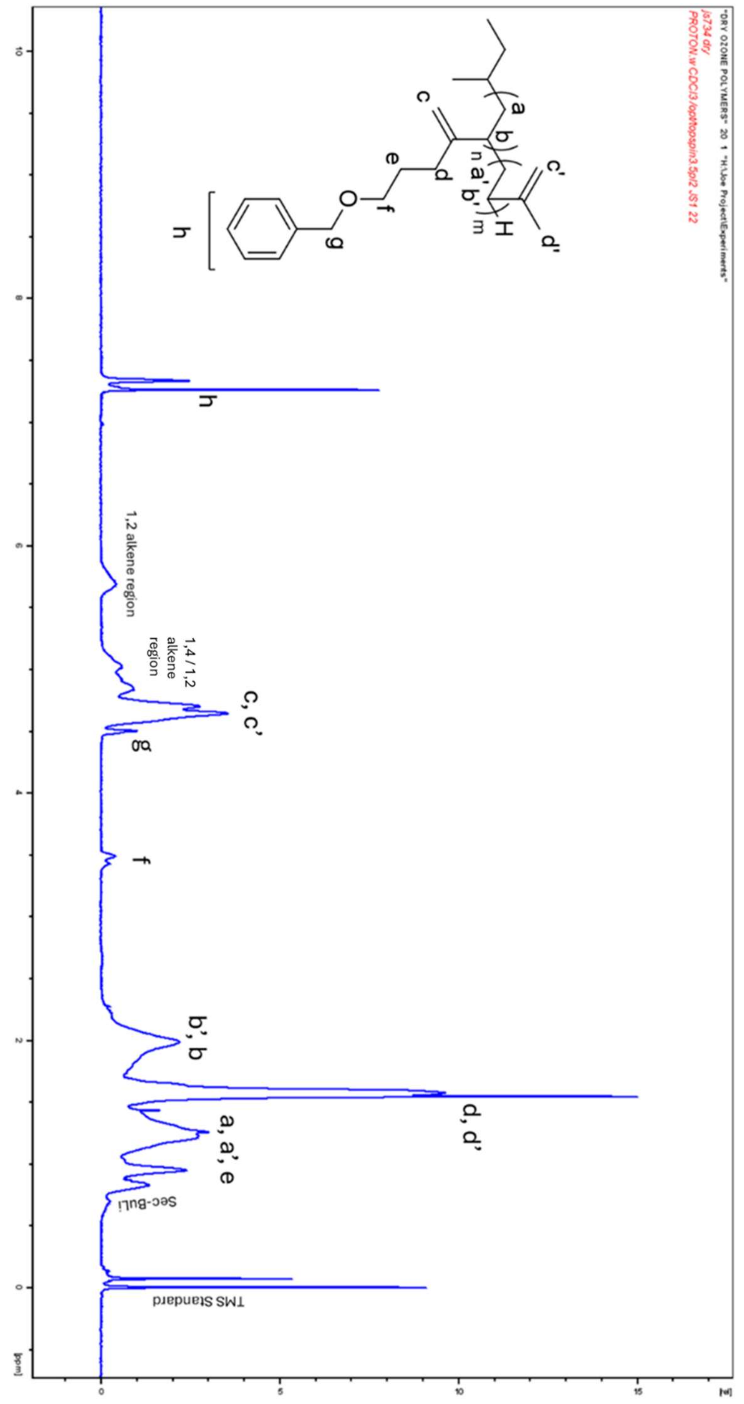

- *Figure S57: ¹H NMR* ***(400 MHz, CDCl₃): Copol 3***

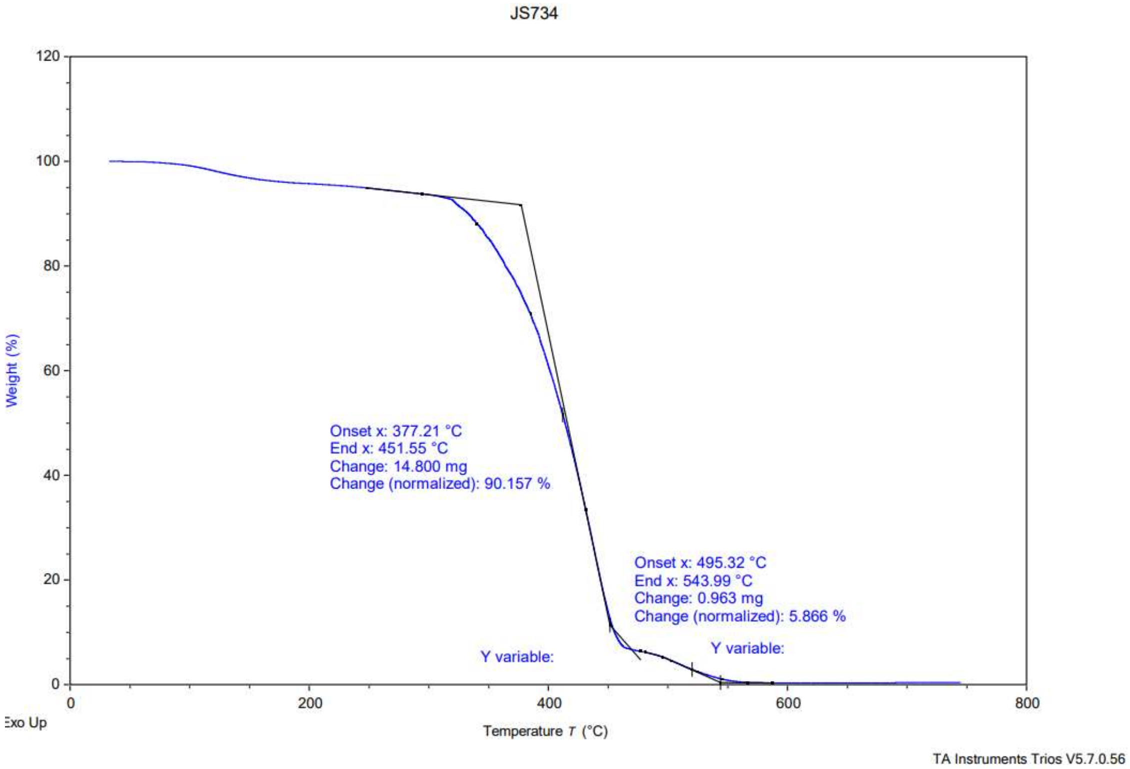

Figure S58: TGA Analysis of Copol 3

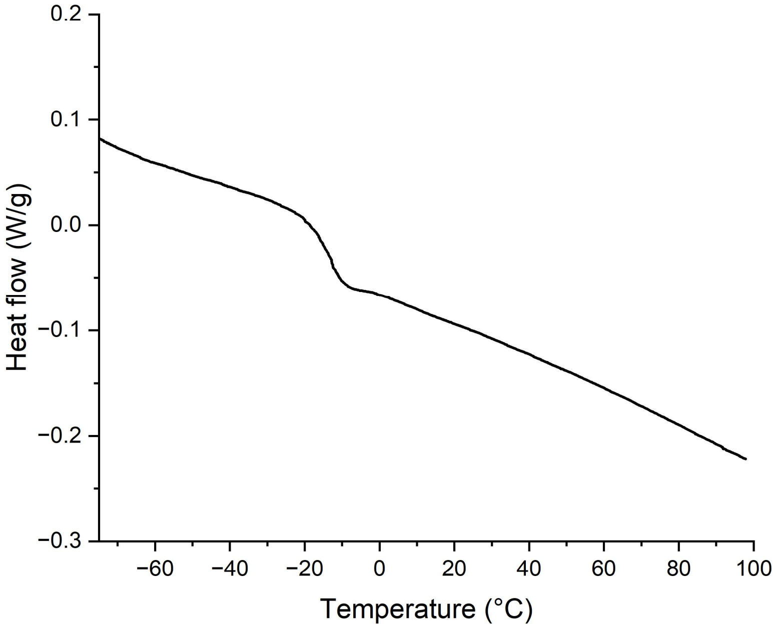

Figure S59: DSC (2nd Heating cycle) of Copol 3

Copol 4

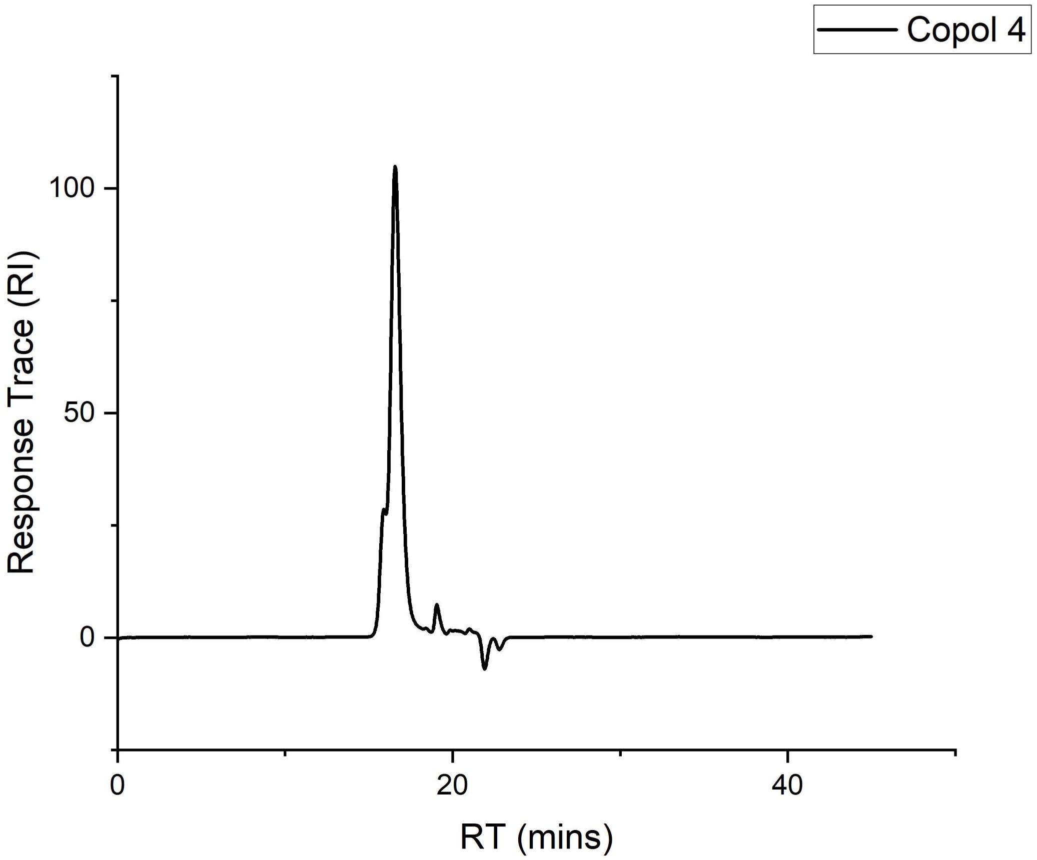

Figure S60: GPC Trace of Copol 4

Table S14: MWt values obtained from GPC analysis of Copol 4

MW Averages							
Peaks	Mp (g/mol)	Mn (g/mol)	Mw (g/mol)	Mz (g/mol)	Mz+1 (g/mol)	Mv (g/mol)	**Đ**
Peak 1	4050	3750	4500	5390	6350	4380	1.20

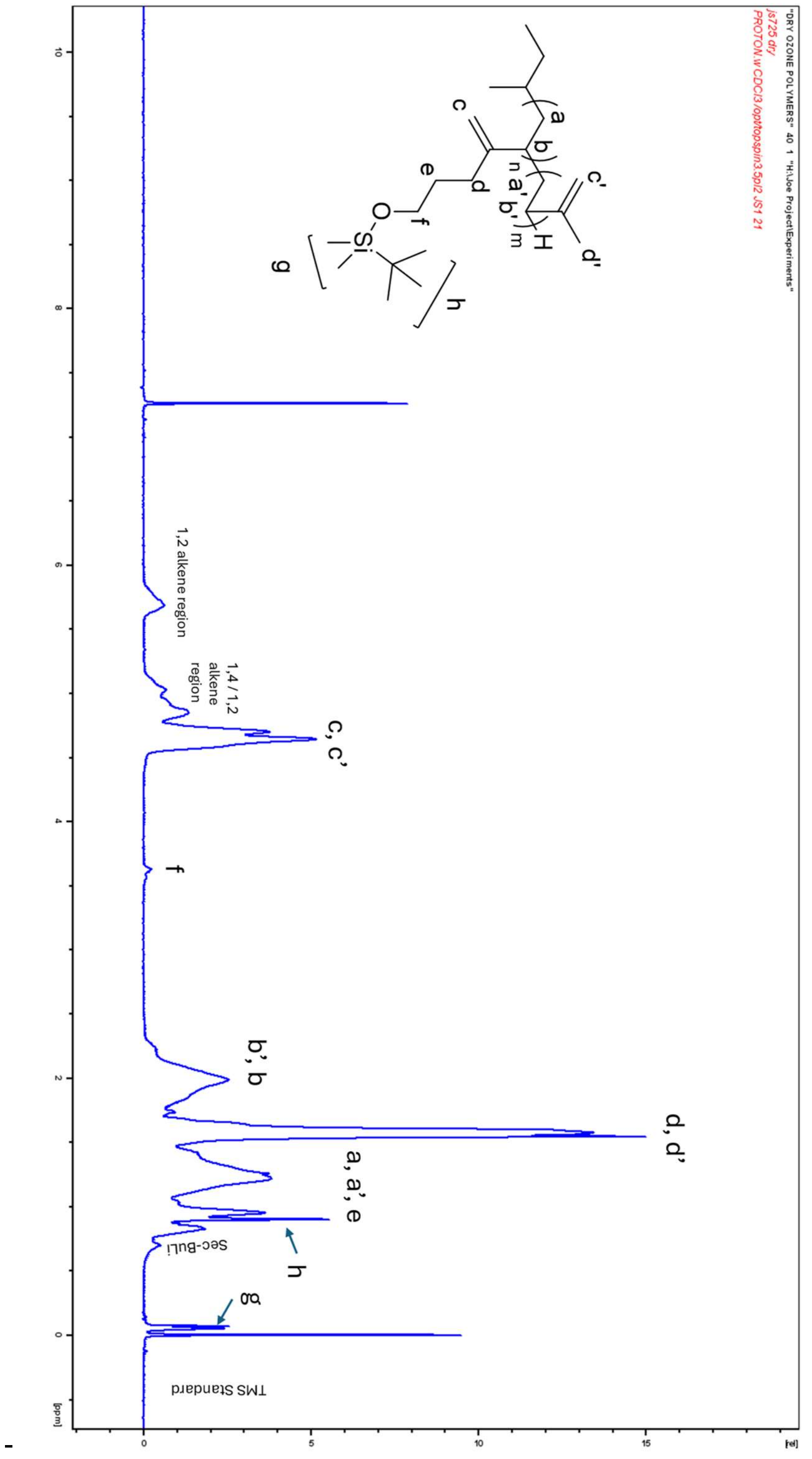

- *Figure S61: **^{1}H NMR (400 MHz, $CDCl_3$): Copol 4***

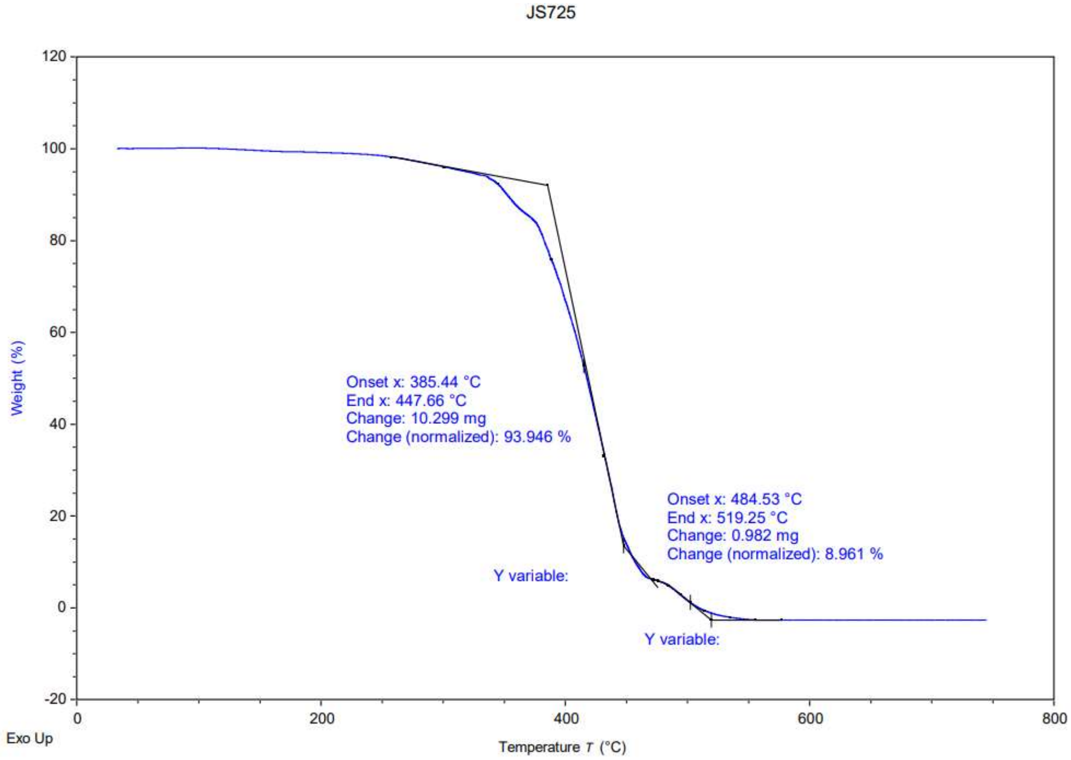

Figure S62: TGA analysis of Copol 4

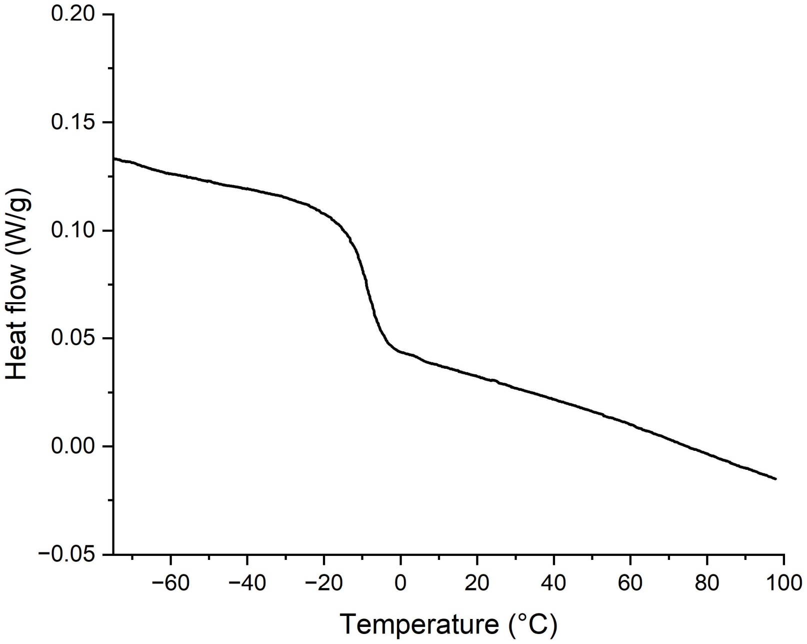

Figure S63: DSC (2nd Heating cycle) Copol 4

Copol 5

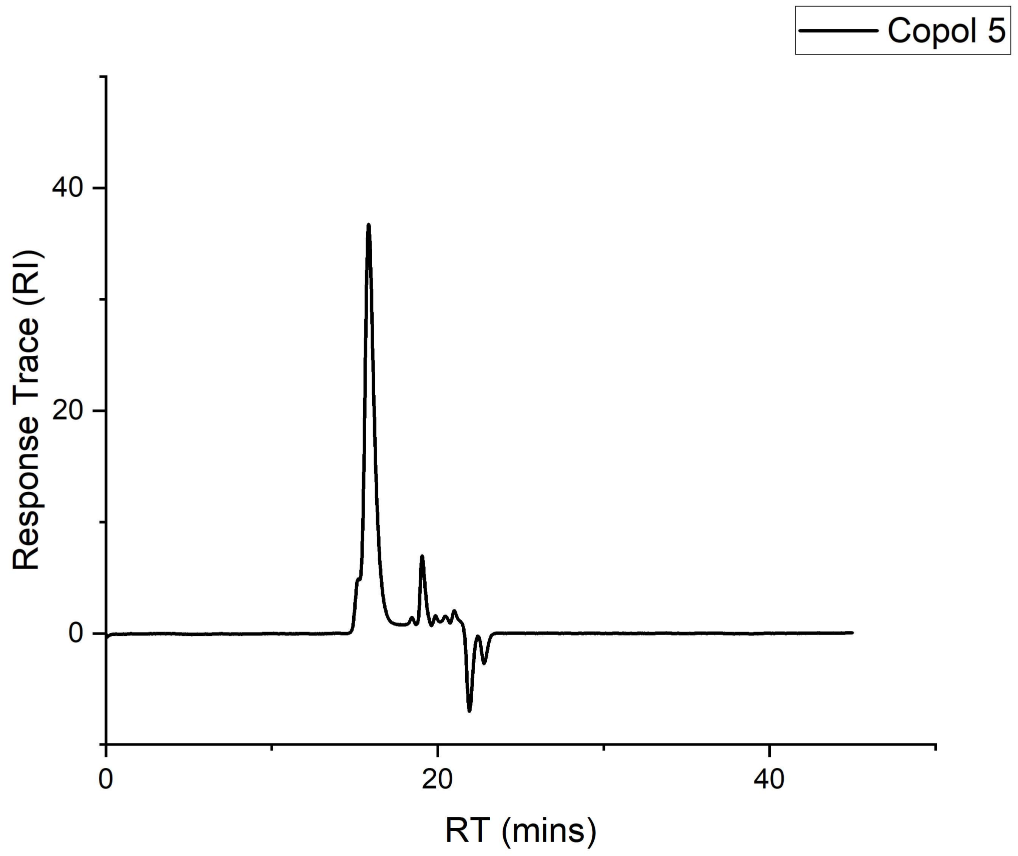

Figure S64: GPC trace of Copol 5

Table S15: MWt values obtained from the GPC analysis of Copol 5

MW Averages							
Peaks	Mp (g/mol)	Mn (g/mol)	Mw (g/mol)	Mz (g/mol)	Mz+1 (g/mol)	Mv (g/mol)	**Đ**
Peak 1	8990	7130	8840	10400	12200	8600	1.24

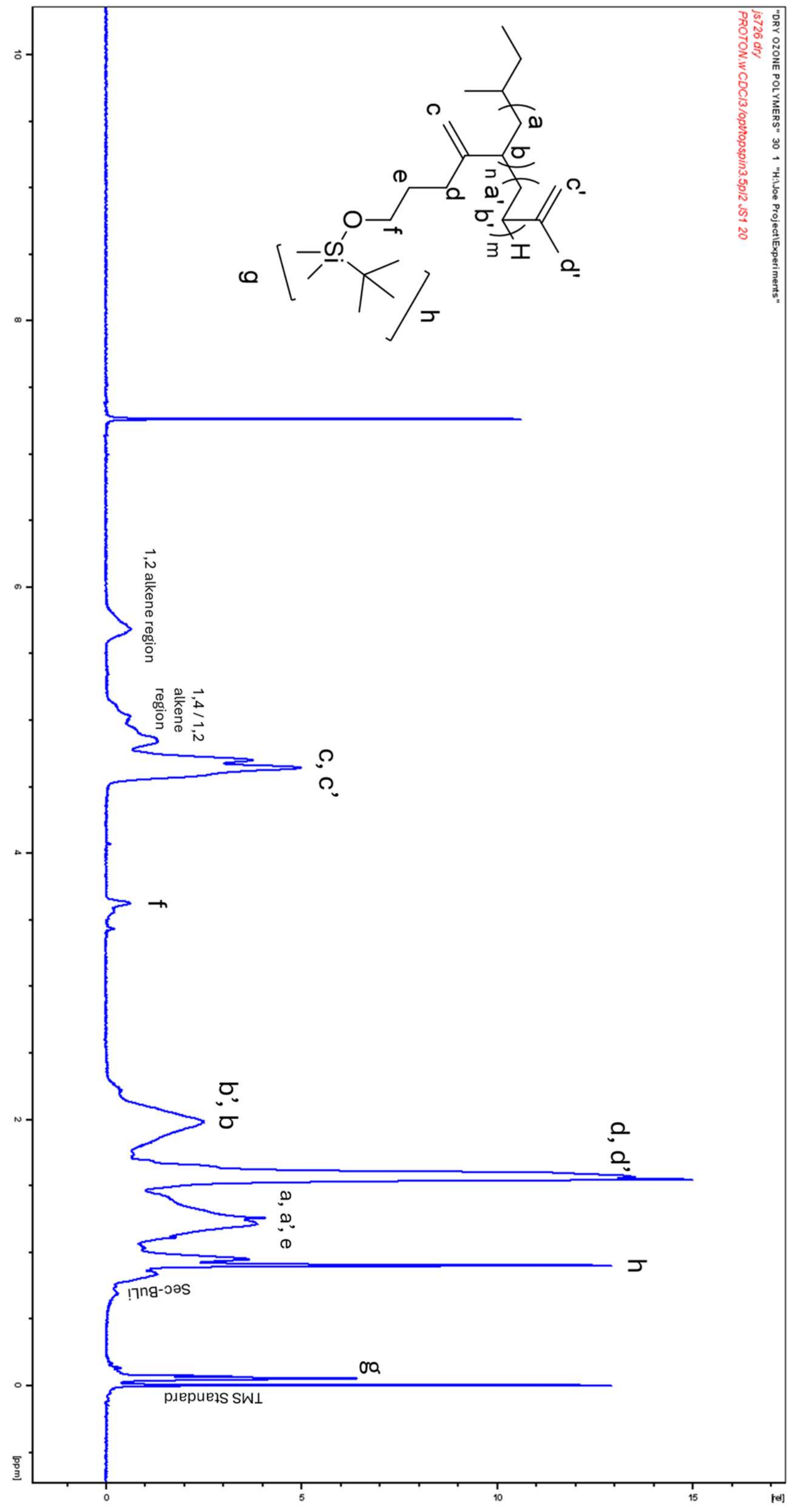

- *Figure S65:* ^{1}*H NMR* ***(400 MHz,*** $CDCl_3$***): Copol 5***

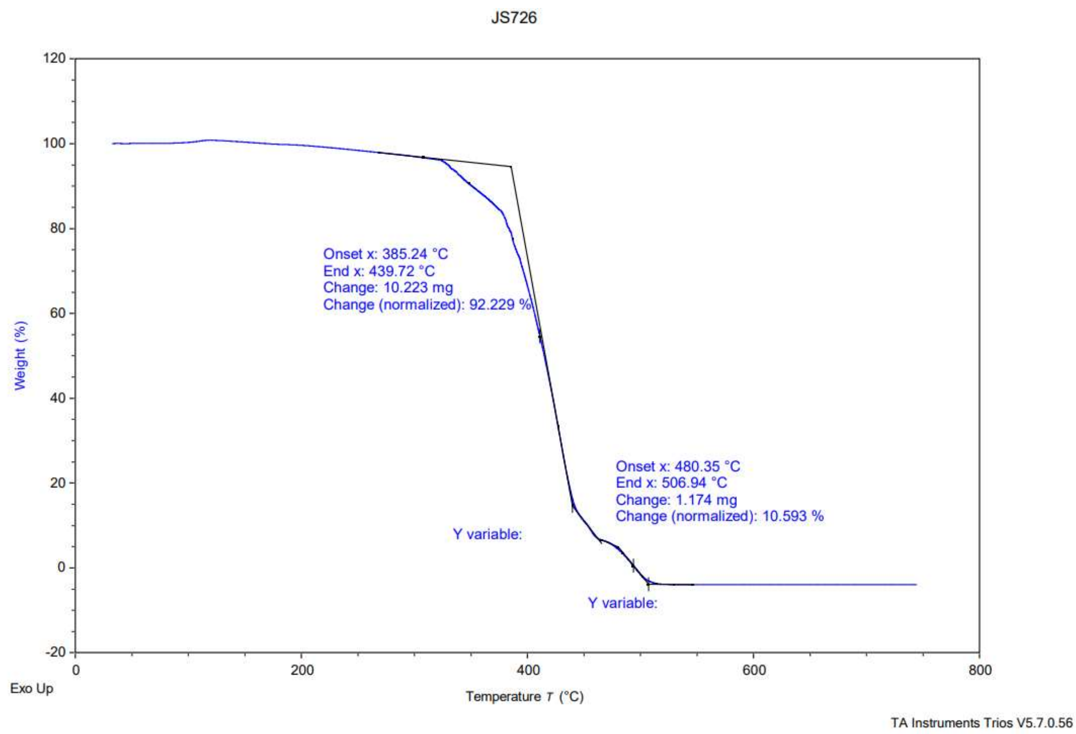

-

Figure S66: TGA Analysis of Copol 5

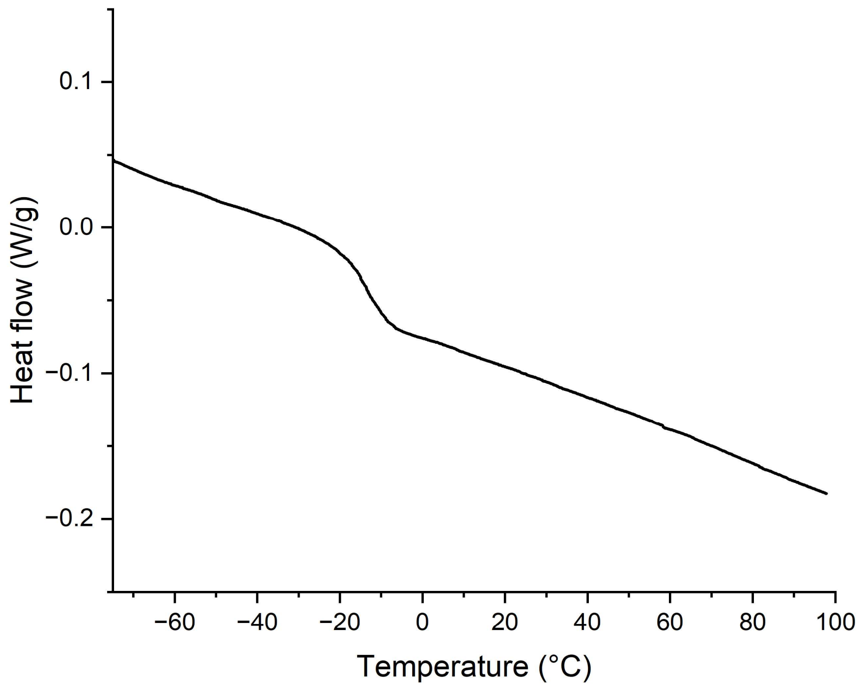

-

Figure S67: DSC (2nd Heating cycle) of Copol 5

Copol 6

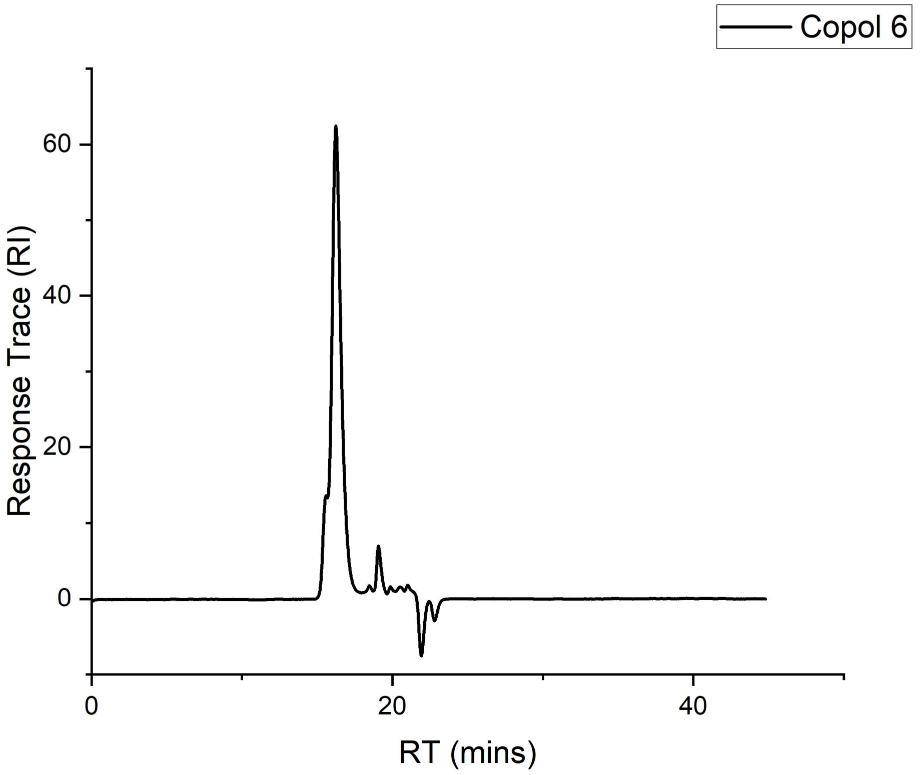

-

Figure S68: GPC trace of Copol 6

Table S16: MWt values obtained from GPC analysis of Copol 6

MW Averages							
Peaks	Mp (g/mol)	Mn (g/mol)	Mw (g/mol)	Mz (g/mol)	Mz+1 (g/mol)	Mv (g/mol)	**Đ**
Peak 1	5840	4910	6220	7590	9150	6030	1.27

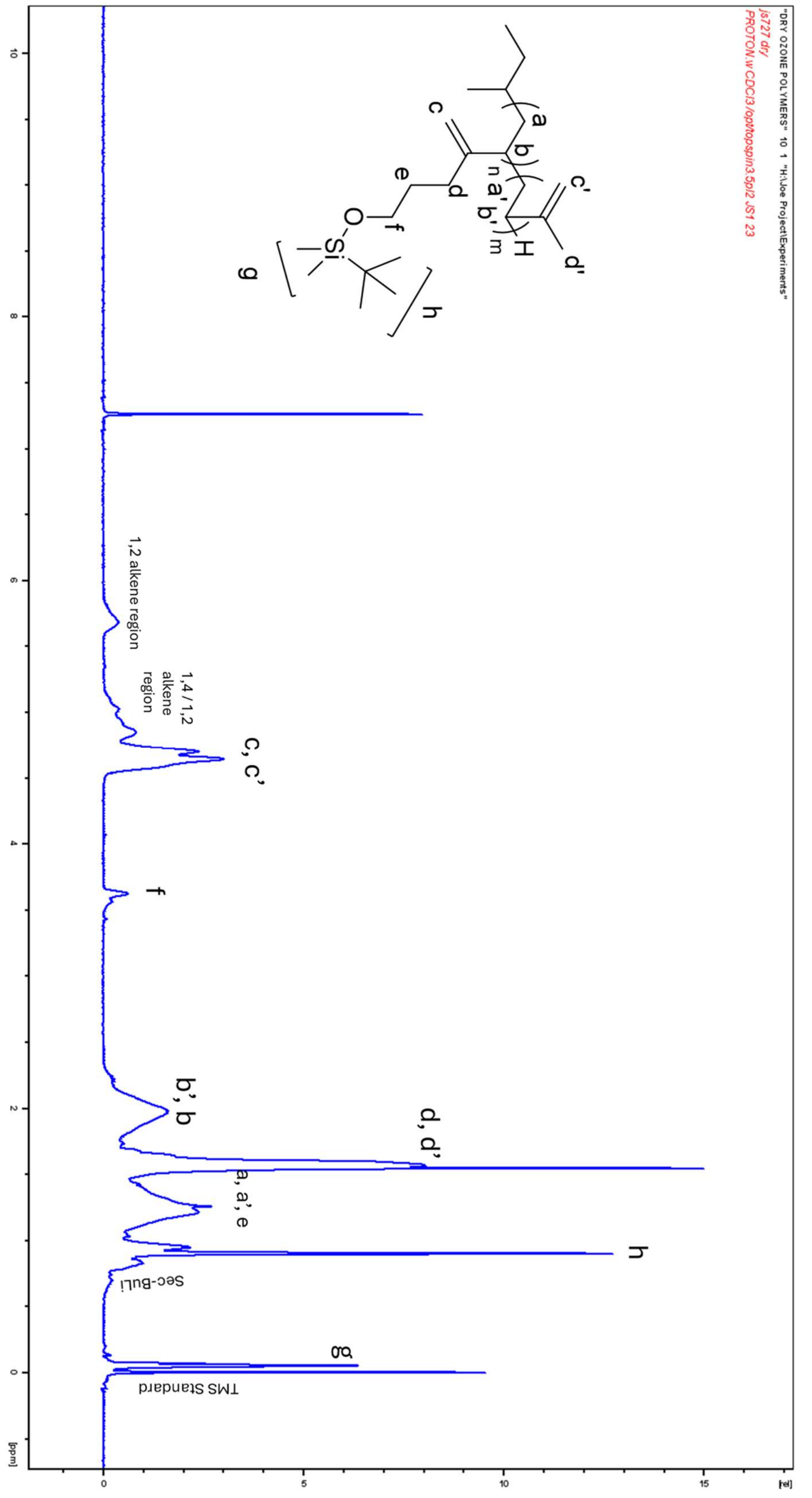

-

- *Figure S69: ^{1}H NMR* ***(400 MHz, $CDCl_3$): Copol 6***

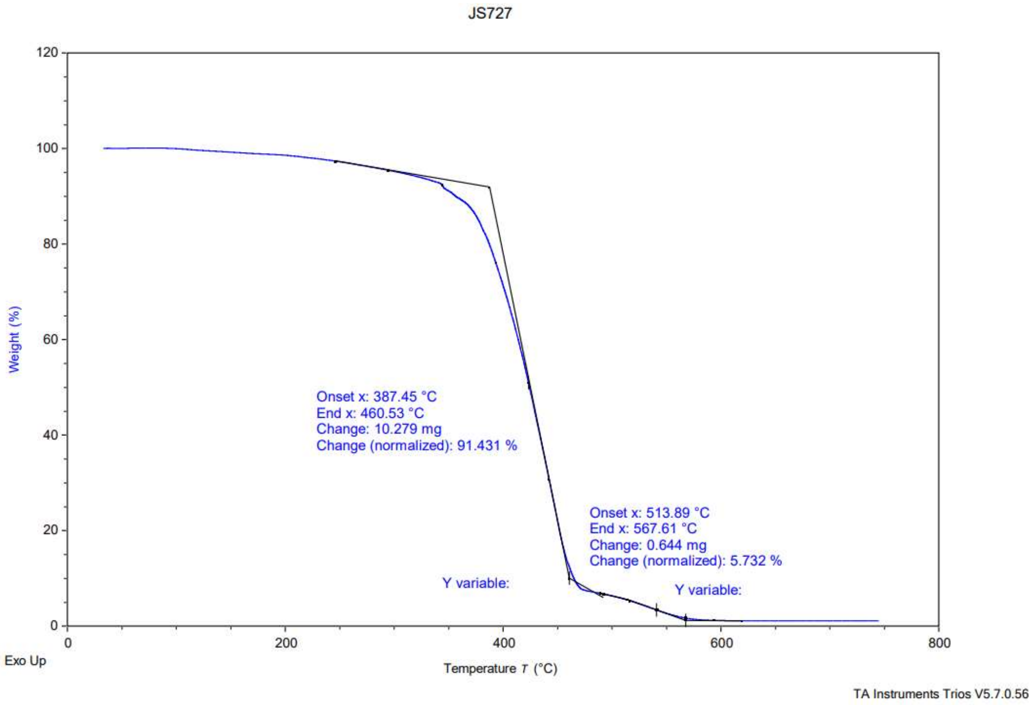

Figure S70: TGA of Copol 6

-

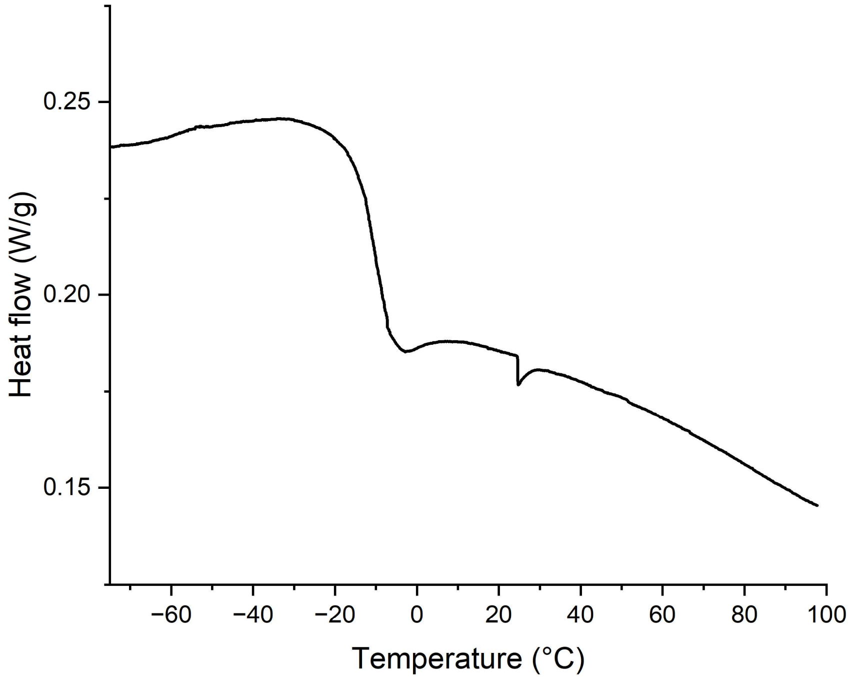

Figure S71: DSC (2nd Heating cycle) of Copol 6

5 Deprotected Copolymer Data

Hydrogenated Copol 3

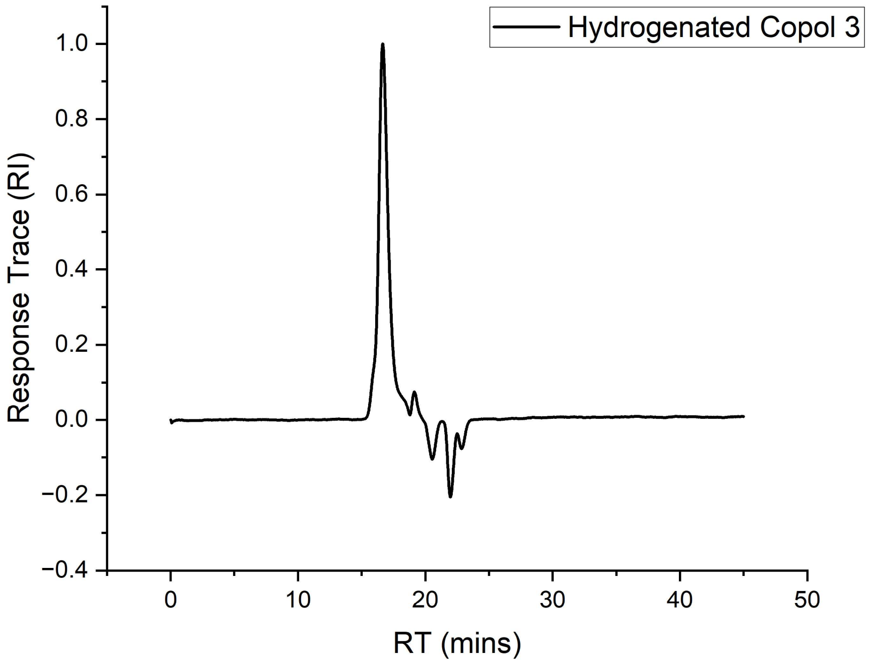

-

Figure S72: Hydrogenated Copol 3 GPC Trace

Table S17: MWt averages obtained from GPC analysis of Hydrogenated Copol 3

MW Averages							
Peaks	Mp (g/mol)	Mn (g/mol)	Mw (g/mol)	Mz (g/mol)	Mz+1 (g/mol)	Mv (g/mol)	**Đ**
Peak 1	3460	2500	3420	4320	5380	3290	1.37

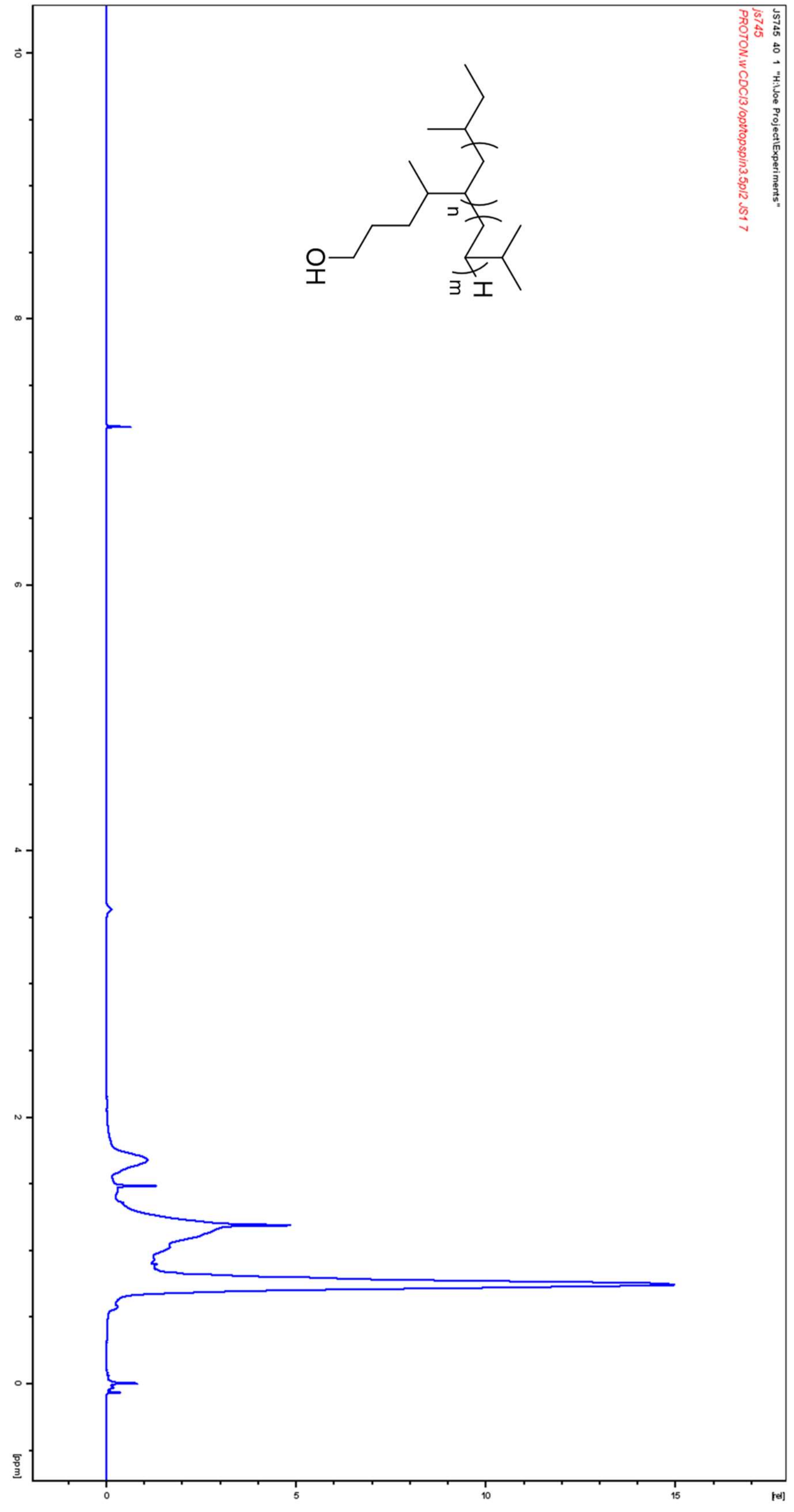

Figure S73: ^{1}H NMR ***(400 MHz, $CDCl_3$): Hydrogenated Copol 3***

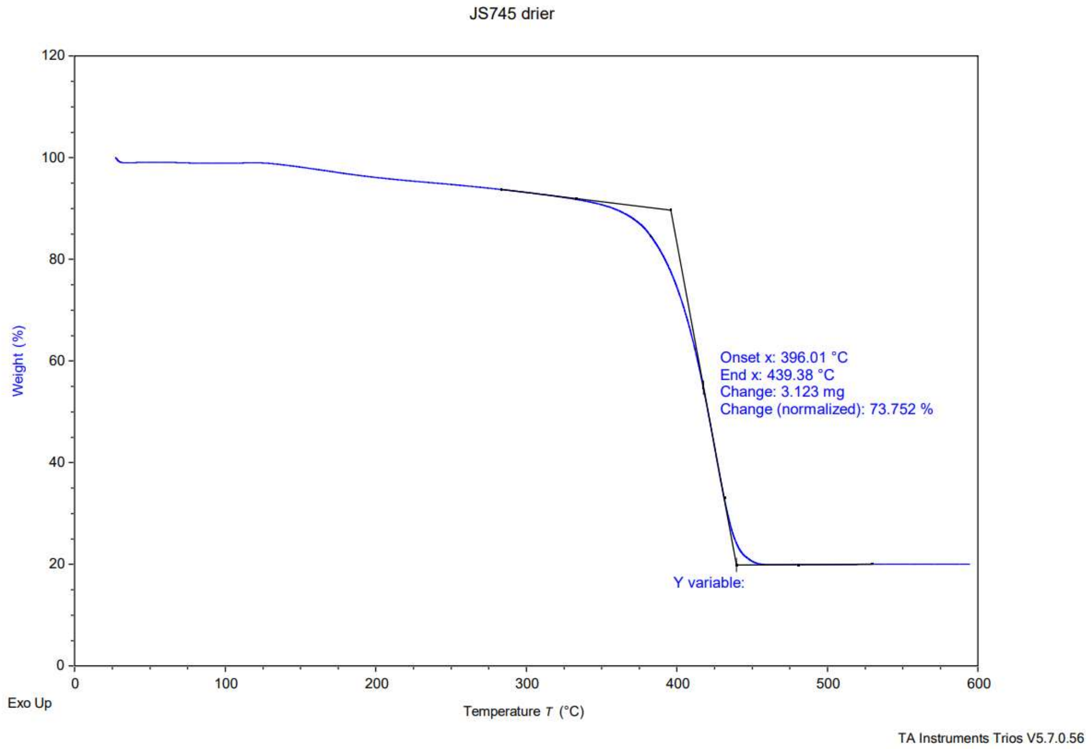

-

Figure S74: TGA Analysis of Hydrogenated Copol 3

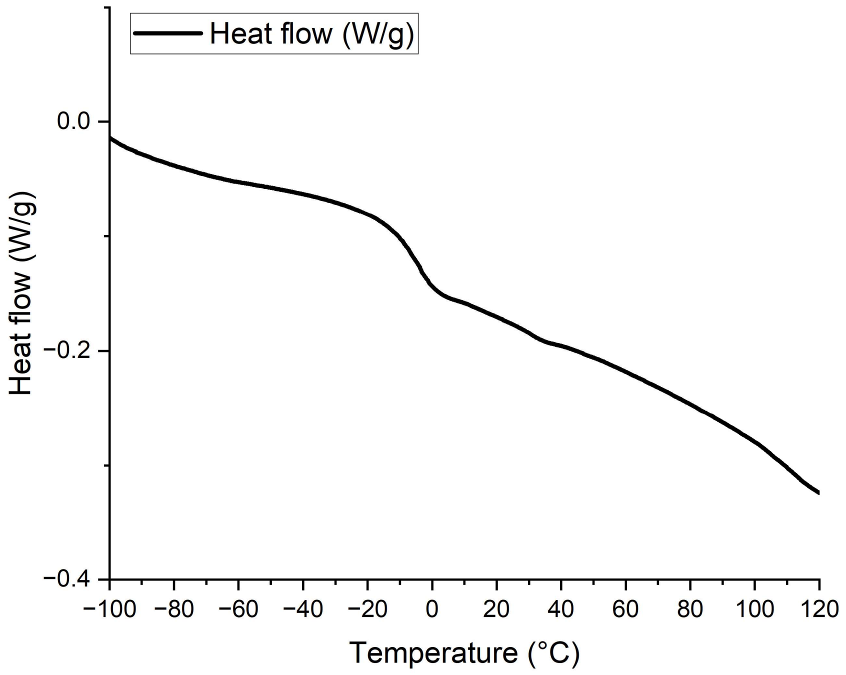

-

Figure S75: DSC (2nd Heating cycle) of Hydrogenated Copol 3

6 TBAF TREATED COPOL 6

- GPC

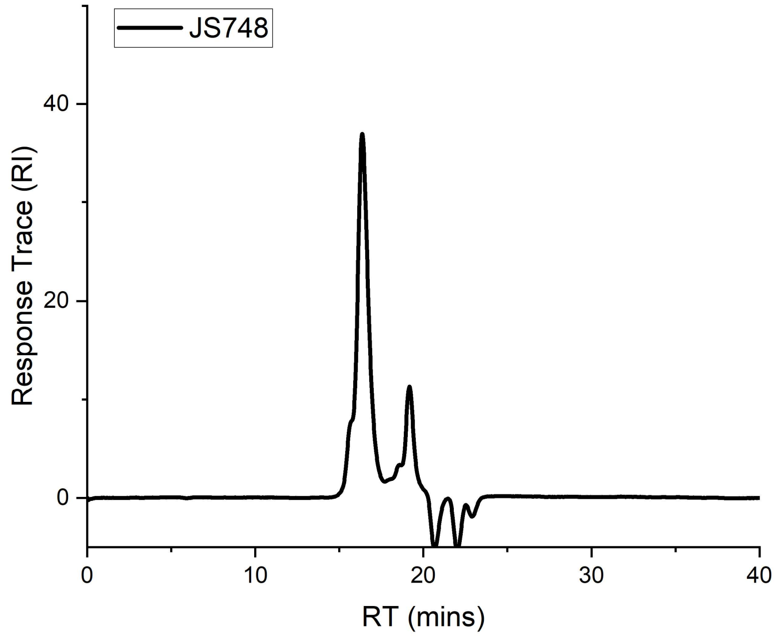

Figure S76: GPC Trace of TBAF Treated Copol 3

Table S18: MWt Avergaes obtained from the GPC analysis of TABF treated Copol 6.

MW Averages							
Peaks	Mp (g/mol)	Mn (g/mol)	Mw (g/mol)	Mz (g/mol)	Mz+1 (g/mol)	Mv (g/mol)	**Đ**
Peak 1	4700	3900	5110	6610	8680	4920	1.31

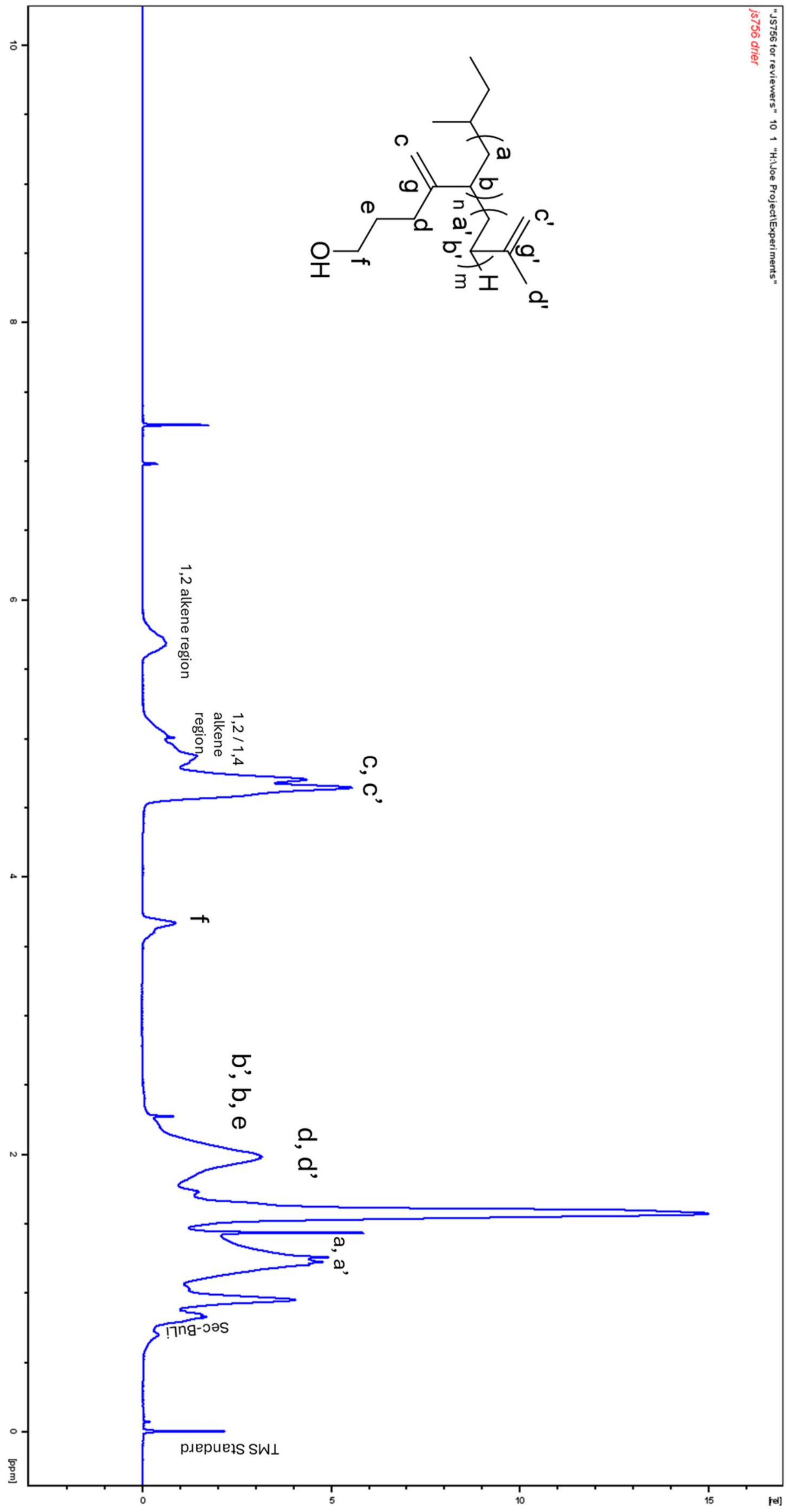

- *Figure S77: ^{1}H NMR **(400 MHz, CDCl$_3$): TBAF treated Copol 6***

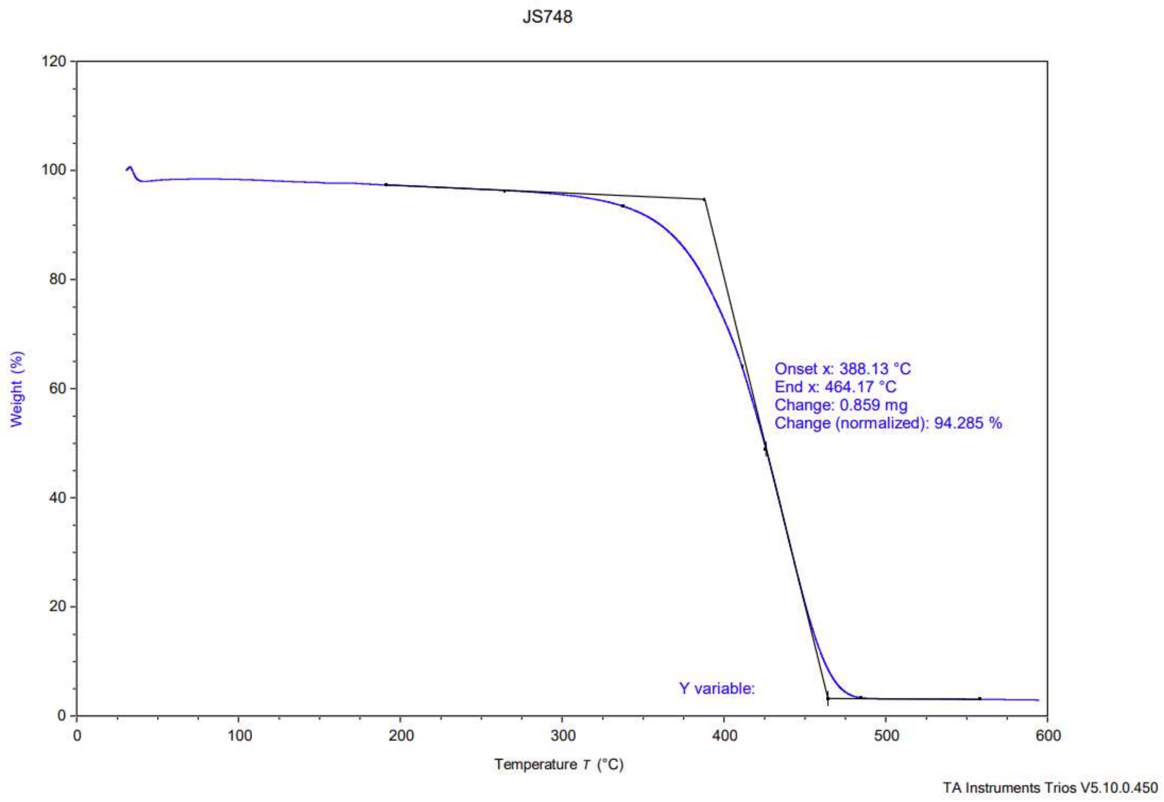

Figure S78: TGA analysis of TBAF treated Copol 6

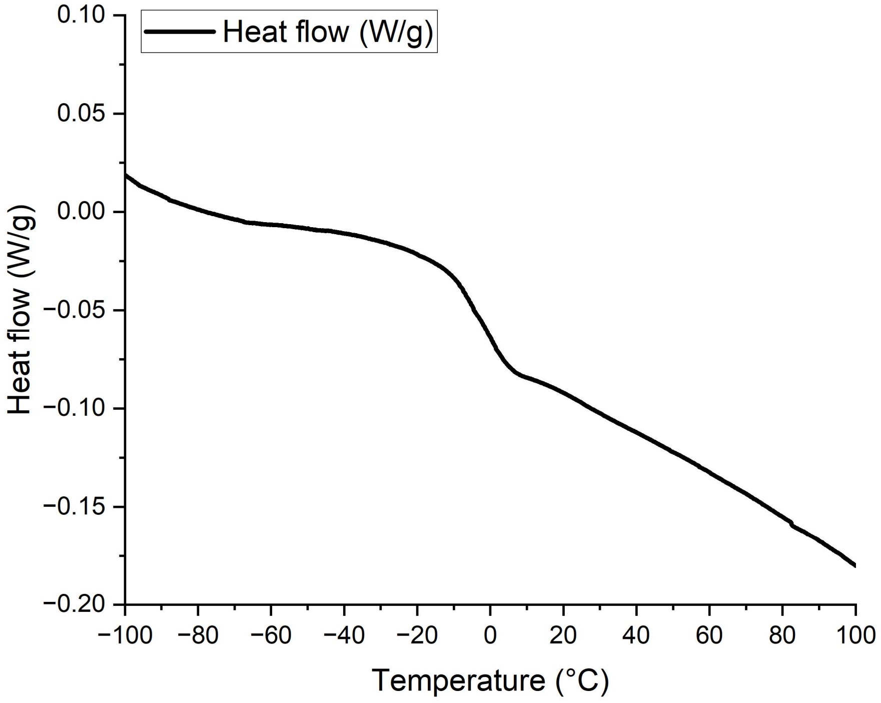

-

Figure S79: DSC (2nd Heating cycle) of TBAF Treated Copol 6

7 DOSY TRACKING EXPERIMENTS DATA

General comments:

Two sets of data are shown for each data point in the DOSY data below:

The first plot is a set of stacked ^{1}H NMR spectra, which is generated during a Pulse Gradient Spin Echo (PGSE) experiment. All species can be witnessed in the bottom spectra while only the slowest diffusing species (largest molecular weight, polymer chains) are seen in the top spectra. The highlighted regions show the peaks which were examined in the subsequent plot.

The second plots show the Stejskal-Tanner relation. Which relates the log of the integral decay (y axis) in the PGSE experiment to the gradient factor (x axis).

Some initial data points from the Stejskal-Tanner plot were omitted, due to significant solvent (cyclohexane) presence causing skewing of the data. Including these values would cause a di-exponential factor in the Stejskal-Tanner plot, rather than a mono-exponential. Hence giving inaccurate and inconsistent molecular weight estimations.

The diffusion constant associated with the *s*-BuLi ^{1}H NMR peak (0.8 ppm) was used to define polymer molecular weights according to calculations detailed in published literature.

Copolymer of 2 (Bn) and Isoprene (Low MWt)

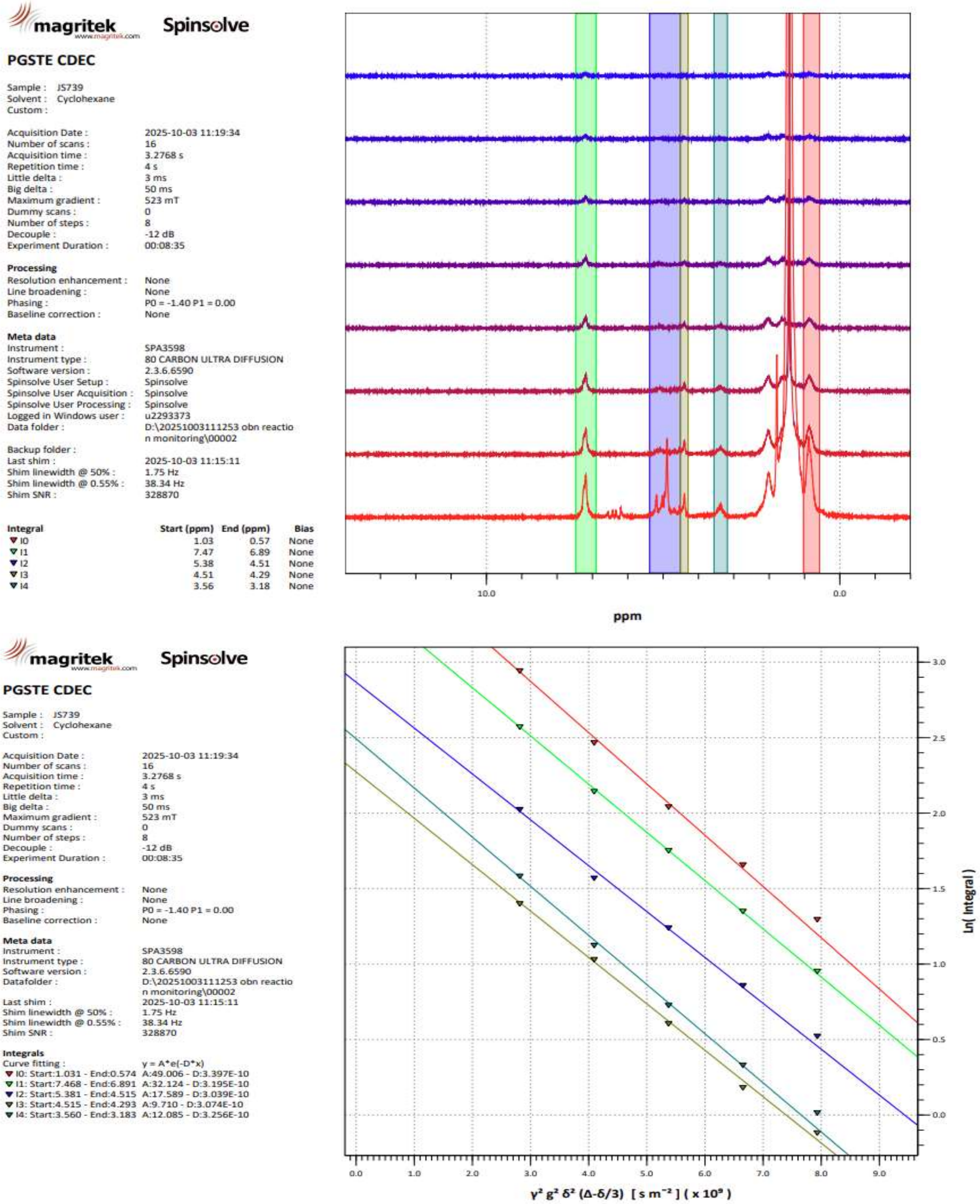

Figure S80: T = 30 minutes DOSY Spectrum with diffusion constants calculated from separate regions of the DOSY NMR through Stejskal-Tanner plot. Copolymer of 2 (Bn) and Isoprene (Low MWt)

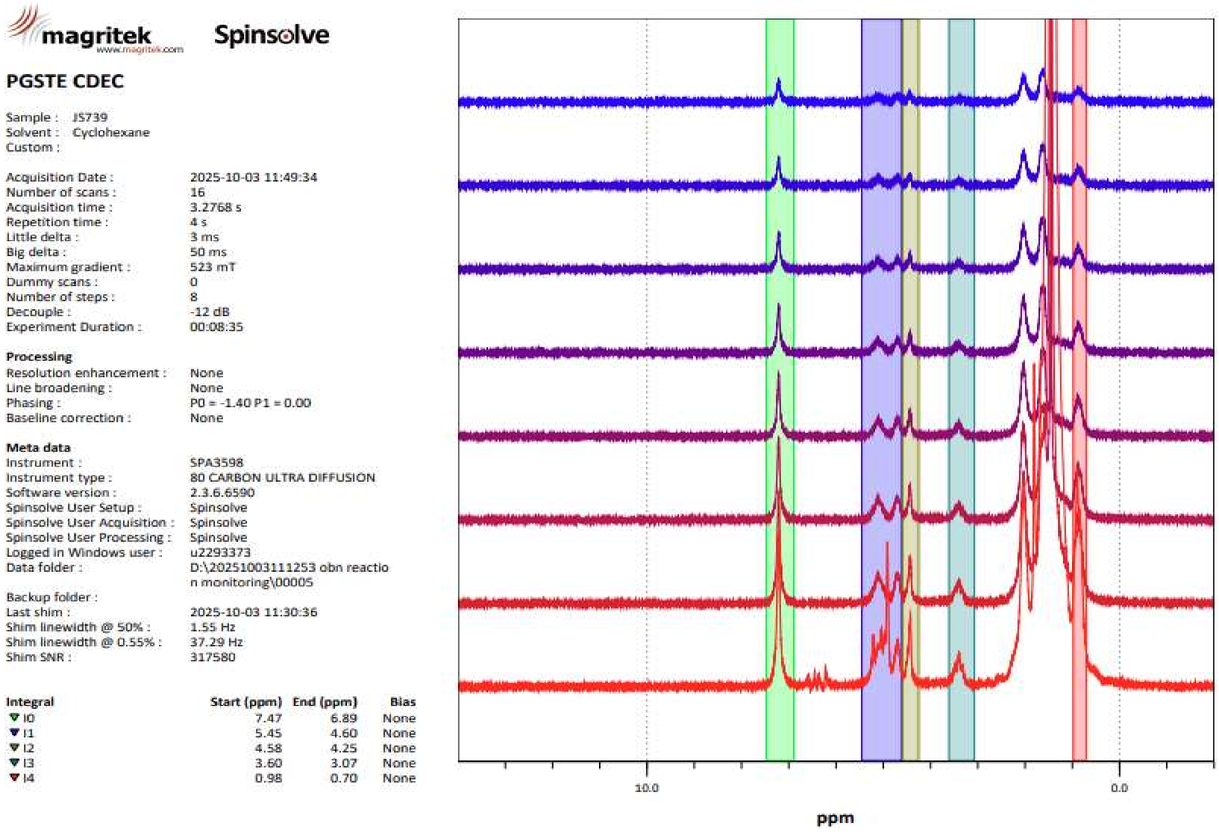

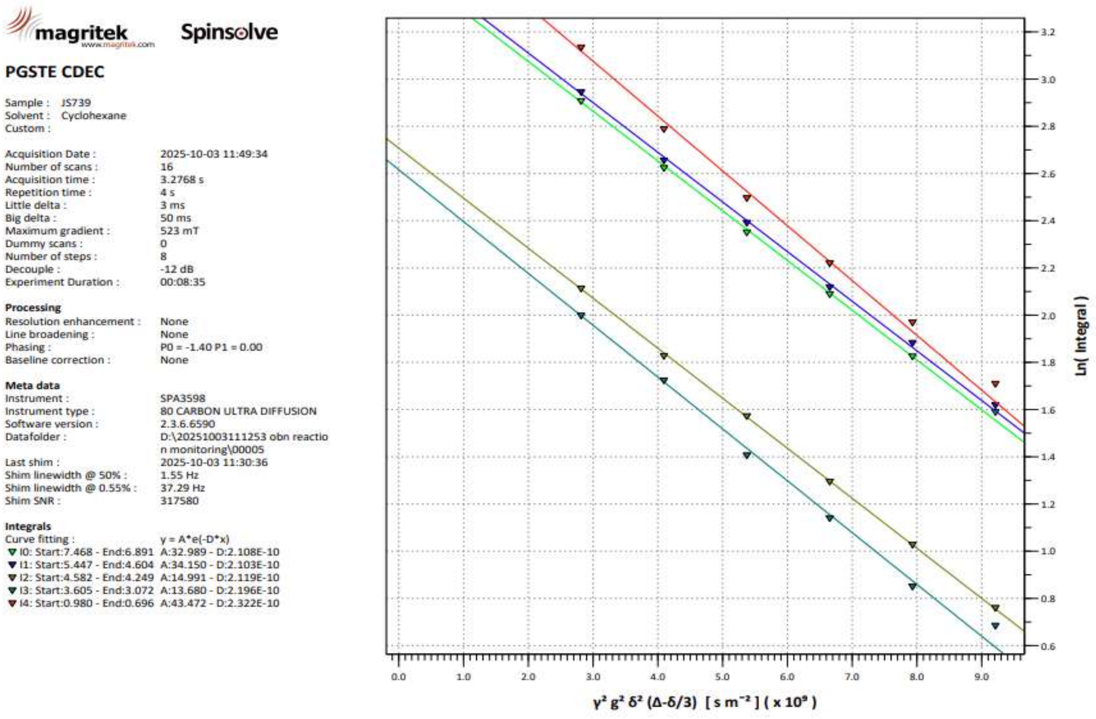

Figure S81: T = 1 hr DOSY Spectrum with diffusion constants calculated from separate regions of the DOSY NMR through Stejskal-Tanner plot. Copolymer of 2 (Bn) and Isoprene (Low MWt)

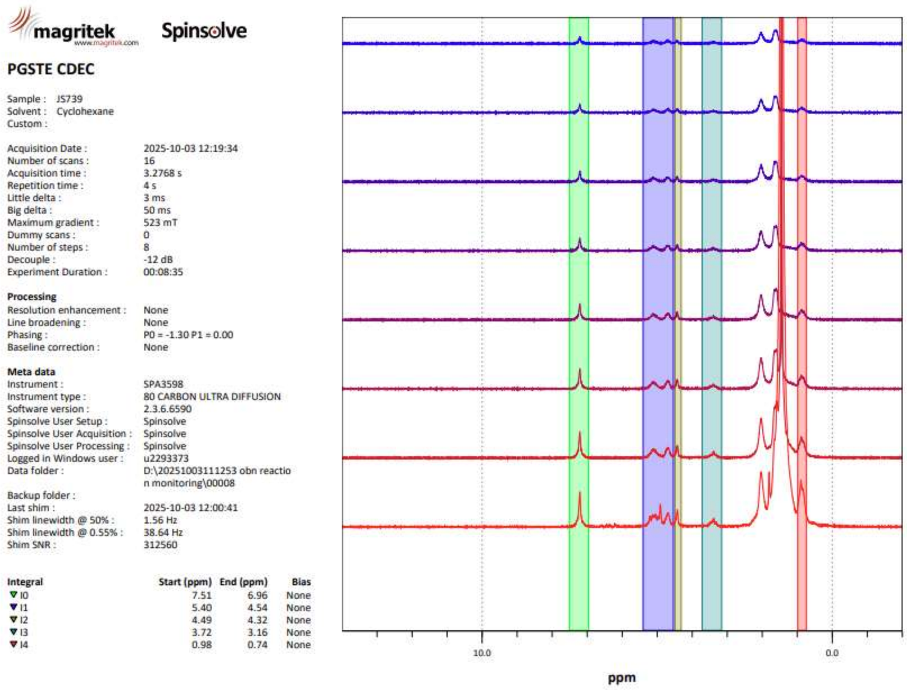

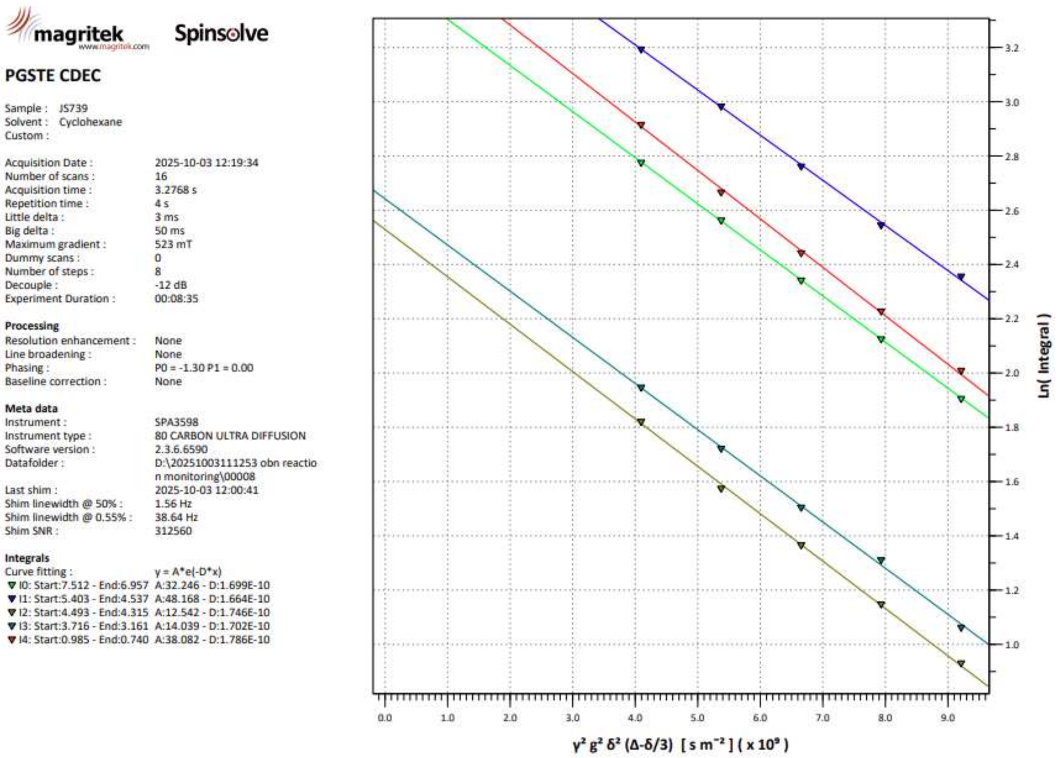

Figure S82: T = 1.5 hr DOSY Spectrum with diffusion constants calculated from separate regions of the DOSY NMR through Stejskal-Tanner plot. Copolymer of 2 (Bn) and Isoprene (Low MWt)

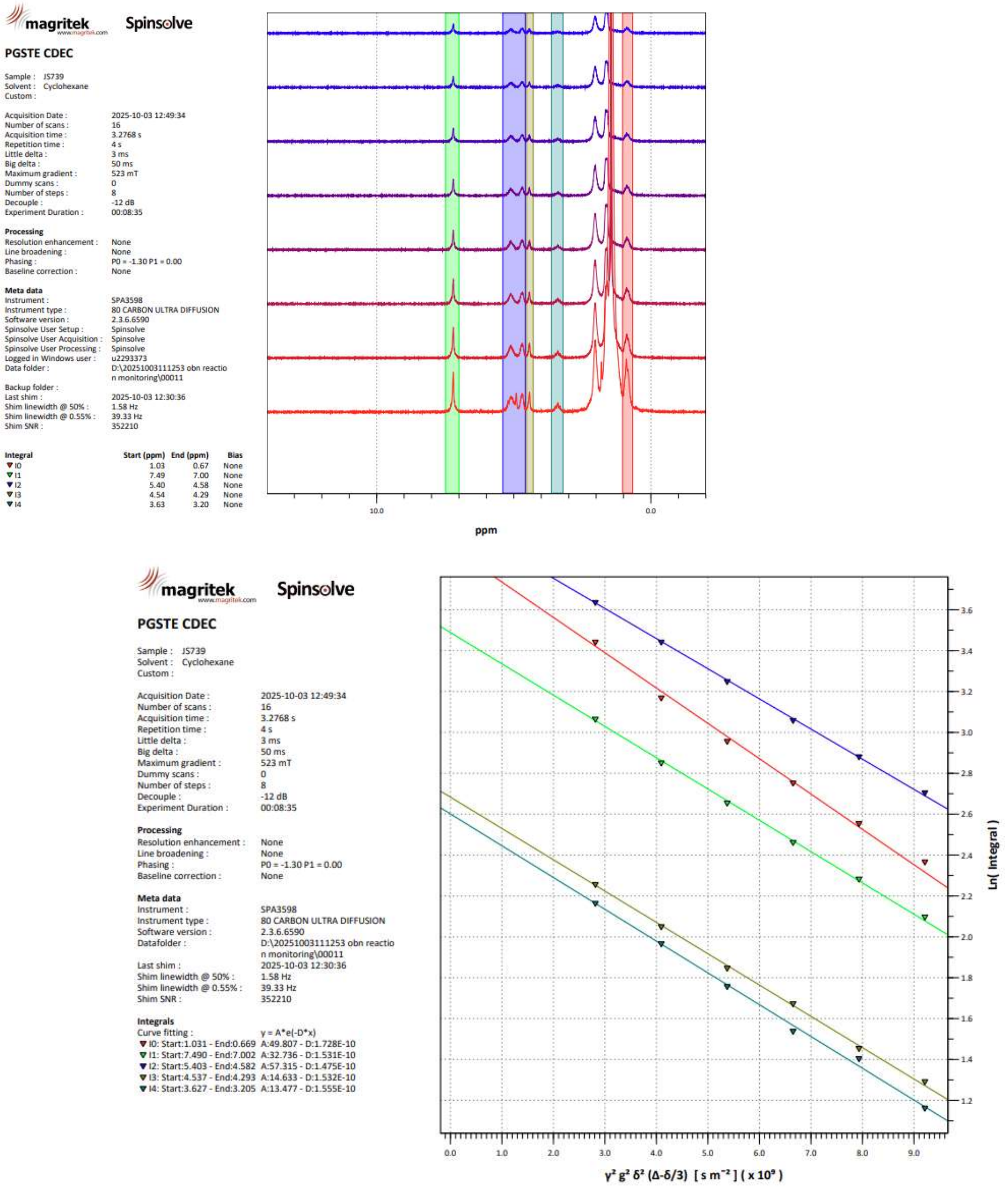

Figure S83: T = 2 hr DOSY Spectrum with diffusion constants calculated from separate regions of the DOSY NMR through Stejskal-Tanner plot. Copolymer of 2 (Bn) and Isoprene (Low MWt)

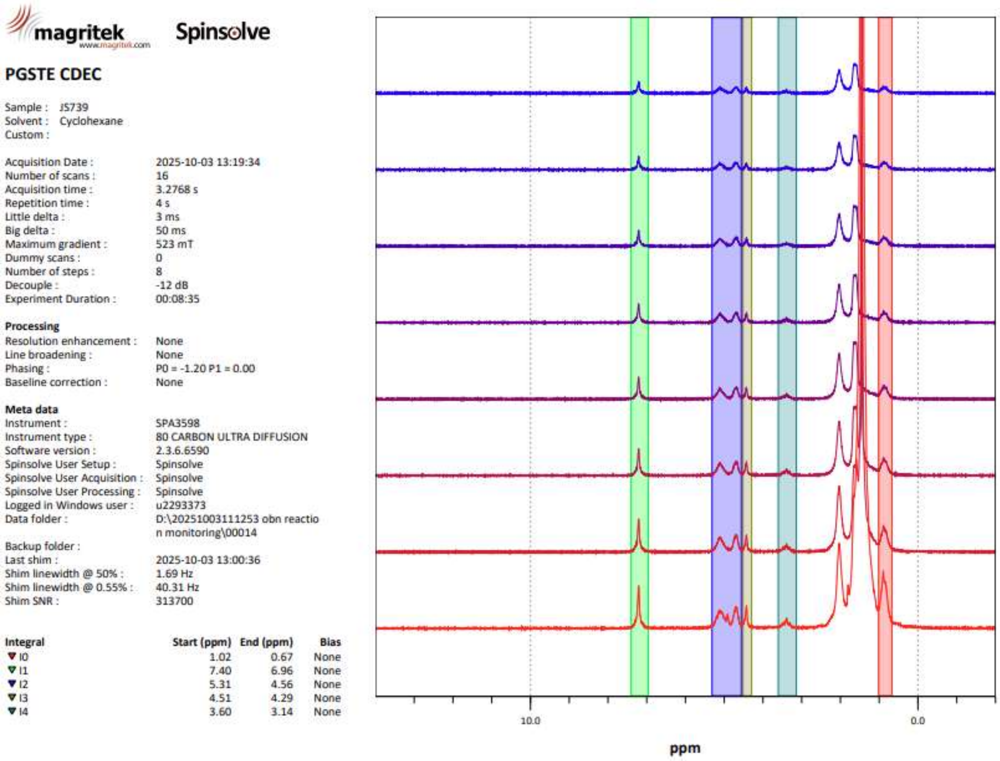

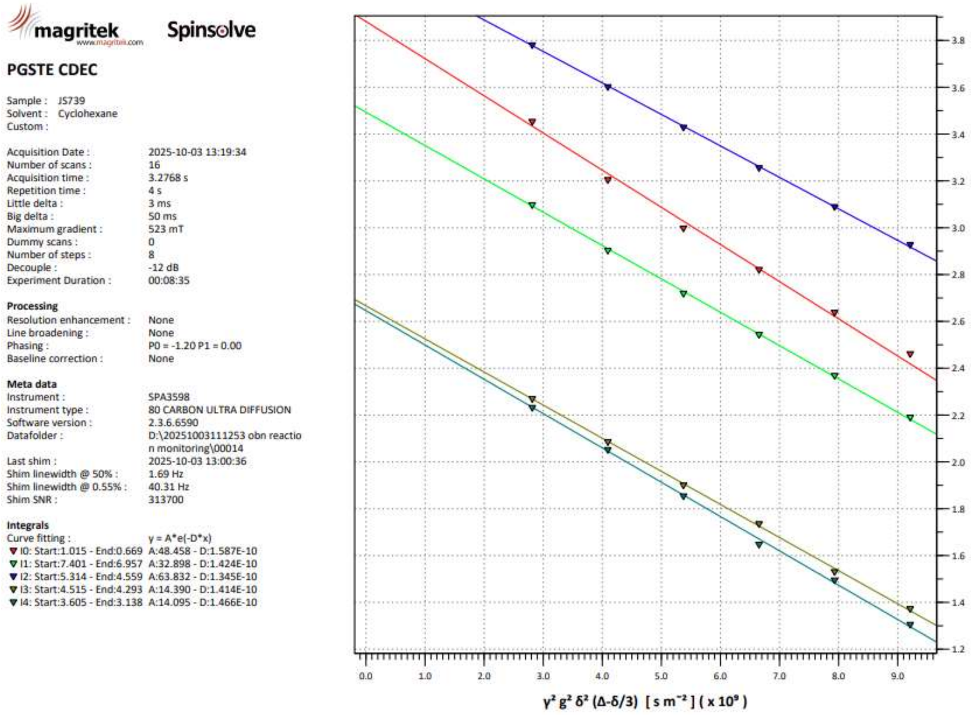

Figure S84: T = 2.5 hr DOSY Spectrum with diffusion constants calculated from separate regions of the DOSY NMR through Stejskal-Tanner plot. Copolymer of 2 (Bn) and Isoprene (Low MWt)

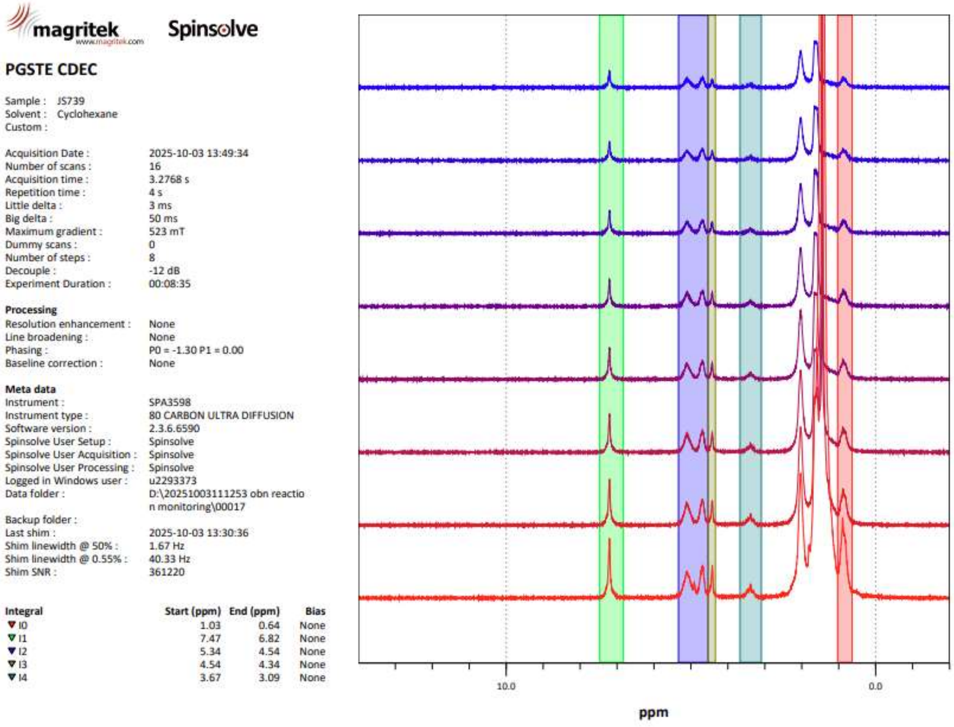

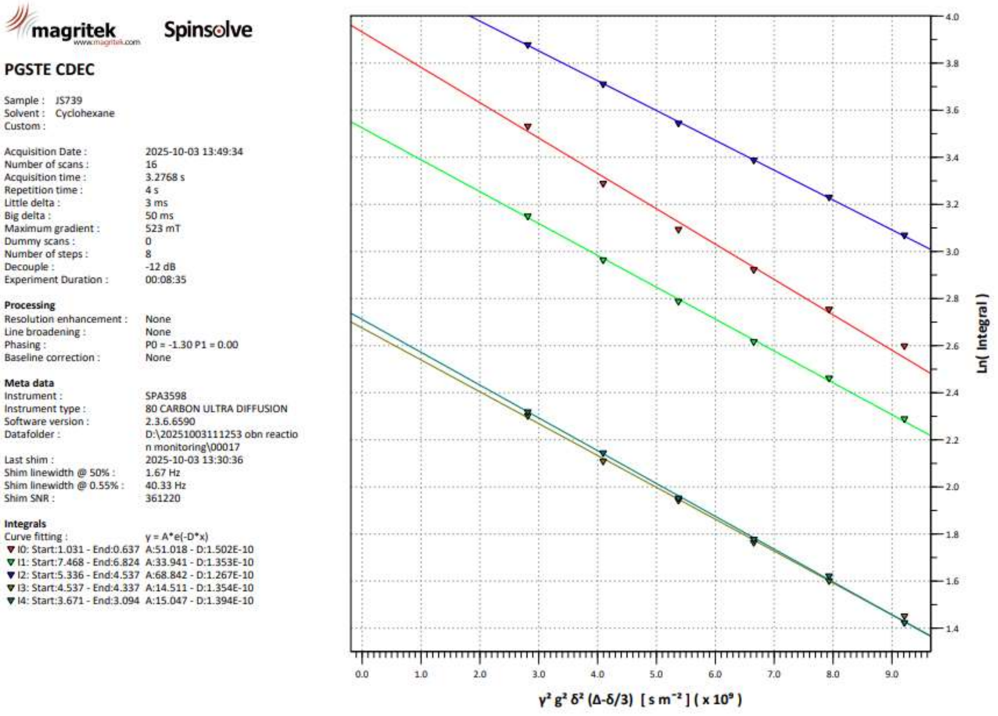

Figure S85: T = 3 hr DOSY Spectrum with diffusion constants calculated from separate regions of the DOSY NMR through Stejskal-Tanner plot. Copolymer of 2 (Bn) and Isoprene (Low MWt)

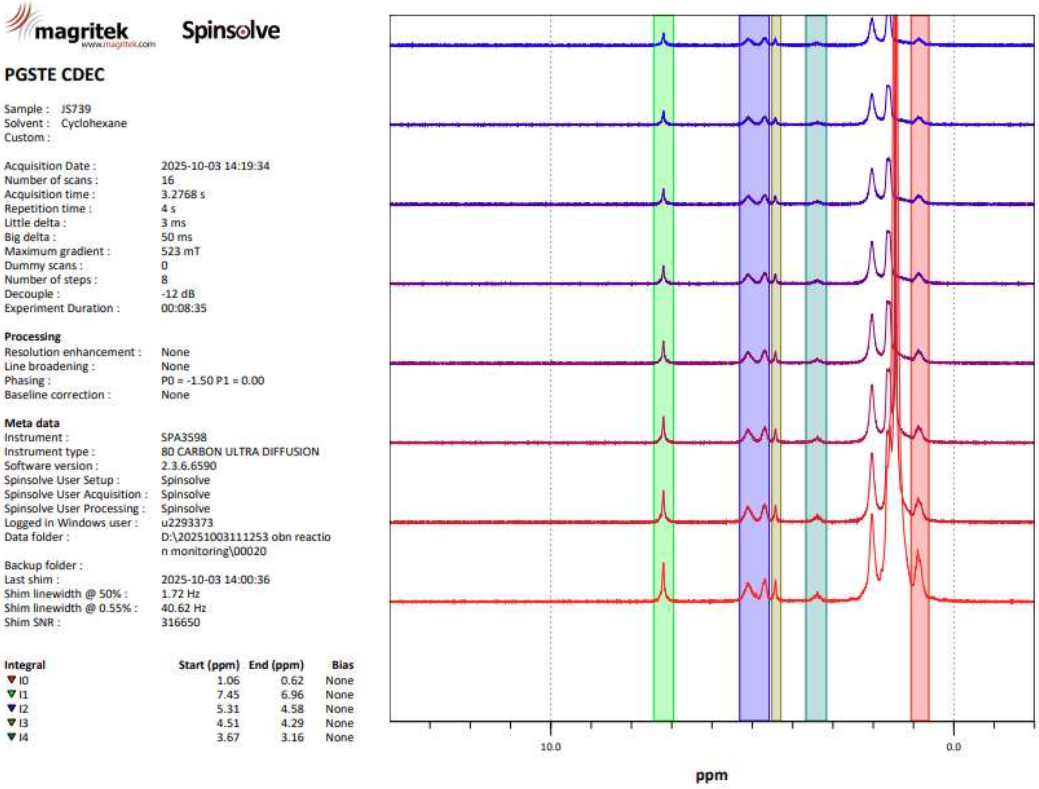

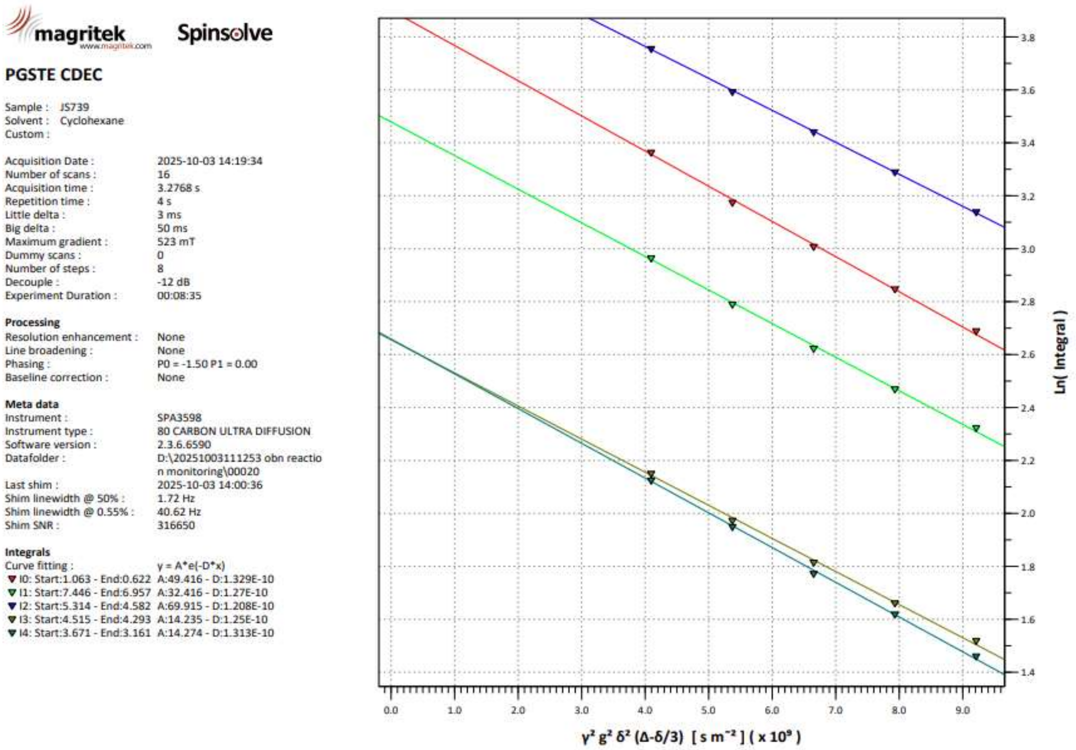

Figure S86: T = 3.5 hr DOSY Spectrum with diffusion constants calculated from separate regions of the DOSY NMR through Stejskal-Tanner plot. Copolymer of 2 (Bn) and Isoprene (Low MWt)

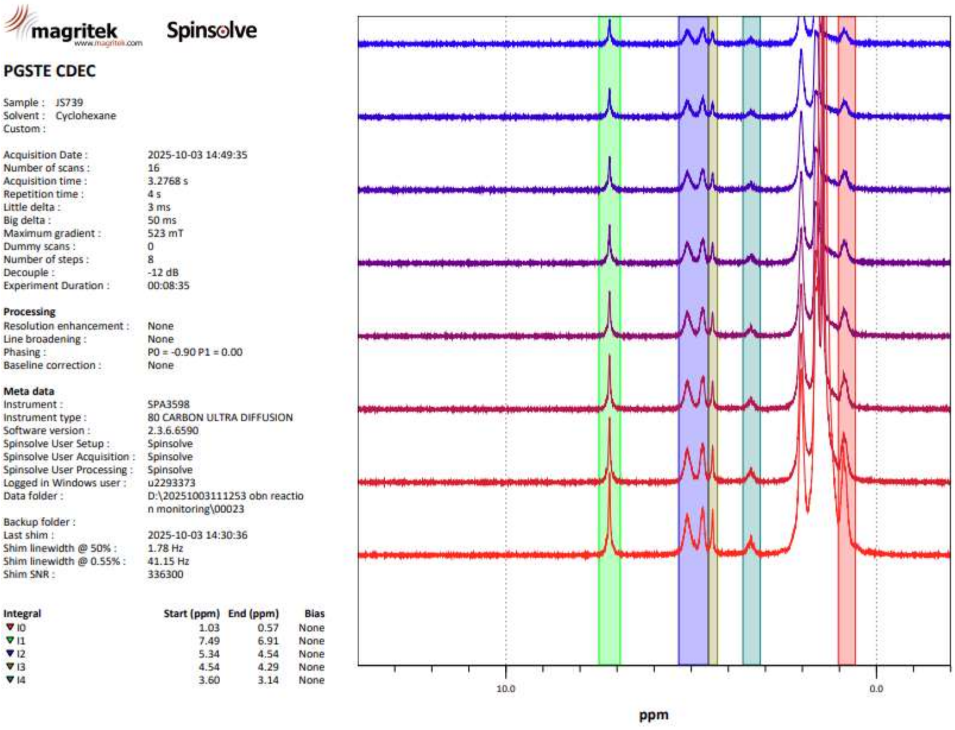

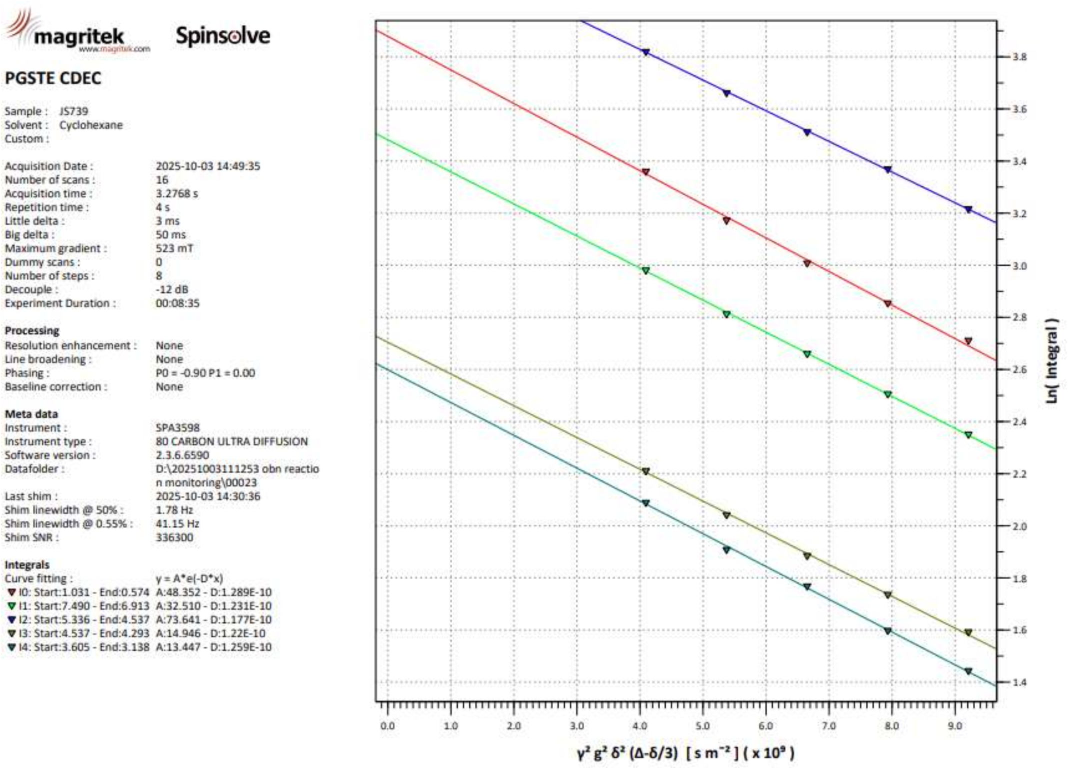

Figure S87: T = 4 hr DOSY Spectrum with diffusion constants calculated from separate regions of the DOSY NMR through Stejskal-Tanner plot. Copolymer of 2 (Bn) and Isoprene (Low MWt)

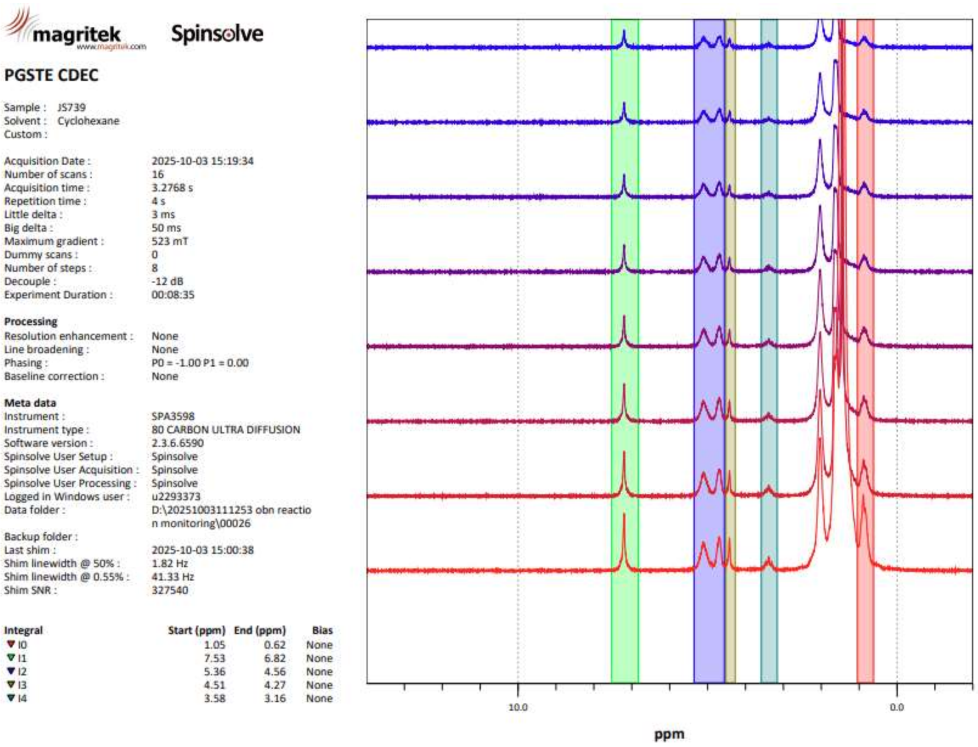

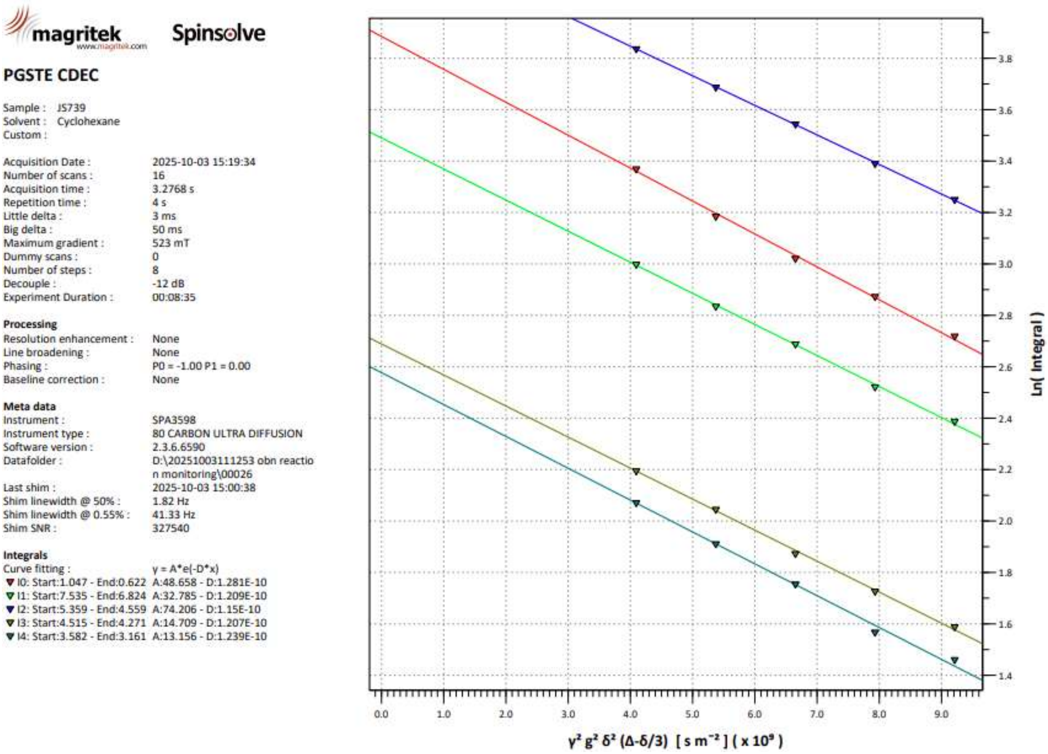

Figure S88: T = 4.5 hr DOSY Spectrum with diffusion constants calculated from separate regions of the DOSY NMR through Stejskal-Tanner plot. Copolymer of 2 (Bn) and Isoprene (Low MWt)

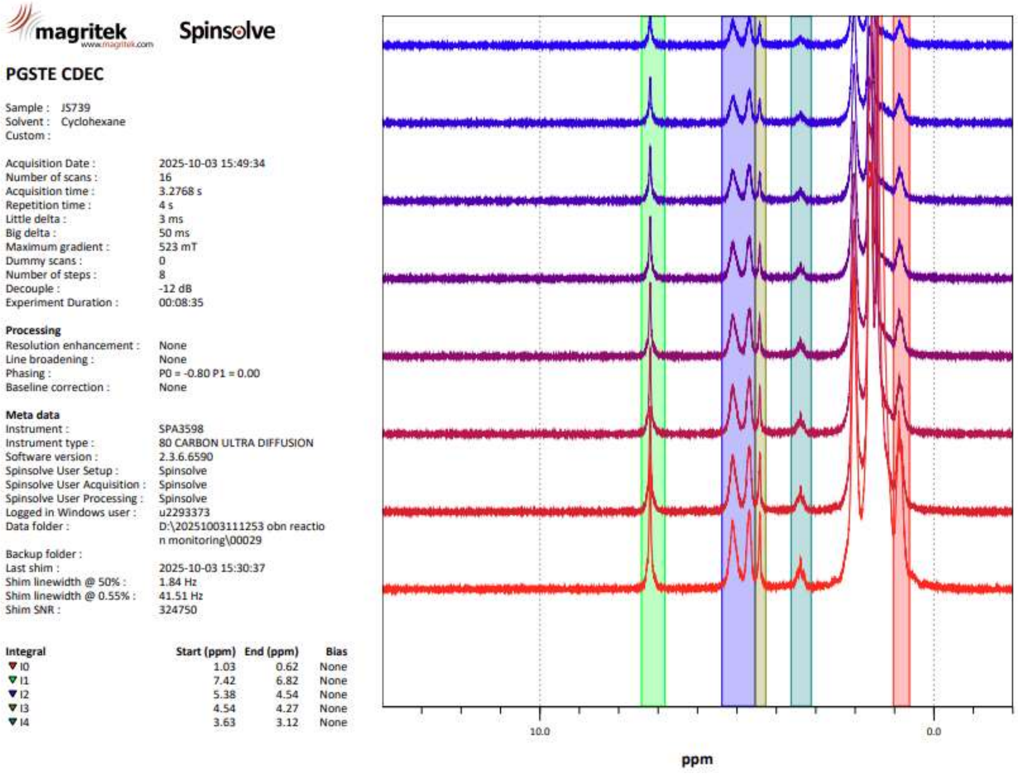

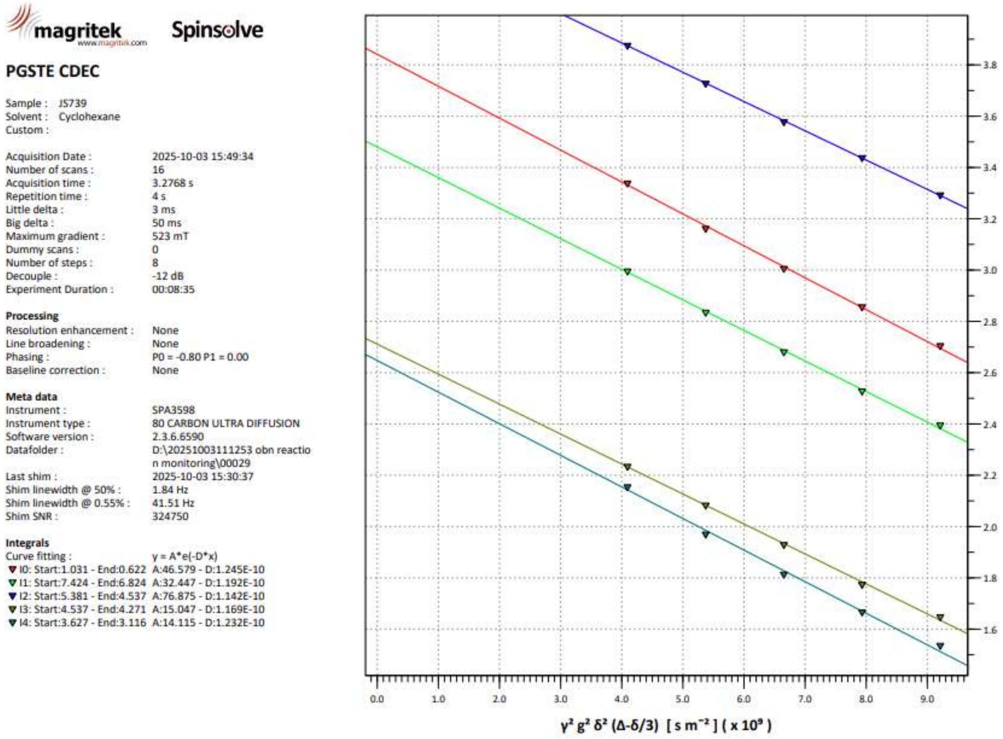

Figure S89: T = 5 hr DOSY Spectrum with diffusion constants calculated from separate regions of the DOSY NMR through Stejskal-Tanner plot. Copolymer of 2 (Bn) and Isoprene (Low MWt)

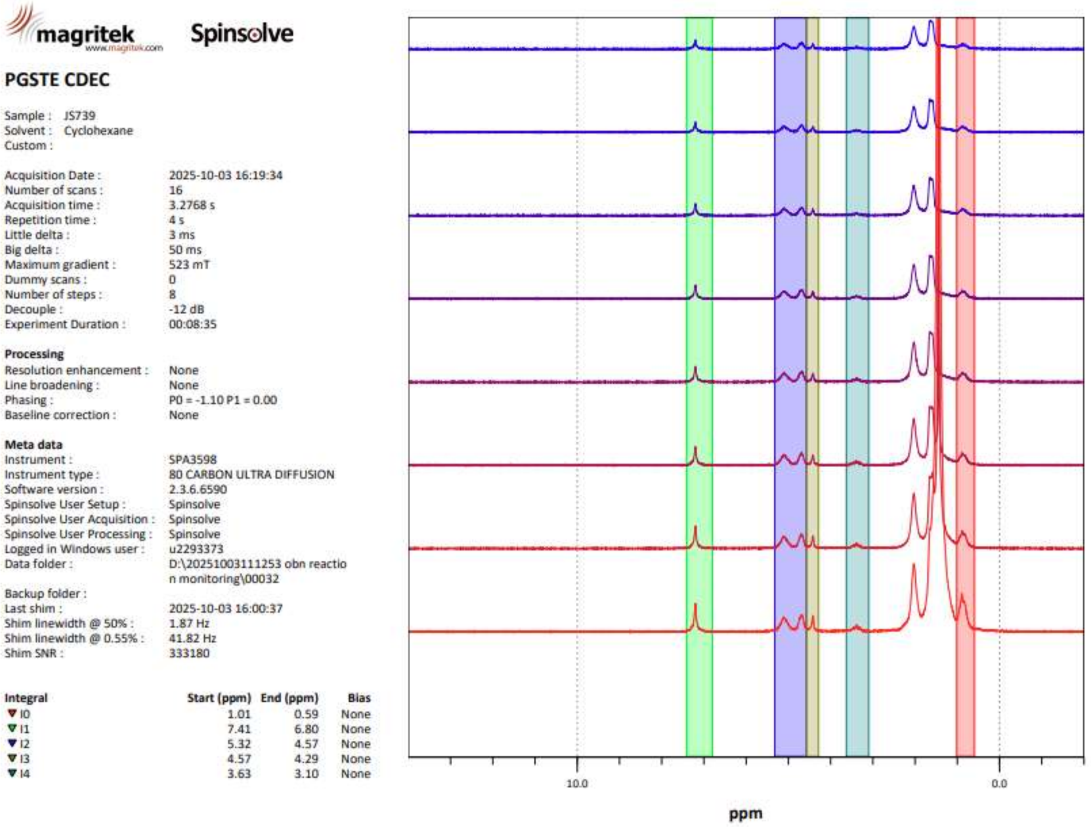

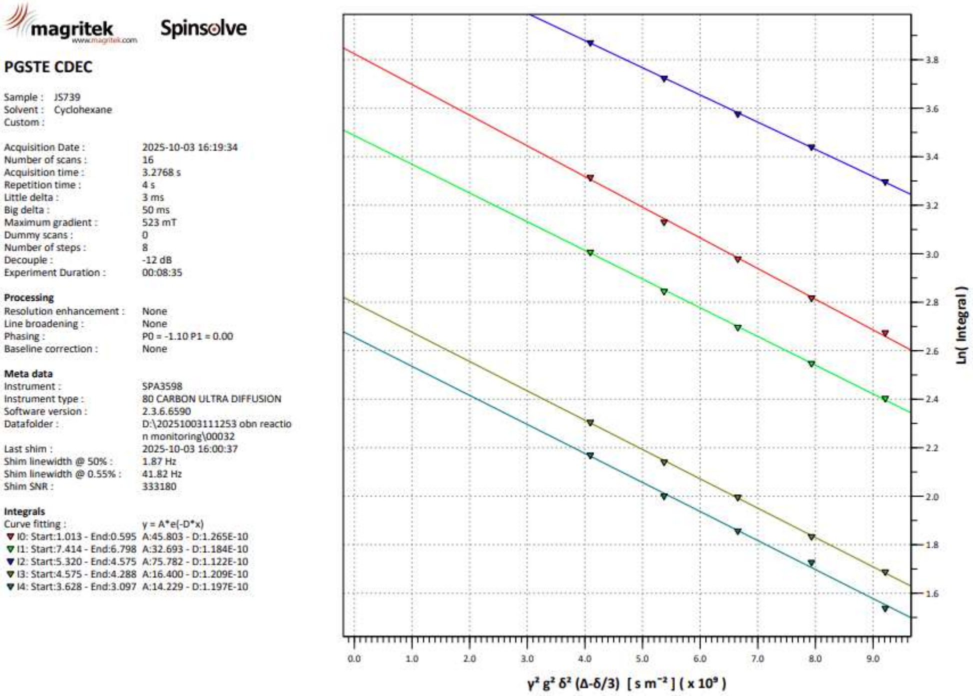

Figure S90: T = 5.5 hr DOSY Spectrum with diffusion constants calculated from separate regions of the DOSY NMR through Stejskal-Tanner plot. Copolymer of 2 (Bn) and Isoprene (Low MWt)

magritek www.magritek.com Spinsolve

PGSTE CDEC

Sample : JS739
Solvent : Cyclohexane
Custom :

Acquisition Date :	2025-10-03 16:49:34
Number of scans :	16
Acquisition time :	3.2768 s
Repetition time :	4 s
Little delta :	3 ms
Big delta :	50 ms
Maximum gradient :	523 mT
Dummy scans :	0
Number of steps :	8
Decouple :	-12 dB
Experiment Duration :	00:08:35

Processing

Resolution enhancement :	None
Line broadening :	None
Phasing :	P0 = -1.30 P1 = 0.00
Baseline correction :	None

Meta data

Instrument :	SPA3598
Instrument type :	80 CARBON ULTRA DIFFUSION
Software version :	2.3.6.6590
Spinsolve User Setup :	Spinsolve
Spinsolve User Acquisition :	Spinsolve
Spinsolve User Processing :	Spinsolve
Logged in Windows user :	u2293373
Data folder :	D:\20251003111253 obn reaction monitoring\00035
Backup folder :	
Last shim :	2025-10-03 16:30:37
Shim linewidth @ 50% :	1.88 Hz
Shim linewidth @ 0.55% :	41.74 Hz
Shim SNR :	332510

Integral	Start (ppm)	End (ppm)	Bias
I0	1.03	0.62	None
I1	7.41	6.97	None
I2	5.38	4.55	None
I3	4.52	4.32	None
I4	3.57	3.23	None

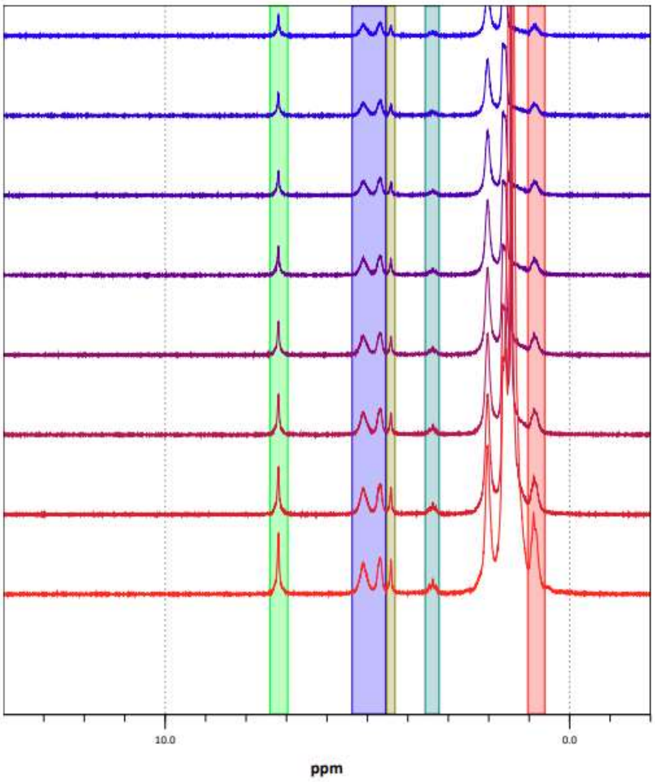

magritek www.magritek.com Spinsolve

PGSTE CDEC

Sample : JS739
Solvent : Cyclohexane
Custom :

Acquisition Date : 2025-10-03 16:49:34
Number of scans : 16
Acquisition time : 3.2768 s
Repetition time : 4 s
Little delta : 3 ms
Big delta : 50 ms
Maximum gradient : 523 mT
Dummy scans : 0
Number of steps : 8
Decouple : -12 dB
Experiment Duration : 00:08:35

Processing
Resolution enhancement : None
Line broadening : None
Phasing : P0 = -1.30 P1 = 0.00
Baseline correction : None

Meta data

Instrument :	SPA3598
Instrument type :	80 CARBON ULTRA DIFFUSION
Software version :	2.3.6.6590
Datafolder :	D:\20251003111253 obn reaction monitoring\00035
Last shim :	2025-10-03 16:30:37
Shim linewidth @ 50% :	1.88 Hz
Shim linewidth @ 0.55% :	41.74 Hz
Shim SNR :	332510

Integrals

Curve fitting : y = A*e(-D*x)
I0: Start:1.034 - End:0.615 A:46.705 - D:1.242E-10
I1: Start:7.414 - End:6.970 A:32.049 - D:1.201E-10
I2: Start:5.378 - End:4.546 A:77.710 - D:1.115E-10
I3: Start:4.517 - End:4.316 A:14.050 - D:1.185E-10
I4: Start:3.571 - End:3.227 A:12.639 - D:1.205E-10

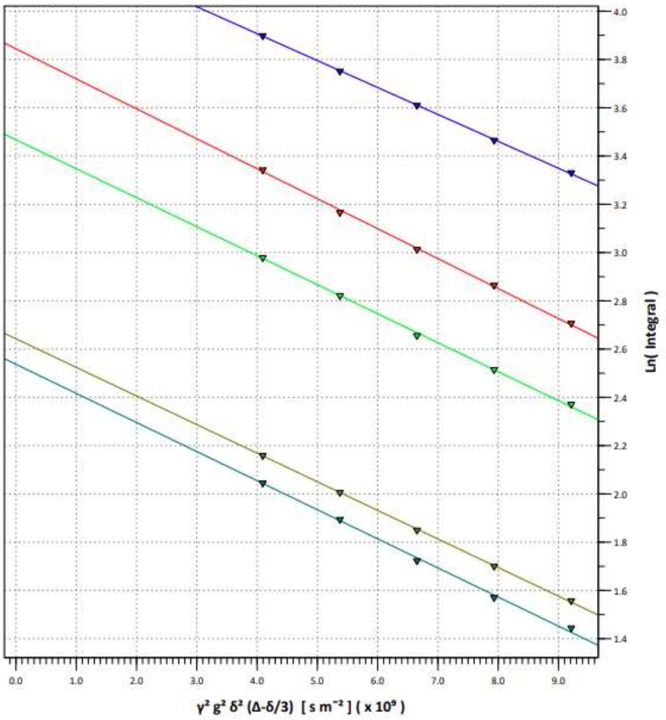

Figure S91: T = 6 hr DOSY Spectrum with diffusion constants calculated from separate regions of the DOSY NMR through Stejskal-Tanner plot. Copolymer of 2 (Bn) and Isoprene (Low MWt)

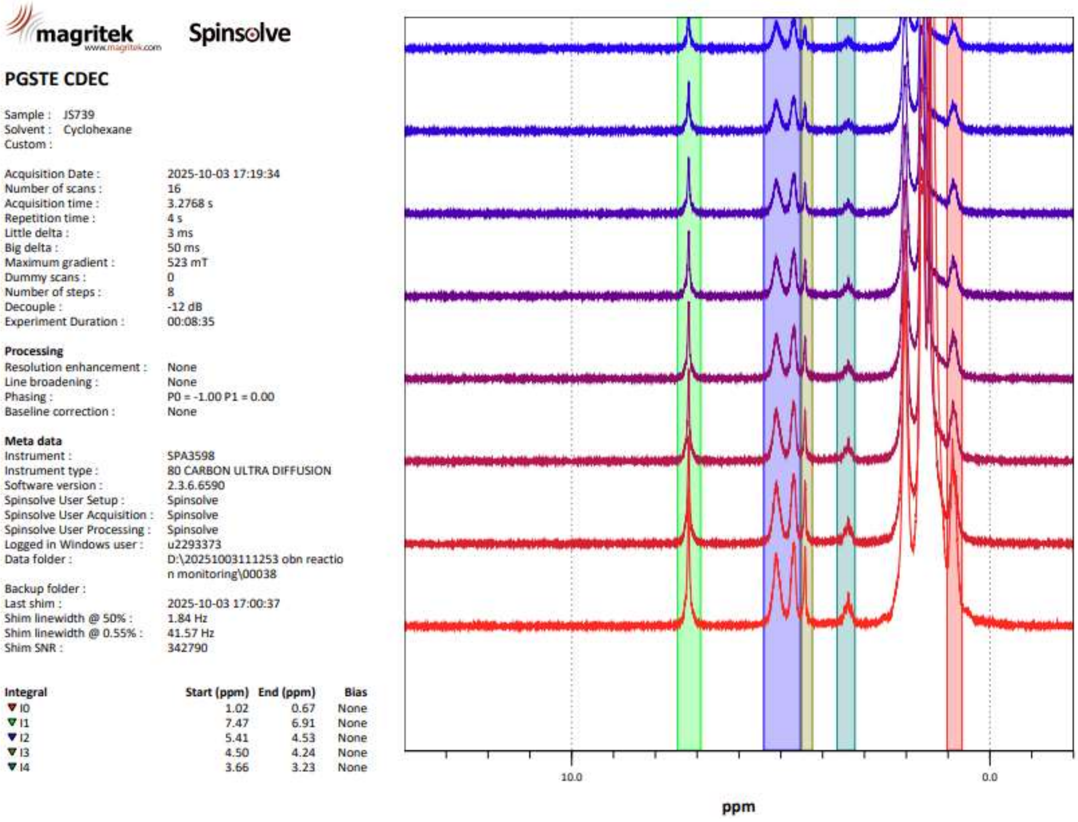

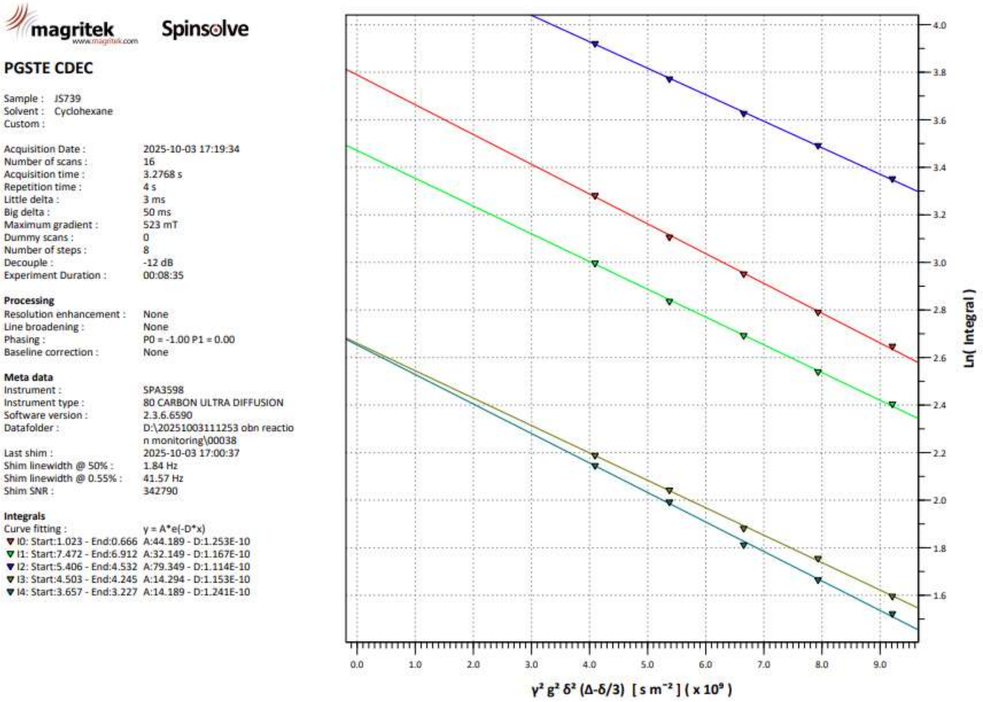

Figure S92: T = 6.5 hr DOSY Spectrum with diffusion constants calculated from separate regions of the DOSY NMR through Stejskal-Tanner plot. Copolymer of 2 (Bn) and Isoprene (Low MWt)

magritek www.magritek.com Spinsolve

1D EXTENDED+

Sample : JS739
Solvent : Cyclohexane
Custom :

Acquisition Date : 2025-10-04 08:15:16
Number of scans : 16
Acquisition time : 6.5536 s
Repetition time : 15 s
Pulse angle : 90 degrees
Experiment Duration : 00:04:03

Processing
Resolution enhancement : None
Line broadening : None
Phasing : P0 = 6.00 P1 = 0.00
Baseline correction : None

Meta data
Instrument : SPA3598
Instrument type : 80 CARBON ULTRA DIFFUSION
Software version : 2.3.6.6590
Spinsolve User Setup : Spinsolve
Spinsolve User Acquisition : Spinsolve
Spinsolve User Processing : Spinsolve
Logged in Windows user : u2293373
Data folder : D:\20251003111253 obn reaction monitoring\00127

Backup folder :
Last shim : 2025-10-04 08:00:39
Shim linewidth @ 50% : 1.66 Hz
Shim linewidth @ 0.55% : 40.27 Hz
Shim SNR : 353060

Integrals

PPM Range		Normalized	Absolute
3.54 -	3.16	7.85 %	28.04
4.88 -	4.49	29.07 %	103.88
5.41 -	4.89	35.12 %	125.49
7.41 -	6.95	27.96 %	99.93

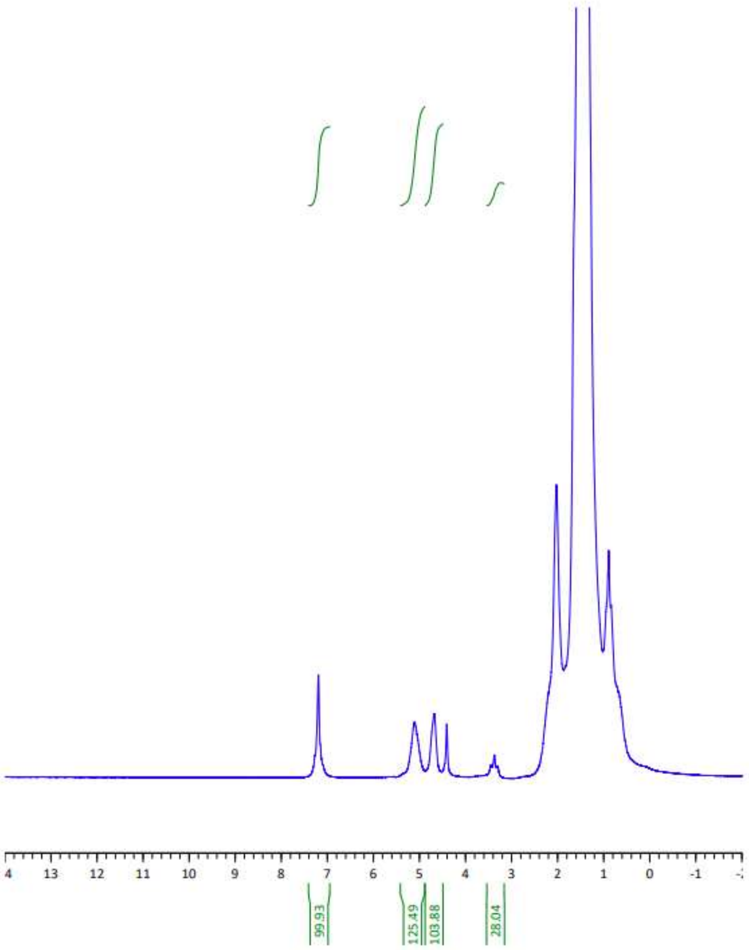

Figure S93: Copolymer of 2 (Bn) and Isoprene (Low MWt) final NMR:

Copolymer of 2 and Isoprene (High MWt)

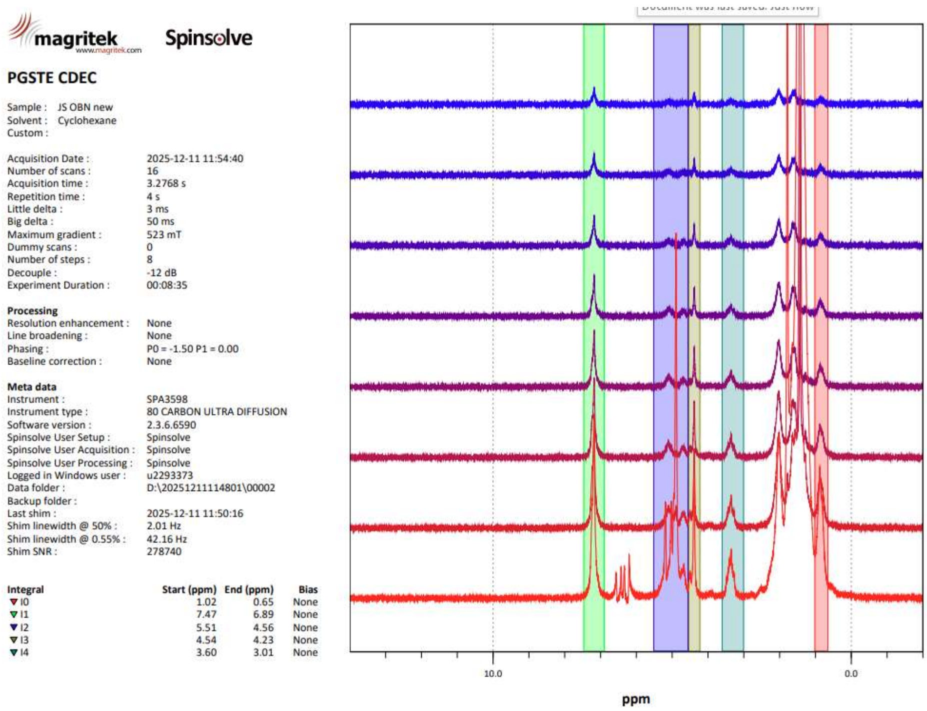

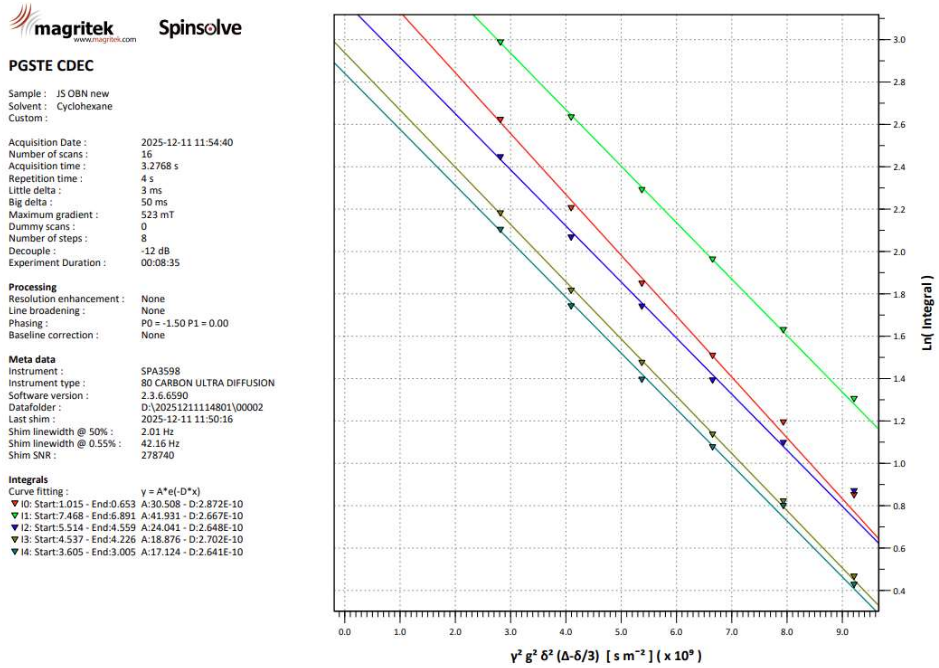

Figure S94: T = 30 Min DOSY Spectrum with diffusion constants calculated from separate regions of the DOSY NMR through Stejskal-Tanner plot.

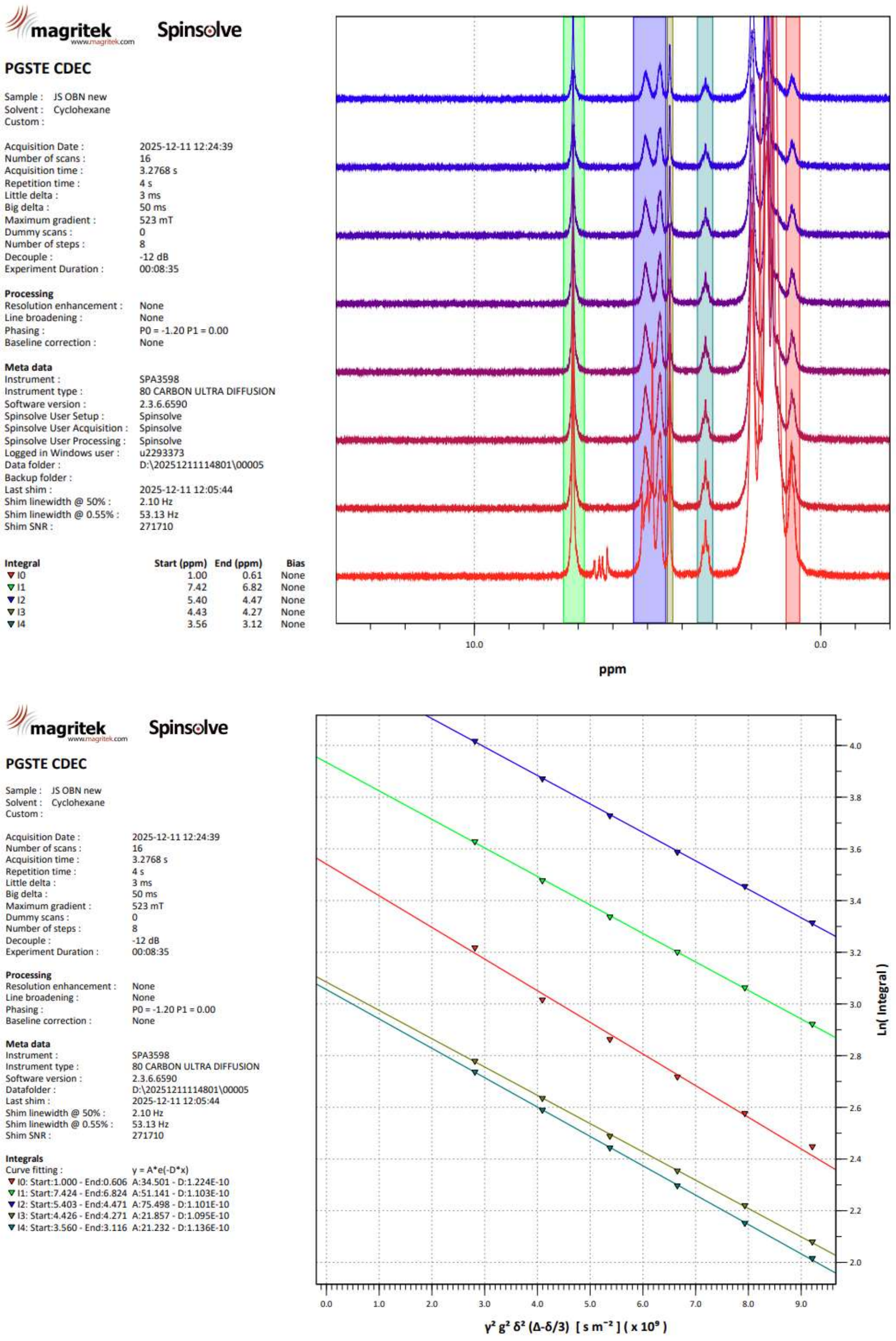

Figure S95: T = 60 Min DOSY Spectrum with diffusion constants calculated from separate regions of the DOSY NMR through Stejskal-Tanner plot. Copolymer of 2 (Bn) and Isoprene (High MWt).

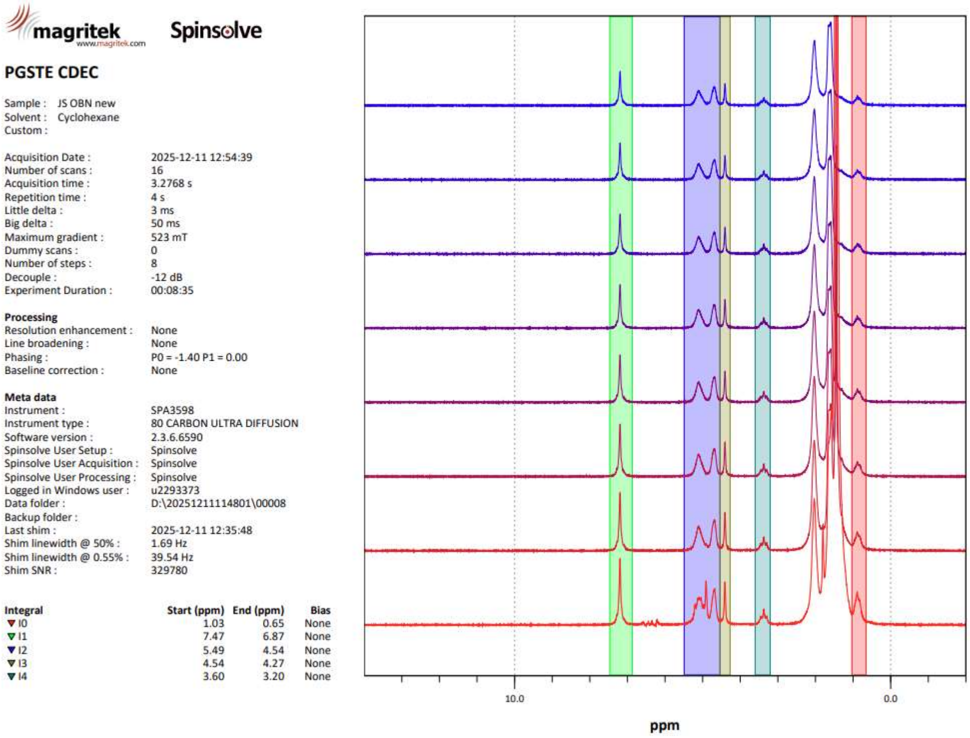

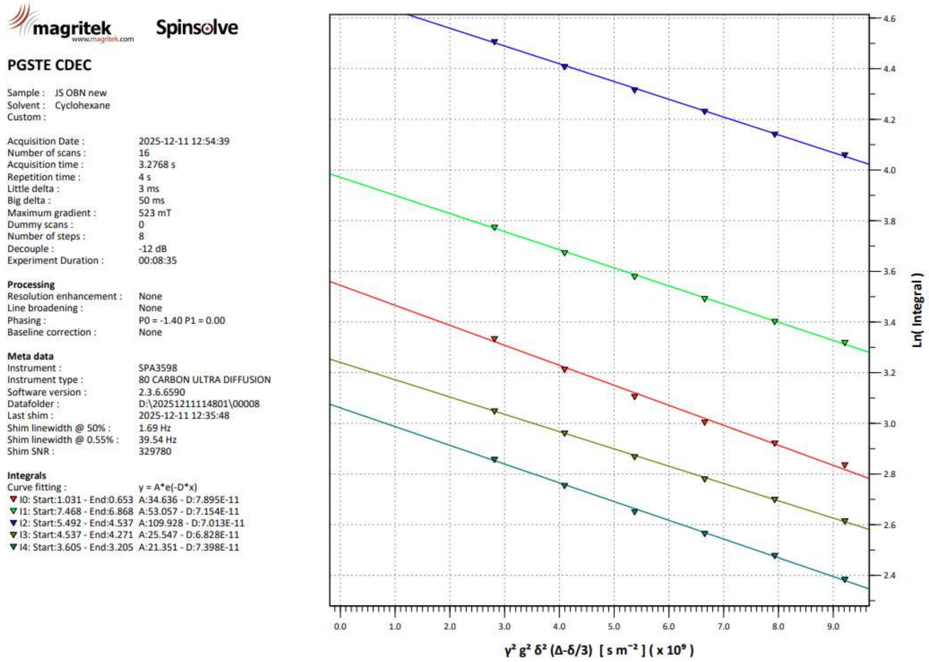

Figure S96: T = 90 Min DOSY Spectrum with diffusion constants calculated from separate regions of the DOSY NMR through Stejskal-Tanner plot. Copolymer of 2 (Bn) and Isoprene (High MWt).

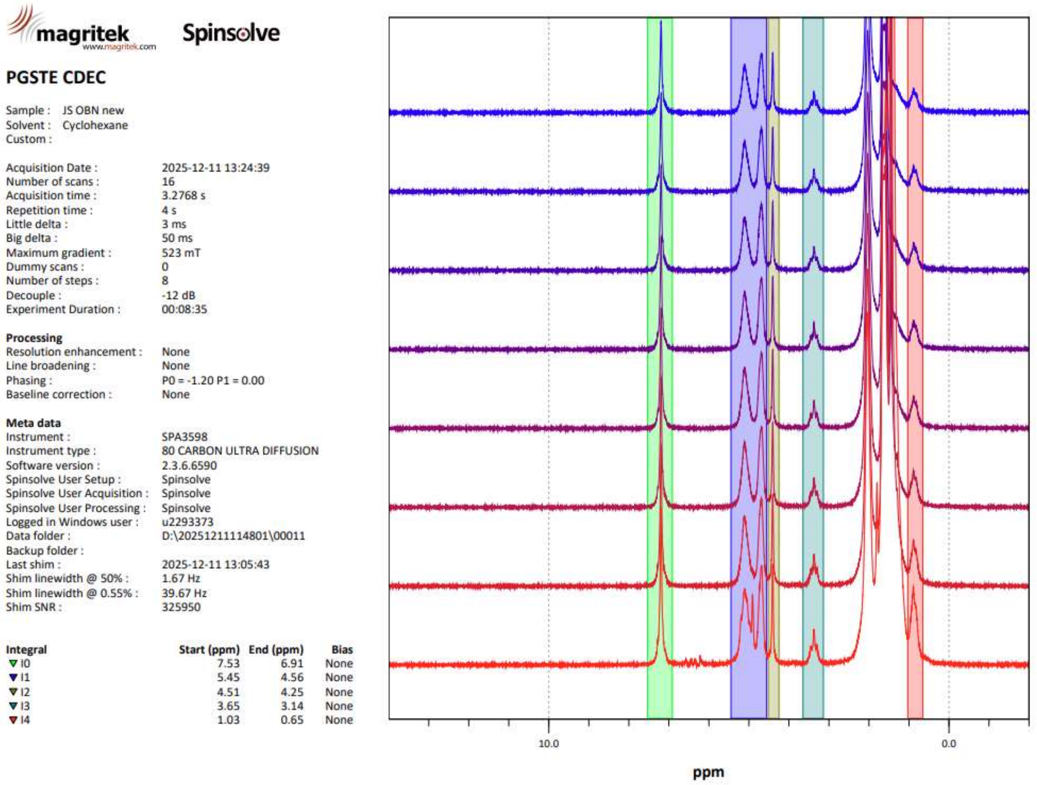

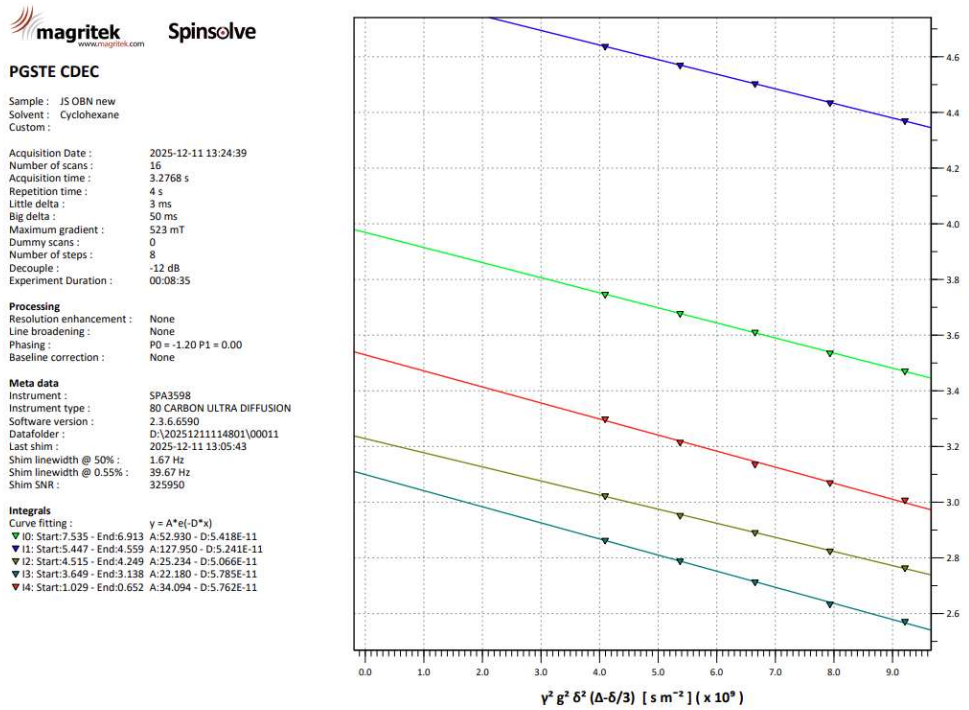

Figure S97: T = 120 Min DOSY Spectrum with diffusion constants calculated from separate regions of the DOSY NMR through Stejskal-Tanner plot. Copolymer of 2 (Bn) and Isoprene (High MWt).

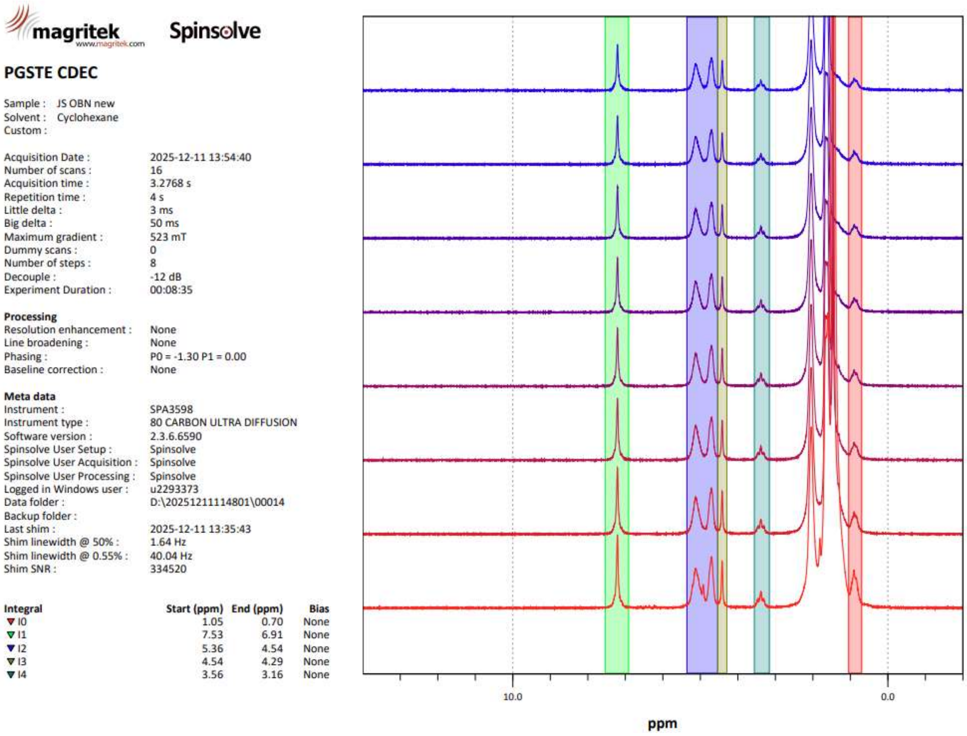

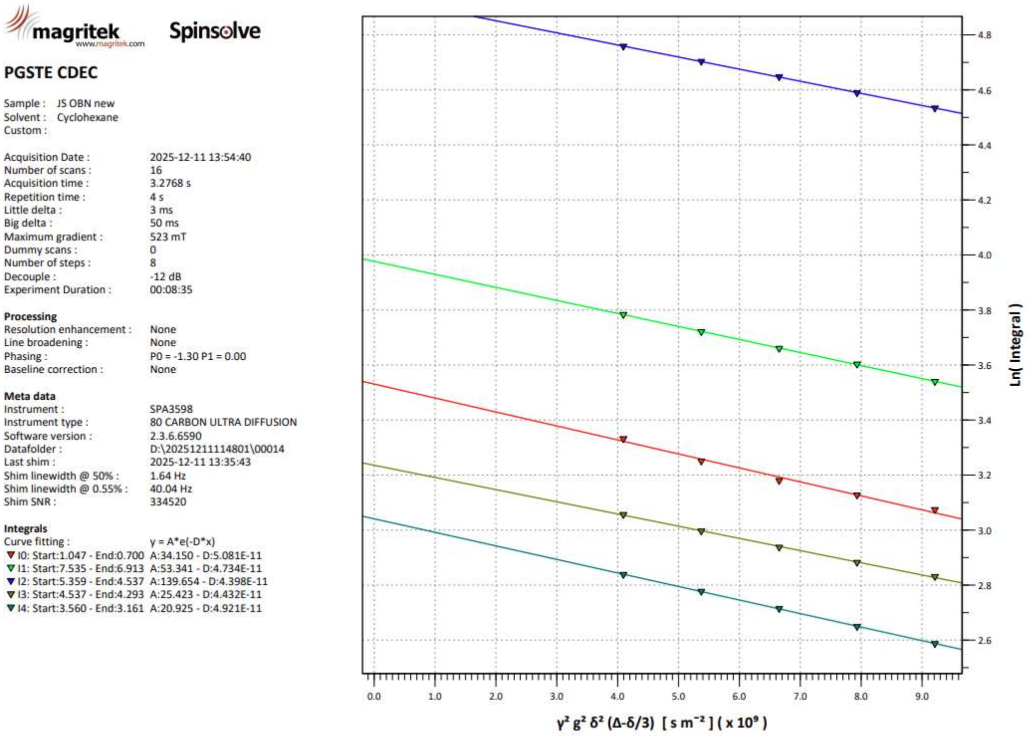

Figure S98: T = 150 Min DOSY Spectrum with diffusion constants calculated from separate regions of the DOSY NMR through Stejskal-Tanner plot. Copolymer of 2 (Bn) and Isoprene (High MWt).

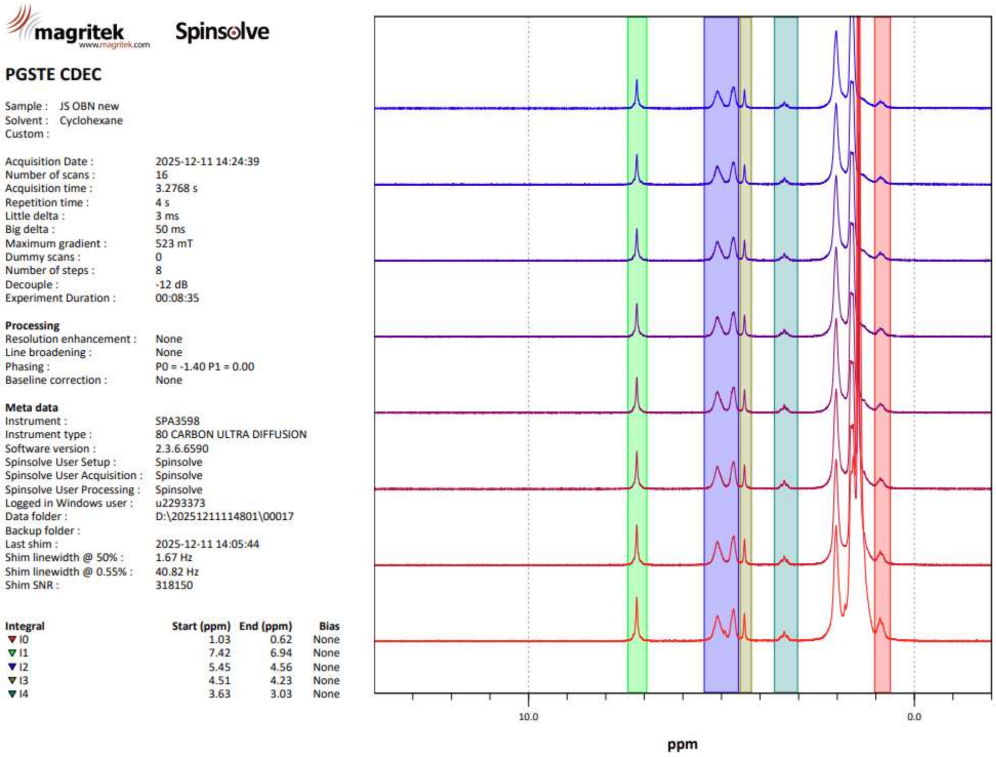

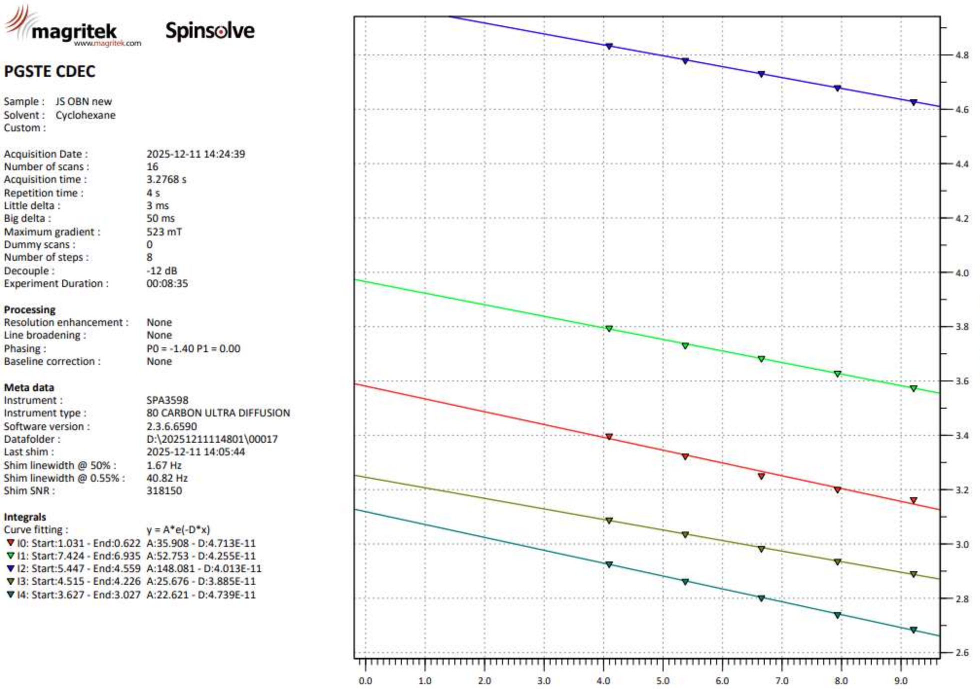

Figure S99: T = 180 Min DOSY Spectrum with diffusion constants calculated from separate regions of the DOSY NMR through Stejskal-Tanner plot. Copolymer of 2 (Bn) and Isoprene (High MWt).

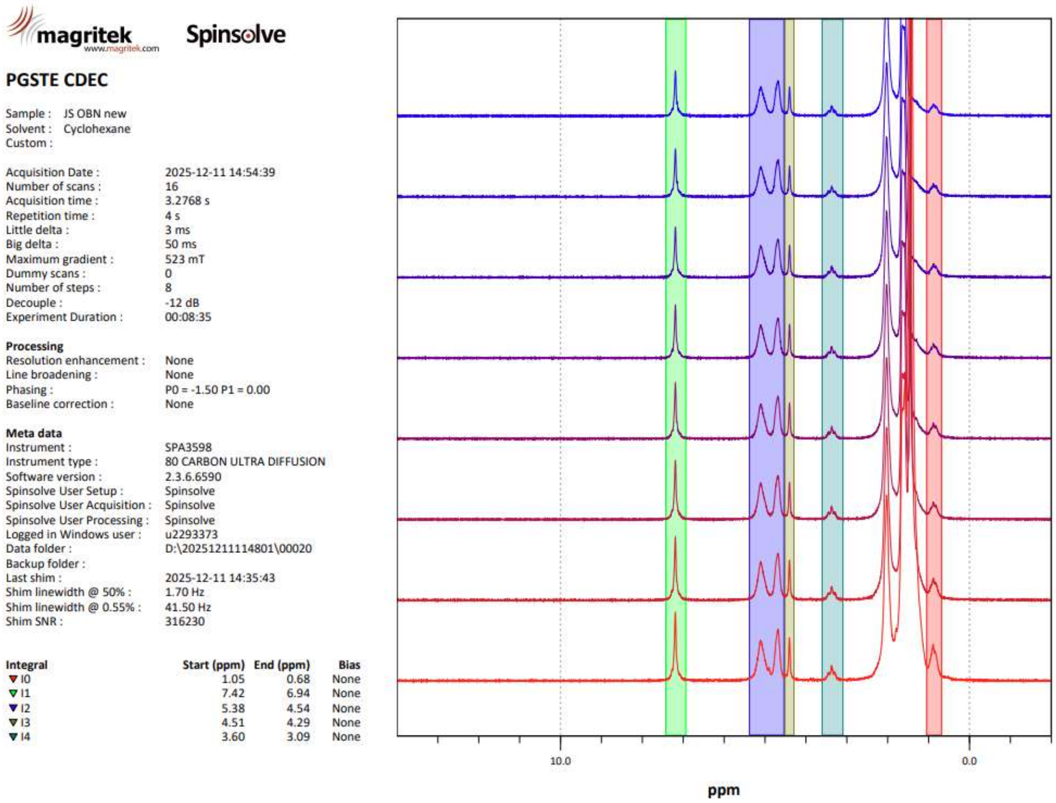

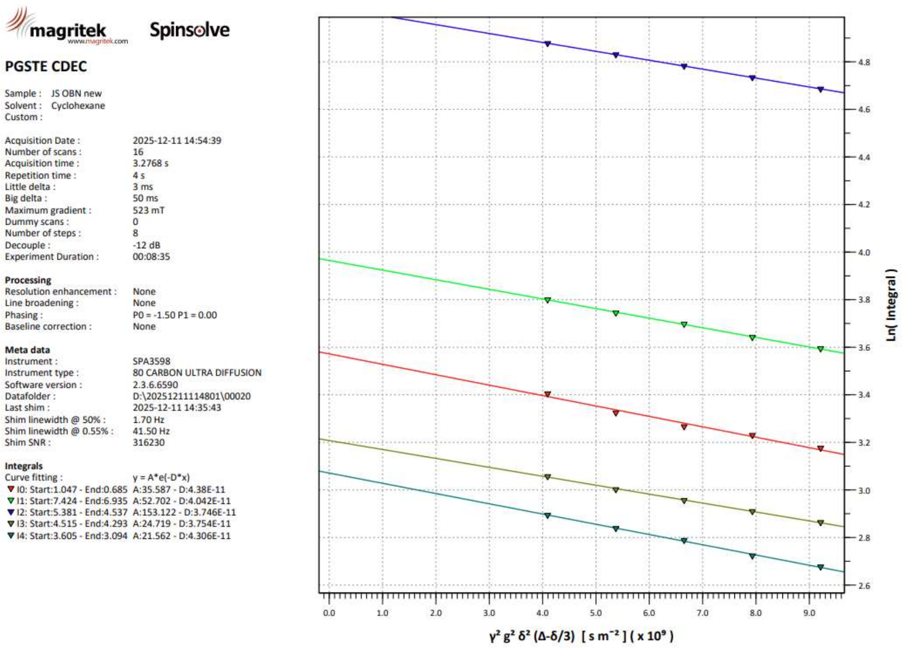

Figure S100: T = 210 Min DOSY Spectrum with diffusion constants calculated from separate regions of the DOSY NMR through Stejskal-Tanner plot. Copolymer of 2 (Bn) and Isoprene (High MWt).

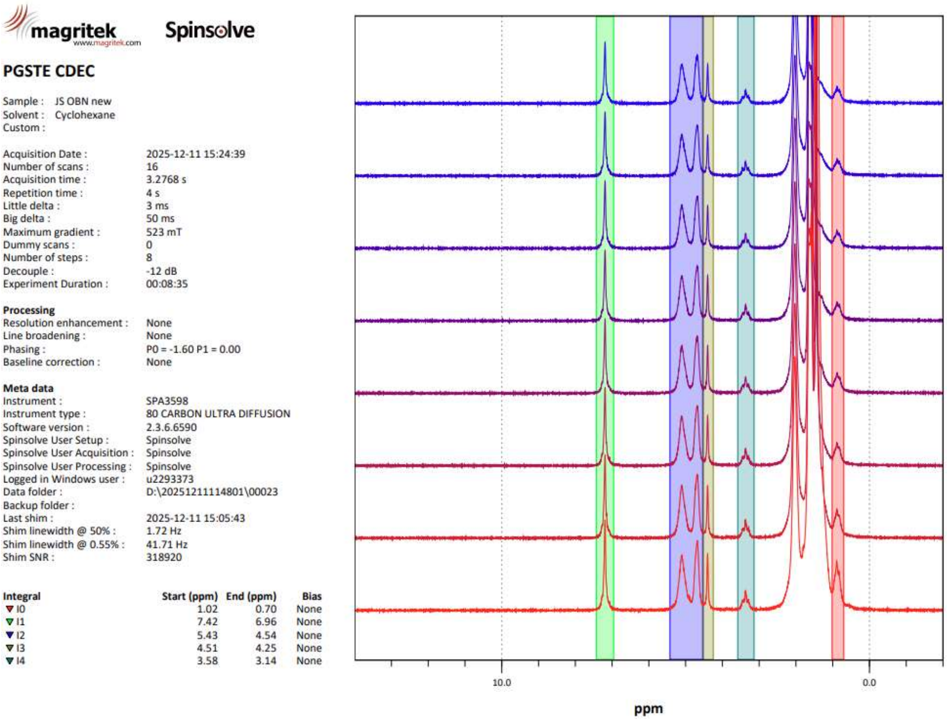

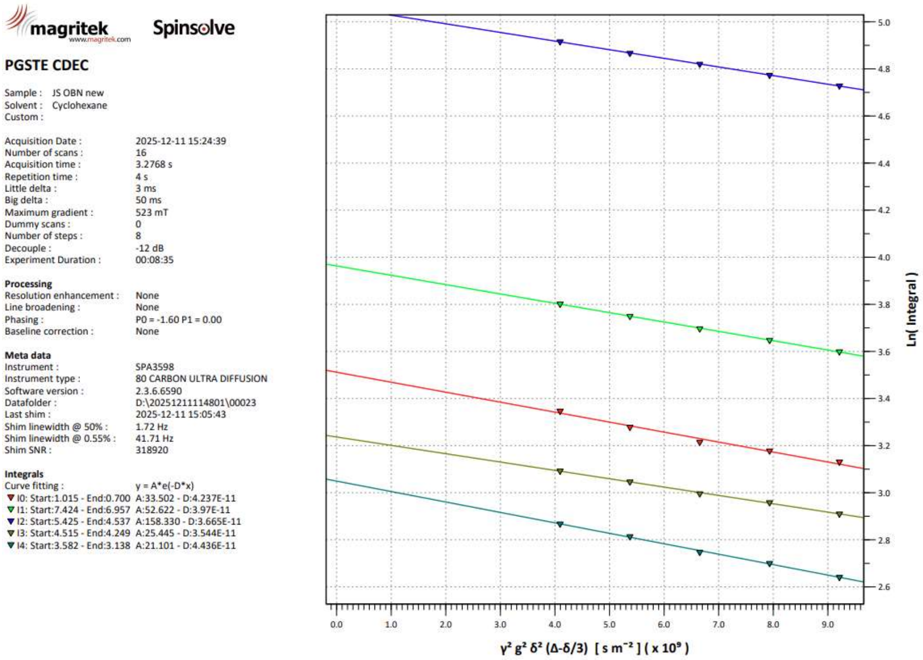

Figure S101: T = 240 Min DOSY Spectrum with diffusion constants calculated from separate regions of the DOSY NMR through Stejskal-Tanner plot. Copolymer of 2 (Bn) and Isoprene (High MWt).

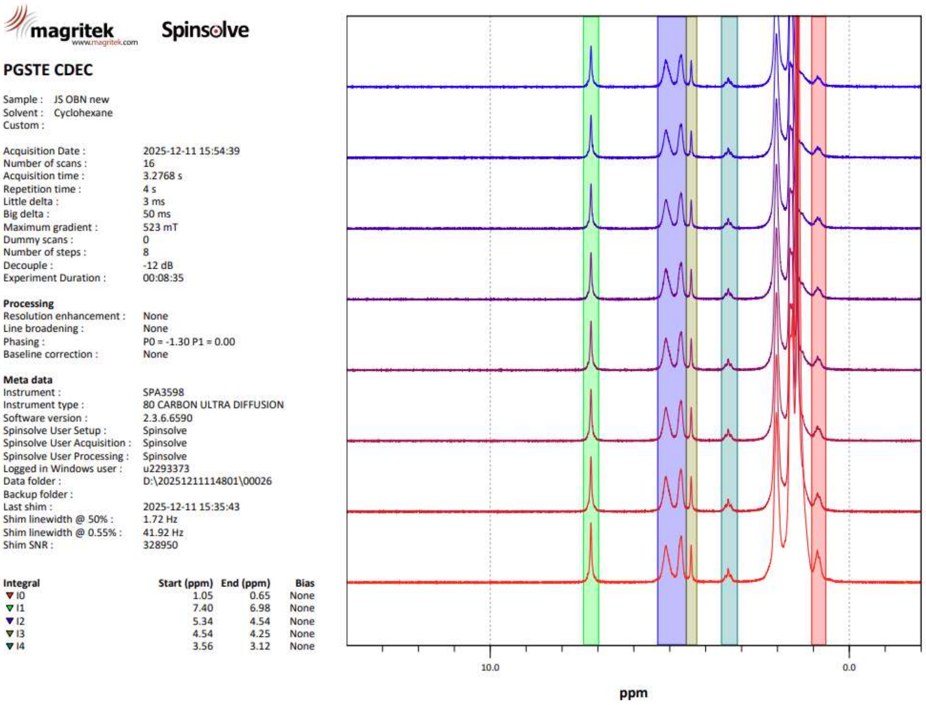

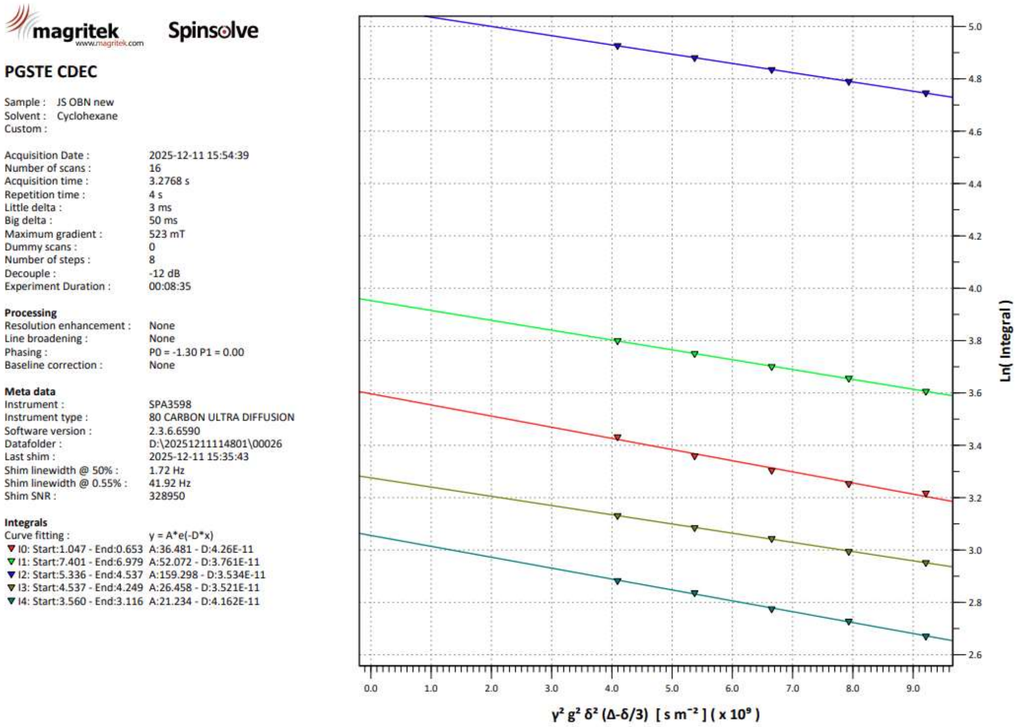

Figure S102: T = 270 Min DOSY Spectrum with diffusion constants calculated from separate regions of the DOSY NMR through Stejskal-Tanner plot. Copolymer of 2 (Bn) and Isoprene (High MWt).

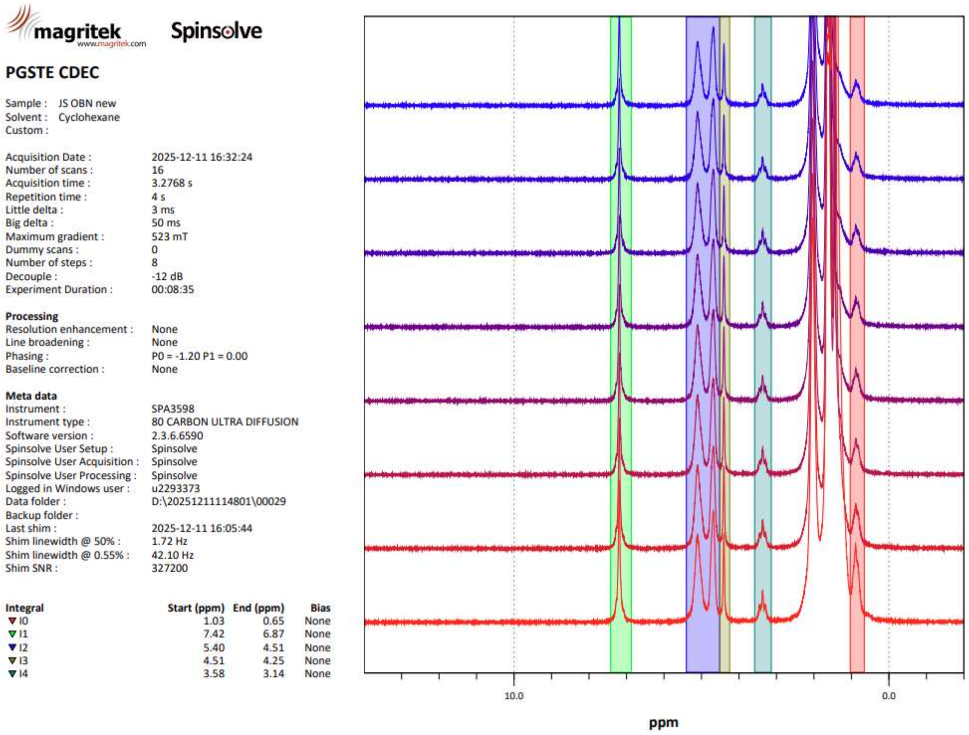

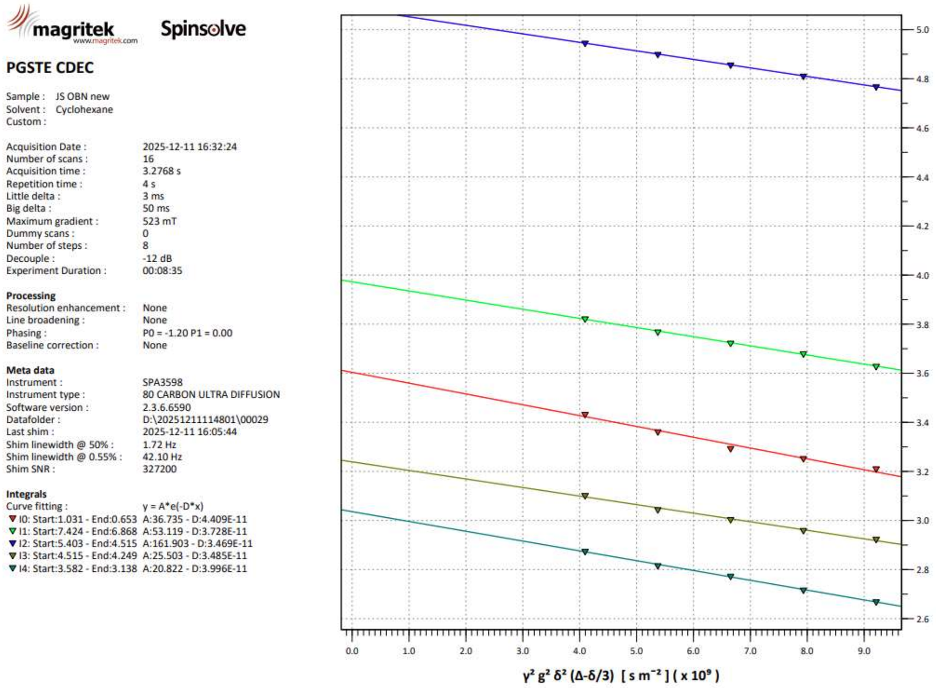

Figure S103: T = 300 Min DOSY Spectrum with diffusion constants calculated from separate regions of the DOSY NMR through Stejskal-Tanner plot. Copolymer of 2 (Bn) and Isoprene (High MWt).

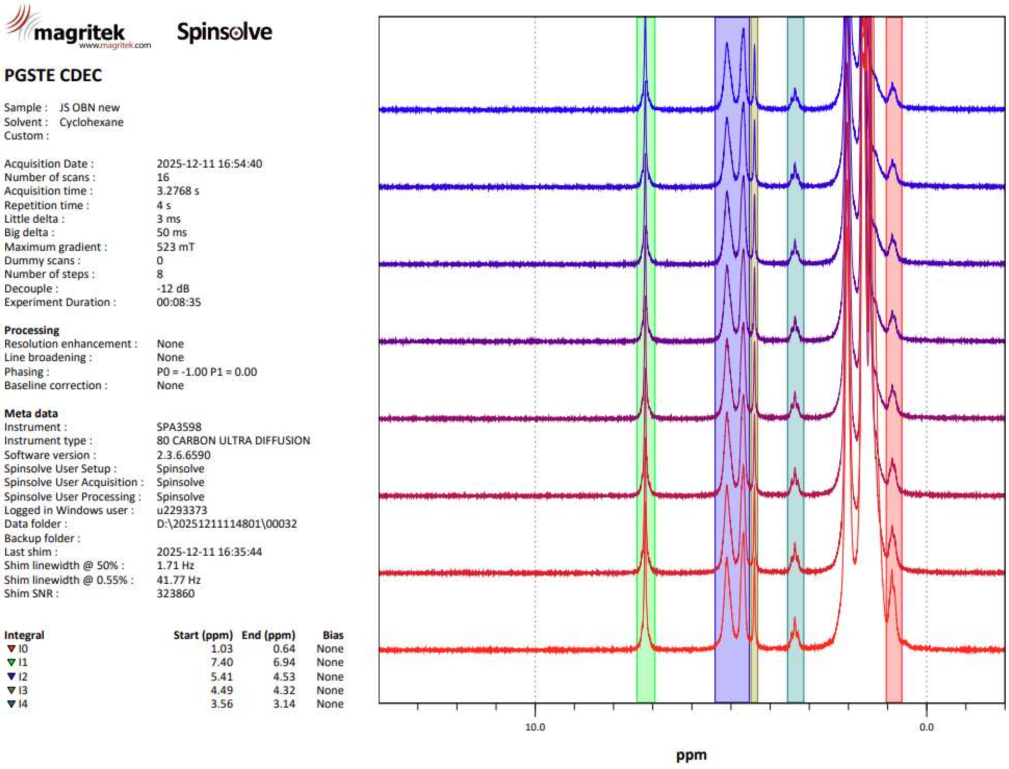

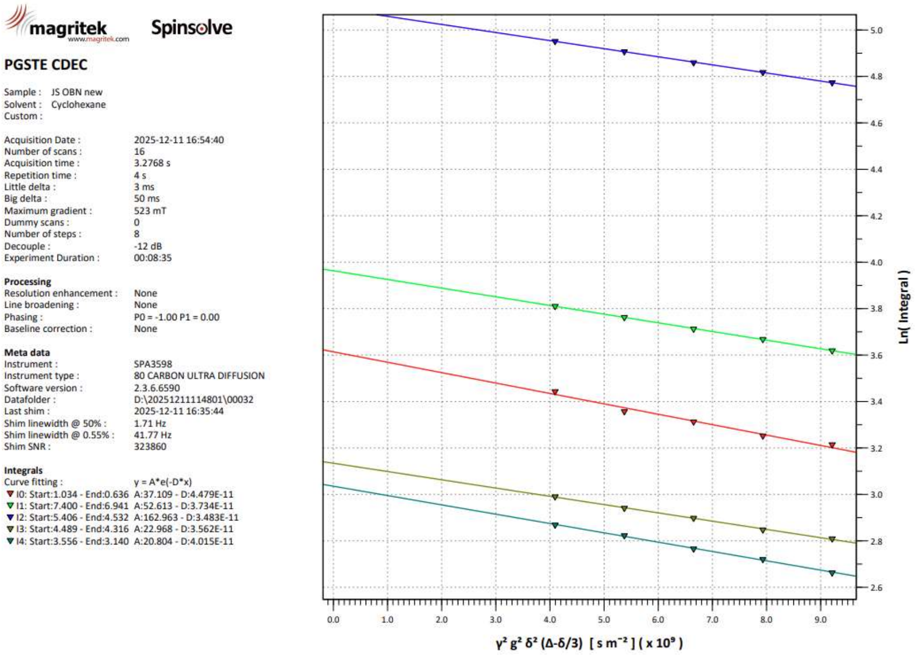

Figure S104: T = 330 Min DOSY Spectrum with diffusion constants calculated from separate regions of the DOSY NMR through Stejskal-Tanner plot. Copolymer of 2 (Bn) and Isoprene (High MWt).

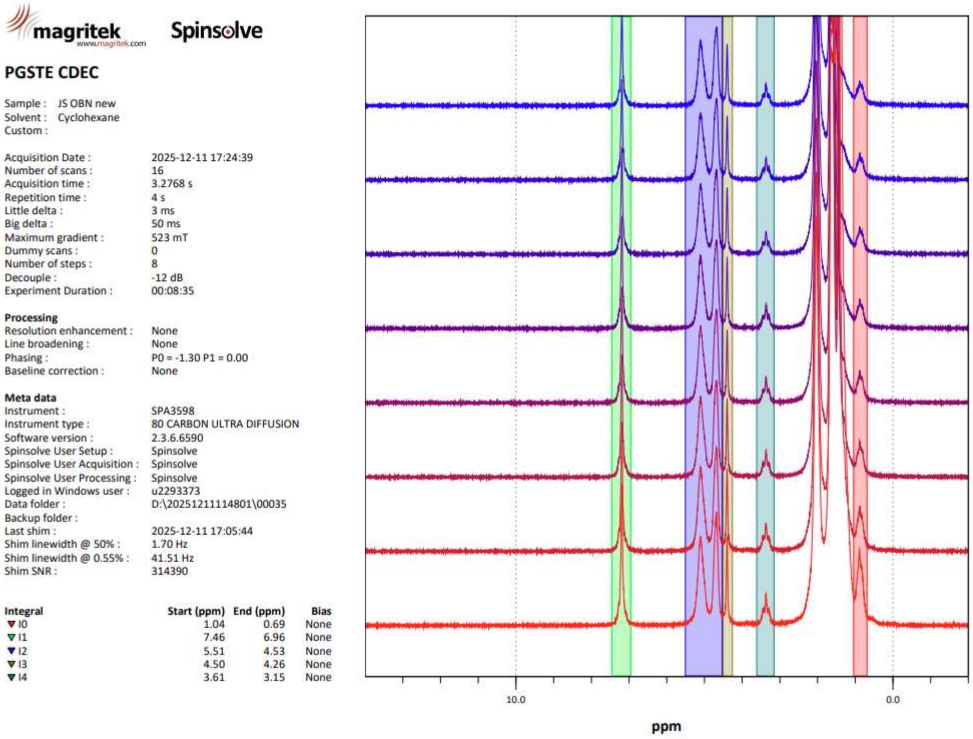

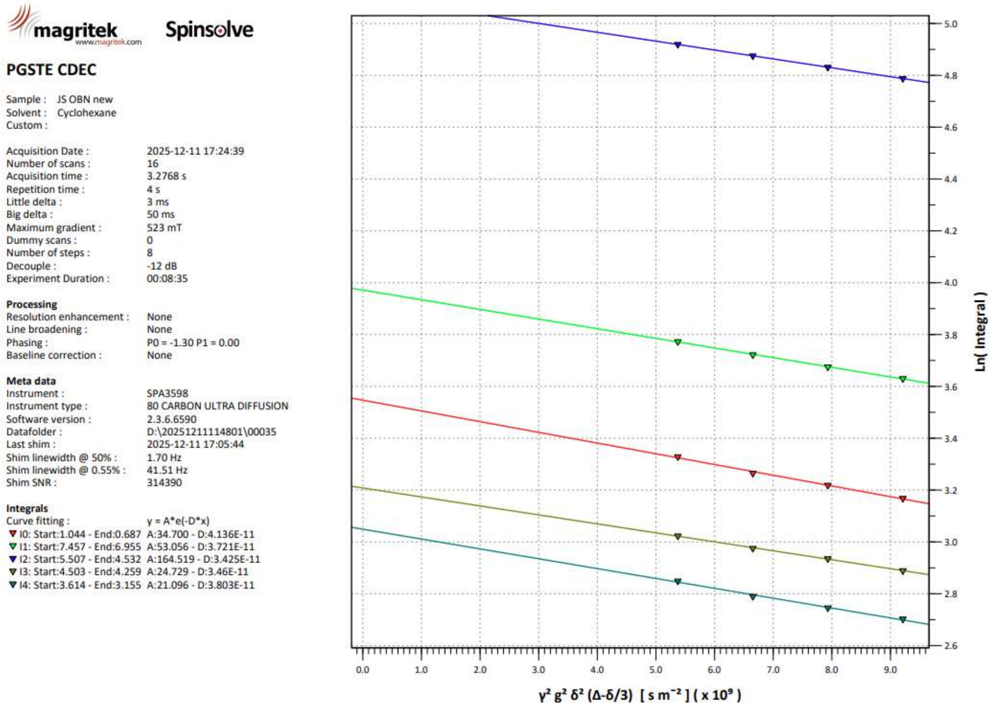

Figure S105: T = 360 Min DOSY Spectrum with diffusion constants calculated from separate regions of the DOSY NMR through Stejskal-Tanner plot. Copolymer of 2 (Bn) and Isoprene (High MWt).

T = 390 Min DOSY Spectrum with diffusion constants calculated from separate regions of the DOSY NMR through Stejskal-Tanner plot. Copolymer of **2** (Bn) and Isoprene (High MWt).

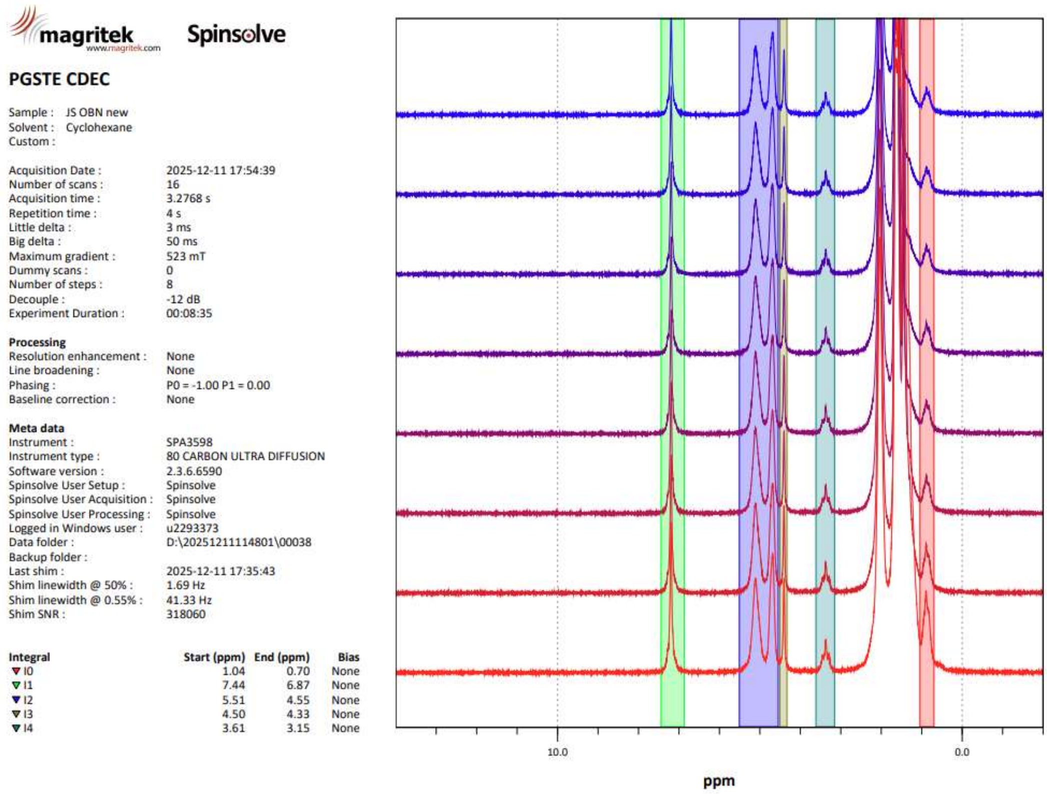

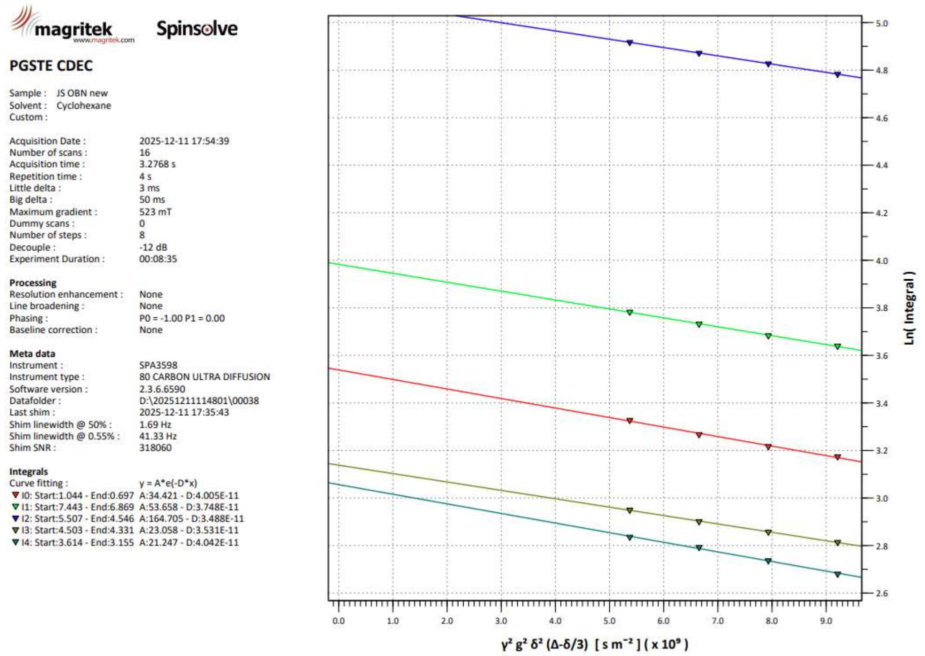

Figure S106: T = 390 Min DOSY Spectrum with diffusion constants calculated from separate regions of the DOSY NMR through Stejskal-Tanner plot. Copolymer of 2 (Bn) and Isoprene (High MWt).

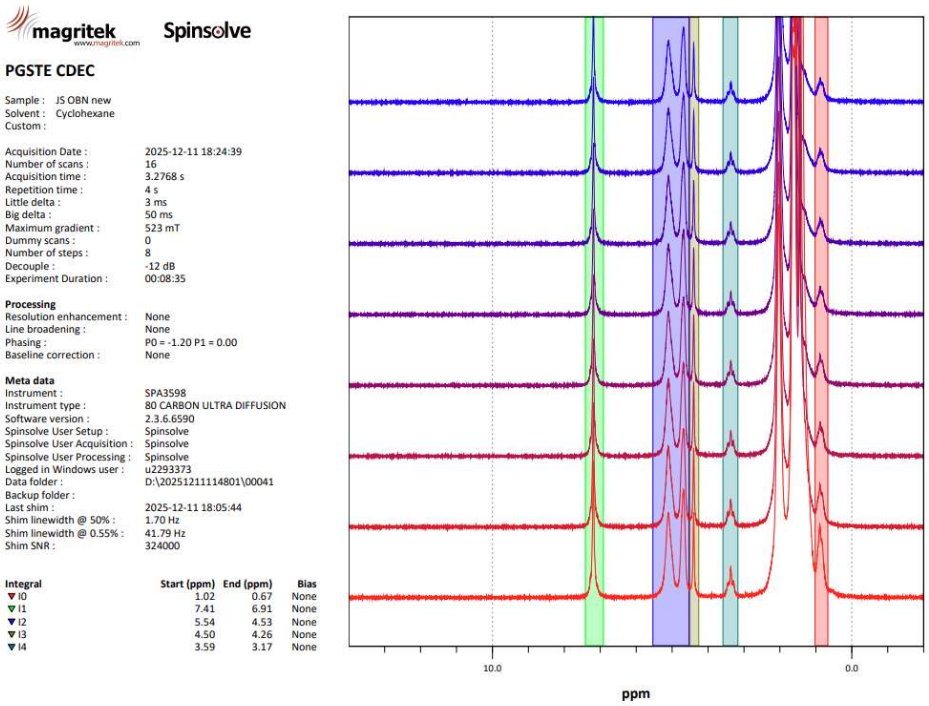

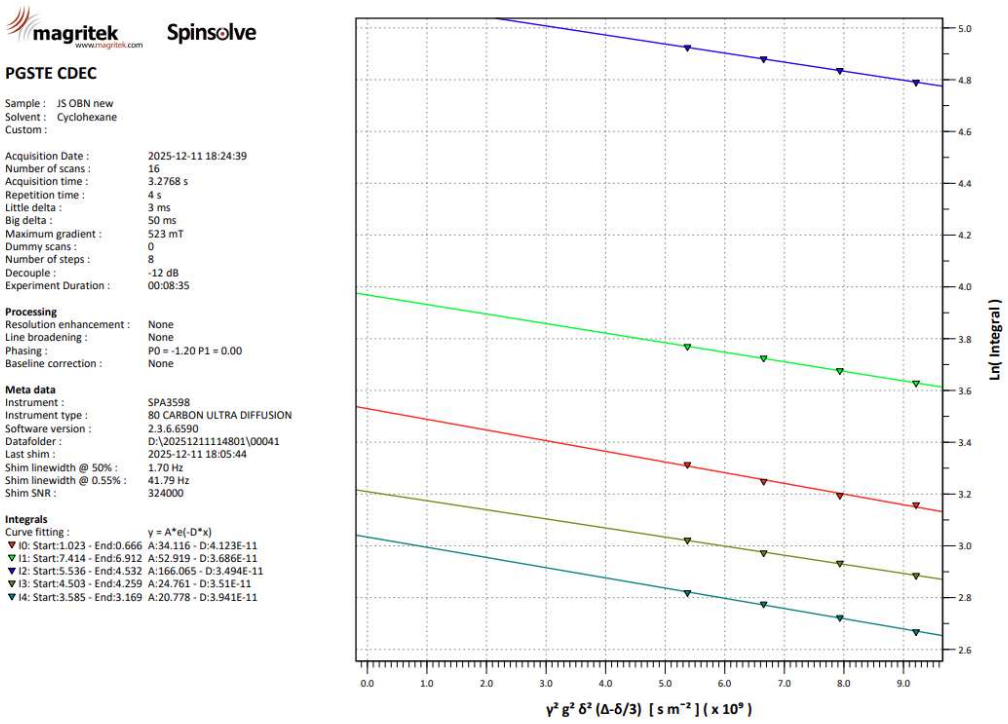

Figure S107: T = 420 Min DOSY Spectrum with diffusion constants calculated from separate regions of the DOSY NMR through Stejskal-Tanner plot. Copolymer of 2 (Bn) and Isoprene (High MWt).

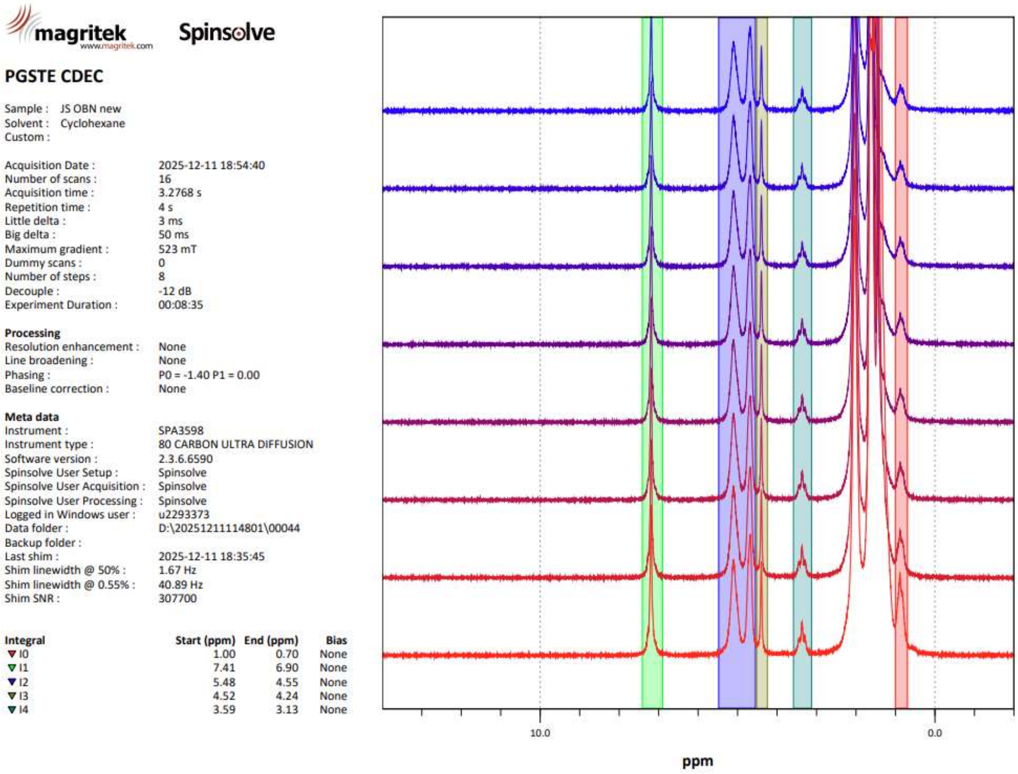

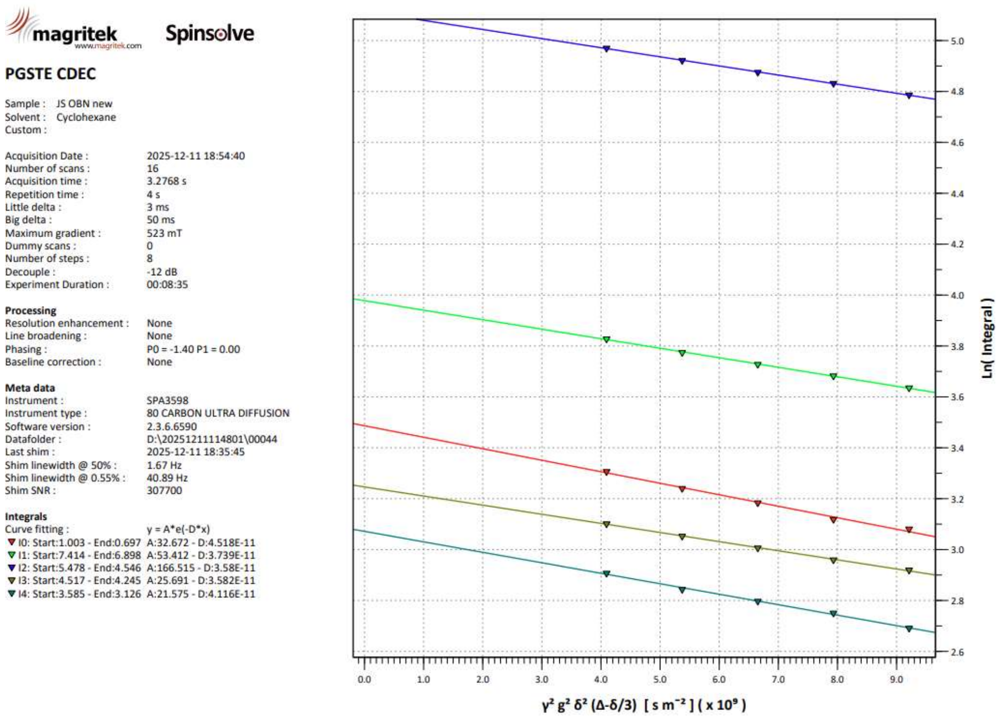

Figure S108: T = 450 Min DOSY Spectrum with diffusion constants calculated from separate regions of the DOSY NMR through Stejskal-Tanner plot. Copolymer of 2 (Bn) and Isoprene (High MWt).

magritek www.magritek.com Spinsolve

1D EXTENDED+

Sample : JS OBN new
Solvent : Cyclohexane
Custom :

Acquisition Date :	2025-12-11 20:50:20
Number of scans :	16
Acquisition time :	6.5536 s
Repetition time :	15 s
Pulse angle :	90 degrees
Experiment Duration :	00:04:03

Processing

Resolution enhancement :	None
Line broadening :	None
Phasing :	P0 = 5.80 P1 = 0.00
Baseline correction :	None

Meta data

Instrument :	SPA3598
Instrument type :	80 CARBON ULTRA DIFFUSION
Software version :	2.3.6.6590
Spinsolve User Setup :	Spinsolve
Spinsolve User Acquisition :	Spinsolve
Spinsolve User Processing :	Spinsolve
Logged in Windows user :	u2293373
Data folder :	D:\20251211114801\00055
Backup folder :	
Last shim :	2025-12-11 20:35:46
Shim linewidth @ 50% :	1.65 Hz
Shim linewidth @ 0.55% :	41.40 Hz
Shim SNR :	318810

Integrals

PPM Range			Normalized	Absolute
3.54	-	3.16	6.91 %	44.86
4.88	-	4.49	33.33 %	216.52
5.41	-	4.89	36.75 %	238.70
7.41	-	6.95	23.02 %	149.52

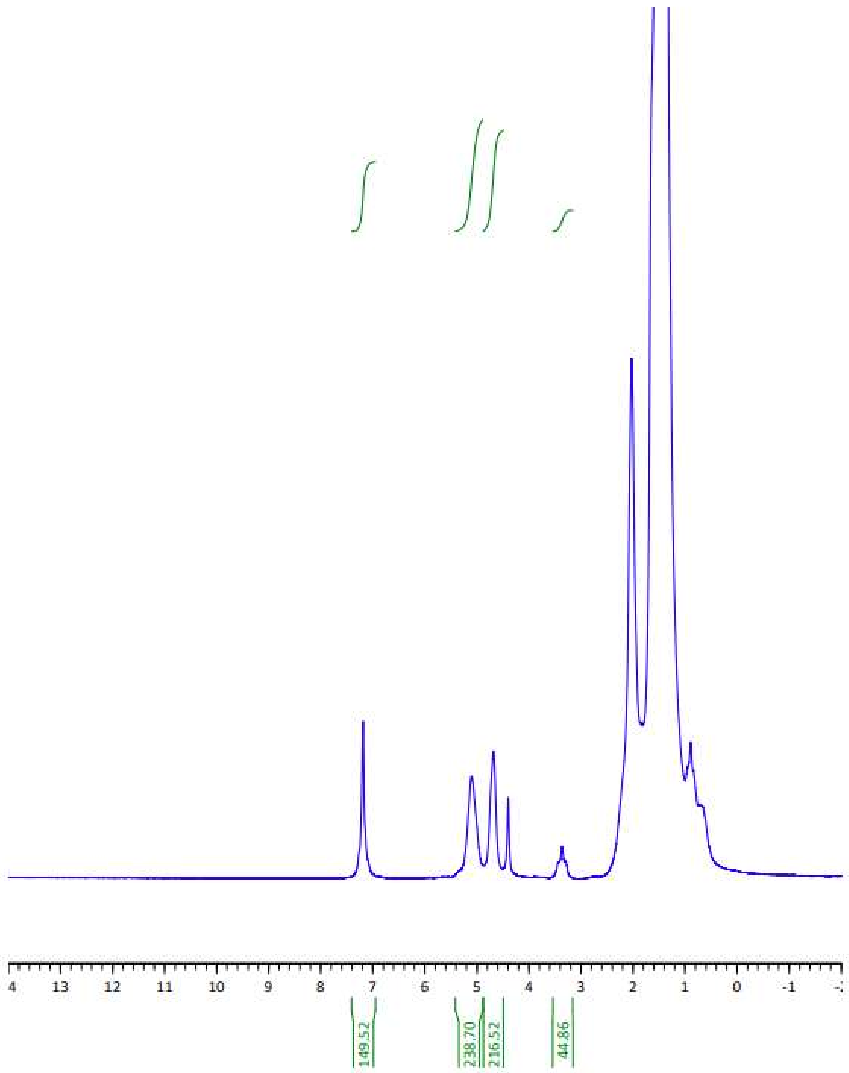

Figure S109: T= Final ^{1}H NMR Spectrum Copolymer of 2 (Bn) and Isoprene (High MWt)

Copolymer of 3 and Isoprene

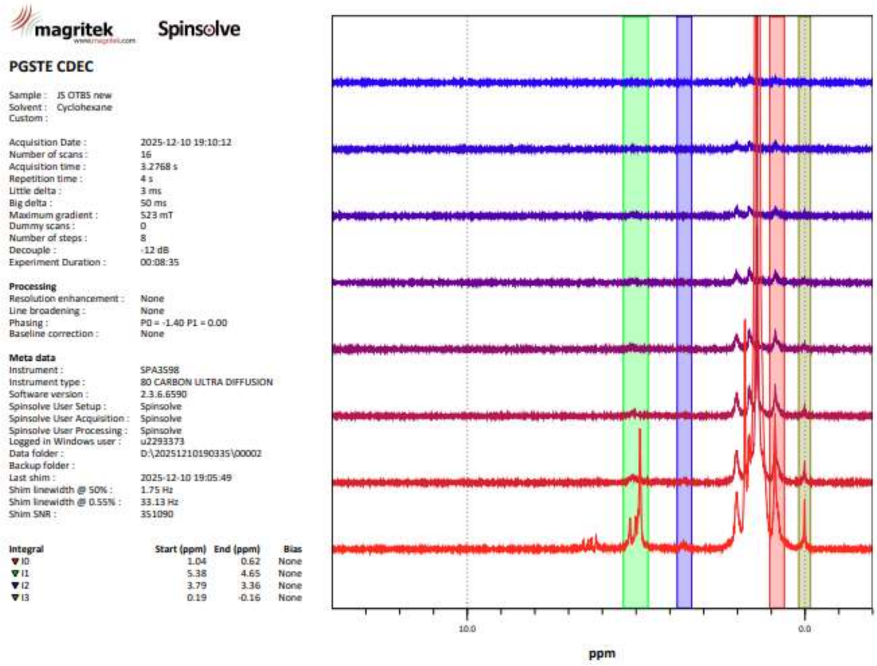

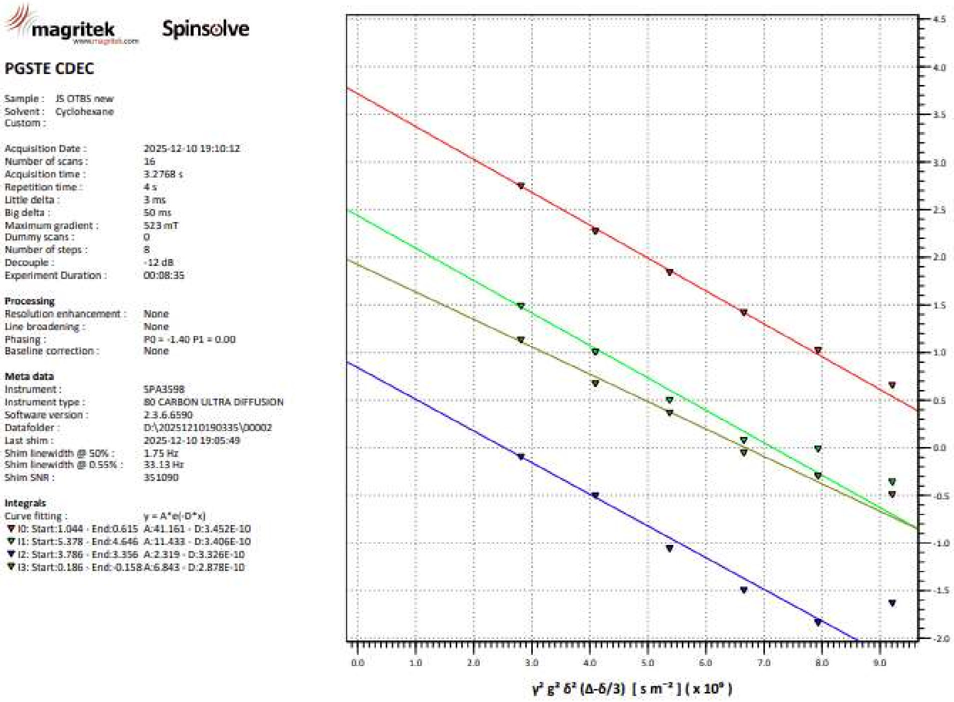

Figure S110: T = 30 mins DOSY Spectrum with diffusion constants calculated from separate regions of the DOSY NMR through Stejskal-Tanner plot. Copolymer of 3 (TBS) and Isoprene.

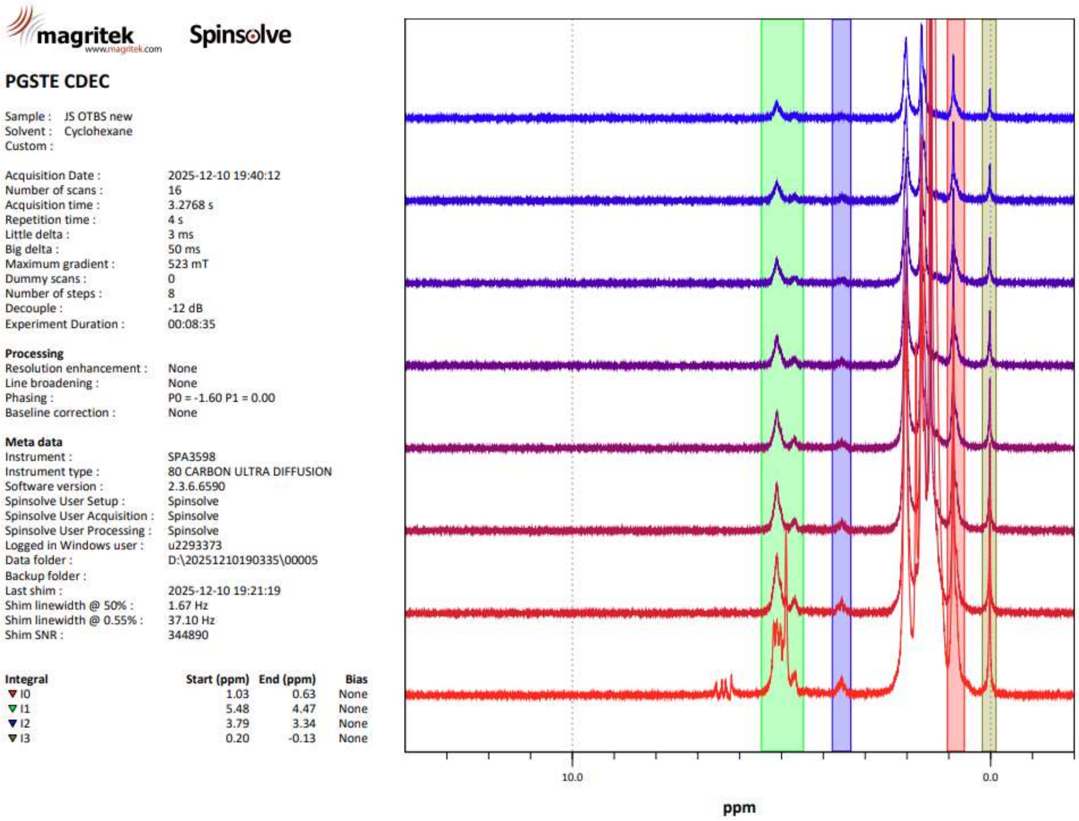

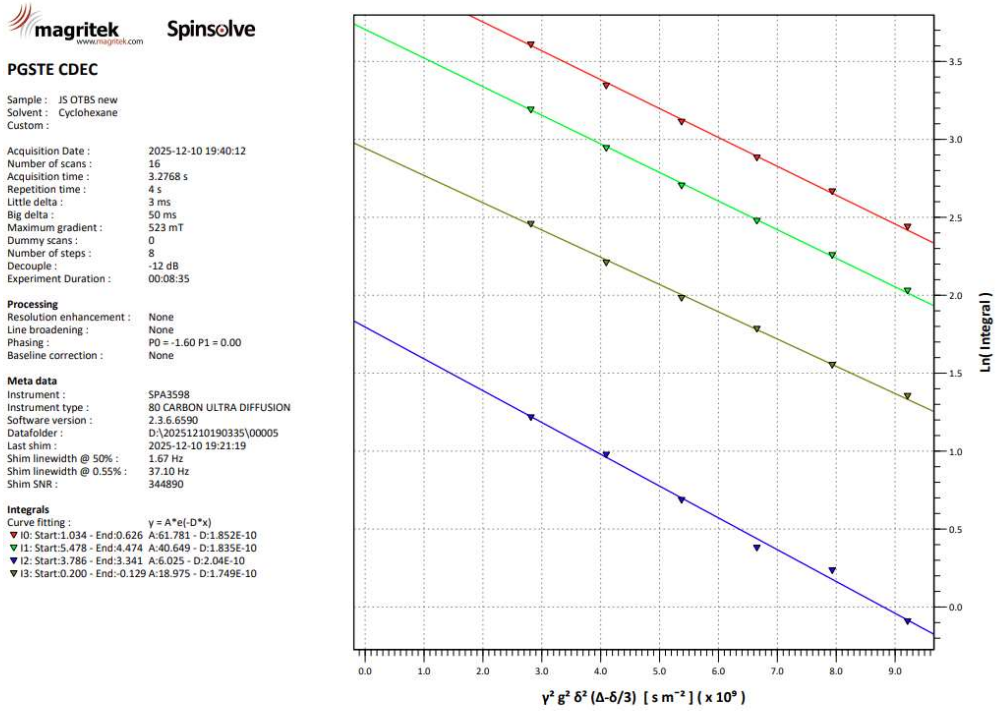

Figure S111: T = 1 hr DOSY Spectrum with diffusion constants calculated from separate regions of the DOSY NMR through Stejskal-Tanner plot. Copolymer of 3 (TBS) and Isoprene.

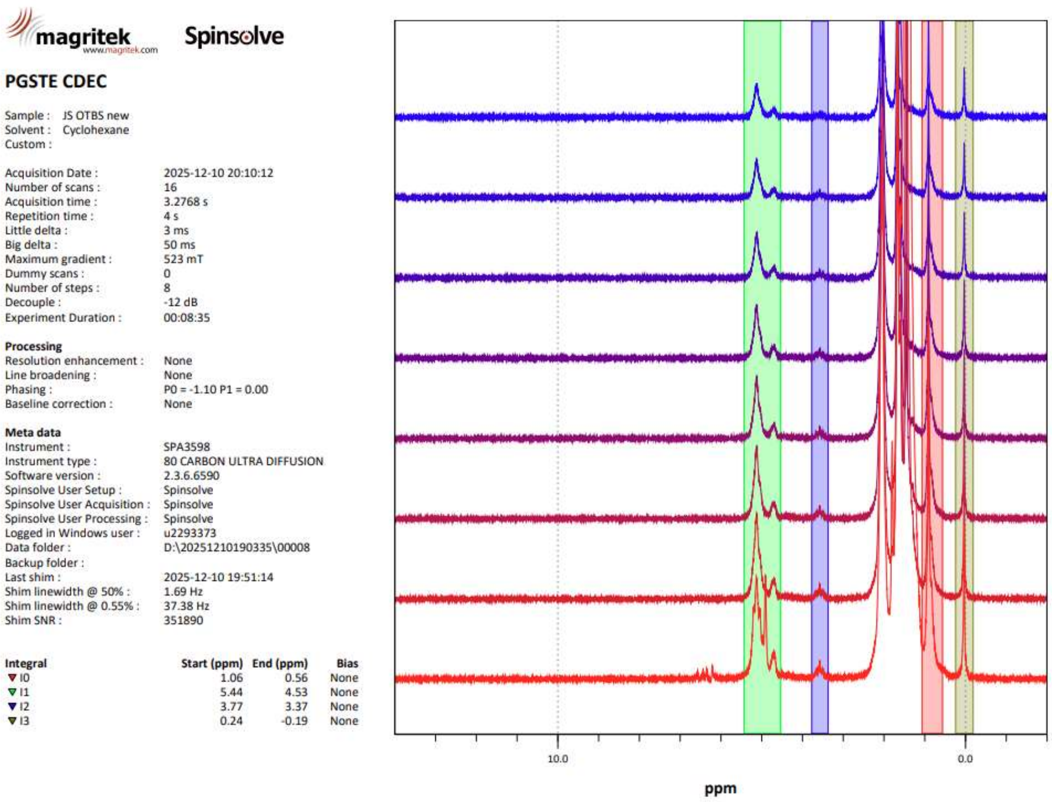

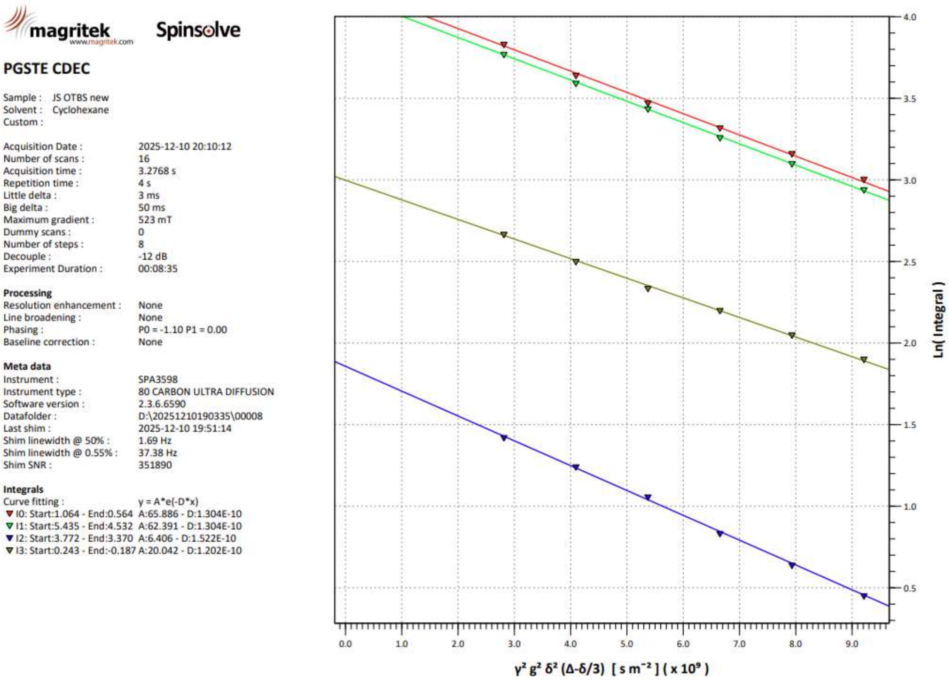

Figure S112: T = 1.5 hr DOSY Spectrum with diffusion constants calculated from separate regions of the DOSY NMR through Stejskal-Tanner plot. Copolymer of 3 (TBS) and Isoprene.

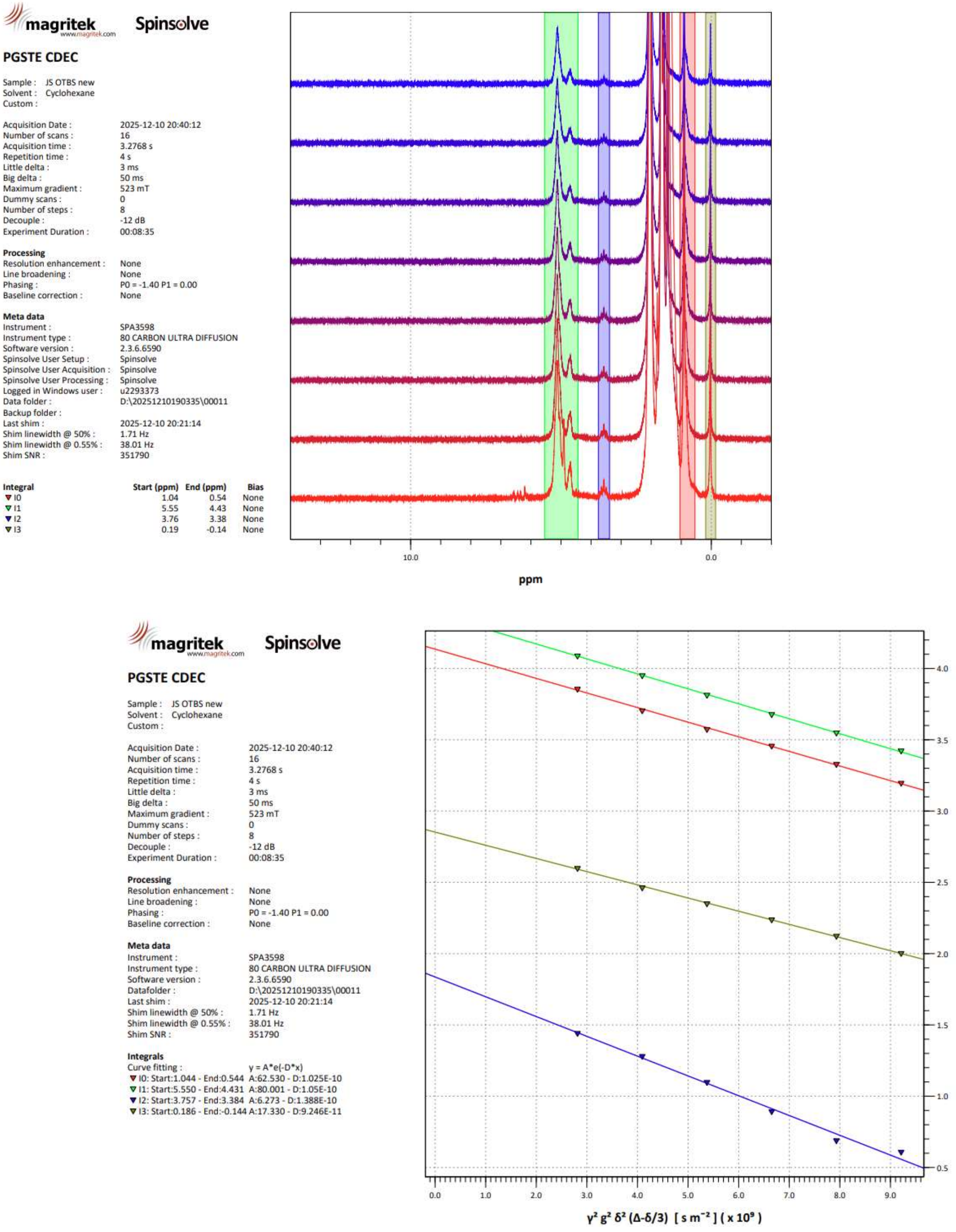

Figure S113: T = 2 hr DOSY Spectrum with diffusion constants calculated from separate regions of the DOSY NMR through Stejskal-Tanner plot. Copolymer of 3 (TBS) and Isoprene.

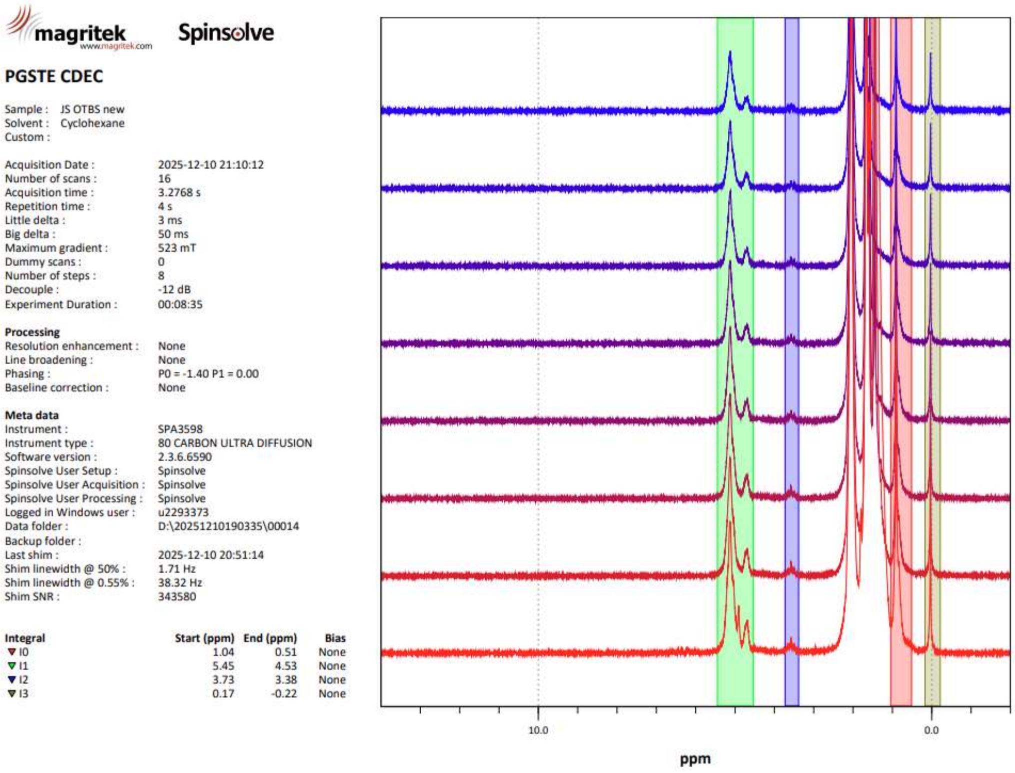

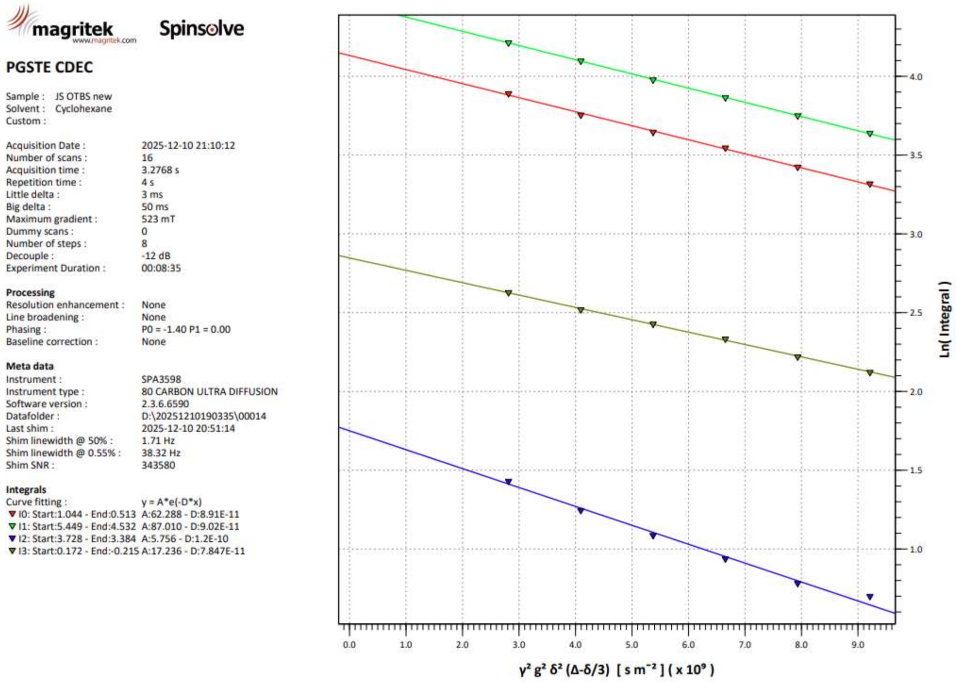

Figure S114: T = 2.5 hr DOSY Spectrum with diffusion constants calculated from separate regions of the DOSY NMR through Stejskal-Tanner plot. Copolymer of 3 (TBS) and Isoprene.

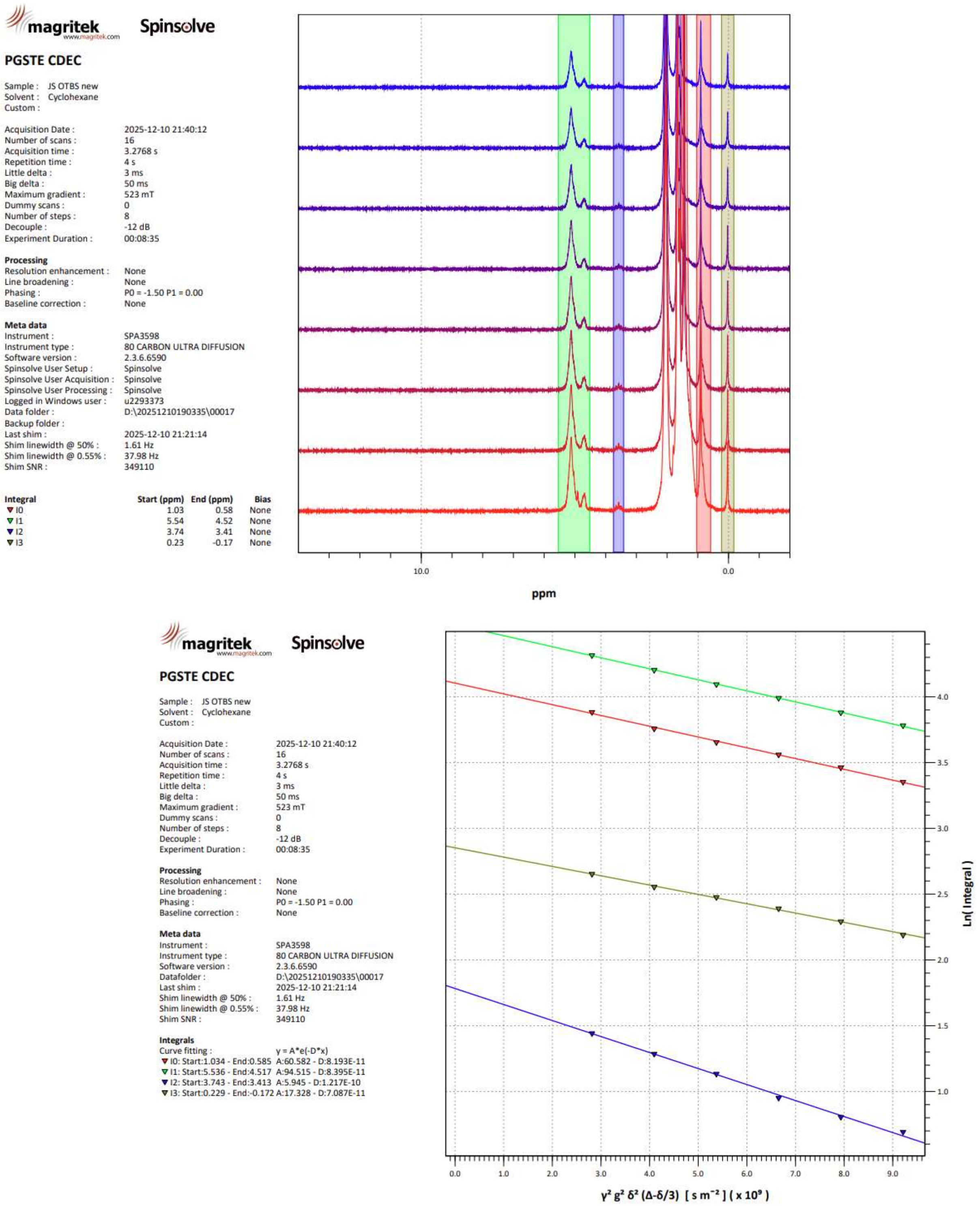

Figure S115: T = 3 hr DOSY Spectrum with diffusion constants calculated from separate regions of the DOSY NMR through Stejskal-Tanner plot. Copolymer of 3 (TBS) and Isoprene.

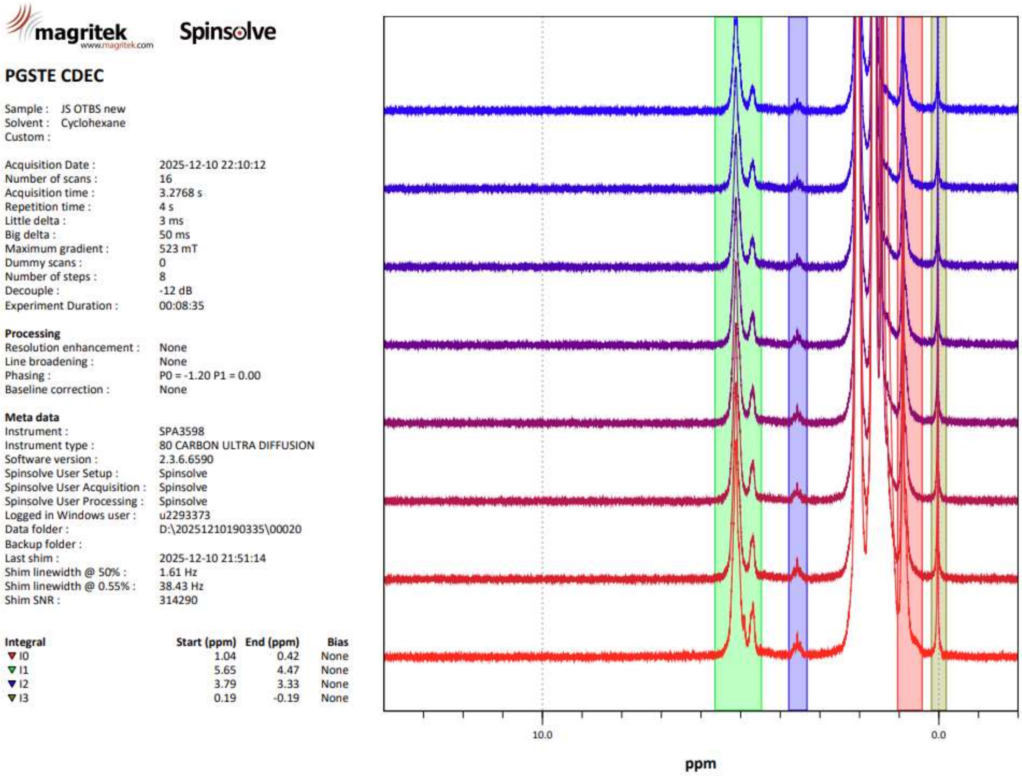

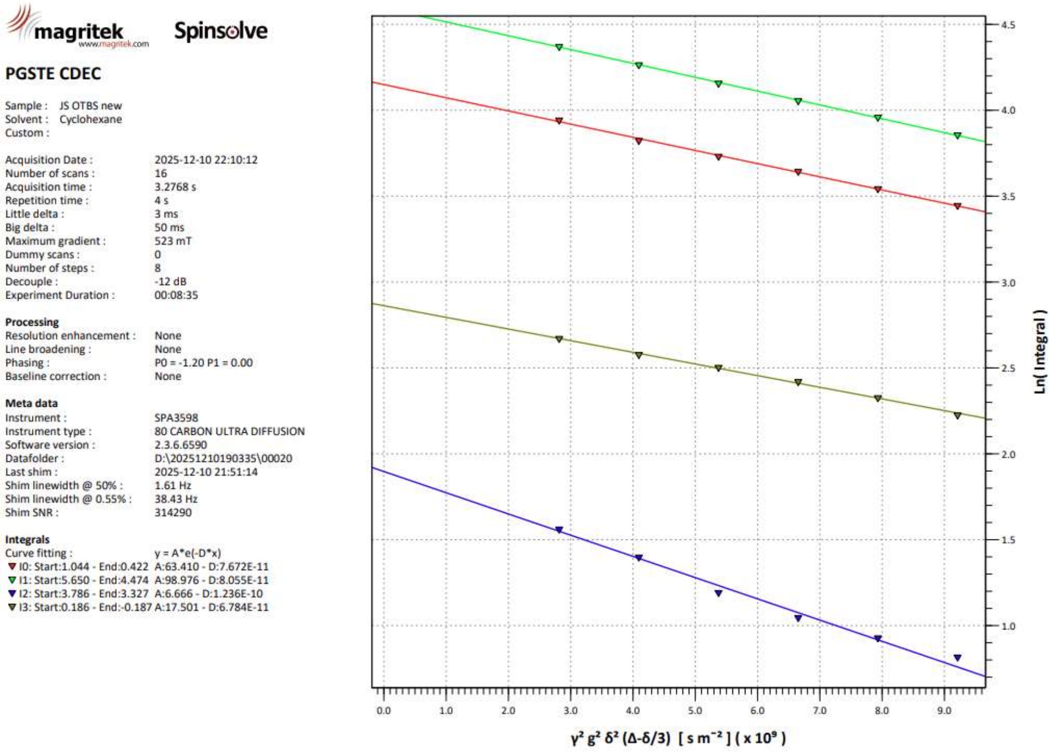

Figure S116: T = 3.5 hr DOSY Spectrum with diffusion constants calculated from separate regions of the DOSY NMR through Stejskal-Tanner plot. Copolymer of 3 (TBS) and Isoprene.

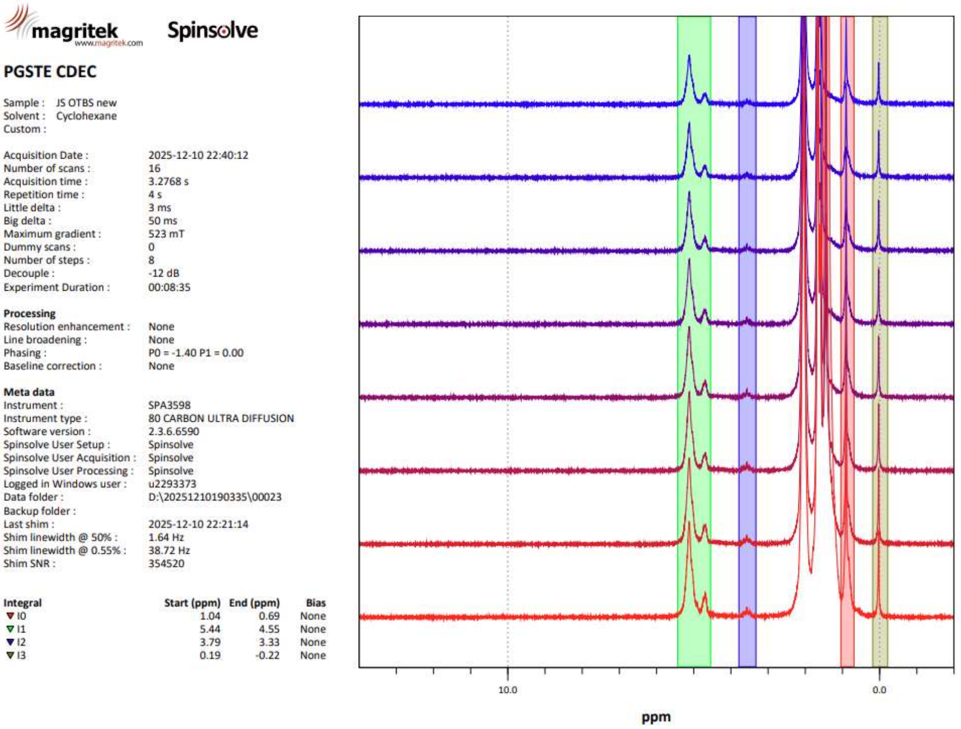

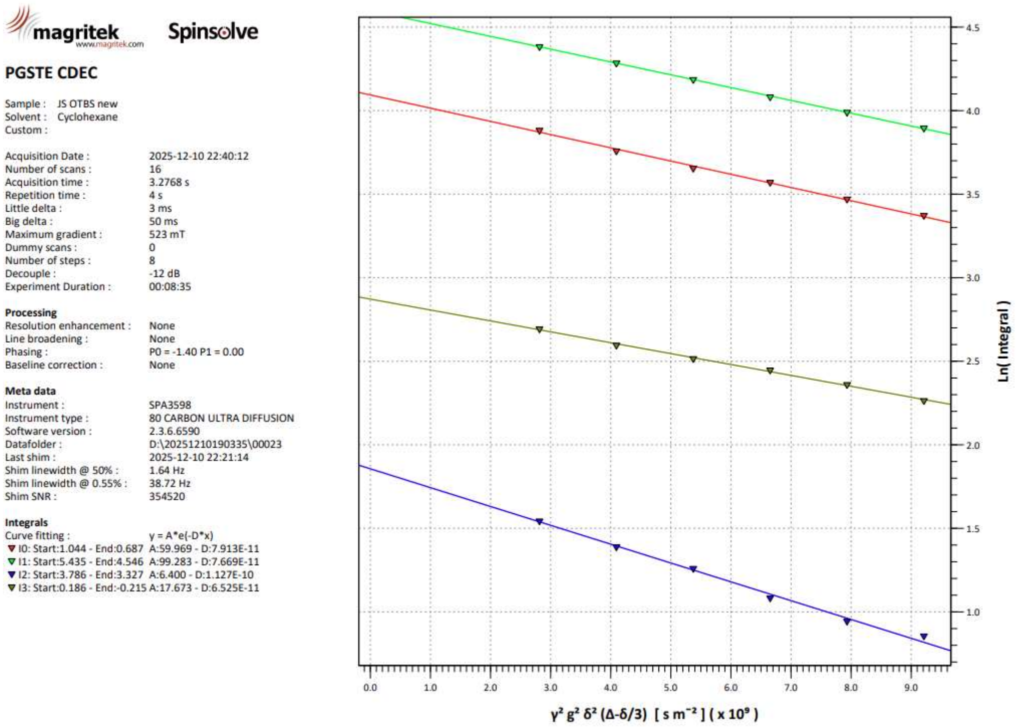

Figure S117: T = 4 hr DOSY Spectrum with diffusion constants calculated from separate regions of the DOSY NMR through Stejskal-Tanner plot. Copolymer of 3 (TBS) and Isoprene.

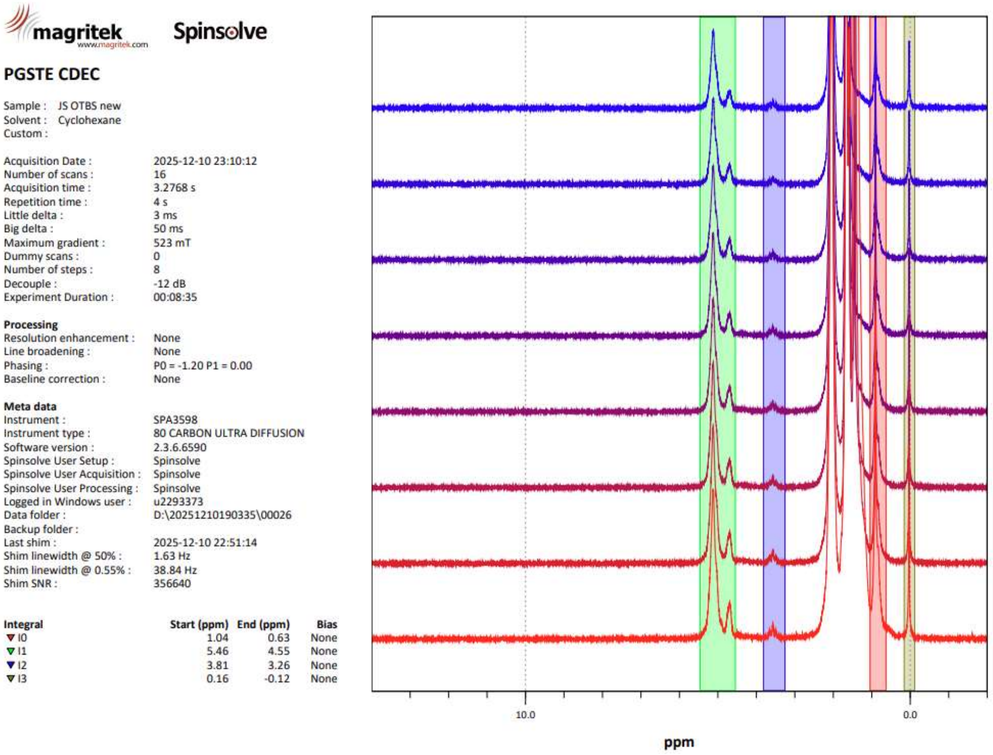

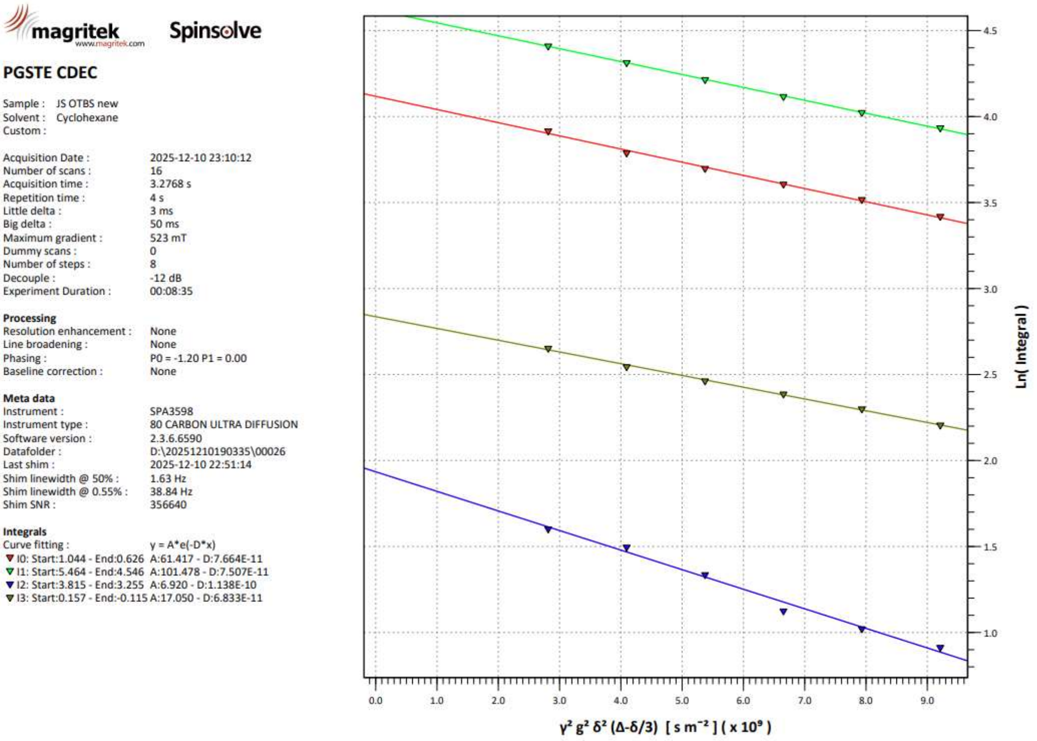

Figure S118: T = 4.5 hr DOSY Spectrum with diffusion constants calculated from separate regions of the DOSY NMR through Stejskal-Tanner plot. Copolymer of 3 (TBS) and Isoprene.

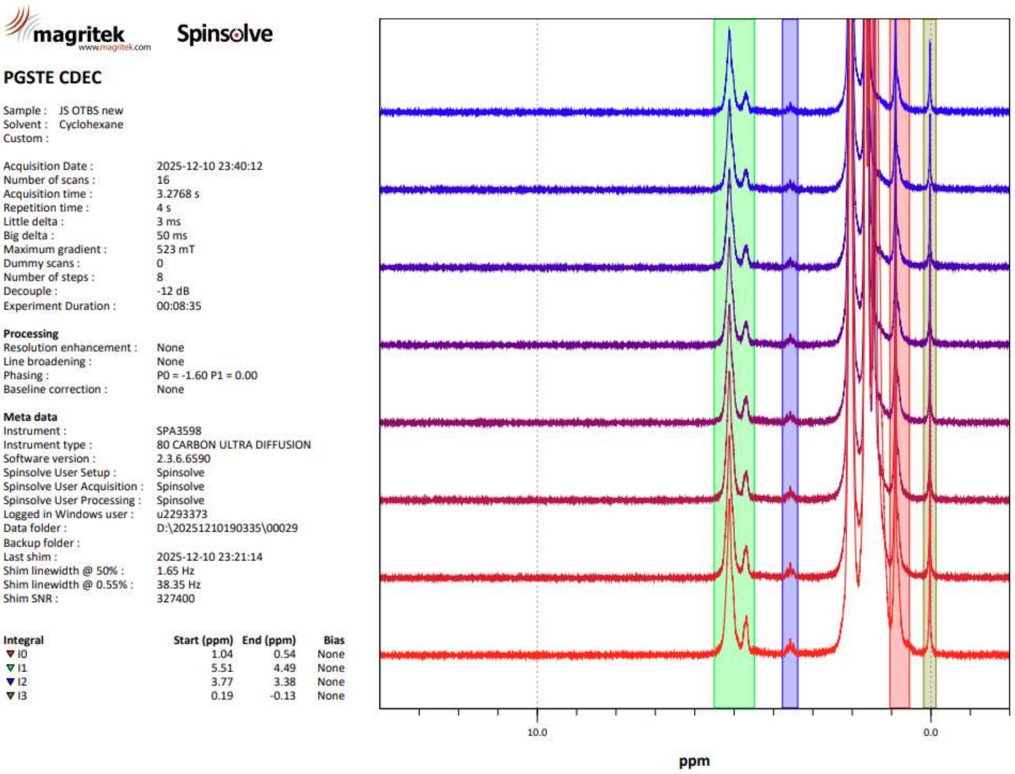

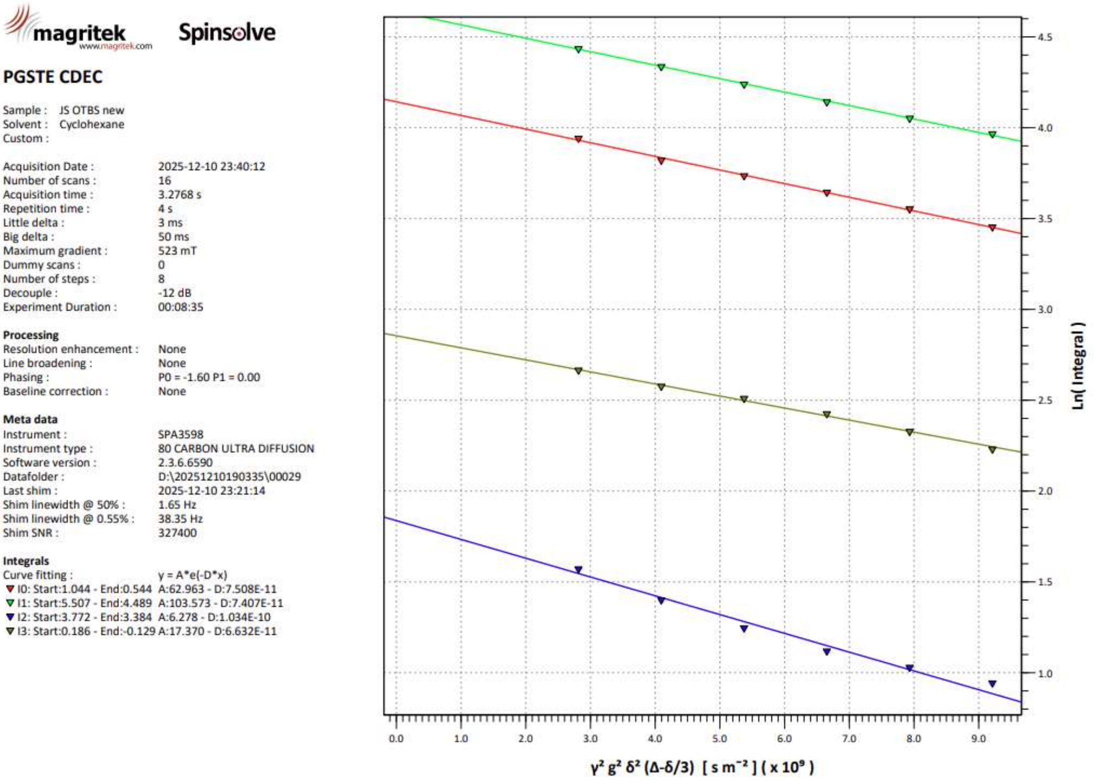

Figure S119: T = 5 hr DOSY Spectrum with diffusion constants calculated from separate regions of the DOSY NMR through Stejskal-Tanner plot. Copolymer of 3 (TBS) and Isoprene.

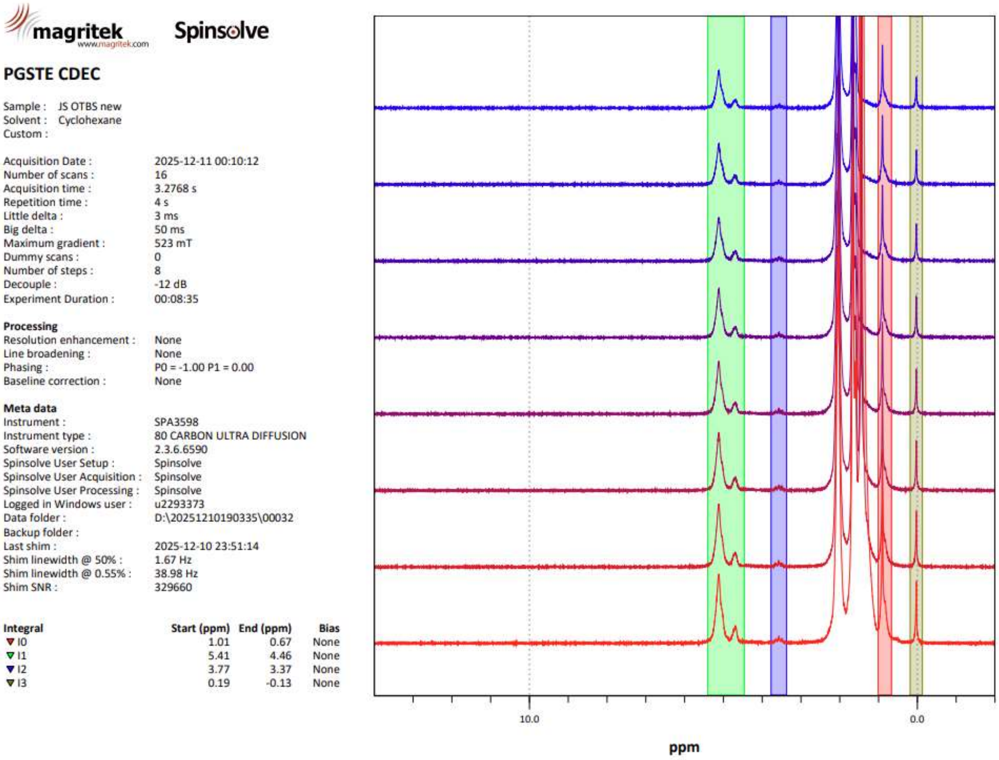

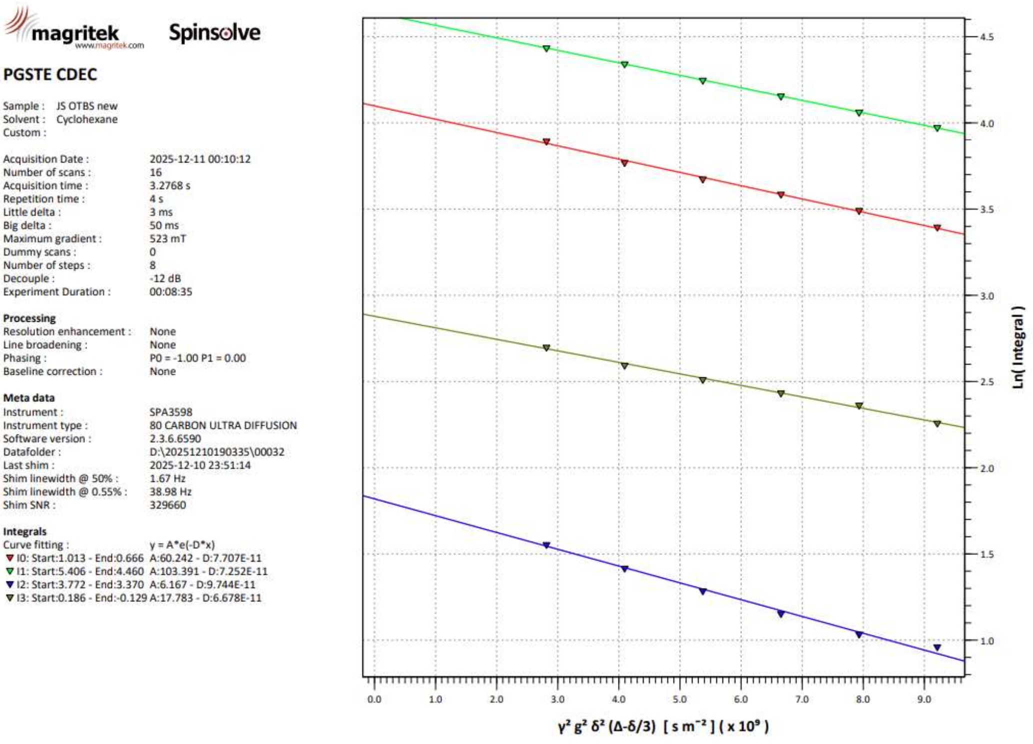

Figure S120: T = 5.5 hr DOSY Spectrum with diffusion constants calculated from separate regions of the DOSY NMR through Stejskal-Tanner plot. Copolymer of 3 (TBS) and Isoprene.

magritek www.magritek.com Spinsolve

1D EXTENDED+

Sample : JS OTBS new
Solvent : Cyclohexane
Custom :

Acquisition Date :	2025-12-11 09:05:53
Number of scans :	16
Acquisition time :	6.5536 s
Repetition time :	15 s
Pulse angle :	90 degrees
Experiment Duration :	00:04:03

Processing

Resolution enhancement :	None
Line broadening :	Exponential = 0.2 Hz<br>Gaussian = 0 Hz
Phasing :	P0 = 5.20 P1 = 0.00
Baseline correction :	Applied

Meta data

Instrument :	SPA3598
Instrument type :	80 CARBON ULTRA DIFFUSION
Software version :	2.3.6.6590
Spinsolve User Setup :	Spinsolve
Spinsolve User Acquisition :	Spinsolve
Spinsolve User Processing :	Spinsolve
Logged in Windows user :	u2293373
Data folder :	D:\20251210190335\00085
Backup folder :	
Last shim :	2025-12-11 08:51:16
Shim linewidth @ 50% :	1.64 Hz
Shim linewidth @ 0.55% :	38.81 Hz
Shim SNR :	327580

Integrals

PPM Range		Normalized	Absolute
3.71 -	3.44	5.93 %	16.99
4.80 -	4.61	13.28 %	38.08
5.34 -	4.93	80.80 %	231.72

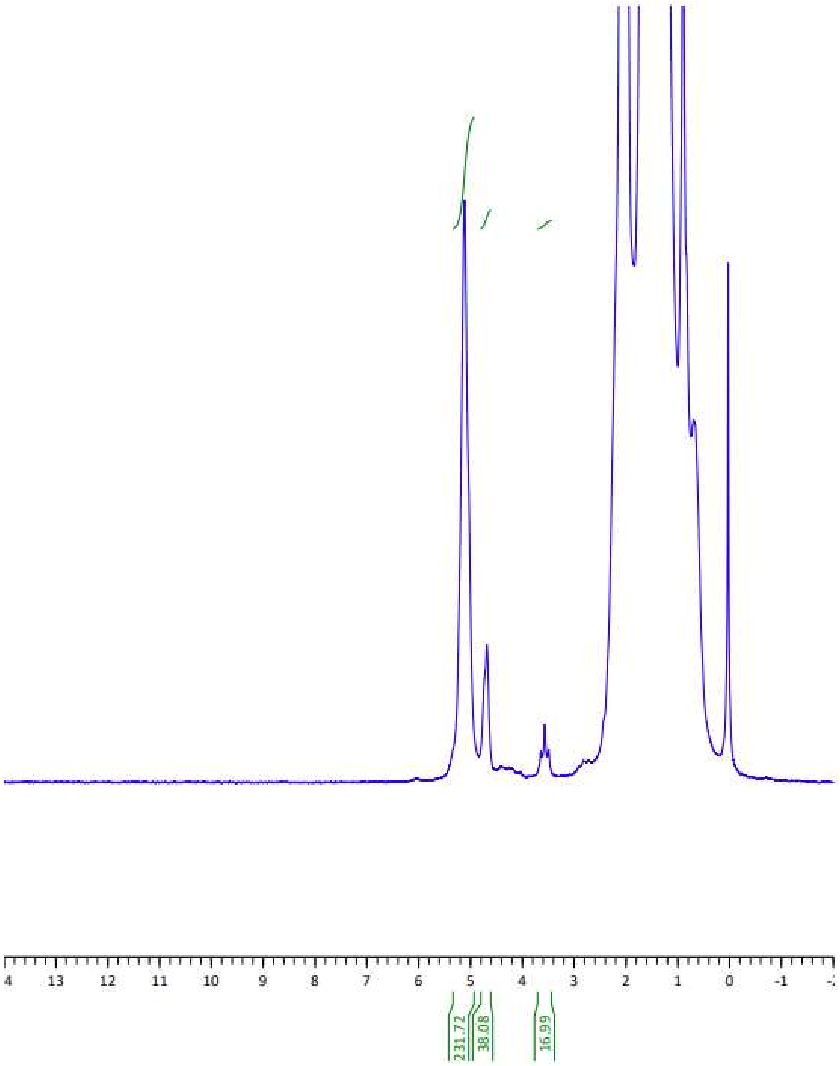

Figure S121: T = Final ^{1}H NMR spectrum Copolymer of 3 (TBS) and Isoprene.

8 TABLE OF QUANTITATIVE MICROSTRUCTURE VALUES FOR POLYMERS FROM TABLE 1.

Table S19: Integration values and calculated microstructure contents of each polymer structure reported.

	Integration Values by NMR (2 s.f.)			Percentage Composition (1 d.p.)		
Polymer ID	1,2	1,4	3,4	1,2	1,4	3,4
HOMO TBS	0.0040	0.38	1.0	0.5 %	42.3 %	57.2 %
HOMO Bn	0.074	0.46	1.0	8.4 %	35.0 %	56.6 %
3,4 PI	1	2.6	6.0	21.8 %	12.8 %	65.3 %
1,4 PI (in hexane)	0	8.6	1.2	0.0 %	93.6 %	6.5 %
3,4-PM	0.16	1.4	1	9.4 %	61.1 %	29.5 %
1,4 PM (in hexane)	0	100	8.6	0.0 %	95.9 %	4.1 %
Copol 1	0.14	0.40	1	17.8 %	16.5 %	65.7 %
Copol 2	1.0	3.1	7.3	17.5 %	18.8 %	63.8 %
Copol 3	1.0	3.0	7.5	17.6 %	16.9 %	65.5 %
Copol 4	0.13	0.38	1.0	17.6 %	15.7 %	66.8 %
Copol 5	0.13	0.37	1.0	18.2 %	13.6 %	68.2 %
Copol 6	0.13	0.36	1.0	17.6 %	14.2 %	68.2 %

9 Diffusion constants obtained from DOSY experiments and their corresponding MWt and error values.

Table S20: OBn/Isoprene (Low MWt) Diffusion constants and corresponding calculated MWt values.

Time (minutes)	Diffusion Constant (D) of Sec-BuLi peak @ 0.8 ppm	MWt (Da)	Error in MWt
30	3.40E-10	945	296
60	2.32E-10	1788	523.5
90	1.95E-10	2404	680
120	1.73E-10	2934	809.5
150	1.59E-10	3384	916.5
180	1.50E-10	3711	993
210	1.33E-10	4555	1185.5
240	1.29E-10	4794	1238.5
270	1.28E-10	4845	1250
300	1.25E-10	5082	1302
330	1.27E-10	4948	1272.5
360	1.24E-10	5102	1306.5
390	1.25E-10	5021	1290

Table S21: OBn/Isoprene (High MWt) Diffusion constants and corresponding calculated MWt values.

Time (minutes)	Diffusion Constant (D) of Sec-BuLi peak @ 0.8 ppm	MWt (Da)	Error in MWt
30	2.87E-10	1252	381
60	1.22E-10	5229	1334
90	7.90E-11	10901	2465
120	5.76E-11	18489	7502
150	5.08E-11	22813	4400.5
180	4.71E-11	25876	4830.5
210	4.38E-11	29256	5278
240	4.24E-11	30929	5490.5
270	4.26E-11	30650	5455.5
300	3.91E-11	35385	6025
330	3.82E-11	36777	6184
360	4.14E-11	32205	5647.5
390	4.01E-11	33990	5862.5

Table S22: OTBS/Isoprene Diffusion constants and corresponding calculated MWt values.

Time (minutes)	Diffusion Constant (D) of Sec-BuLi peak @ 0.8 ppm	MWt (Da)	Error in MWt
30	3.45E-10	920	577
60	1.85E-10	2612	731.5
90	1.30E-10	4702	1218.5
120	1.03E-10	7039	1716
150	8.91E-11	8901	2086.5
180	8.19E-11	10245	2343
210	7.72E-11	11437	2540
240	7.91E-11	11313	2457
270	7.66E-11	11457	2566
300	7.51E-11	11859	2639
330	7.71E-10	11350	2547

10 CONVERSION / TIME PLOTS FOR DOSY SPECTRA.

Methodology:

Using the ^{1}H NMR spectra from the DOSY time course experiments, we calculated the overall conversion of monomer over time. The monomers have a distinct dd at ca 6.6 – 6.1 ppm corresponding to the central CH whilst the other alkene peaks from the monomers overlap with those from the 1,4-polymer (see the diagram below). As the reaction proceeds, the amount of remaining monomer can be measured using the 6.6 – 6.1 ppm peak and 4 x this integral value can be deducted from the 1,4- polymer integral to give the contribution from the polymer alone. Adding this to the of the 3,4- polymer peak integral (divided by 2 to correct for no. Of Hs) gives the total integral per H of the polymer component. This allows the conversions to be calculated by dividing the integral (per H) of the polymer by the total integral per H of the combined monomer and polymer.

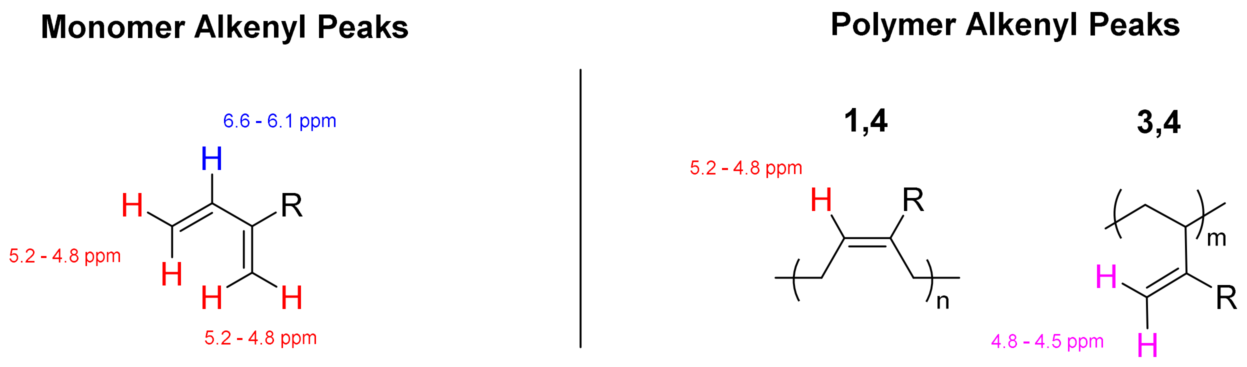

Figure S122: Alkenyl proton environments witnessed in DOSY Conversion.

Tables to Show Integration Values and Conversion Over Time:

Table S23: OBn/Isoprene copolymerization (Low MWt) Integrations and calculated conversion data.

Time (Minutes)	Integral of dd from monomers (1H)	Integral of monomers + 1,4-	Integral of 3,4-polymer (2H)	Integral for 1,4-polymer corrected for presence of monomer (1H)	Integral for 3,4-polymer / 2 (to correct for no. of Hs)	% Conversion = total integral per H for polymer / (integral per H for monomers + integral per H for polymer)
30	94.1	451.7	6.2	75.3	3.1	45.5
60	66.6	359.8	32.4	93.6	16.2	62.3
90	45.1	286.8	53.6	106.6	26.8	74.8
120	30.4	236.7	67.0	115.2	33.5	83.0
150	18.7	200.0	75.1	125.1	37.6	89.7
180	14.2	181.5	82.9	124.6	41.4	92.1
210	7.4	162.6	85.3	133.0	42.7	96.0
240	4.7	154.1	88.7	135.5	44.4	97.5
270	1.1	145.0	89.7	140.4	44.8	99.4
300	0.1	141.5	91.4	141.1	45.7	99.9
330	0.0	140.7	93.0	140.5	46.5	100.0
360	0.9	141.7	94.3	138.1	47.2	99.5
390	1.0	142.4	95.2	138.5	47.6	99.5

Table S24: OBn/Isoprene copolymerization (High MWt) Integrations and calculated conversion data.

Time (Minutes)	Integral of dd from monomers (1H)	Integral of monomers + 1,4-	Integral of 3,4-polymer (2H)	Integral for 1,4-polymer corrected for presence of monomer (1H)	Integral for 3,4-polymer / 2 (to correct for no. of Hs)	% Conversion = total integral per H for polymer / (integral per H for monomers + integral per H for polymer)
30	191.6	879.6	11.6	113.2	5.8	38.3
60	130.3	673.7	88.6	152.3	44.3	60.1
90	79.4	502.8	139.1	185.2	69.5	76.2
120	47.6	401.9	168.1	211.4	84.1	86.1
150	29.1	342.6	184.7	226.3	92.3	91.6
180	17.1	306.6	197.0	238.2	98.5	95.2
210	9.1	283.8	203.6	247.6	101.8	97.5
240	0.3	267.3	206.1	266.2	103.0	99.9
270	0.2	263.6	211.5	262.8	105.7	99.9
300	0.2	260.9	215.2	260.2	107.6	100.0
330	0.5	260.2	218.1	258.4	109.0	99.9
360	0.4	259.0	220.0	257.3	110.0	99.9
390	0.7	258.4	221.0	255.8	110.5	99.8
420	0.4	257.6	221.9	256.1	110.9	99.9
450	0.1	258.0	222.5	257.5	111.2	100.0

Table S25: OTBS/Isoprene copolymerization Integrations and calculated conversion data.

Time (Minutes)	Integral of dd from monomers (1H)	Integral of monomers + 1,4	Integral of 3,4-polymer (2H)	Integral for 1,4-polymer corrected for presence of monomer (1H)	Integral for 3,4-polymer / 2 (to correct for no. of Hs)	% Conversion = total integral per H for polymer / (integral per H for monomers + integral per H for polymer)
30	131.6	585.3	1.7	58.9	0.9	31.2
60	77.0	432.2	10.9	124.2	5.5	62.7
90	46.6	356.6	21.0	170.3	10.5	79.5
120	27.7	314.2	26.2	203.4	13.1	88.7
150	15.1	283.4	28.7	223.2	14.4	94.0
180	6.5	264.8	30.6	239.0	15.3	97.5
210	1.1	251.9	31.7	247.7	15.9	99.6
240	0.4	246.2	34.1	244.6	17.0	99.8
270	1.1	246.5	37.3	242.1	18.6	99.6
300	0.5	244.9	38.5	243.1	19.2	99.8
330	0.5	244.9	39.9	242.9	20.0	99.8

OBn / Isoprene copolymerization (Low MWt) ^{1}H NMR Spectra:

30 Minutes:

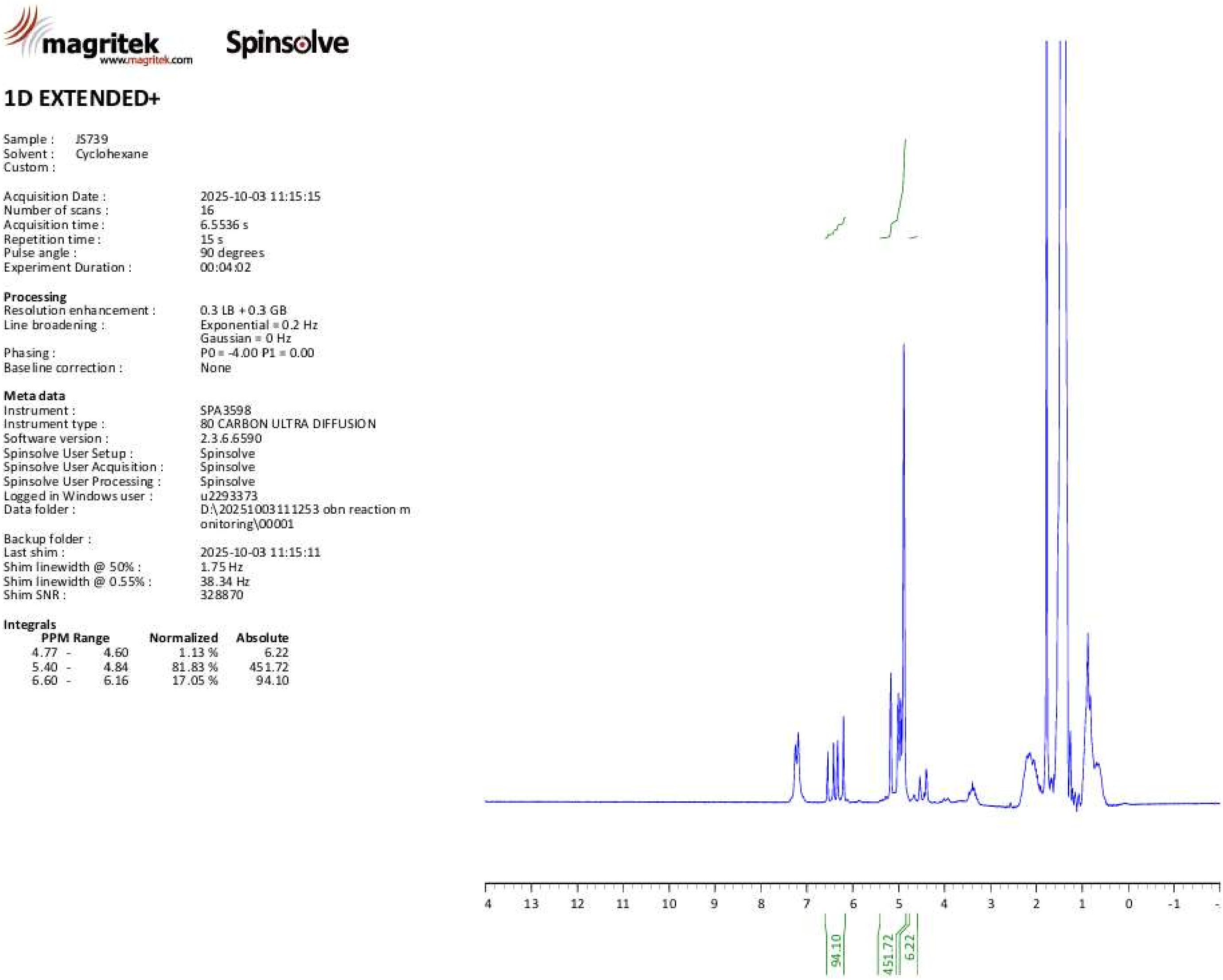

Figure S123: 30 Minutes OBn / Isoprene copolymerization (Low MWt) ^{1}H NMR Spectra

OBn / Isoprene copolymerization (Low MWt) ^1H NMR Spectra:

60 Minutes:

magritek www.magritek.com Spinsolve

1D EXTENDED+

Sample :	JS739
Solvent :	Cyclohexane
Custom :	

Acquisition Date :	2025-10-03 11:45:15
Number of scans :	16
Acquisition time :	6.5536 s
Repetition time :	15 s
Pulse angle :	90 degrees
Experiment Duration :	00:04:02

Processing

Resolution enhancement :	0.3 LB + 0.3 GB
Line broadening :	Exponential = 0.2 Hz<br>Gaussian = 0 Hz
Phasing :	P0 = 5.00 P1 = 0.00
Baseline correction :	None

Meta data

Instrument :	SPA3598
Instrument type :	80 CARBON ULTRA DIFFUSION
Software version :	2.3.6.6590
Spinsolve User Setup :	Spinsolve
Spinsolve User Acquisition :	Spinsolve
Spinsolve User Processing :	Spinsolve
Logged in Windows user :	u2293373
Data folder :	D:\20251003111253 obn reaction monitoring\00004
Backup folder :	
Last shim :	2025-10-03 11:30:36
Shim linewidth @ 50% :	1.55 Hz
Shim linewidth @ 0.55% :	37.29 Hz
Shim SNR :	317580

Integrals

PPM Range		Normalized	Absolute
4.77 -	4.60	7.07 %	32.43
5.40 -	4.84	78.43 %	359.83
6.60 -	6.16	14.50 %	66.55

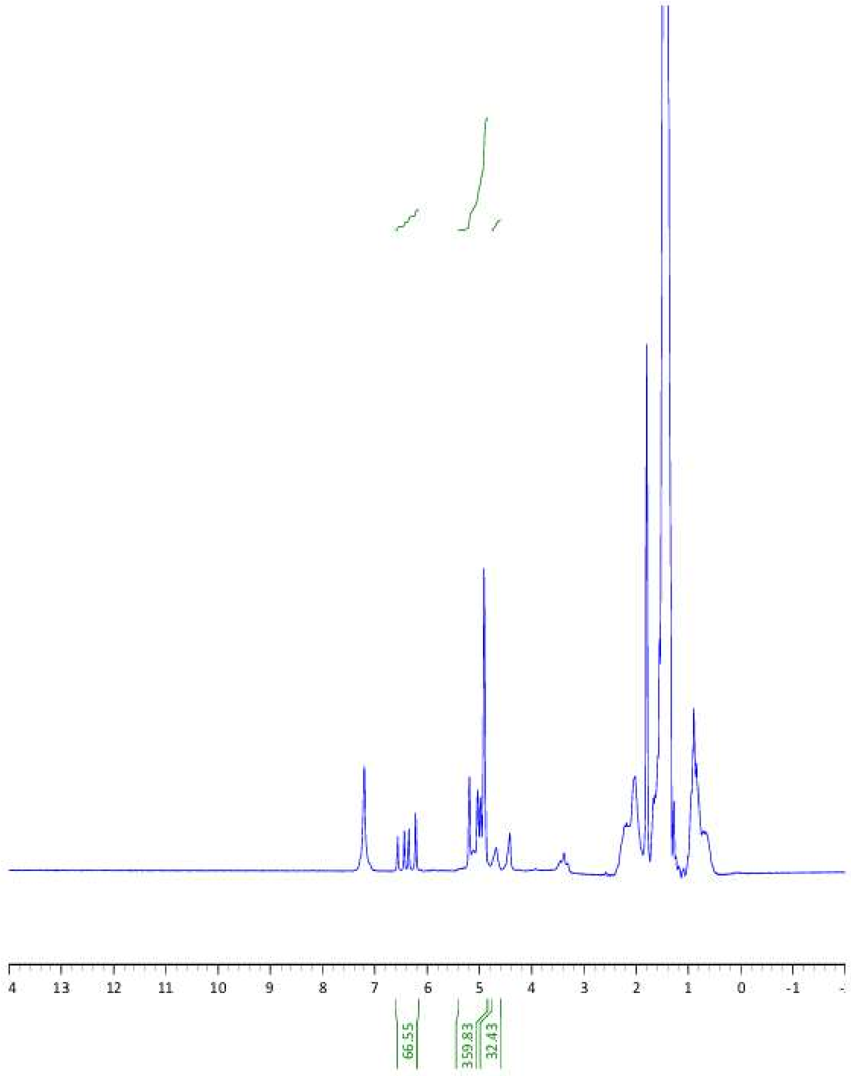

Figure S124: 60 Minutes OBn / Isoprene copolymerization (Low MWt) ^1H NMR Spectra

OBn / Isoprene copolymerization (Low MWt) ^{1}H NMR Spectra:

90 Minutes:

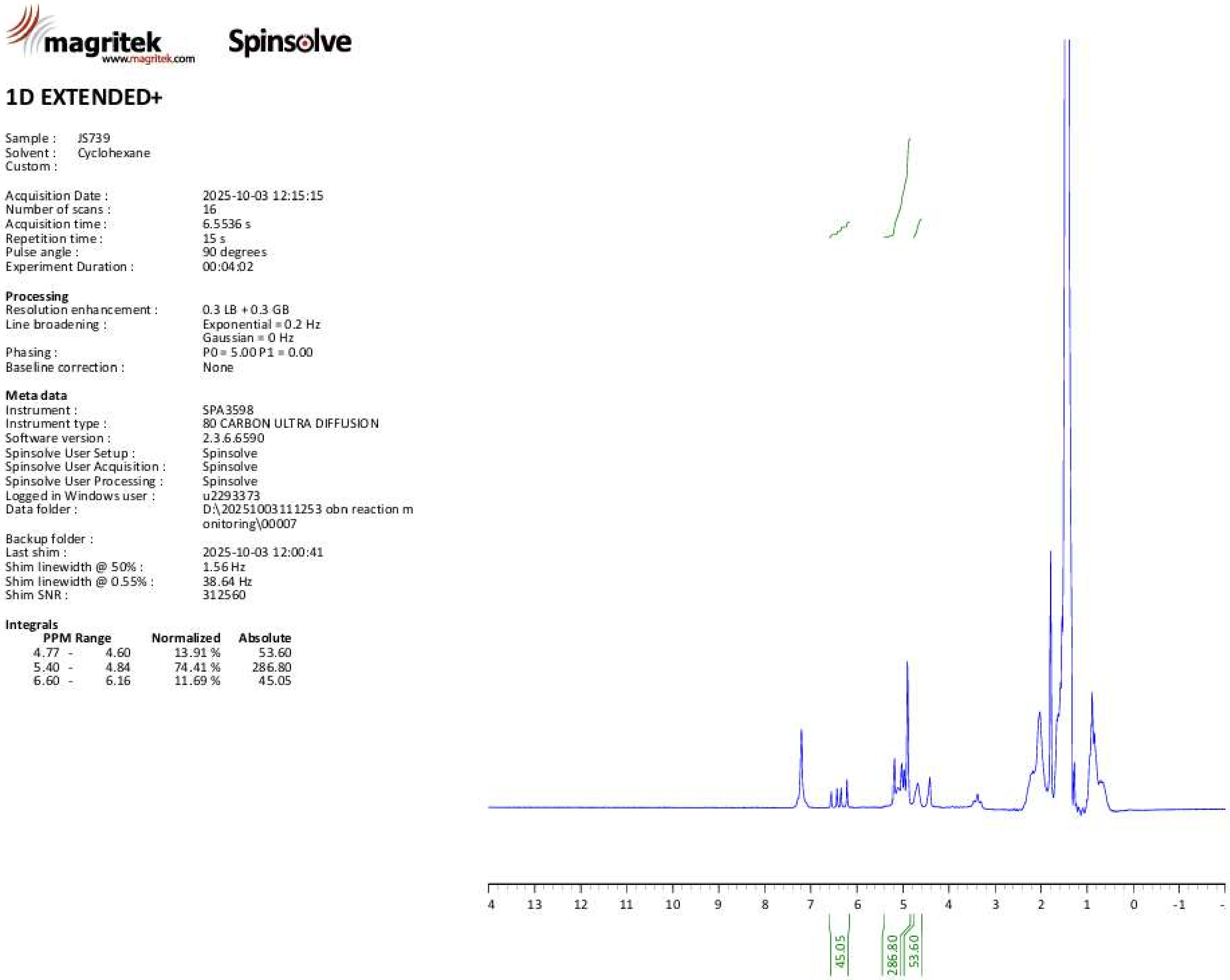

Figure S125: 90 Minutes OBn / Isoprene copolymerization (Low MWt) ^{1}H NMR Spectra

OBn / Isoprene copolymerization (Low MWt) ^{1}H NMR Spectra:

120 Minutes:

magritek www.magritek.com Spinsolve

1D EXTENDED+

Sample : JS739
Solvent : Cyclohexane
Custom :

Acquisition Date : 2025-10-03 12:45:15
Number of scans : 16
Acquisition time : 6.5536 s
Repetition time : 15 s
Pulse angle : 90 degrees
Experiment Duration : 00:04:02

Processing
Resolution enhancement : 0.3 LB + 0.3 GB
Line broadening : Exponential = 0.2 Hz
Gaussian = 0 Hz
Phasing : P0 = 5.00 P1 = 0.00
Baseline correction : None

Meta data
Instrument : SPA3598
Instrument type : 80 CARBON ULTRA DIFFUSION
Software version : 2.3.6.6590
Spinsolve User Setup : Spinsolve
Spinsolve User Acquisition : Spinsolve
Spinsolve User Processing : Spinsolve
Logged in Windows user : u2293373
Data folder : D:\20251003111253 obn reaction monitoring\00010
Backup folder :
Last shim : 2025-10-03 12:30:36
Shim linewidth @ 50% : 1.58 Hz
Shim linewidth @ 0.55% : 39.33 Hz
Shim SNR : 352210

Integrals

PPM Range			Normalized	Absolute
4.77	-	4.60	20.05 %	67.00
5.40	-	4.84	70.85 %	236.70
6.60	-	6.16	9.09 %	30.38

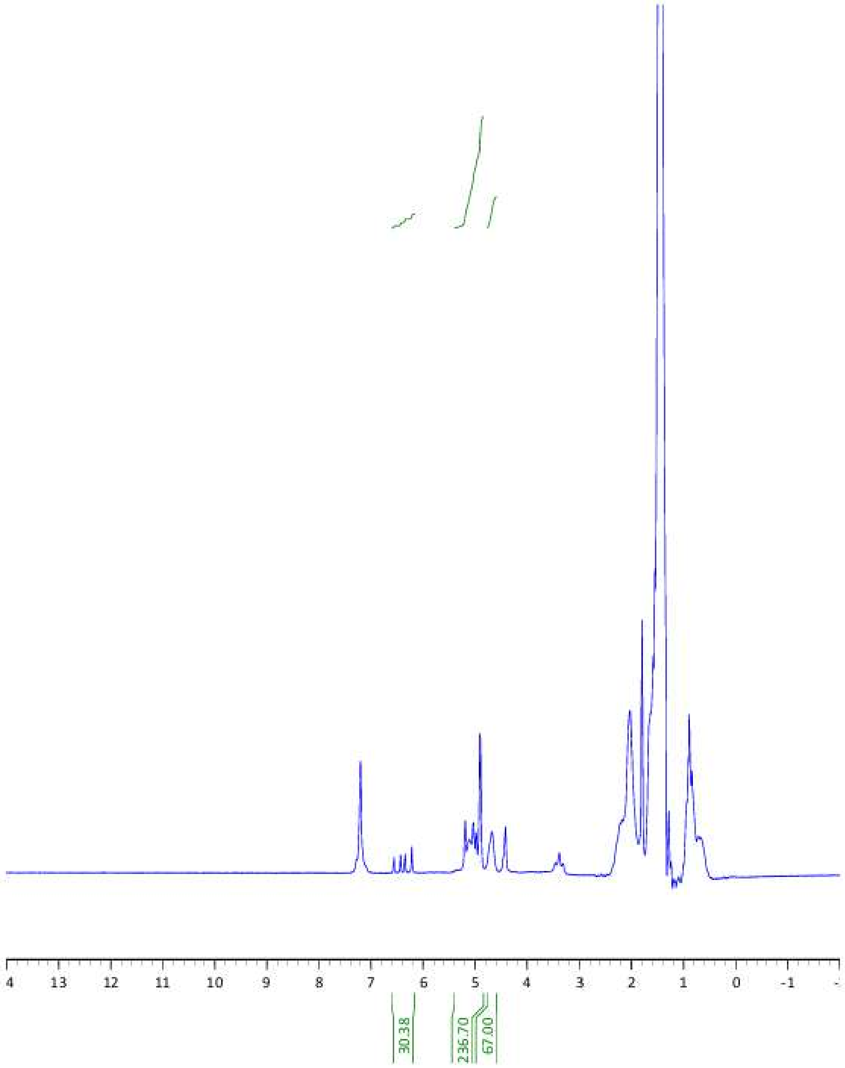

Figure S126: 120 Minutes OBn / Isoprene copolymerization (Low MWt) ^{1}H NMR Spectra

OBn / Isoprene copolymerization (Low MWt) ^1H NMR Spectra:

150 Minutes:

Spinsolve

1D EXTENDED+

Sample : JS739
Solvent : Cyclohexane
Custom :

Acquisition Date : 2025-10-03 13:15:15
Number of scans : 16
Acquisition time : 6.5536 s
Repetition time : 15 s
Pulse angle : 90 degrees
Experiment Duration : 00:04:02

Processing
Resolution enhancement : 0.3 LB + 0.3 GB
Line broadening : Exponential = 0.2 Hz
Gaussian = 0 Hz
Phasing : P0 = 5.00 P1 = 0.00
Baseline correction : None

Meta data
Instrument : SPA3598
Instrument type : 80 CARBON ULTRA DIFFUSION
Software version : 2.3.6.6590
Spinsolve User Setup : Spinsolve
Spinsolve User Acquisition : Spinsolve
Spinsolve User Processing : Spinsolve
Logged in Windows user : u2293373
Data folder : D:\20251003111253 obn reaction monitoring\00013

Backup folder :
Last shim : 2025-10-03 13:00:36
Shim linewidth @ 50% : 1.69 Hz
Shim linewidth @ 0.55% : 40.31 Hz
Shim SNR : 313700

Integrals

PPM Range	Normalized	Absolute
4.77 - 4.60	25.56 %	75.11
5.40 - 4.84	68.06 %	199.99
6.60 - 6.16	6.38 %	18.73

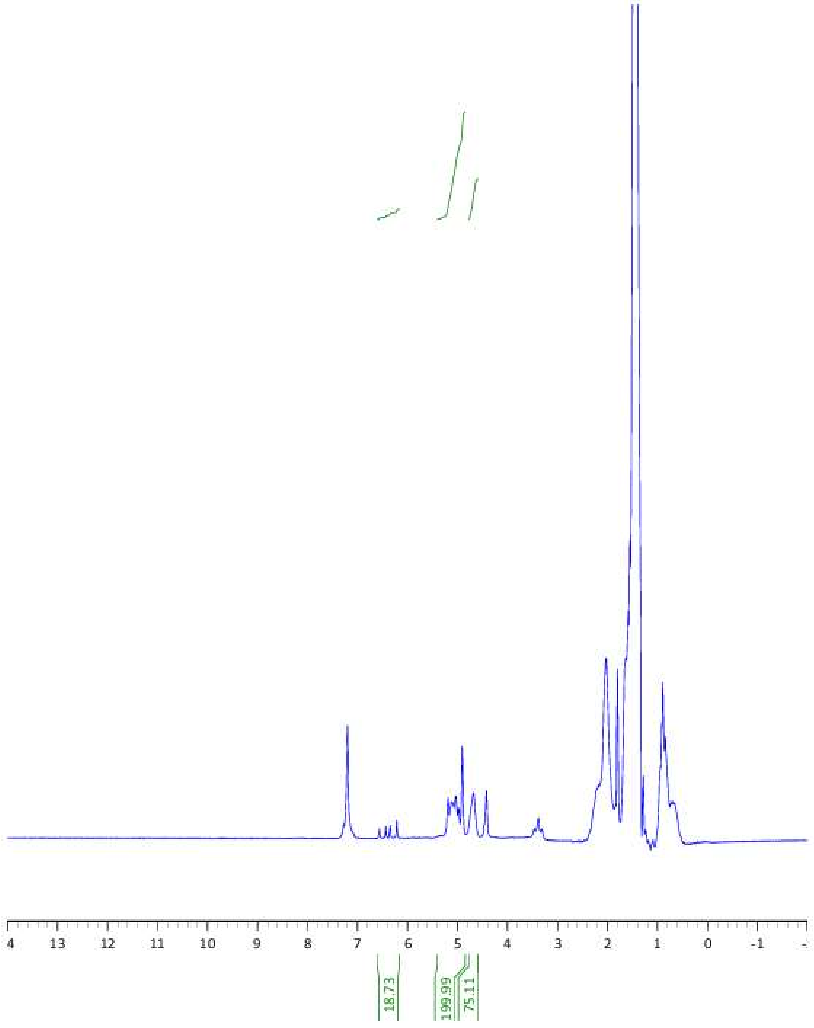

Figure S127: 150 Minutes OBn / Isoprene copolymerization (Low MWt) ^1H NMR Spectra

OBn / Isoprene copolymerization (Low MWt) ^1H NMR Spectra:

180 Minutes:

Spinsolve

1D EXTENDED+

Sample : JS739
Solvent : Cyclohexane
Custom :

Acquisition Date :	2025-10-03 13:45:15
Number of scans :	16
Acquisition time :	6.5536 s
Repetition time :	15 s
Pulse angle :	90 degrees
Experiment Duration :	00:04:02

Processing

Resolution enhancement :	0.3 LB + 0.3 GB
Line broadening :	Exponential = 0.2 Hz<br>Gaussian = 0 Hz
Phasing :	P0 = 5.00 P1 = 0.00
Baseline correction :	None

Meta data

Instrument :	SPA3598
Instrument type :	80 CARBON ULTRA DIFFUSION
Software version :	2.3.6.6590
Spinsolve User Setup :	Spinsolve
Spinsolve User Acquisition :	Spinsolve
Spinsolve User Processing :	Spinsolve
Logged in Windows user :	u2293373
Data folder :	D:\20251003111253 obn reaction m onitoring\00016
Backup folder :	
Last shim :	2025-10-03 13:30:36
Shim linewidth @ 50% :	1.67 Hz
Shim linewidth @ 0.55% :	40.33 Hz
Shim SNR :	361220

Integrals

PPM Range		Normalized	Absolute
4.77 -	4.60	29.74 %	82.85
5.40 -	4.84	65.15 %	181.49
6.60 -	6.16	5.11 %	14.23

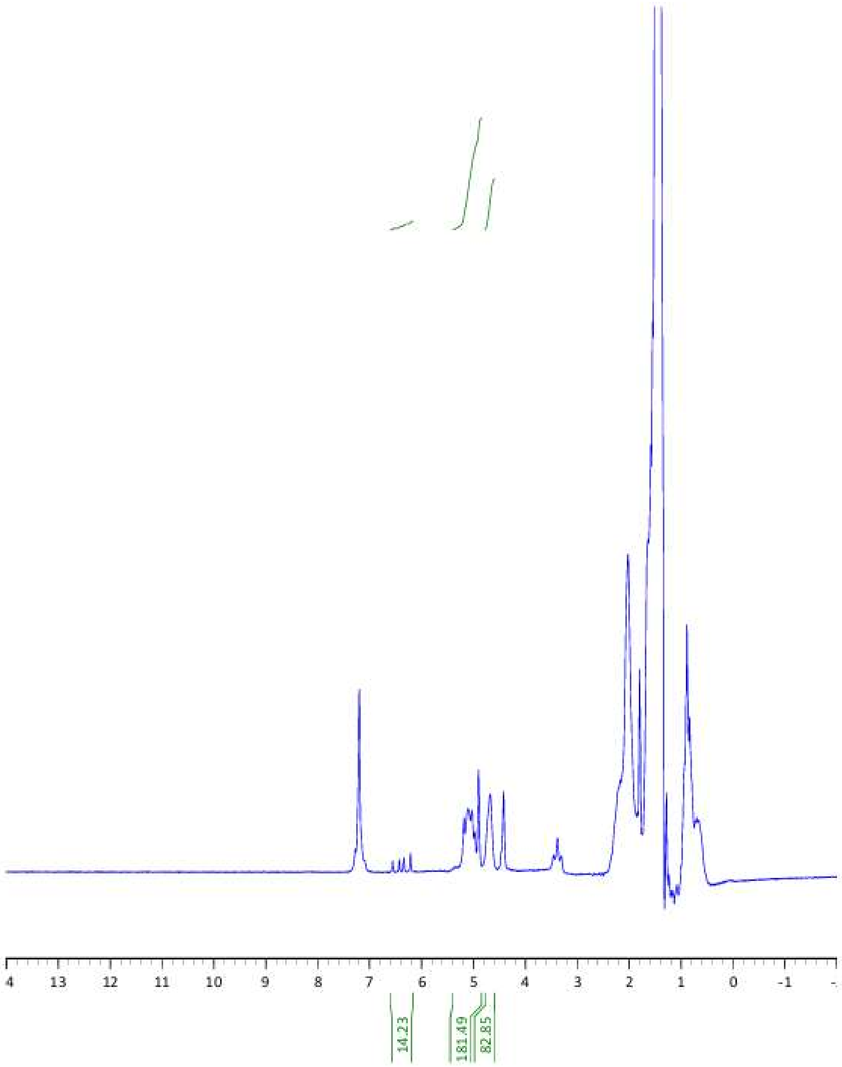

Figure S128: 180 Minutes OBn / Isoprene copolymerization (Low MWt) ^1H NMR Spectra

OBn / Isoprene copolymerization (Low MWt) ^{1}H NMR Spectra:

210 Minutes:

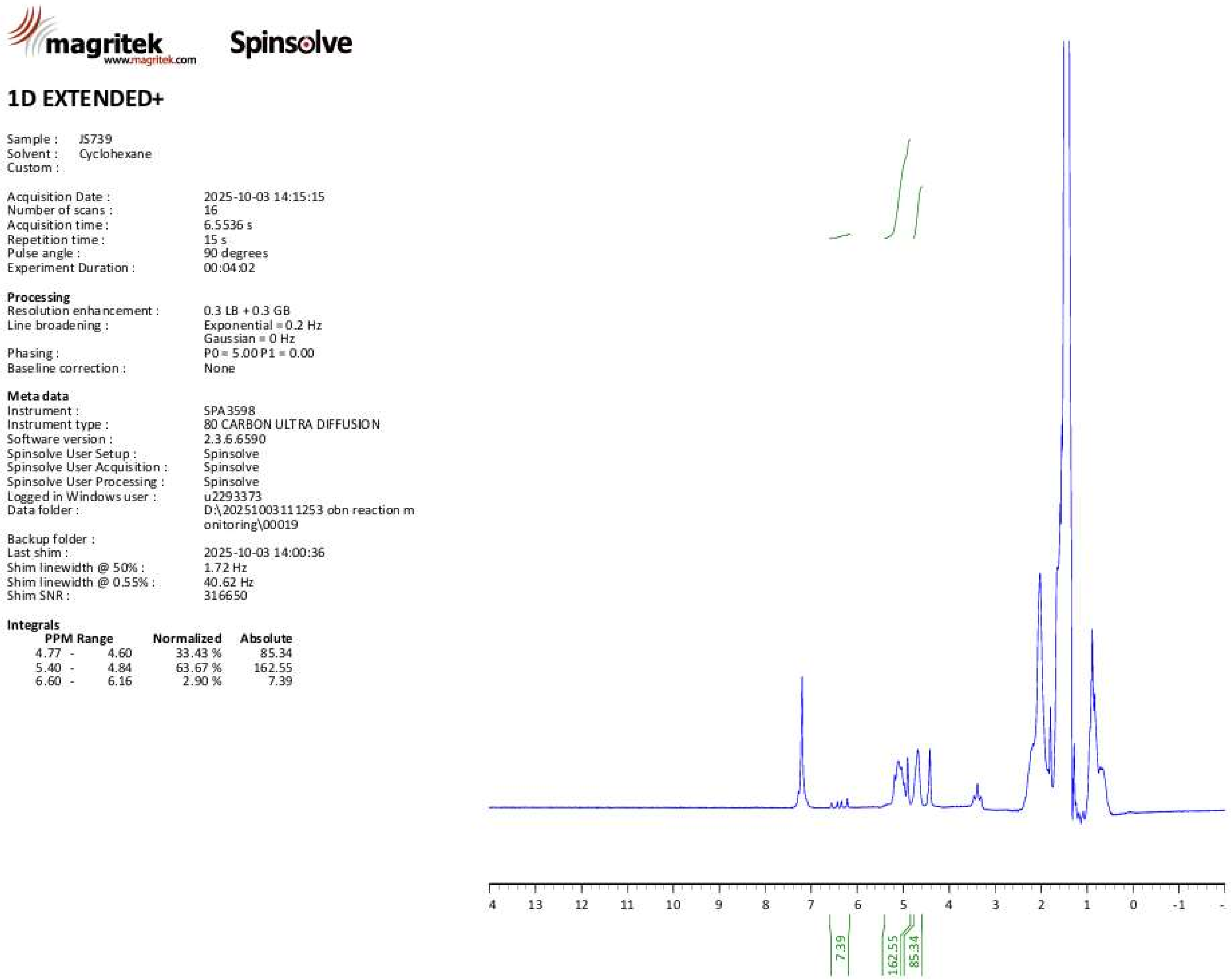

Figure S129: 210 Minutes OBn / Isoprene copolymerization (Low MWt) ^{1}H NMR Spectra

OBn / Isoprene copolymerization (Low MWt) ^{1}H NMR Spectra:

240 Minutes:

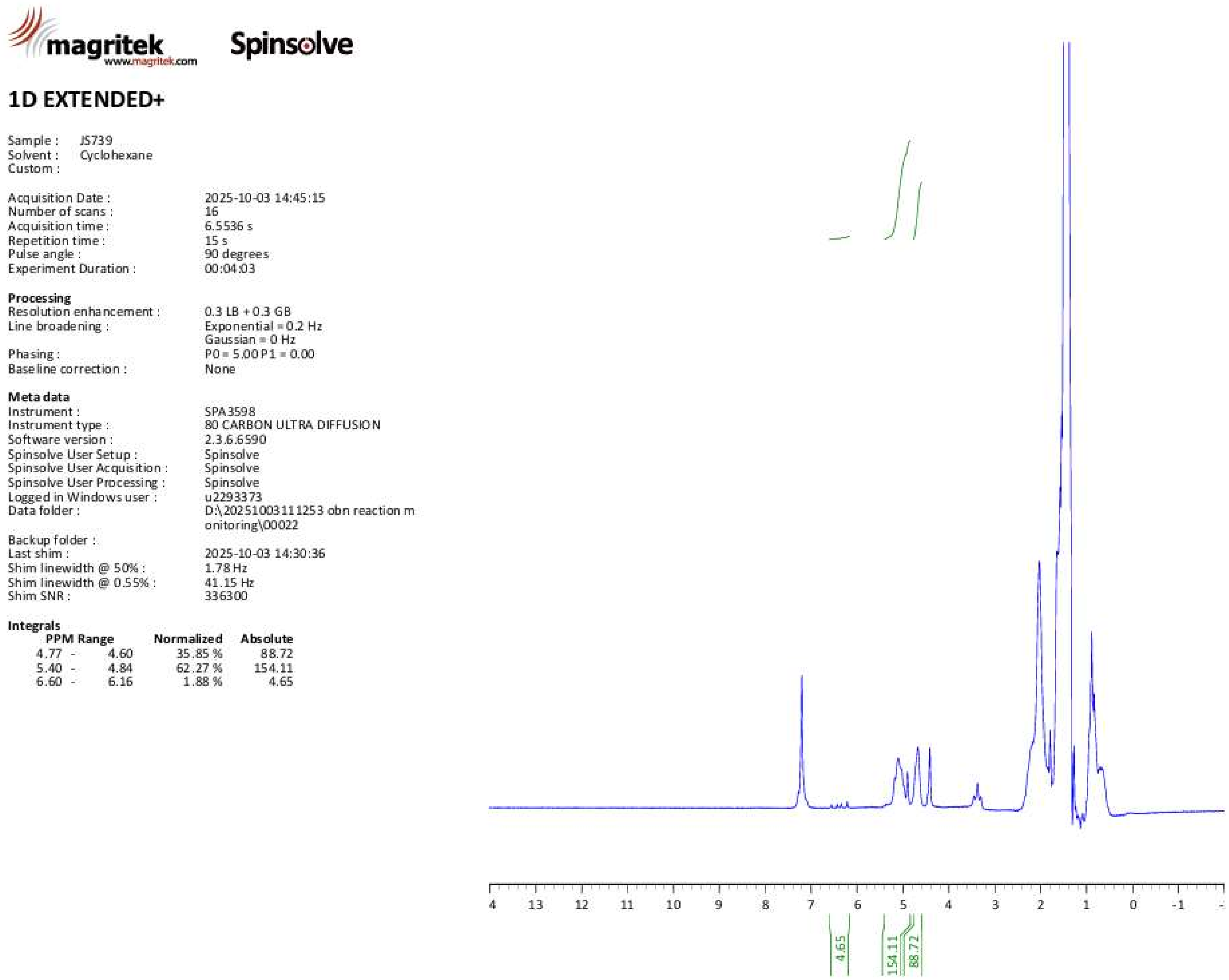

Figure S130: 240 Minutes OBn / Isoprene copolymerization (Low MWt) ^{1}H NMR Spectra

OBn / Isoprene copolymerization (Low MWt) ^{1}H NMR Spectra:

270 Minutes:

magritek www.magritek.com Spinsolve

1D EXTENDED+

Sample : JS739
Solvent : Cyclohexane
Custom :

Acquisition Date : 2025-10-03 15:15:15
Number of scans : 16
Acquisition time : 6.5536 s
Repetition time : 15 s
Pulse angle : 90 degrees
Experiment Duration : 00:04:02

Processing
Resolution enhancement : 0.3 LB + 0.3 GB
Line broadening : Exponential = 0.2 Hz
Gaussian = 0 Hz
Phasing : P0 = 5.40 P1 = 0.00
Baseline correction : None

Meta data
Instrument : SPA3598
Instrument type : 80 CARBON ULTRA DIFFUSION
Software version : 2.3.6.6590
Spinsolve User Setup : Spinsolve
Spinsolve User Acquisition : Spinsolve
Spinsolve User Processing : Spinsolve
Logged in Windows user : u2293373
Data folder : D:\20251003111253 obn reaction monitoring\00025
Backup folder :
Last shim : 2025-10-03 15:00:38
Shim linewidth @ 50% : 1.82 Hz
Shim linewidth @ 0.55% : 41.33 Hz
Shim SNR : 327540

Integrals

PPM Range			Normalized	Absolute
4.77	-	4.60	38.03 %	89.66
5.40	-	4.84	61.49 %	144.95
6.60	-	6.16	0.48 %	1.13

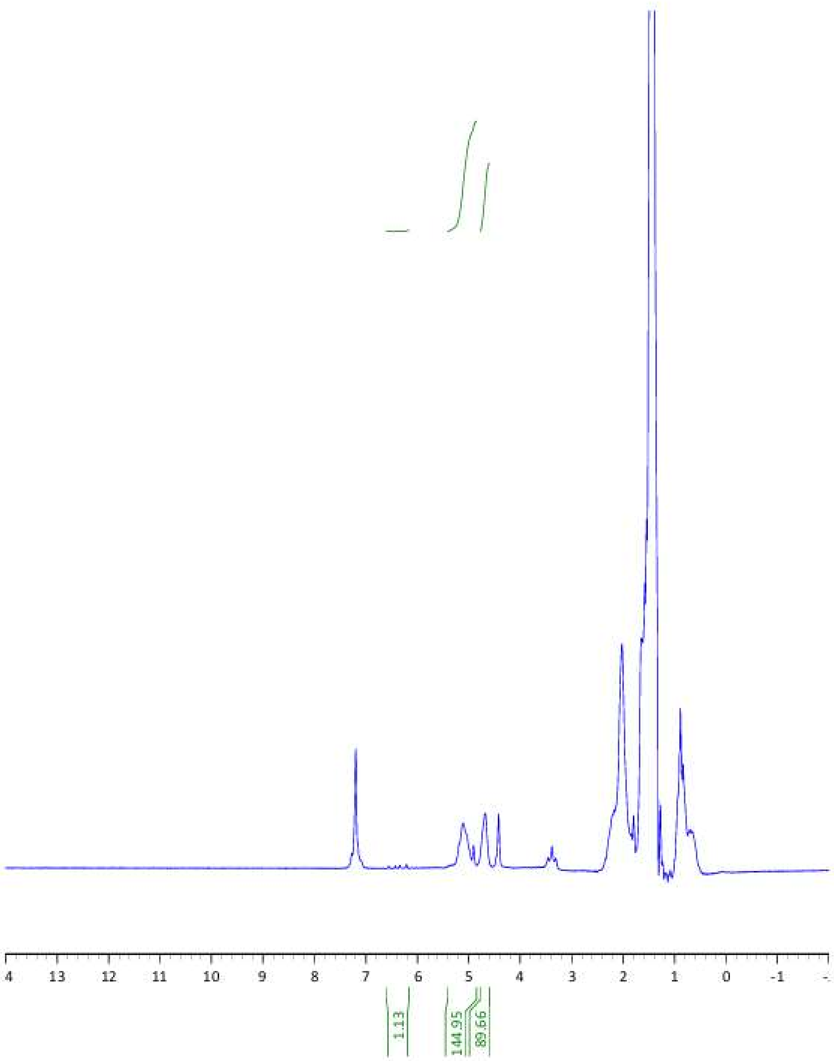

Figure S131: 270 Minutes OBn / Isoprene copolymerization (Low MWt) ^{1}H NMR Spectra

OBn / Isoprene copolymerization (Low MWt) ^1H NMR Spectra:

300 Minutes:

magritek
www.magritek.com

Spinsolve

1D EXTENDED+

Sample : JS739
Solvent : Cyclohexane
Custom :

Acquisition Date : 2025-10-03 15:45:15
Number of scans : 16
Acquisition time : 6.5536 s
Repetition time : 15 s
Pulse angle : 90 degrees
Experiment Duration : 00:04:02

Processing
Resolution enhancement : 0.3 LB + 0.3 GB
Line broadening : Exponential = 0.2 Hz
Gaussian = 0 Hz
Phasing : P0 = 5.40 P1 = 0.00
Baseline correction : None

Meta data
Instrument : SPA3598
Instrument type : 80 CARBON ULTRA DIFFUSION
Software version : 2.3.6.6590
Spinsolve User Setup : Spinsolve
Spinsolve User Acquisition : Spinsolve
Spinsolve User Processing : Spinsolve
Logged in Windows user : u2293373
Data folder : D:\20251003111253 obn reaction monitoring\00028
Backup folder :
Last shim : 2025-10-03 15:30:37
Shim linewidth @ 50% : 1.84 Hz
Shim linewidth @ 0.55% : 41.51 Hz
Shim SNR : 324750

Integrals

PPM Range		Normalized	Absolute
4.77 -	4.60	39.23 %	91.38
5.40 -	4.84	60.73 %	141.46
6.60 -	6.16	0.04 %	0.10

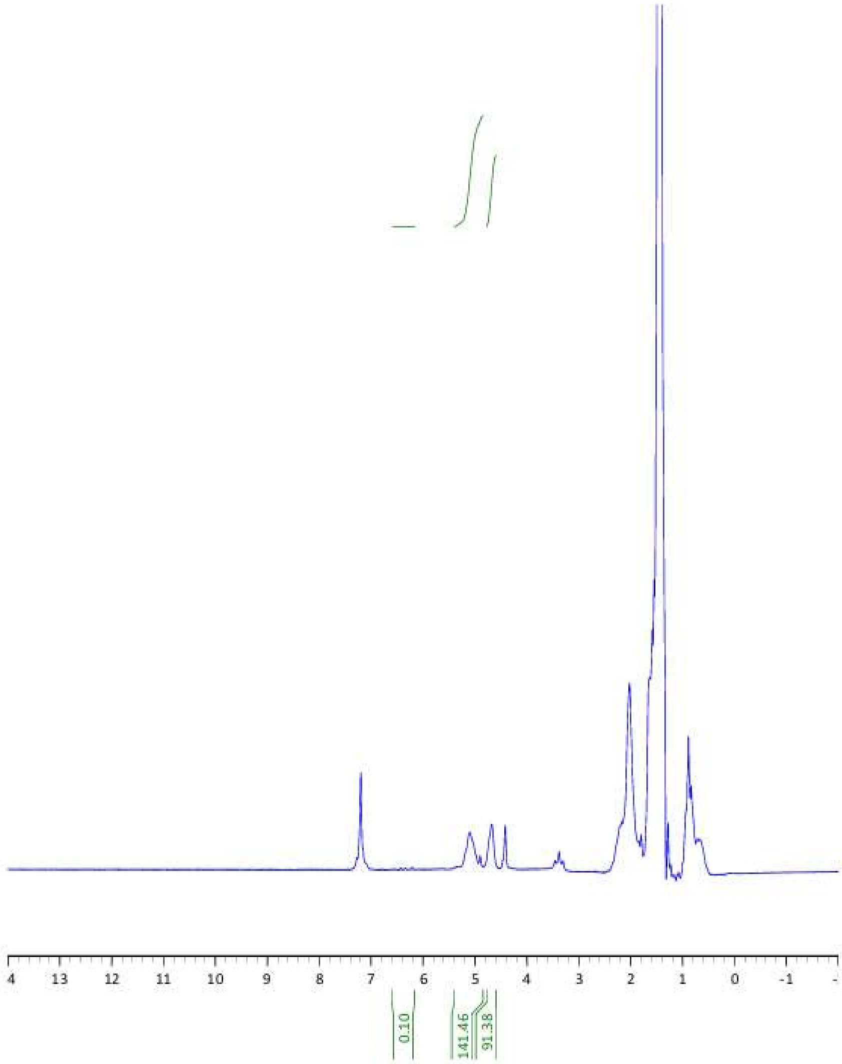

Figure S132: 300 Minutes OBn / Isoprene copolymerization (Low MWt) ^1H NMR Spectra

OBn / Isoprene copolymerization (Low MWt) ^{1}H NMR Spectra:

330 minutes:

magritek www.magritek.com Spinsolve

1D EXTENDED+

Sample : JS739
Solvent : Cyclohexane
Custom :

Acquisition Date :	2025-10-03 16:15:15
Number of scans :	16
Acquisition time :	6.5536 s
Repetition time :	15 s
Pulse angle :	90 degrees
Experiment Duration :	00:04:02

Processing

Resolution enhancement :	0.3 LB + 0.3 GB
Line broadening :	Exponential = 0.2 Hz<br>Gaussian = 0 Hz
Phasing :	P0 = 6.00 P1 = 0.00
Baseline correction :	None

Meta data

Instrument :	SPA3598
Instrument type :	80 CARBON ULTRA DIFFUSION
Software version :	2.3.6.6590
Spinsolve User Setup :	Spinsolve
Spinsolve User Acquisition :	Spinsolve
Spinsolve User Processing :	Spinsolve
Logged in Windows user :	u2293373
Data folder :	D:\20251003111253 obn reaction m onitoring\00031
Backup folder :	
Last shim :	2025-10-03 16:00:37
Shim linewidth @ 50% :	1.87 Hz
Shim linewidth @ 0.55% :	41.82 Hz
Shim SNR :	333180

Integrals

PPM Range		Normalized	Absolute
4.77 -	4.60	39.78 %	92.95
5.40 -	4.84	60.20 %	140.67
6.60 -	6.16	0.02 %	0.04

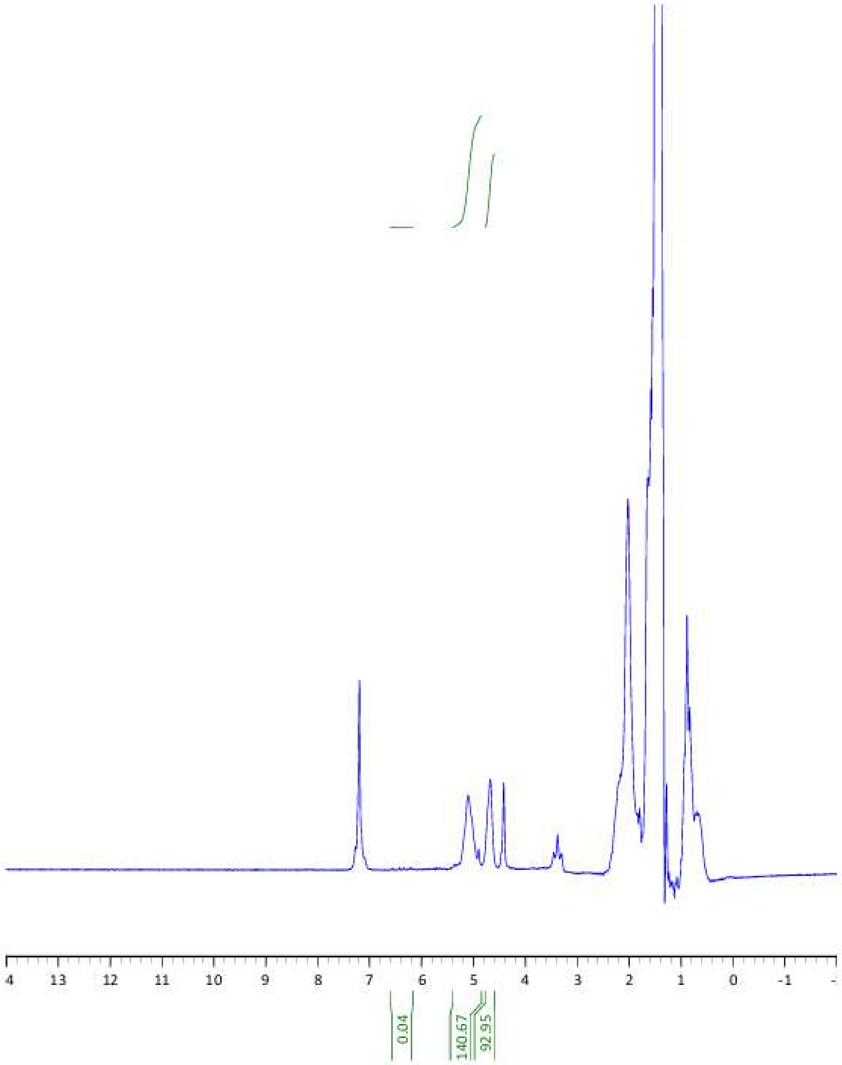

Figure S133: 330 Minutes OBn / Isoprene copolymerization (Low MWt) ^{1}H NMR Spectra

OBn / Isoprene copolymerization (Low MWt) ^{1}H NMR Spectra:

360 minutes:

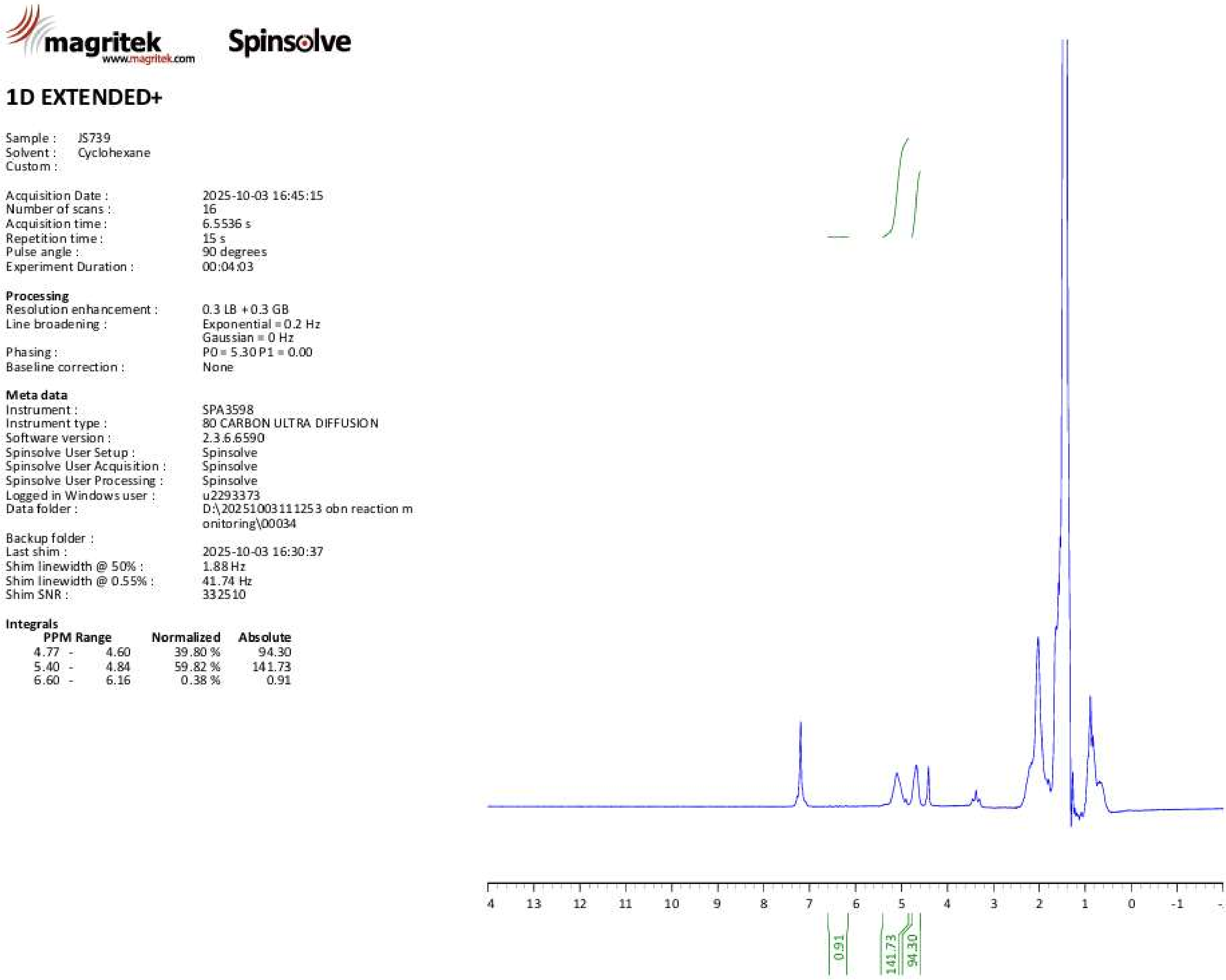

Figure S134: 360 Minutes OBn / Isoprene copolymerization (Low MWt) ^{1}H NMR Spectra

OBn / Isoprene copolymerization (High MWt) ^{1}H NMR Spectra:

30 Minutes:

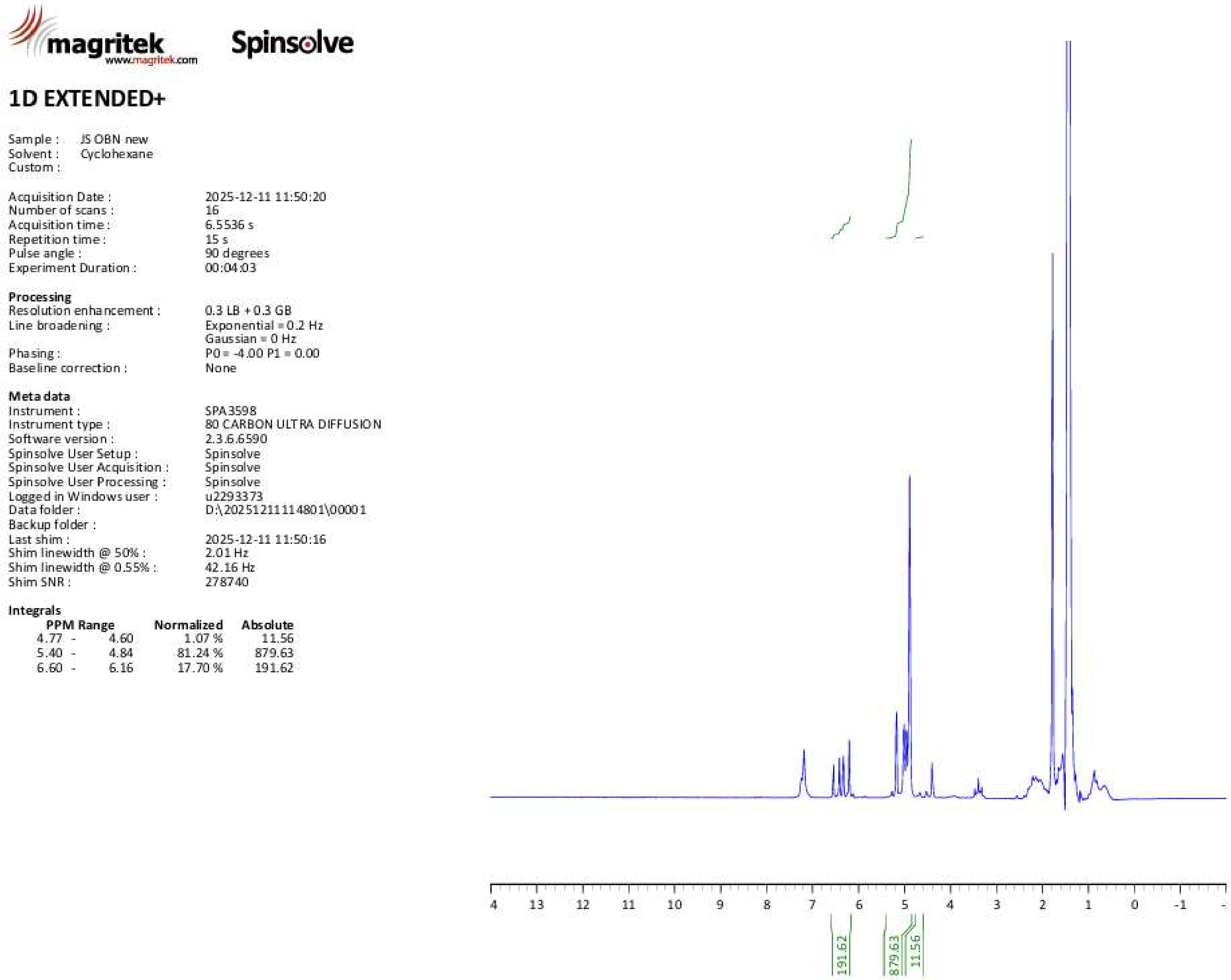

Figure S135: 30 Minutes OBn / Isoprene copolymerization (High MWt) ^{1}H NMR Spectra:

OBn / Isoprene copolymerization (High MWt) ^{1}H NMR Spectra:

60 minutes:

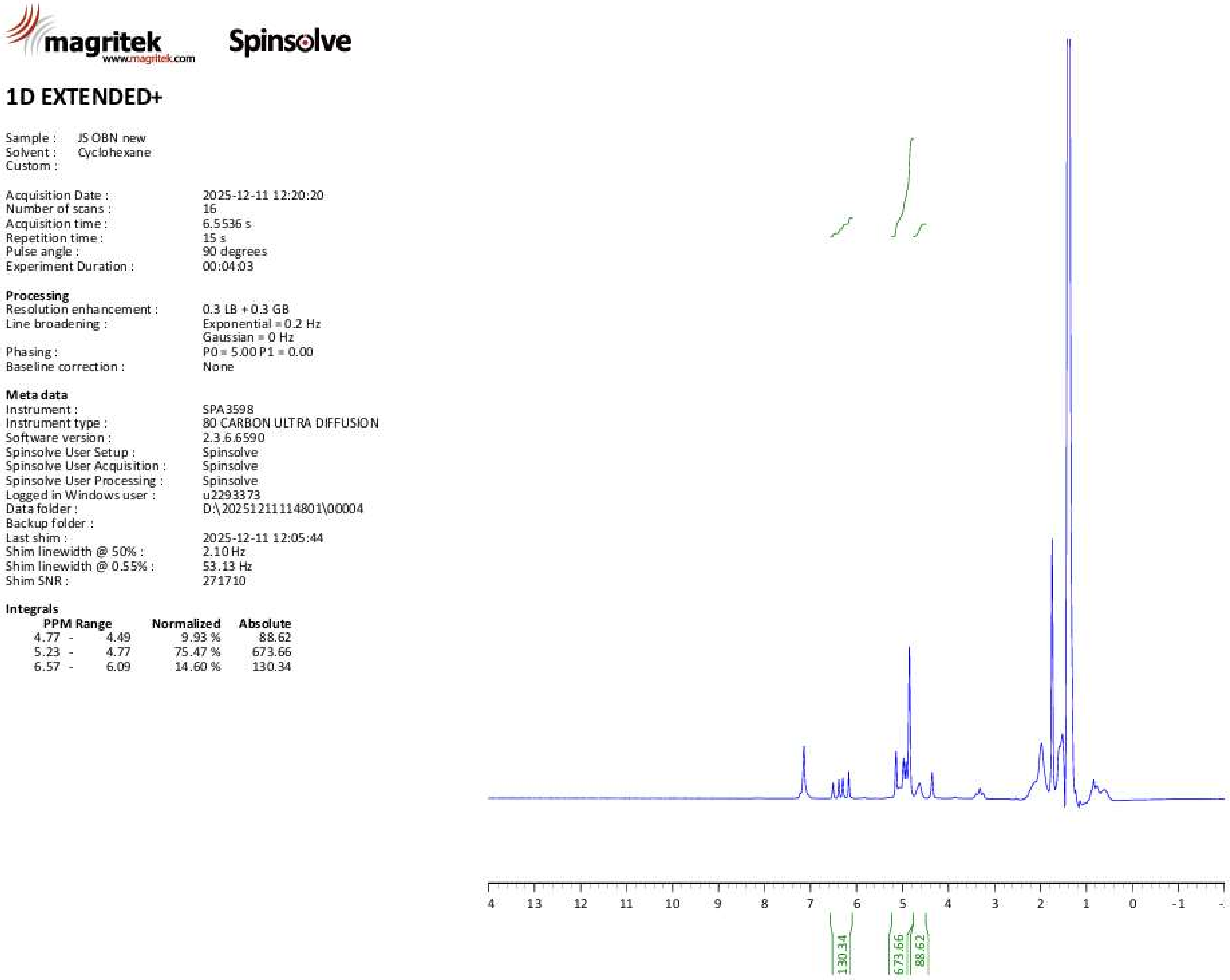

Figure S136: 60 Minutes OBn / Isoprene copolymerization (High MWt) ^{1}H NMR Spectra:

OBn / Isoprene copolymerization (High MWt) ^{1}H NMR Spectra:

90 minutes:

Spinsolve

1D EXTENDED+

Sample : JS OBN new
Solvent : Cyclohexane
Custom :

Acquisition Date : 2025-12-11 12:50:20
Number of scans : 16
Acquisition time : 6.5536 s
Repetition time : 15 s
Pulse angle : 90 degrees
Experiment Duration : 00:04:03

Processing
Resolution enhancement : 0.3 LB + 0.3 GB
Line broadening : Exponential = 0.2 Hz
Gaussian = 0 Hz
Phasing : P0 = 5.00 P1 = 0.00
Baseline correction : None

Meta data
Instrument : SPA3598
Instrument type : 80 CARBON ULTRA DIFFUSION
Software version : 2.3.6.6590
Spinsolve User Setup : Spinsolve
Spinsolve User Acquisition : Spinsolve
Spinsolve User Processing : Spinsolve
Logged in Windows user : u2293373
Data folder : D:\20251211114801\00007
Backup folder :
Last shim : 2025-12-11 12:35:48
Shim linewidth @ 50% : 1.69 Hz
Shim linewidth @ 0.55% : 39.54 Hz
Shim SNR : 329780

Integrals

PPM Range			Normalized	Absolute
4.81	-	4.55	19.28 %	139.09
5.28	-	4.84	69.71 %	502.83
6.61	-	6.13	11.01 %	79.41

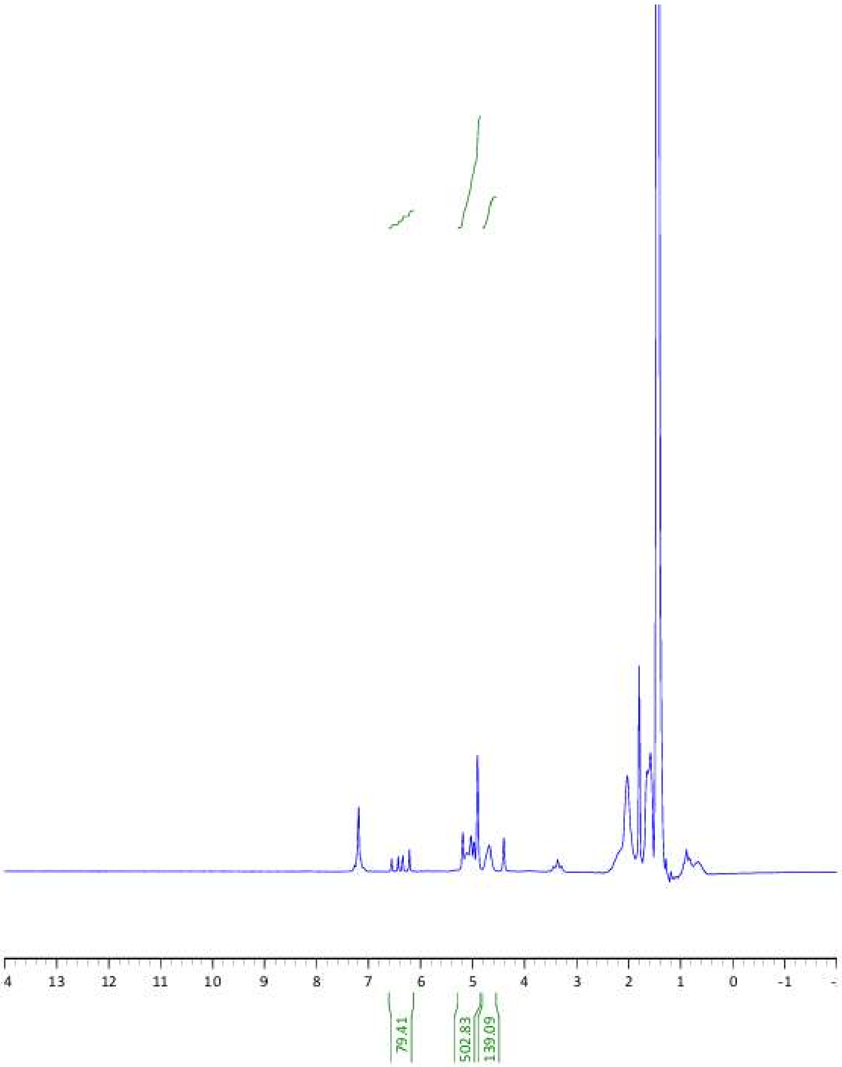

Figure S137: 90 Minutes OBn / Isoprene copolymerization (High MWt) ^{1}H NMR Spectra:

OBn / Isoprene copolymerization (High MWt) ^{1}H NMR Spectra:

120 minutes:

Spinsolve

1D EXTENDED+

Sample :	JS OBN new
Solvent :	Cyclohexane
Custom :	

Acquisition Date :	2025-12-11 13:20:20
Number of scans :	16
Acquisition time :	6.5536 s
Repetition time :	15 s
Pulse angle :	90 degrees
Experiment Duration :	00:04:03

Processing

Resolution enhancement :	0.3 LB + 0.3 GB
Line broadening :	Exponential = 0.2 Hz<br>Gaussian = 0 Hz
Phasing :	P0 = 5.00 P1 = 0.00
Baseline correction :	None

Meta data

Instrument :	SPA3598
Instrument type :	80 CARBON ULTRA DIFFUSION
Software version :	2.3.6.6590
Spinsolve User Setup :	Spinsolve
Spinsolve User Acquisition :	Spinsolve
Spinsolve User Processing :	Spinsolve
Logged in Windows user :	u2293373
Data folder :	D:\20251211114801\00010
Backup folder :	
Last shim :	2025-12-11 13:05:43
Shim linewidth @ 50% :	1.67 Hz
Shim linewidth @ 0.55% :	39.67 Hz
Shim SNR :	325950

Integrals

PPM Range	Normalized	Absolute
4.81 - 4.55	27.22 %	168.13
5.28 - 4.84	65.07 %	401.86
6.61 - 6.13	7.71 %	47.61

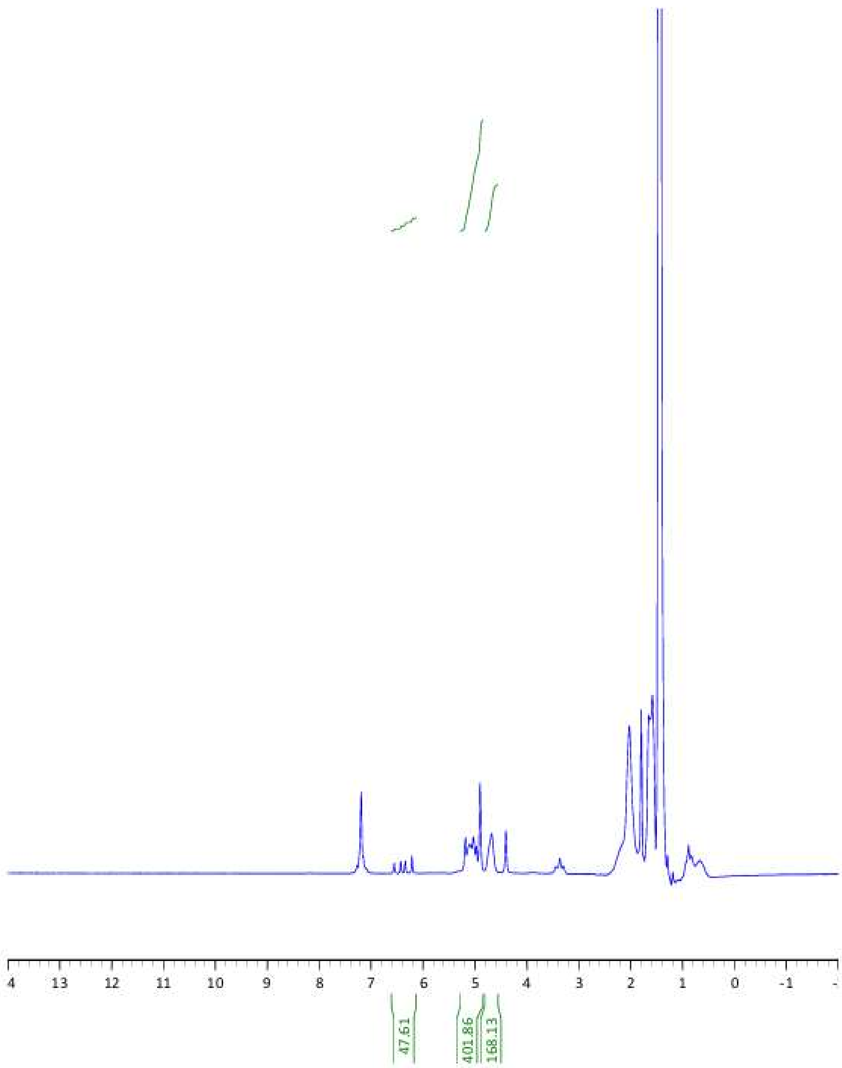

Figure S138: 120 Minutes OBn / Isoprene copolymerization (High MWt) ^{1}H NMR Spectra:

OBn / Isoprene copolymerization (High MWt) ^{1}H NMR Spectra:

150 minutes:

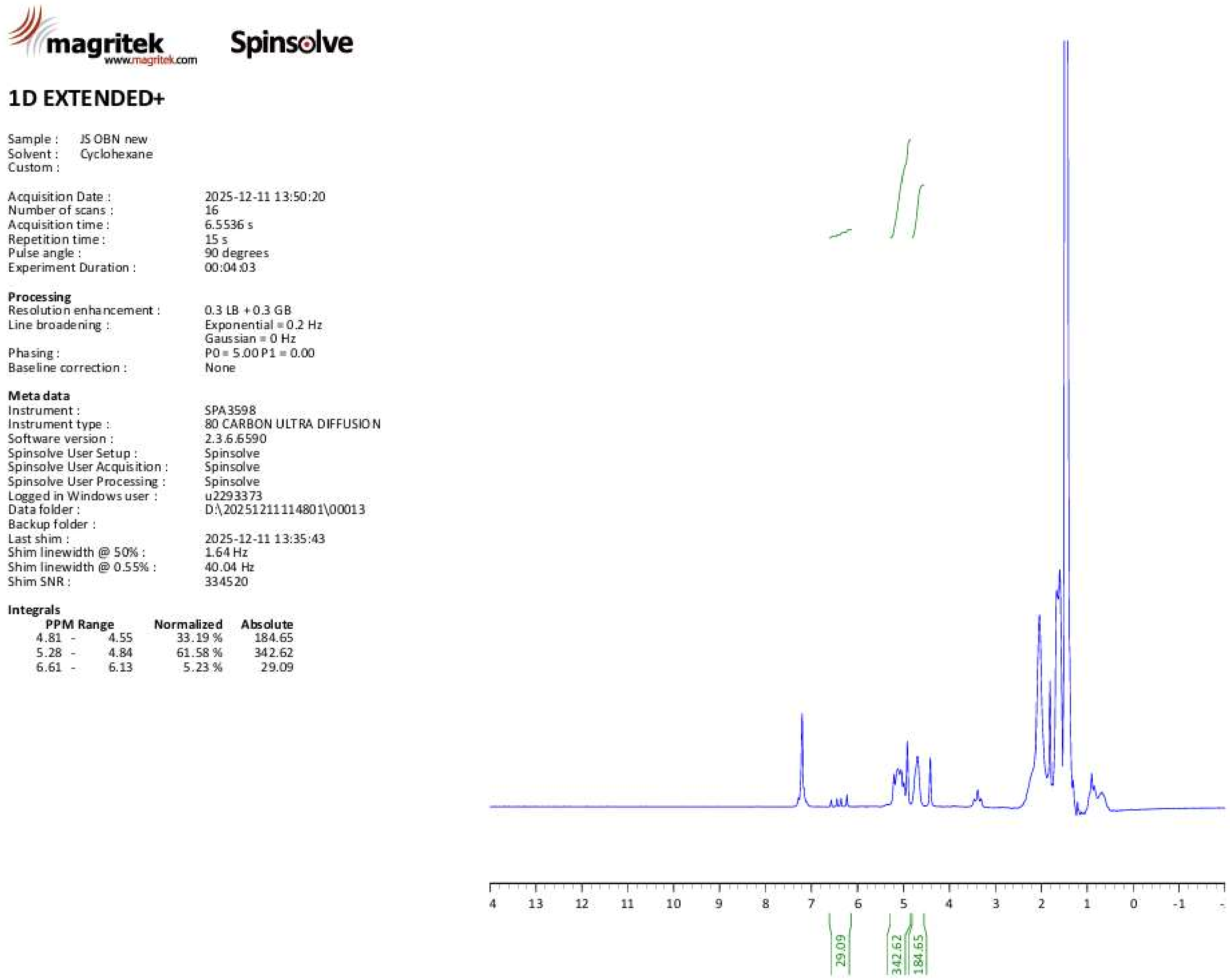

Figure S139: 150 *Minutes OBn / Isoprene copolymerization (High MWt)* ^{1}*H NMR Spectra:*

OBn / Isoprene copolymerization (High MWt) ^{1}H NMR Spectra:

180 minutes:

magritek www.magritek.com Spinsolve

1D EXTENDED+

Sample : JS OBN new
Solvent : Cyclohexane
Custom :

Acquisition Date :	2025-12-11 14:20:20
Number of scans :	16
Acquisition time :	6.5536 s
Repetition time :	15 s
Pulse angle :	90 degrees
Experiment Duration :	00:04:03

Processing

Resolution enhancement :	0.3 LB + 0.3 GB
Line broadening :	Exponential = 0.2 Hz<br>Gaussian = 0 Hz
Phasing :	P0 = 5.00 P1 = 0.00
Baseline correction :	None

Meta data

Instrument :	SPA3598
Instrument type :	80 CARBON ULTRA DIFFUSION
Software version :	2.3.6.6590
Spinsolve User Setup :	Spinsolve
Spinsolve User Acquisition :	Spinsolve
Spinsolve User Processing :	Spinsolve
Logged in Windows user :	u2293373
Data folder :	D:\20251211114801\00016
Backup folder :	
Last shim :	2025-12-11 14:05:44
Shim linewidth @ 50% :	1.67 Hz
Shim linewidth @ 0.55% :	40.82 Hz
Shim SNR :	318150

Integrals

PPM Range			Normalized	Absolute
4.81	-	4.55	37.84 %	197.04
5.28	-	4.84	58.88 %	306.62
6.61	-	6.13	3.28 %	17.10

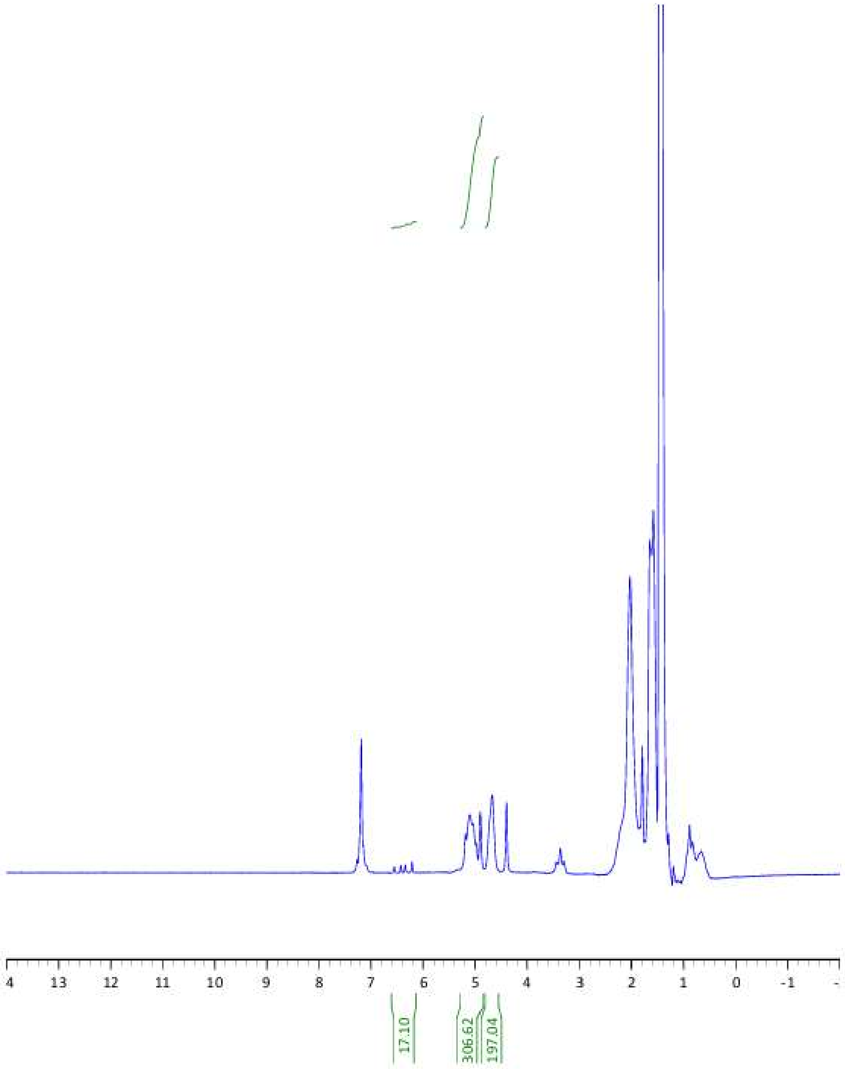

Figure S140: 180 Minutes OBn / Isoprene copolymerization (High MWt) ^{1}H NMR Spectra:

OBn / Isoprene copolymerization (High MWt) ^{1}H NMR Spectra:

210 minutes:

Spinsolve

1D EXTENDED+

Sample :	JS OBN new
Solvent :	Cyclohexane
Custom :	

Acquisition Date :	2025-12-11 14:50:20
Number of scans :	16
Acquisition time :	6.5536 s
Repetition time :	15 s
Pulse angle :	90 degrees
Experiment Duration :	00:04:03

Processing

Resolution enhancement :	0.3 LB + 0.3 GB
Line broadening :	Exponential = 0.2 Hz<br>Gaussian = 0 Hz
Phasing :	P0 = 5.00 P1 = 0.00
Baseline correction :	None

Meta data

Instrument :	SPA3598
Instrument type :	80 CARBON ULTRA DIFFUSION
Software version :	2.3.6.6590
Spinsolve User Setup :	Spinsolve
Spinsolve User Acquisition :	Spinsolve
Spinsolve User Processing :	Spinsolve
Logged in Windows user :	u2293373
Data folder :	D:\20251211114801\00019
Backup folder :	
Last shim :	2025-12-11 14:35:43
Shim linewidth @ 50% :	1.70 Hz
Shim linewidth @ 0.55% :	41.50 Hz
Shim SNR :	316230

Integrals

PPM Range			Normalized	Absolute
4.81	-	4.55	41.00 %	203.55
5.28	-	4.84	57.17 %	283.80
6.61	-	6.13	1.82 %	9.06

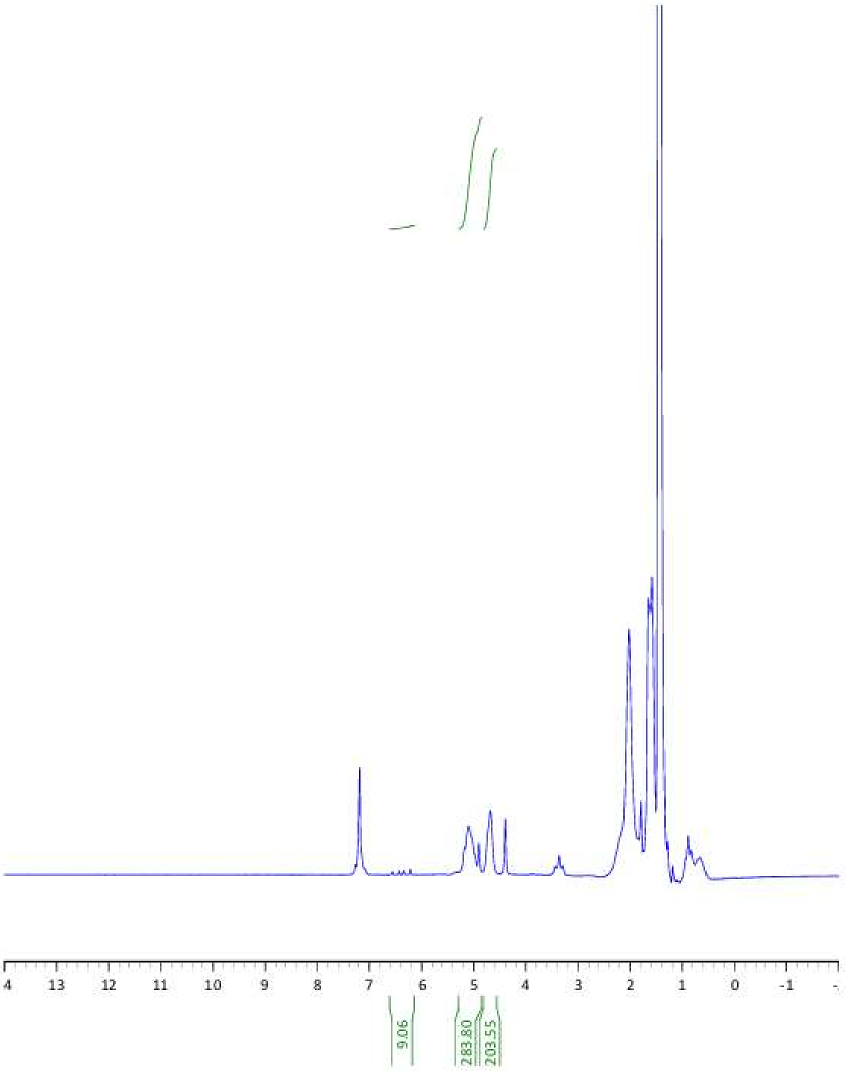

Figure S141: 210 Minutes OBn / Isoprene copolymerization (High MWt) ^{1}H NMR Spectra:

OBn / Isoprene copolymerization (High MWt) ^{1}H NMR Spectra:

240 minutes:

magritek www.magritek.com Spinsolve

1D EXTENDED+

Sample : JS OBN new
Solvent : Cyclohexane
Custom :

Acquisition Date : 2025-12-11 15:20:20
Number of scans : 16
Acquisition time : 6.5536 s
Repetition time : 15 s
Pulse angle : 90 degrees
Experiment Duration : 00:04:03

Processing
Resolution enhancement : 0.3 LB + 0.3 GB
Line broadening : Exponential = 0.2 Hz
Gaussian = 0 Hz
Phasing : P0 = 4.87 P1 = 0.00
Baseline correction : None

Meta data
Instrument : SPA3598
Instrument type : 80 CARBON ULTRA DIFFUSION
Software version : 2.3.6.6590
Spinsolve User Setup : Spinsolve
Spinsolve User Acquisition : Spinsolve
Spinsolve User Processing : Spinsolve
Logged in Windows user : u2293373
Data folder : D:\20251211114801\00022
Backup folder :
Last shim : 2025-12-11 15:05:43
Shim linewidth @ 50% : 1.72 Hz
Shim linewidth @ 0.55% : 41.71 Hz
Shim SNR : 318920

Integrals

PPM Range	Normalized	Absolute
4.81 - 4.55	43.50 %	206.06
5.28 - 4.84	56.44 %	267.33
6.61 - 6.13	0.06 %	0.29

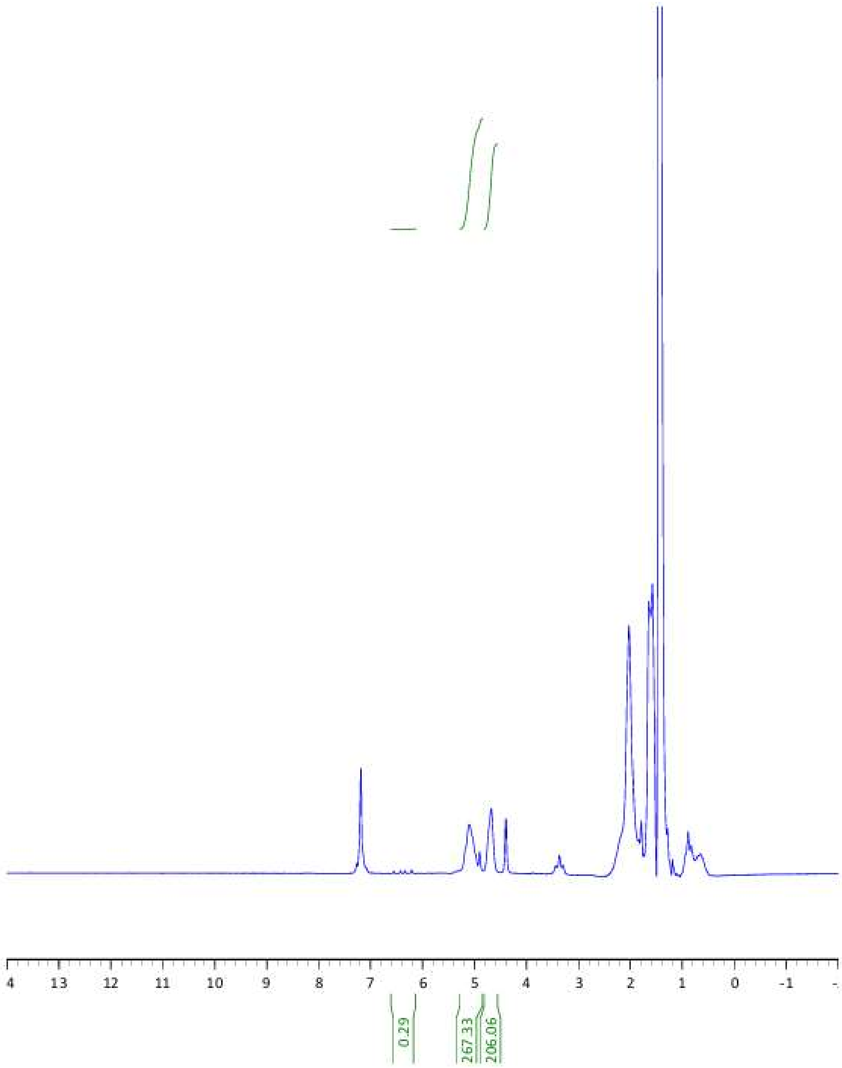

Figure S142: 240 Minutes OBn / Isoprene copolymerization (High MWt) ^{1}H NMR Spectra:

OBn / Isoprene copolymerization (High MWt) ^{1}H NMR Spectra:

270 minutes:

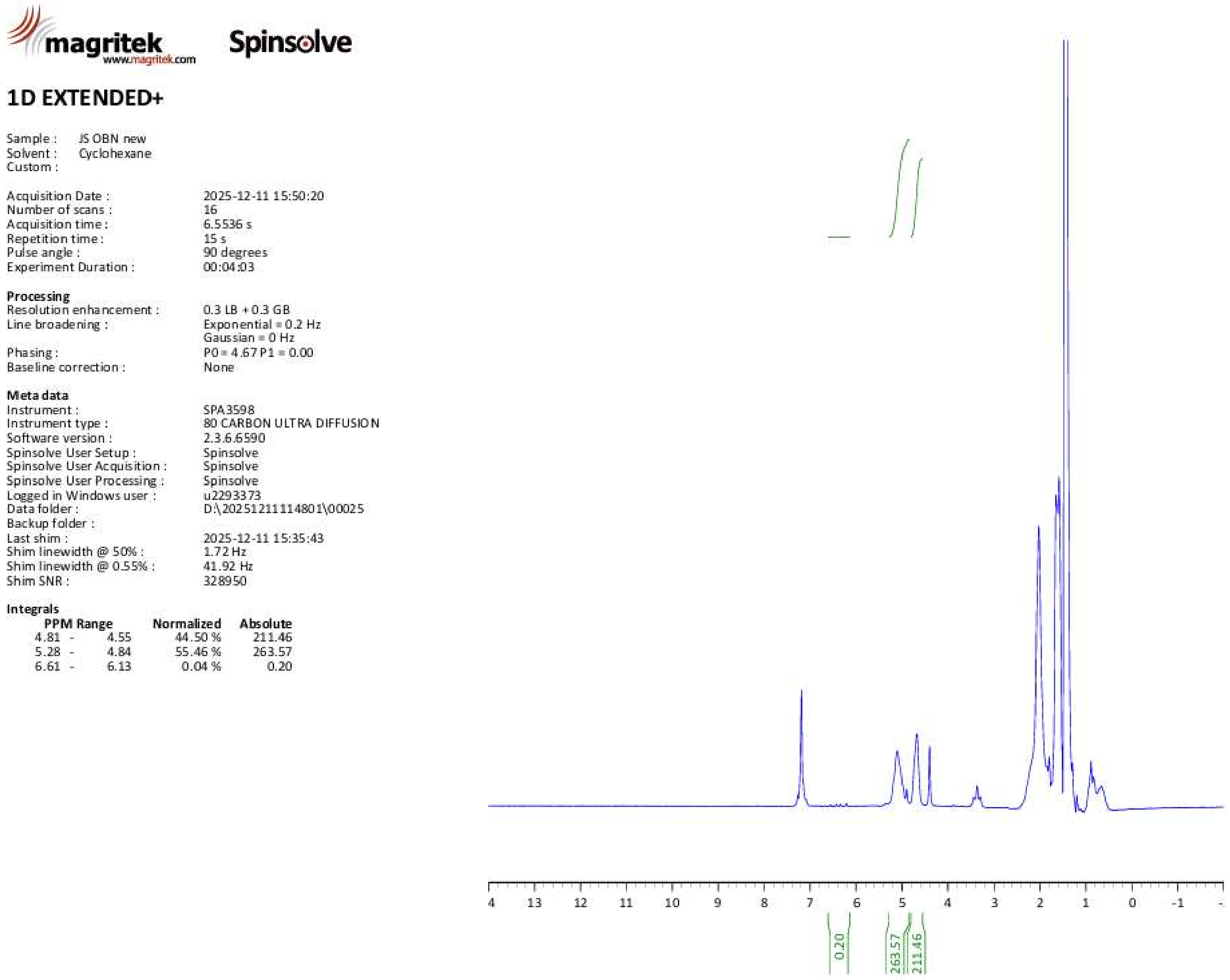

Figure S143: 270 Minutes OBn / Isoprene copolymerization (High MWt) ^{1}H NMR Spectra:

OBn / Isoprene copolymerization (High MWt) ^{1}H NMR Spectra:

300 minutes:

1D EXTENDED+

Sample : JS OBN new
Solvent : Cyclohexane
Custom :

Acquisition Date :	2025-12-11 16:20:20
Number of scans :	16
Acquisition time :	6.5536 s
Repetition time :	15 s
Pulse angle :	90 degrees
Experiment Duration :	00:04:03

Processing

Resolution enhancement :	0.3 LB + 0.3 GB
Line broadening :	Exponential = 0.2 Hz<br>Gaussian = 0 Hz
Phasing :	P0 = 5.12 P1 = 0.00
Baseline correction :	None

Meta data

Instrument :	SPA3598
Instrument type :	80 CARBON ULTRA DIFFUSION
Software version :	2.3.6.6590
Spinsolve User Setup :	Spinsolve
Spinsolve User Acquisition :	Spinsolve
Spinsolve User Processing :	Spinsolve
Logged in Windows user :	u2293373
Data folder :	D:\20251211114801\00028
Backup folder :	
Last shim :	2025-12-11 16:05:44
Shim linewidth @ 50% :	1.72 Hz
Shim linewidth @ 0.55% :	42.10 Hz
Shim SNR :	327200

Integrals

PPM Range	Normalized	Absolute
4.81 - 4.55	45.19 %	215.21
5.28 - 4.84	54.78 %	260.90
6.61 - 6.13	0.04 %	0.17

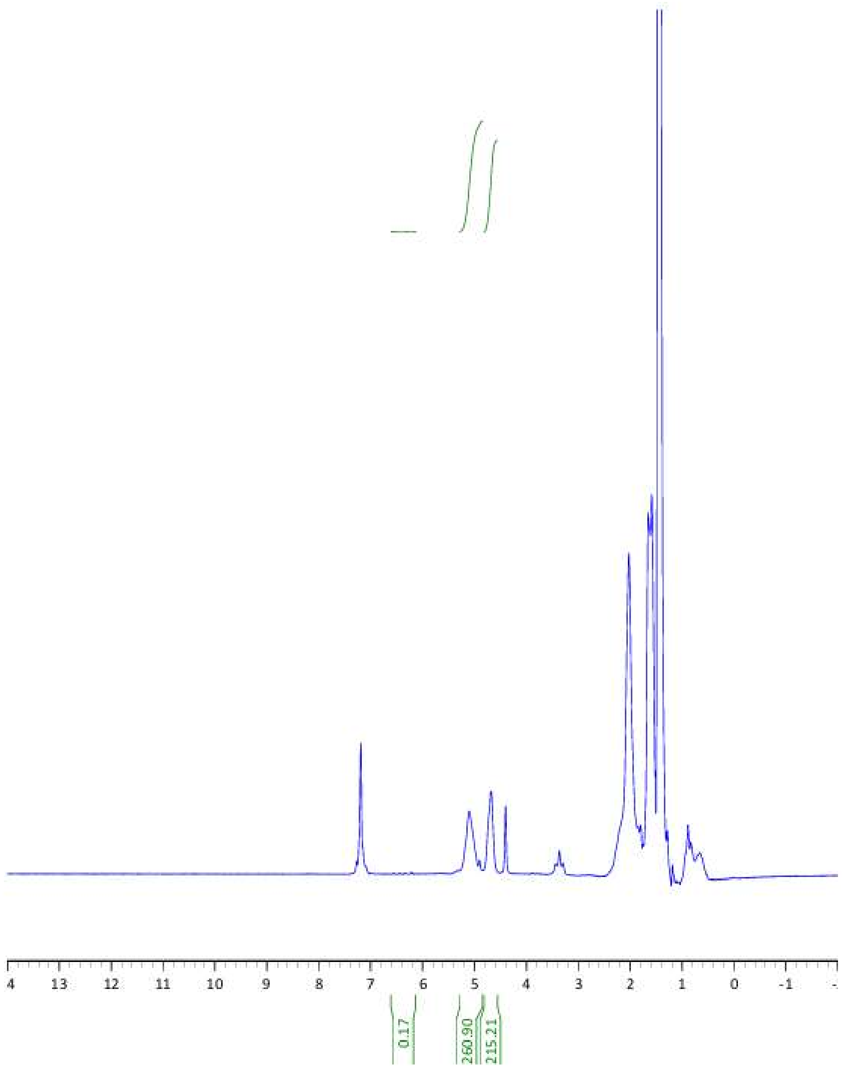

Figure S144: 300 Minutes OBn / Isoprene copolymerization (High MWt) ^{1}H NMR Spectra:

OBn / Isoprene copolymerization (High MWt) ^{1}H NMR Spectra:

330 Minutes:

magritek www.magritek.com Spinsolve

1D EXTENDED+

Sample : JS OBN new
Solvent : Cyclohexane
Custom :

Acquisition Date : 2025-12-11 16:50:20
Number of scans : 16
Acquisition time : 6.5536 s
Repetition time : 15 s
Pulse angle : 90 degrees
Experiment Duration : 00:04:03

Processing
Resolution enhancement : 0.3 LB + 0.3 GB
Line broadening : Exponential = 0.2 Hz
Gaussian = 0 Hz
Phasing : P0 = 4.80 P1 = 0.00
Baseline correction : None

Meta data
Instrument : SPA3598
Instrument type : 80 CARBON ULTRA DIFFUSION
Software version : 2.3.6.6590
Spinsolve User Setup : Spinsolve
Spinsolve User Acquisition : Spinsolve
Spinsolve User Processing : Spinsolve
Logged in Windows user : u2293373
Data folder : D:\20251211114801\00031
Backup folder :
Last shim : 2025-12-11 16:35:44
Shim linewidth @ 50% : 1.71 Hz
Shim linewidth @ 0.55% : 41.77 Hz
Shim SNR : 323860

Integrals

PPM Range		Normalized	Absolute
4.81 -	4.55	45.55 %	218.09
5.28 -	4.84	54.35 %	260.20
6.61 -	6.13	0.10 %	0.46

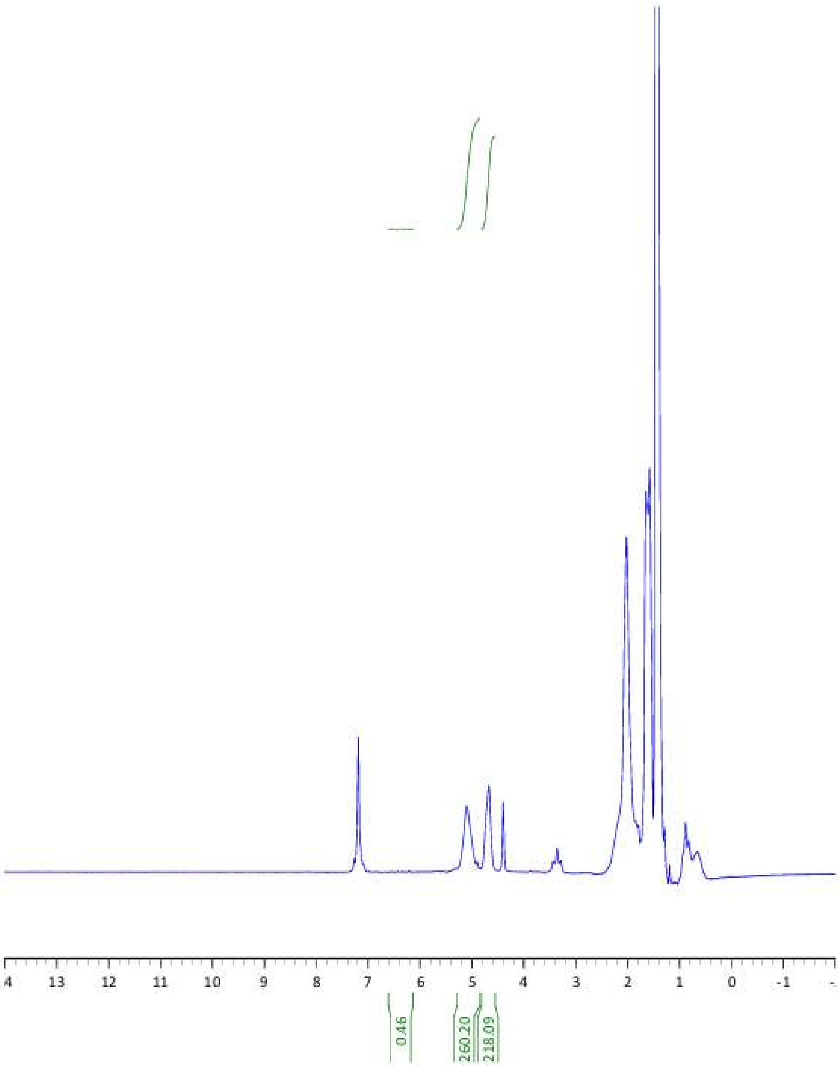

Figure S145: 330 Minutes OBn / Isoprene copolymerization (High MWt) ^{1}H NMR Spectra:

OBn / Isoprene copolymerization (High MWt) ^{1}H NMR Spectra:

360 Minutes:

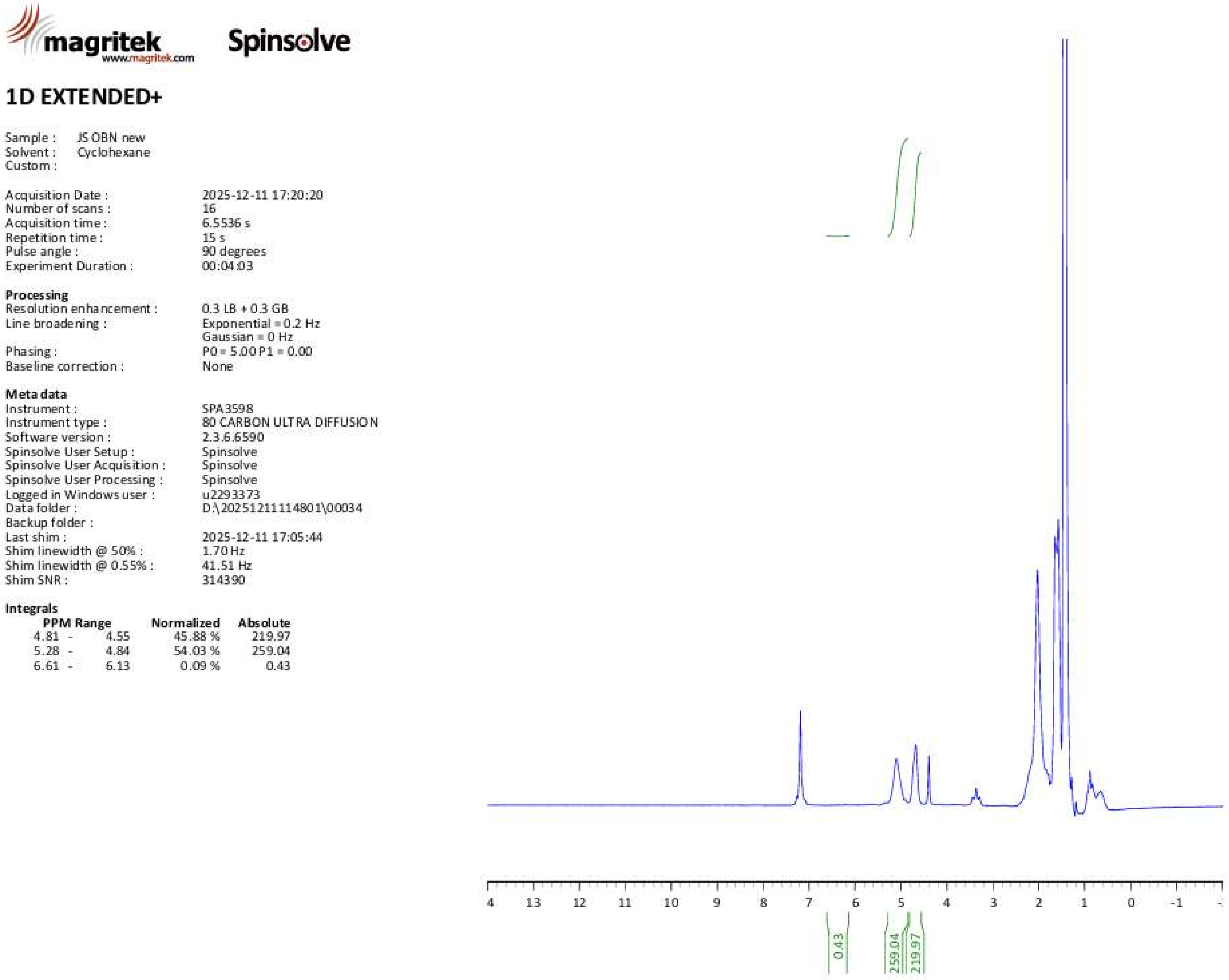

Figure S146: 360 Minutes OBn / Isoprene copolymerization (High MWt) ^{1}H NMR Spectra:

OBn / Isoprene copolymerization (High MWt) ^{1}H NMR Spectra:

390 Minutes:

magritek Spinsolve

1D EXTENDED+

Sample : JS OBN new
Solvent : Cyclohexane
Custom :

Acquisition Date :	2025-12-11 17:50:20
Number of scans :	16
Acquisition time :	6.5536 s
Repetition time :	15 s
Pulse angle :	90 degrees
Experiment Duration :	00:04:03

Processing

Resolution enhancement :	0.3 LB + 0.3 GB
Line broadening :	Exponential = 0.2 Hz<br>Gaussian = 0 Hz
Phasing :	P0 = 5.00 P1 = 0.00
Baseline correction :	None

Meta data

Instrument :	SPA3598
Instrument type :	80 CARBON ULTRA DIFFUSION
Software version :	2.3.6.6590
Spinsolve User Setup :	Spinsolve
Spinsolve User Acquisition :	Spinsolve
Spinsolve User Processing :	Spinsolve
Logged in Windows user :	u2293373
Data folder :	D:\20251211114801\00037
Backup folder :	
Last shim :	2025-12-11 17:35:43
Shim linewidth @ 50% :	1.69 Hz
Shim linewidth @ 0.55% :	41.33 Hz
Shim SNR :	318060

Integrals

PPM Range			Normalized	Absolute
4.81	-	4.55	46.04 %	221.02
5.28	-	4.84	53.82 %	258.37
6.61	-	6.13	0.14 %	0.65

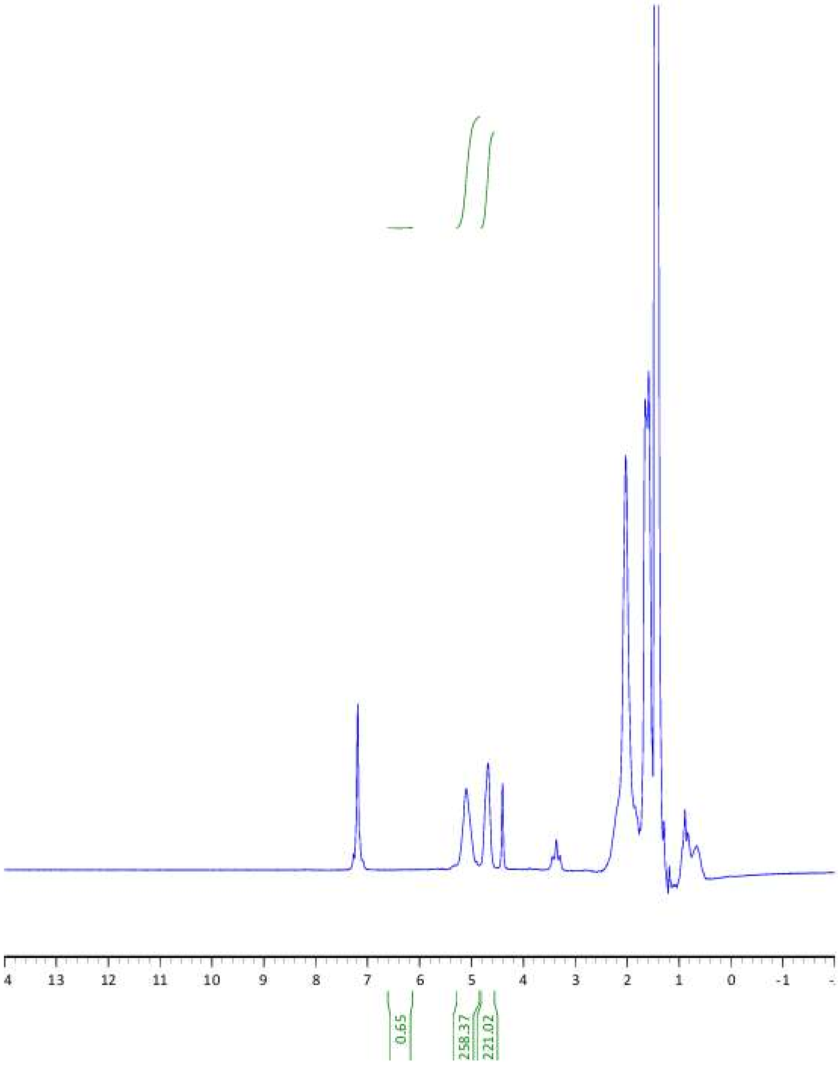

Figure S147: 390 Minutes OBn / Isoprene copolymerization (High MWt) ^{1}H NMR Spectra:

OBn / Isoprene copolymerization (High MWt) ^{1}H NMR Spectra:

420 Minutes:

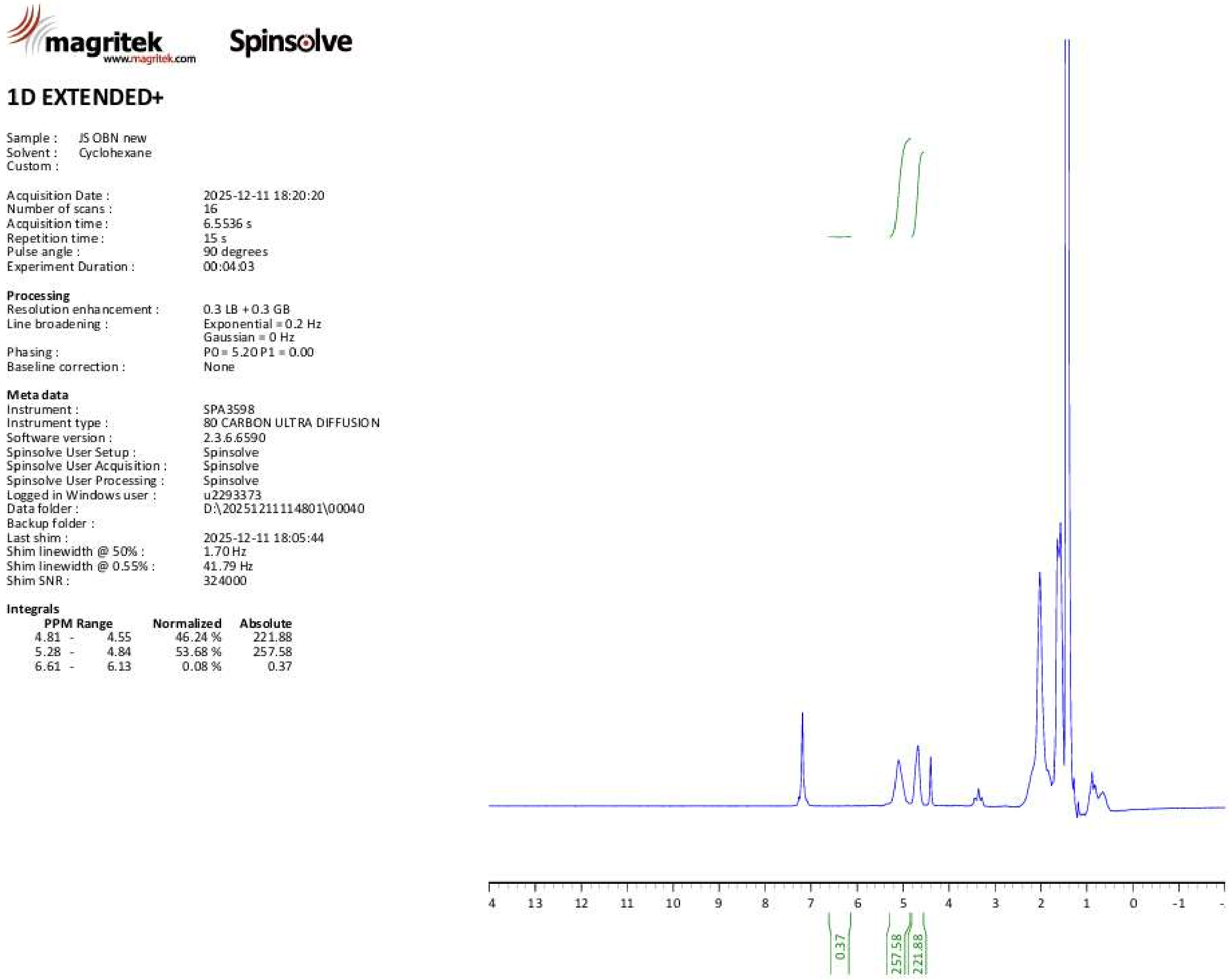

Figure S148: 420 Minutes OBn / Isoprene copolymerization (High MWt) ^{1}H NMR Spectra:

OTBS / Isoprene copolymerization ^{1}H NMR Spectra:

30 Minutes:

magritek www.magritek.com Spinsolve

1D EXTENDED+

Sample : JS OTBS new
Solvent : Cyclohexane
Custom :

Acquisition Date :	2025-12-10 19:05:53
Number of scans :	16
Acquisition time :	6.5536 s
Repetition time :	15 s
Pulse angle :	90 degrees
Experiment Duration :	00:04:02

Processing

Resolution enhancement :	0.3 LB + 0.3 GB
Line broadening :	Exponential = 0.2 Hz<br>Gaussian = 0 Hz
Phasing :	P0 = -3.80 P1 = 0.00
Baseline correction :	None

Meta data

Instrument :	SPA3598
Instrument type :	80 CARBON ULTRA DIFFUSION
Software version :	2.3.6.6590
Spinsolve User Setup :	Spinsolve
Spinsolve User Acquisition :	Spinsolve
Spinsolve User Processing :	Spinsolve
Logged in Windows user :	u2293373
Data folder :	D:\20251210190335\00001
Backup folder :	
Last shim :	2025-12-10 19:05:49
Shim linewidth @ 50% :	1.75 Hz
Shim linewidth @ 0.55% :	33.13 Hz
Shim SNR :	351090

Integrals

PPM Range		Normalized	Absolute
4.74 -	4.59	0.24 %	1.70
5.30 -	4.77	81.45 %	585.26
6.65 -	6.07	18.31 %	131.59

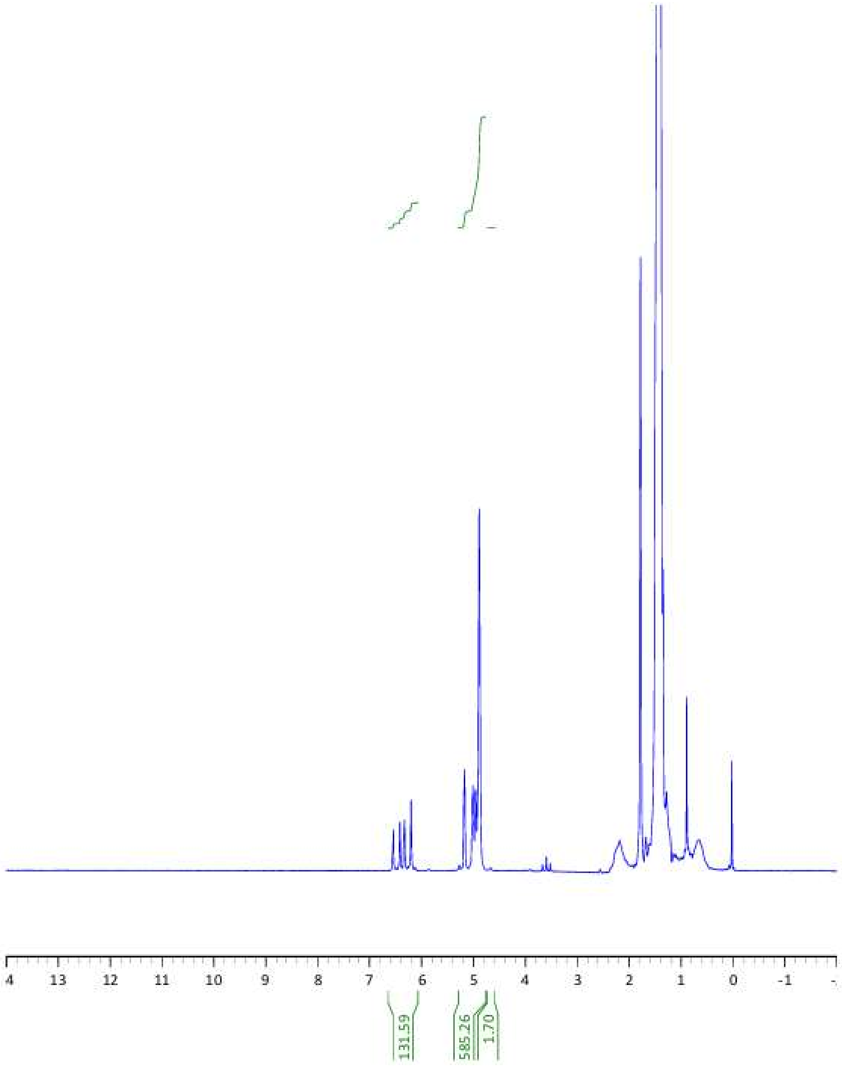

Figure S149: 30 minutes OTBS / Isoprene copolymerization ^{1}H NMR Spectra:

OTBS / Isoprene copolymerization ^{1}H NMR Spectra:

60 minutes:

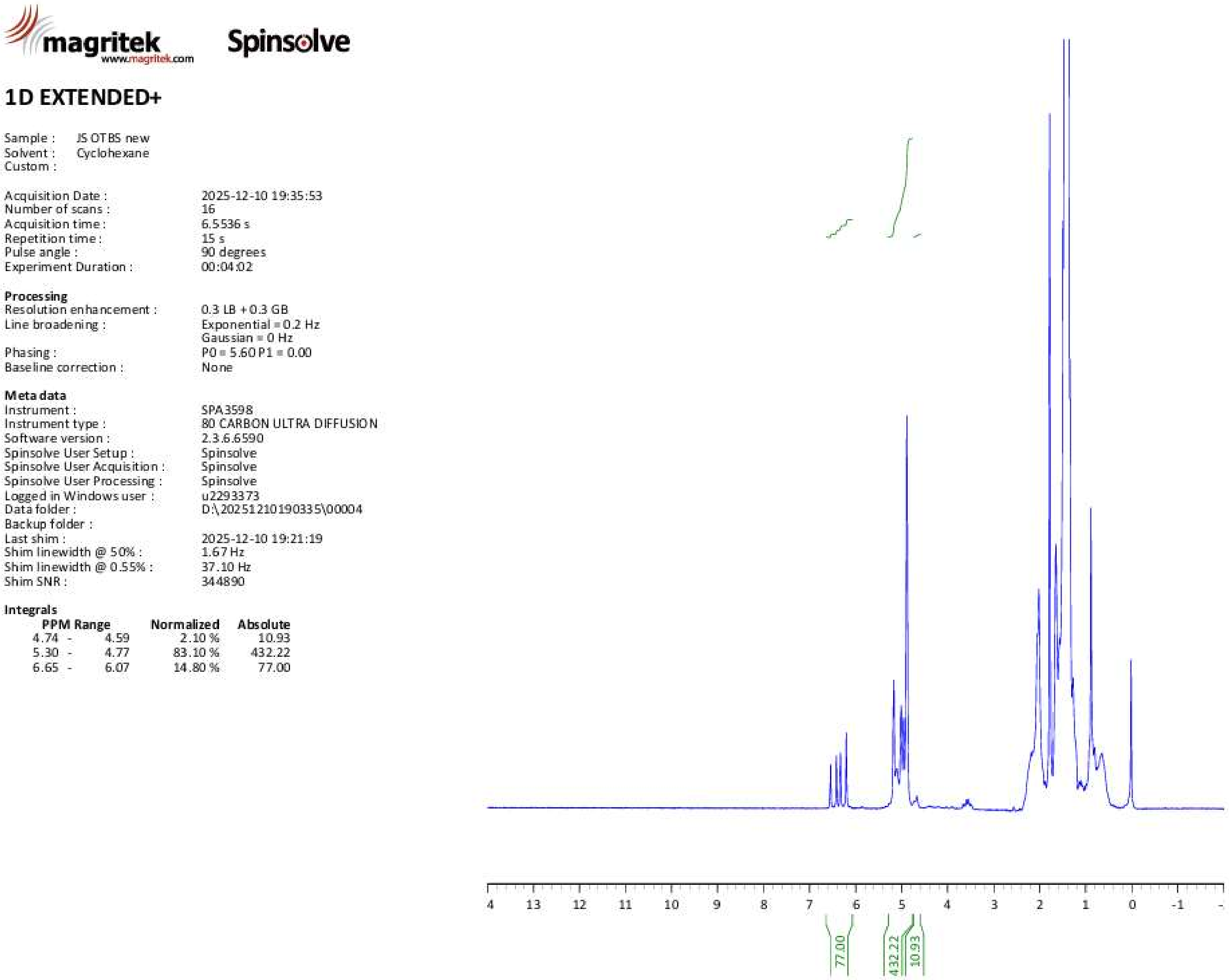

Figure S150: 60 minutes OTBS / Isoprene copolymerization ^{1}H NMR Spectra:

OTBS / Isoprene copolymerization ^{1}H NMR Spectra:

90 Minutes:

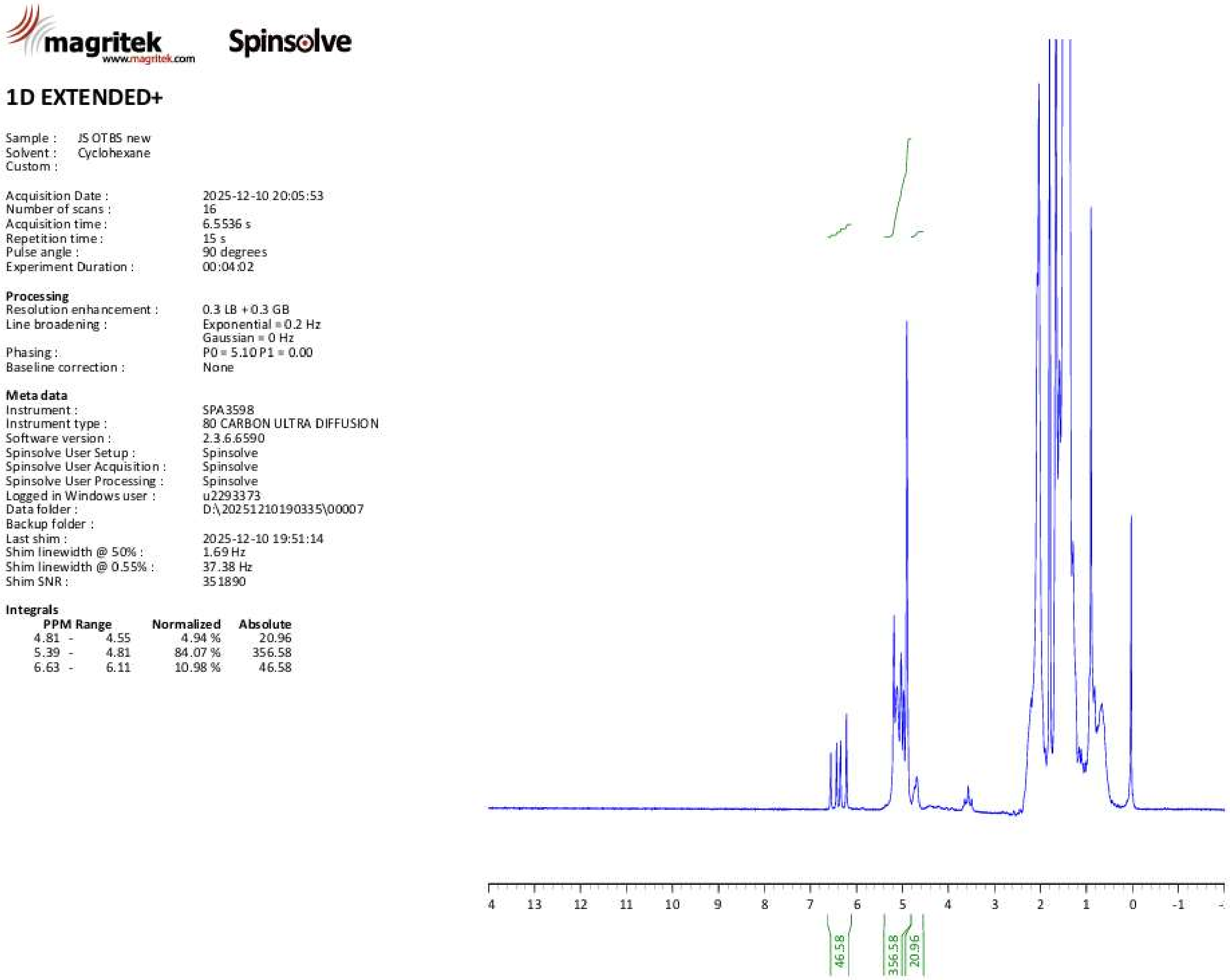

Figure S151: 90 minutes OTBS / Isoprene copolymerization ^{1}H NMR Spectra:

OTBS / Isoprene copolymerization ^{1}H NMR Spectra:

120 Minutes:

magritek www.magritek.com Spinsolve

1D EXTENDED+

Sample : JS OTBS new
Solvent : Cyclohexane
Custom :

Acquisition Date :	2025-12-10 20:35:53
Number of scans :	16
Acquisition time :	6.5536 s
Repetition time :	15 s
Pulse angle :	90 degrees
Experiment Duration :	00:04:02

Processing

Resolution enhancement :	0.3 LB + 0.3 GB
Line broadening :	Exponential = 0.2 Hz<br>Gaussian = 0 Hz
Phasing :	P0 = 5.60 P1 = 0.00
Baseline correction :	None

Meta data

Instrument :	SPA3598
Instrument type :	80 CARBON ULTRA DIFFUSION
Software version :	2.3.6.6590
Spinsolve User Setup :	Spinsolve
Spinsolve User Acquisition :	Spinsolve
Spinsolve User Processing :	Spinsolve
Logged in Windows user :	u2293373
Data folder :	D:\20251210190335\00010
Backup folder :	
Last shim :	2025-12-10 20:21:14
Shim linewidth @ 50% :	1.71 Hz
Shim linewidth @ 0.55% :	38.01 Hz
Shim SNR :	351790

Integrals

PPM Range	Normalized	Absolute
4.81 - 4.55	7.12 %	26.22
5.39 - 4.81	85.35 %	314.21
6.63 - 6.11	7.53 %	27.71

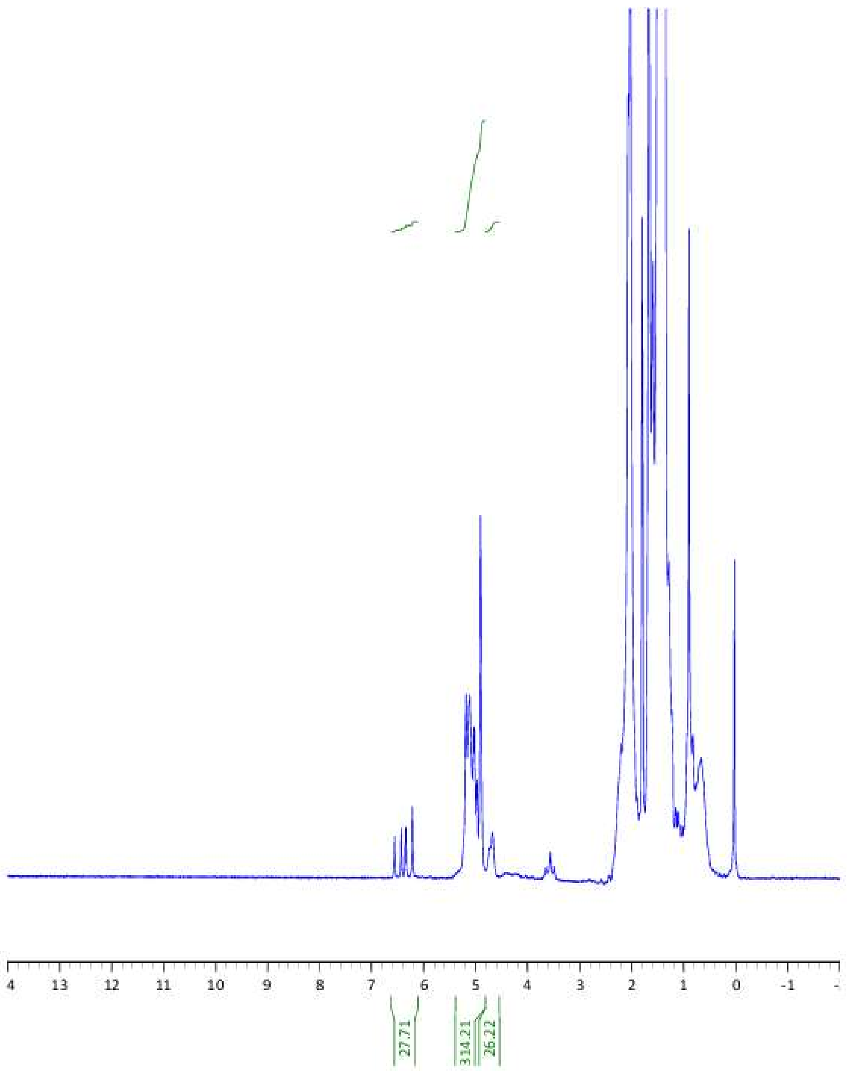

Figure S152: 120 minutes OTBS / Isoprene copolymerization ^{1}H NMR Spectra:

OTBS / Isoprene copolymerization ^{1}H NMR Spectra:

150 Minutes:

magritek www.magritek.com Spinsolve

1D EXTENDED+

Sample : JS OTBS new
Solvent : Cyclohexane
Custom :

Acquisition Date :	2025-12-10 21:05:53
Number of scans :	16
Acquisition time :	6.5536 s
Repetition time :	15 s
Pulse angle :	90 degrees
Experiment Duration :	00:04:02

Processing

Resolution enhancement :	0.3 LB + 0.3 GB
Line broadening :	Exponential = 0.2 Hz<br>Gaussian = 0 Hz
Phasing :	P0 = 5.20 P1 = 0.00
Baseline correction :	None

Meta data

Instrument :	SPA3598
Instrument type :	80 CARBON ULTRA DIFFUSION
Software version :	2.3.6.6590
Spinsolve User Setup :	Spinsolve
Spinsolve User Acquisition :	Spinsolve
Spinsolve User Processing :	Spinsolve
Logged in Windows user :	u2293373
Data folder :	D:\20251210190335\00013
Backup folder :	
Last shim :	2025-12-10 20:51:14
Shim linewidth @ 50% :	1.71 Hz
Shim linewidth @ 0.55% :	38.32 Hz
Shim SNR :	343580

Integrals

PPM Range			Normalized	Absolute
4.81	-	4.55	8.78 %	28.74
5.39	-	4.81	86.62 %	283.40
6.63	-	6.11	4.60 %	15.05

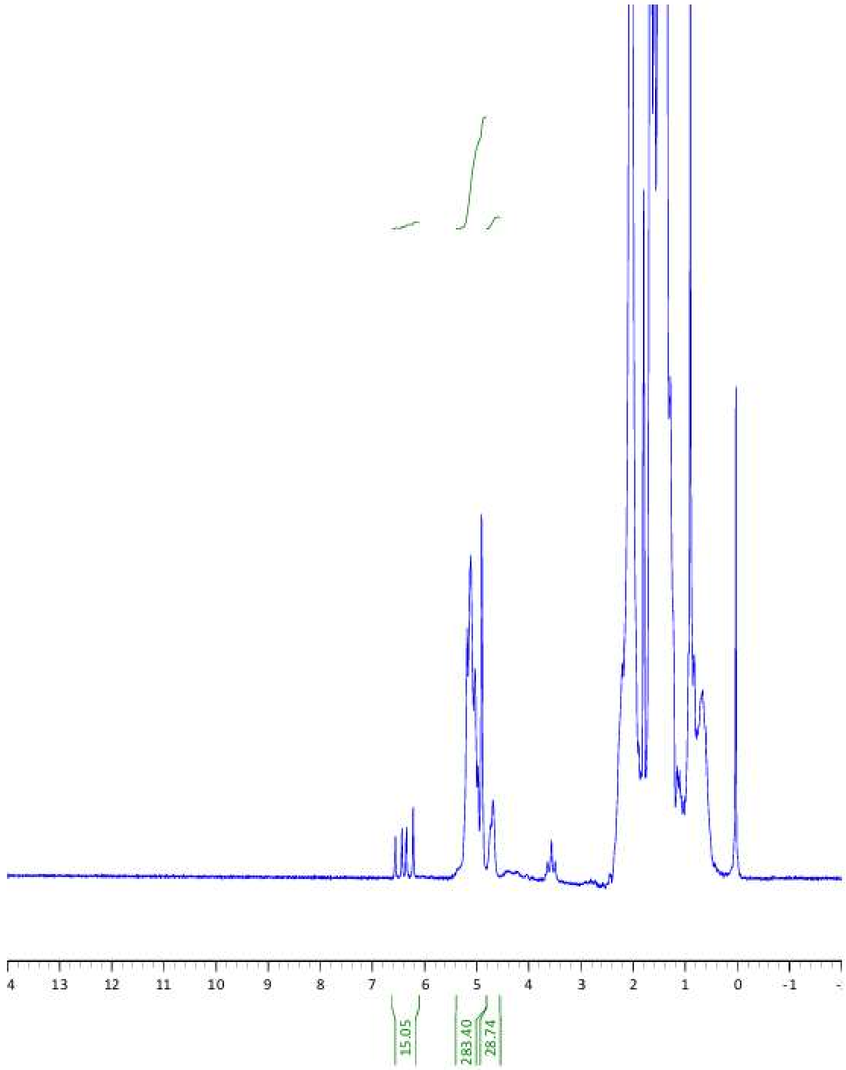

Figure S153: 150 minutes OTBS / Isoprene copolymerization ^{1}H NMR Spectra:

OTBS / Isoprene copolymerization ^{1}H NMR Spectra:

180 Minutes:

magritek www.magritek.com Spinsolve

1D EXTENDED+

Sample : JS OTBS new
Solvent : Cyclohexane
Custom :

Acquisition Date :	2025-12-10 21:35:53
Number of scans :	16
Acquisition time :	6.5536 s
Repetition time :	15 s
Pulse angle :	90 degrees
Experiment Duration :	00:04:02

Processing

Resolution enhancement :	0.3 LB + 0.3 GB
Line broadening :	Exponential = 0.2 Hz<br>Gaussian = 0 Hz
Phasing :	P0 = 5.40 P1 = 0.00
Baseline correction :	None

Meta data

Instrument :	SPA3598
Instrument type :	80 CARBON ULTRA DIFFUSION
Software version :	2.3.6.6590
Spinsolve User Setup :	Spinsolve
Spinsolve User Acquisition :	Spinsolve
Spinsolve User Processing :	Spinsolve
Logged in Windows user :	u2293373
Data folder :	D:\20251210190335\00016
Backup folder :	
Last shim :	2025-12-10 21:21:14
Shim linewidth @ 50% :	1.61 Hz
Shim linewidth @ 0.55% :	37.98 Hz
Shim SNR :	349110

Integrals

PPM Range			Normalized	Absolute
4.81	-	4.55	10.12 %	30.56
5.39	-	4.81	87.73 %	264.83
6.63	-	6.11	2.14 %	6.47

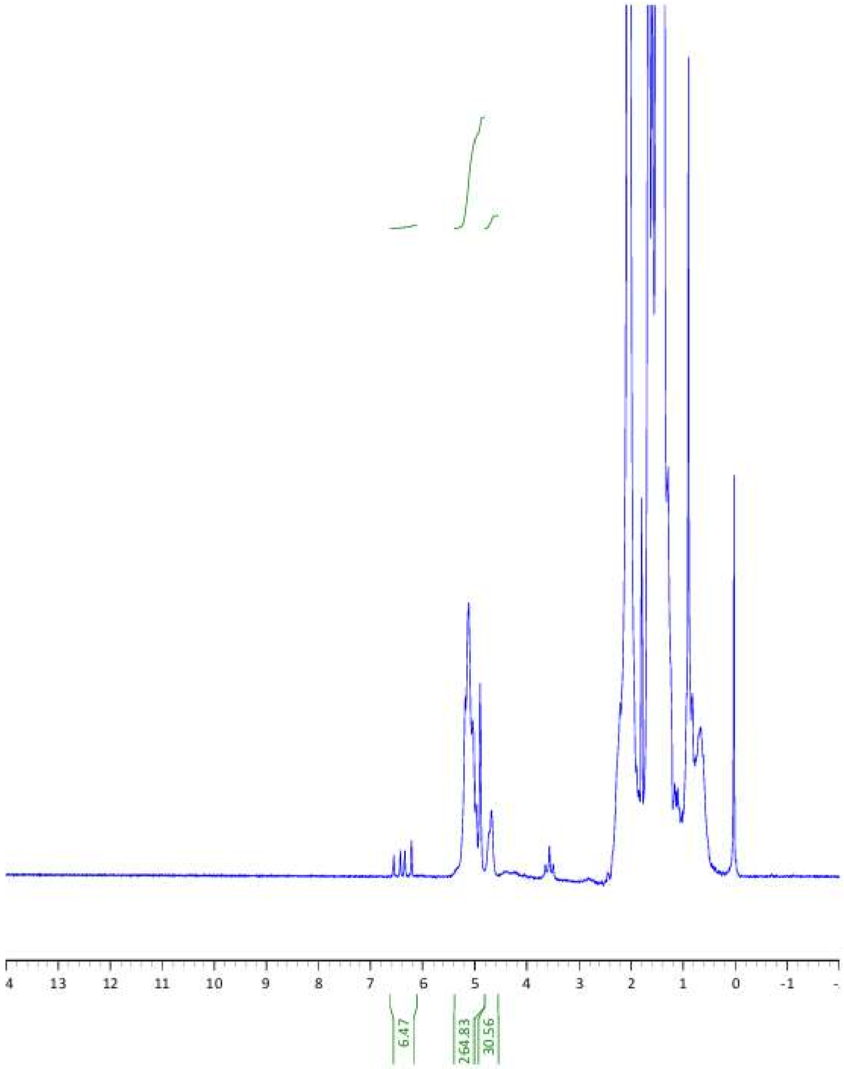

Figure S154: 180 minutes OTBS / Isoprene copolymerization ^{1}H NMR Spectra:

OTBS / Isoprene copolymerization ^{1}H NMR Spectra:

210 Minutes:

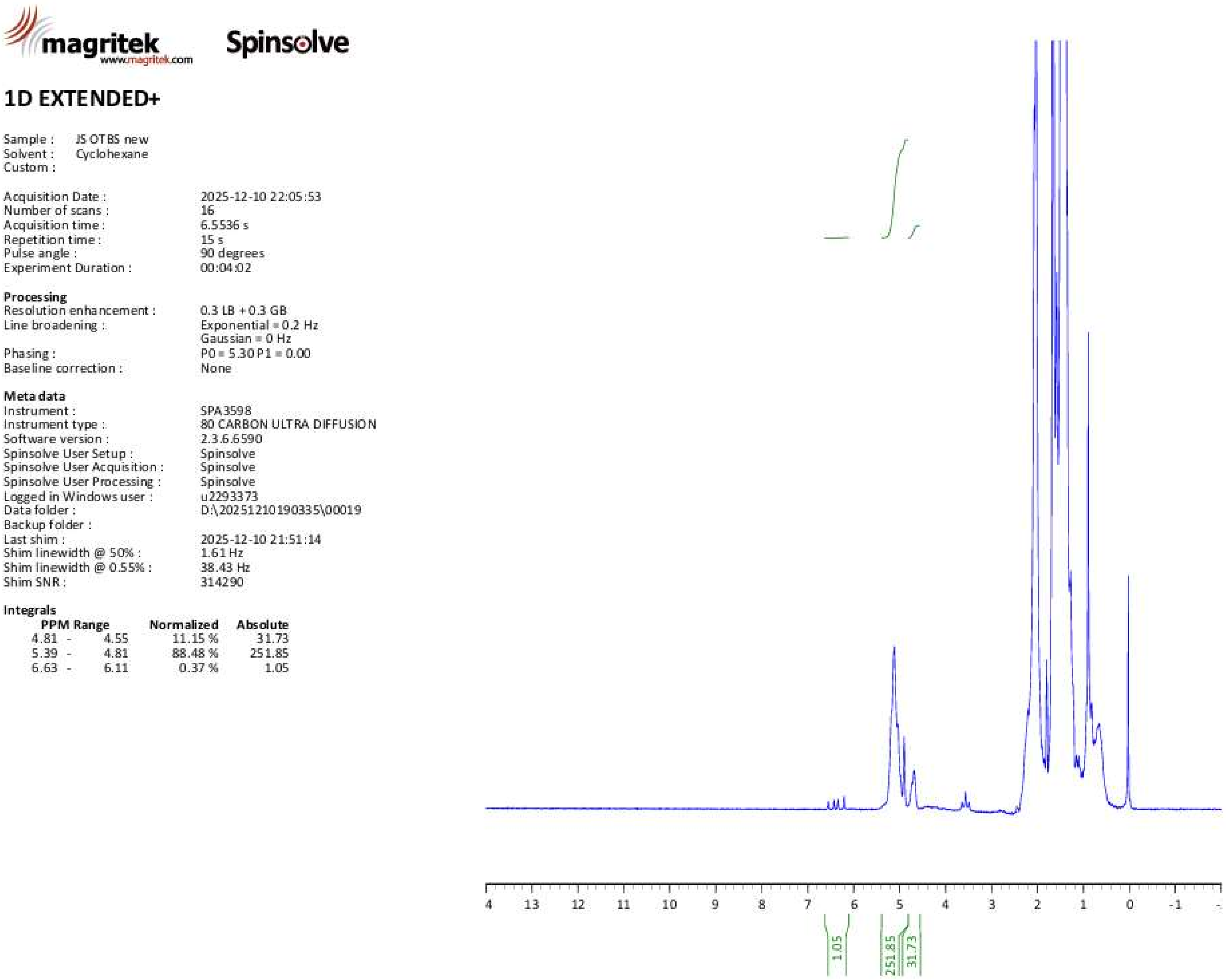

Figure S155: 210 minutes OTBS / Isoprene copolymerization ^{1}H NMR Spectra:

OTBS / Isoprene copolymerization ^1H NMR Spectra:

240 Minutes:

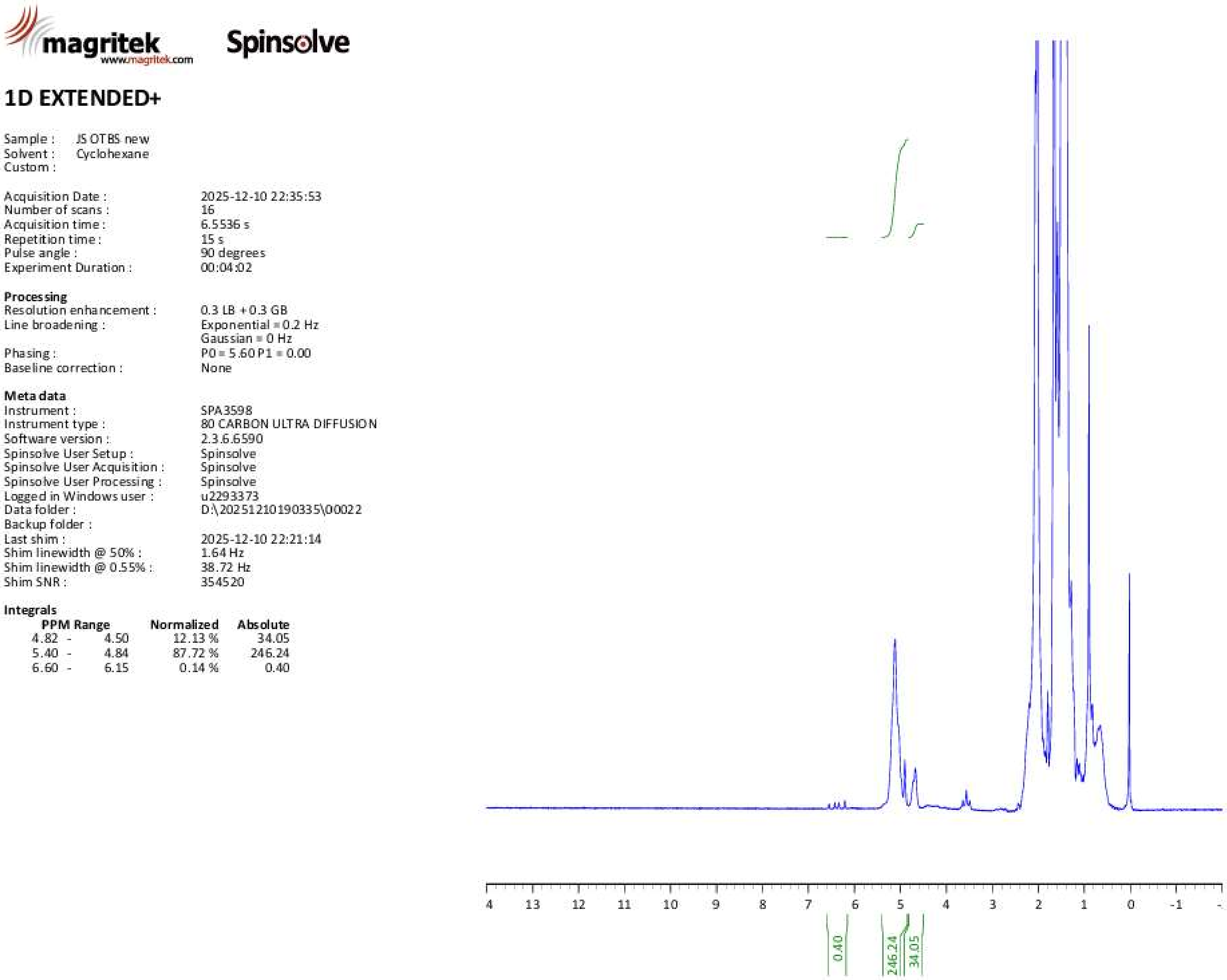

Figure S156: 240 minutes OTBS / Isoprene copolymerization ^1H NMR Spectra:

OTBS / Isoprene copolymerization ^{1}H NMR Spectra:

270 Minutes:

magritek www.magritek.com Spinsolve

1D EXTENDED+

Sample : JS OTBS new
Solvent : Cyclohexane
Custom :

Acquisition Date : 2025-12-10 23:05:53
Number of scans : 16
Acquisition time : 6.5536 s
Repetition time : 15 s
Pulse angle : 90 degrees
Experiment Duration : 00:04:02

Processing
Resolution enhancement : 0.3 LB + 0.3 GB
Line broadening : Exponential = 0.2 Hz
Gaussian = 0 Hz
Phasing : P0 = 4.90 P1 = 0.00
Baseline correction : None

Meta data
Instrument : SPA3598
Instrument type : 80 CARBON ULTRA DIFFUSION
Software version : 2.3.6.6590
Spinsolve User Setup : Spinsolve
Spinsolve User Acquisition : Spinsolve
Spinsolve User Processing : Spinsolve
Logged in Windows user : u2293373
Data folder : D:\20251210190335\00025
Backup folder :
Last shim : 2025-12-10 22:51:14
Shim linewidth @ 50% : 1.63 Hz
Shim linewidth @ 0.55% : 38.84 Hz
Shim SNR : 356640

Integrals

PPM Range	Normalized	Absolute
4.82 - 4.50	13.08 %	37.27
5.40 - 4.84	86.53 %	246.51
6.60 - 6.16	0.39 %	1.11

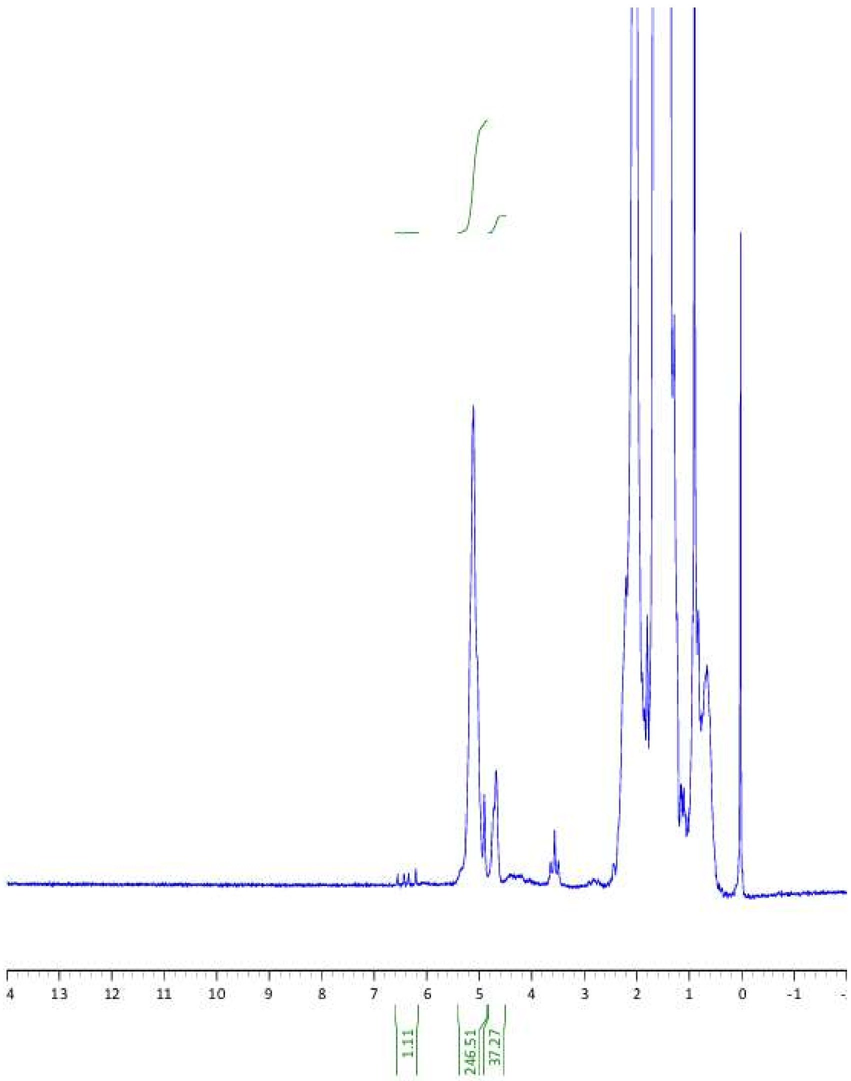

Figure S157: 270 minutes OTBS / Isoprene copolymerization ^{1}H NMR Spectra:

OTBS / Isoprene copolymerization ^{1}H NMR Spectra:

300 Minutes:

magritek www.magritek.com Spinsolve

1D EXTENDED+

Sample : JS OTBS new
Solvent : Cyclohexane
Custom :

Acquisition Date :	2025-12-10 23:35:53
Number of scans :	16
Acquisition time :	6.5536 s
Repetition time :	15 s
Pulse angle :	90 degrees
Experiment Duration :	00:04:02

Processing

Resolution enhancement :	0.3 LB + 0.3 GB
Line broadening :	Exponential = 0.2 Hz<br>Gaussian = 0 Hz
Phasing :	P0 = 5.30 P1 = 0.00
Baseline correction :	None

Meta data

Instrument :	SPA3598
Instrument type :	80 CARBON ULTRA DIFFUSION
Software version :	2.3.6.6590
Spinsolve User Setup :	Spinsolve
Spinsolve User Acquisition :	Spinsolve
Spinsolve User Processing :	Spinsolve
Logged in Windows user :	u2293373
Data folder :	D:\20251210190335\00028
Backup folder :	
Last shim :	2025-12-10 23:21:14
Shim linewidth @ 50% :	1.65 Hz
Shim linewidth @ 0.55% :	38.35 Hz
Shim SNR :	327400

Integrals

PPM Range			Normalized	Absolute
4.82	-	4.50	13.56 %	38.48
5.40	-	4.84	86.29 %	244.93
6.60	-	6.16	0.16 %	0.45

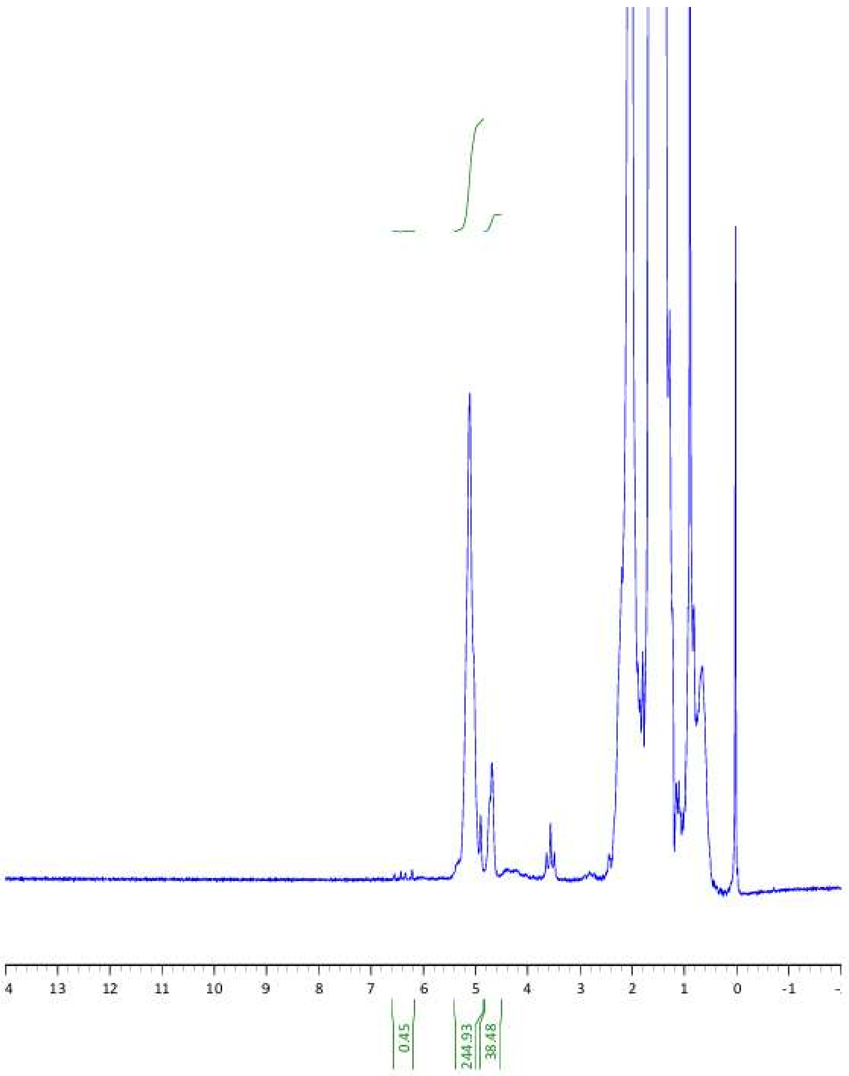

Figure S158: 300 minutes OTBS / Isoprene copolymerization ^{1}H NMR Spectra:

OTBS / Isoprene copolymerization ^1H NMR Spectra:

330 Minutes:

magritek www.magritek.com Spinsolve

1D EXTENDED+

Sample :	JS OTBS new
Solvent :	Cyclohexane
Custom :	

Acquisition Date :	2025-12-11 00:05:53
Number of scans :	16
Acquisition time :	6.5536 s
Repetition time :	15 s
Pulse angle :	90 degrees
Experiment Duration :	00:04:02

Processing

Resolution enhancement :	0.3 LB + 0.3 GB
Line broadening :	Exponential = 0.2 Hz<br>Gaussian = 0 Hz
Phasing :	P0 = 4.85 P1 = 0.00
Baseline correction :	None

Meta data

Instrument :	SPA3598
Instrument type :	80 CARBON ULTRA DIFFUSION
Software version :	2.3.6.6590
Spinsolve User Setup :	Spinsolve
Spinsolve User Acquisition :	Spinsolve
Spinsolve User Processing :	Spinsolve
Logged in Windows user :	u2293373
Data folder :	D:\20251210190335\00031
Backup folder :	
Last shim :	2025-12-10 23:51:14
Shim linewidth @ 50% :	1.67 Hz
Shim linewidth @ 0.55% :	38.98 Hz
Shim SNR :	329660

Integrals

PPM Range	Normalized	Absolute
4.82 - 4.50	13.98 %	39.90
5.40 - 4.84	85.84 %	244.92
6.60 - 6.16	0.18 %	0.50

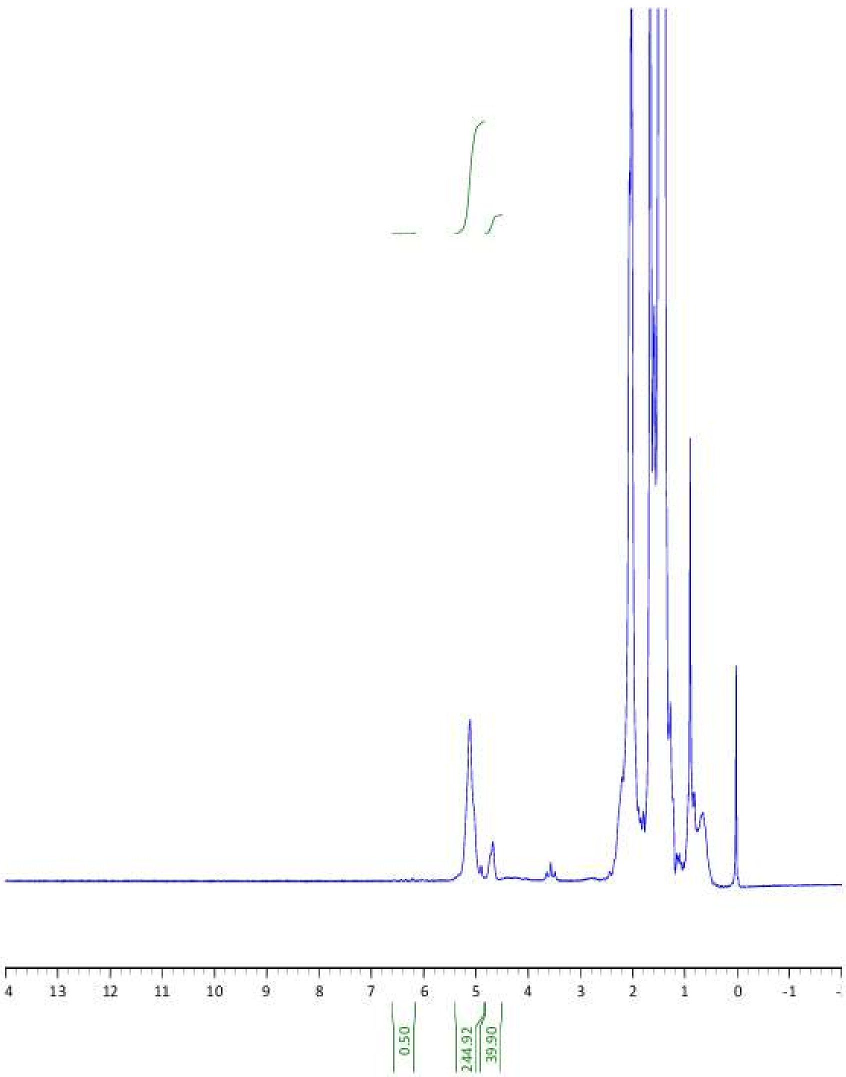

Figure S159: 330 minutes OTBS / Isoprene copolymerization ^1H NMR Spectra:

11 Plots of time vs ln([M]o/[M]t)

For each example, both the full dataset and a truncated dataset, excluding data points at very high conversion (>97.5–99.9%), are presented. Deviations from linearity at high conversion are likely attributable to baseline variations between spectra, particularly as the monomer concentration becomes very low or approaches complete consumption, creating a large error due to the low denominator value.

OBn / Isoprene Copolymerisation (Low MWt):

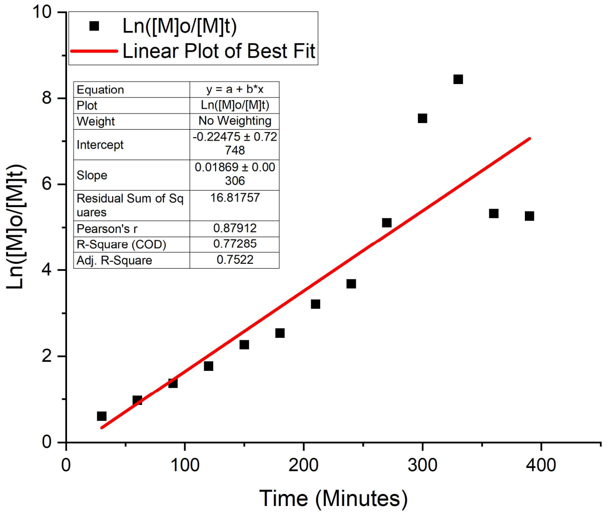

Figure S160: ln([M]o/[M]t) for OBn/Isoprene Copolymerization (Low MWt)

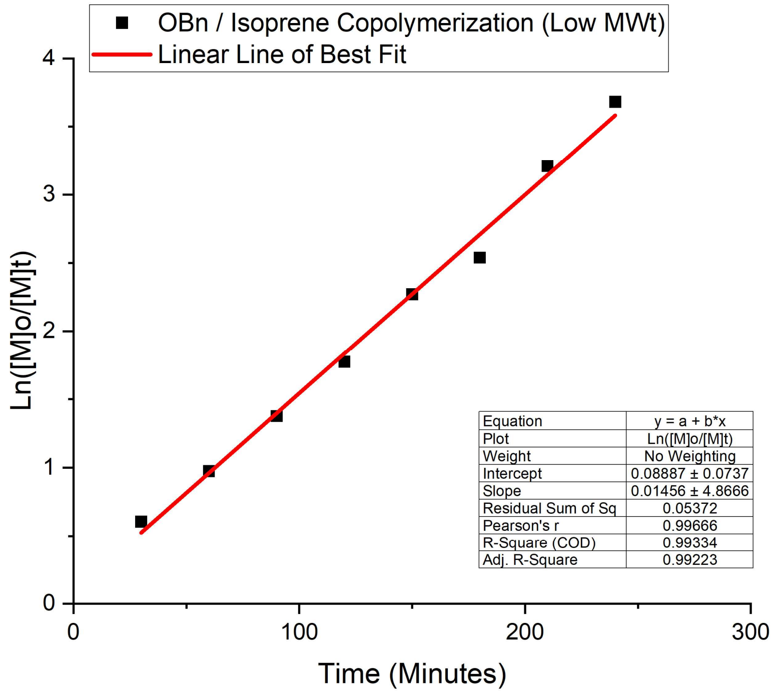

Figure S161: ln([M]o/[M]t) for OBn/Isoprene Copolymerization (Low MWt) with points >99% conversion omitted.

OBn / Isoprene Copolymerisation (High Mwt):

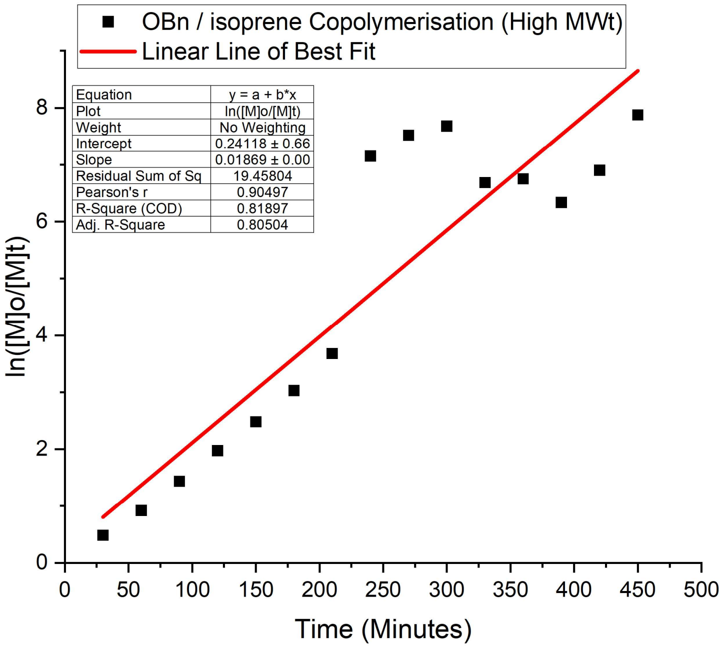

Figure S162: ln([M]o/[M]t) for OBn/Isoprene Copolymerization (High MWt)

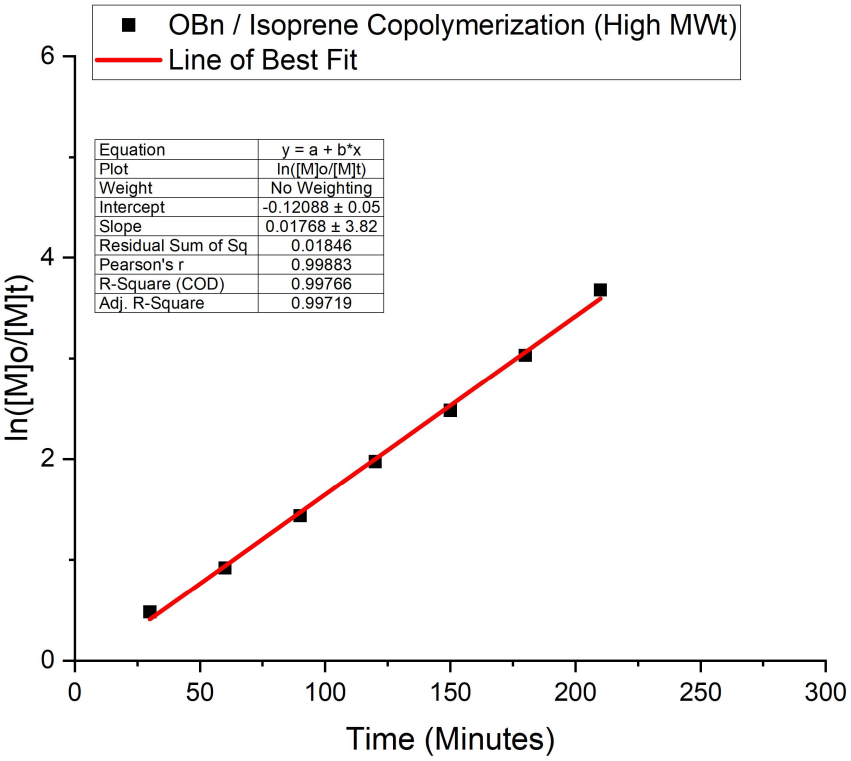

Figure S163: ln([M]o/[M]t) for OBn/Isoprene Copolymerization (High MWt) with points >99.9% conversion omitted.

OTBS / Isoprene Copolymerisation:

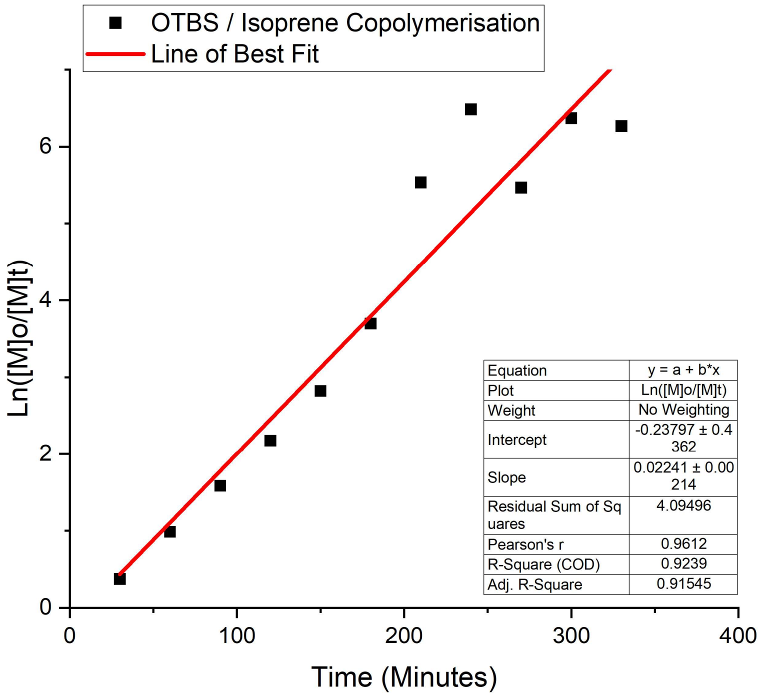

Figure S164: ln([M]o/[M]t) for OTBS/Isoprene Copolymerization

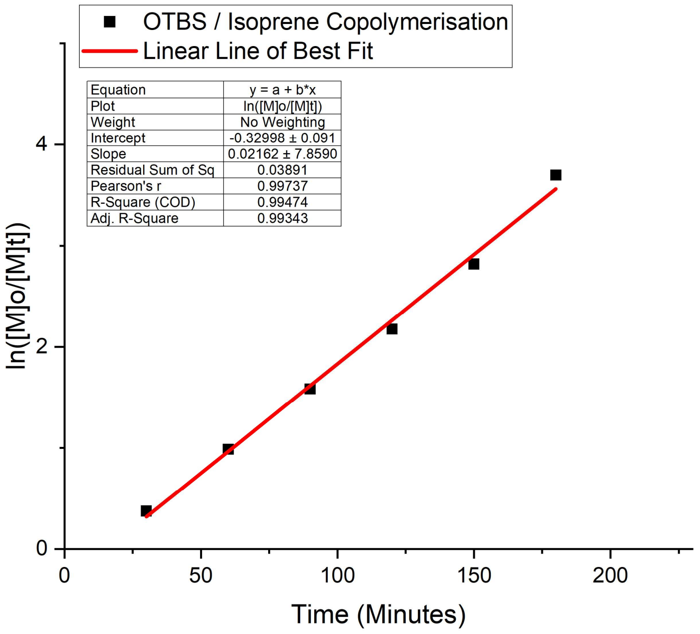

Figure S164: ln([M]o/[M]t) for OTBS/Isoprene Copolymerization with points >97.5% conversion omitted.

12 References:

(1) Wu, J. Y.; Moreau, B.; Ritter, T. Iron-Catalyzed 1,4-Hydroboration of 1,3-Dienes. *Journal of the American Chemical Society* **2009**, *131* (36), 12915-12917. DOI: 10.1021/ja9048493.

(2) Nilsson, B. L.; Overman, L. E.; Read de Alaniz, J.; Rohde, J. M. Enantioselective Total Syntheses of Nankakurines A and B: Confirmation of Structure and Establishment of Absolute Configuration. *Journal of the American Chemical Society* **2008**, *130* (34), 11297-11299. DOI: 10.1021/ja804624u.

(3) Rodina, D.; Vaith, J.; Paradine, S. M. Ligand control of regioselectivity in palladium-catalyzed heteroannulation reactions of 1,3-Dienes. *Nature Communications* **2024**, *15* (1), 5433. DOI: 10.1038/s41467-024-49803-y.

(4) Tooley, O.; Pointer, W.; Radmall, R.; Hall, M.; Beyer, V.; Stakem, K.; Swift, T.; Town, J.; Junkers, T.; Wilson, P.; et al. MaDDOSY (Mass Determination Diffusion Ordered Spectroscopy) using an 80 MHz Bench Top NMR for the Rapid Determination of Polymer and Macromolecular Molecular Weight. *Macromolecular Rapid Communications* **2024**, *45* (8), 2300692. DOI: https://doi.org/10.1002/marc.202300692 (acccessed 2025/10/07).

(5) Wahlen, C.; Rauschenbach, M.; Blankenburg, J.; Kersten, E.; Ender, C. P.; Frey, H. Myrcenol-Based Monomer for Carbanionic Polymerization: Functional Copolymers with Myrcene and Bio-Based Graft Copolymers. *Macromolecules* **2020**, *53* (20), 9008-9017. DOI: 10.1021/acs.macromol.0c01734.

(6) Wang, H.; Yang, Y.; Nishiura, M.; Hong, Y.-l.; Nishiyama, Y.; Higaki, Y.; Hou, Z. Making Polyisoprene Self-Healable through Microstructure Regulation by Rare-Earth Catalysts. *Angewandte Chemie International Edition* **2022**, *61* (42), e202210023. DOI: https://doi.org/10.1002/anie.202210023 (acccessed 2025/12/15).

(7) Asandei, A. D.; Simpson, C. P.; Yu, H. S.; Adebolu, O. I.; Saha, G.; Chen, Y. Cp₂TiCl-Mediated Controlled Radical Polymerization of Isoprene Initiated by Epoxide Radical Ring Opening. In *Controlled/Living Radical Polymerization: Progress in RAFT, DT, NMP & OMRP*, ACS Symposium Series, Vol. 1024; American Chemical Society, 2009; pp 149-163.
